# Synthesis and Reactions of Hydrazine Trifluoroborate Salts

**DOI:** 10.1021/acs.orglett.6c02953

**Published:** 2026-07-27

**Authors:** Lorenzo Ballerini, Wenhao Zhao, Anika Tarasewicz, María Méndez, Joseph P. A. Harrity

**Affiliations:** † The University of Sheffield, Sheffield, S3 7HF, U.K.; ‡ Integrated Drug Discovery, R&D, Sanofi Aventis DeutschlandGmbH, 65926 Frankfurt am Main, Germany

## Abstract

We report a new class of hydrazine trifluoroborate salts that undergo chemoselective reactions to deliver a range of new functionalized building blocks for organic synthesis. In particular, we show that a wide range of pyrazole *N*-methylboronates can be prepared by condensation reactions with 1,3-dicarbonyl compounds and conjugated ynones with high regiocontrol. Finally, the synthetic potential of these pyrazole derivatives is demonstrated by S_*E*_Ar, three-component alkyl Petasis Boron–Mannich reaction, and through the synthesis of palovarotene analogs.

## Graphical abstract

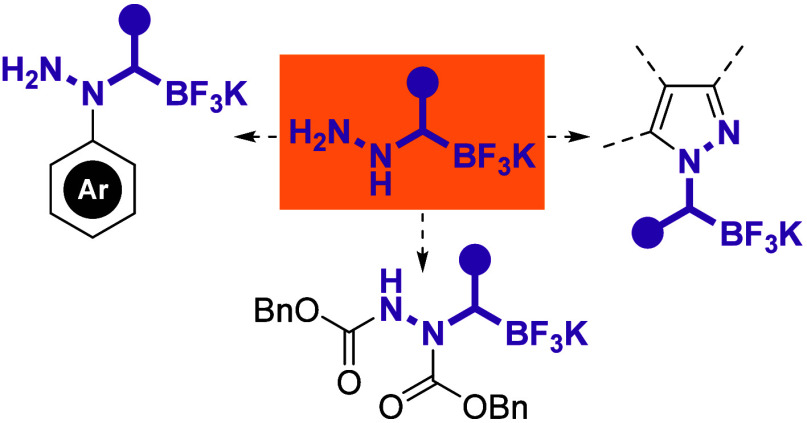

Organotrifluoroborate salts are now established as high value synthetic intermediates due to their ease of use and wide-ranging reactivity.^1^ In addition, the stability of this motif toward oxidation and protodeborylation offers the enticing opportunity to combine them with additional functional groups thereby offering routes to new tailored organoboron products. In this regard, Molander has exploited potassium halomethyltrifluoroborate salts for the synthesis of alkoxymethyl-,^2^ aminomethyl-^3^ and azidomethyl-borate^4^ intermediates, while in our laboratories we have established ynone trifluoroborates^5^ as useful substrates for the synthesis of a series of (hetero)­aromatic boronic acid derivatives.

We envisaged that a novel hydrazine based trifluoroborate salt could offer significant opportunities with respect to the synthesis of nitrogen containing compounds.^6^ As shown in Scheme 1, such a reagent would provide a platform for the synthesis of functionalized hydrazines and *N*-heteroaromatic products that could be further elaborated by exploiting the rich chemistry of organoboron reagents. Accordingly, we set about exploring the synthesis and reactivity of *N*-alkyl trifluoroborate hydrazine salts.

**Scheme 1:**
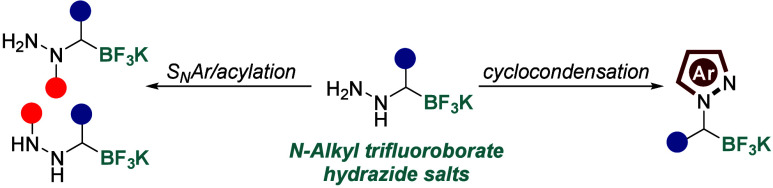
Strategies for Elaborating Hydrazine Trifluoroborate Salts

We began by exploring the reaction of hydrazine with the commercially available potassium bromomethyltrifluoroborate salt **1a**. The use of hydrazine hydrate in the presence of triethylamine successfully generated the corresponding hydrazine trifluoroborate salt **2**. However, purification of this compound required a long and tedious work up procedure involving azeotropic removal of water with toluene and several iterations of precipitating the salt from methanol/ether mixtures. Hydrazine can also be purchased as a 1 M solution in THF which offered a means of obviating the water removal steps. Pleasingly, this procedure produced clean product reproducibly on gram scale after just one or two precipitation steps with high yield and within a much shorter purification protocol. This chemistry could be extended to include an α-methyl hydrazine salt **3**, and both products were found to be air stable solids that could be stored for several weeks without any noticeable degradation (Scheme 2).

**Scheme 2:**
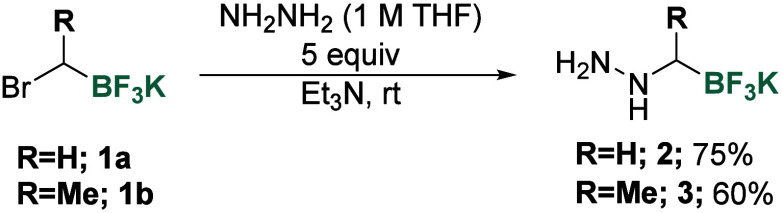
Synthesis of Hydrazine Trifluoroborate Salts

Characterization of **2** by ^19^F NMR spectroscopy in D_2_O showed the expected quartet at −142 ppm together with a smaller quartet at −140 ppm that we attributed to dialkylation byproduct which accounted for <5% of the sample. However, the spectrum also indicated a sharp singlet at −122 ppm which increased in intensity upon addition of KF allowing us to identify this as fluoride anion. While the concentration of fluoride varied somewhat batch to batch, its concentration was significant and typically constituted 50–60% of the sample. Finally, we were able to grow suitable crystals of **2** which confirmed that the hydrazine was isolated (at least in part) as the HF adduct (Scheme 3).

**Scheme 3:**
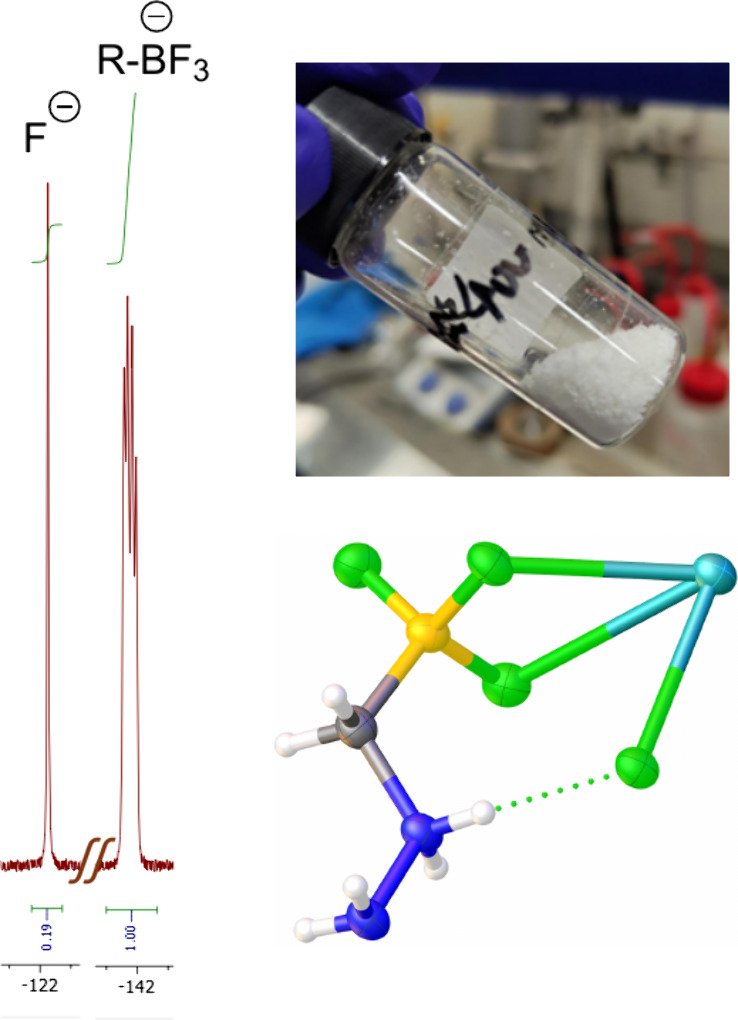
Solution and Solid-State Characterization of **2**

With a reproducible and scalable route to the hydrazine trifluoroborate salts in hand, we began preliminary investigations into their reactivity. We explored the potential for these compounds to undergo cross-coupling but were disappointed to find that this transformation could not be successfully implemented despite extensive screening of catalyst and conditions (Scheme 4, eq (1)). However, we were pleased to find that **2** underwent smooth acylation with Cbz-Cl allowing **4** to be isolated as a bench stable colorless solid. Furthermore, this compound successfully participated in Pd-catalyzed coupling using conditions developed by Molander^7^ to deliver the corresponding hydrazide **5** in good yield (eq (2)). We were also able to prepare *N*-aryl hydrazine **6** via an S_*N*_Ar reaction with 2,4-dinitro fluorobenzene (eq (3)). Taken together, these preliminary transformations highlighted the potential of hydrazine trifluoroborate salts to undergo chemoselective functionalization at nitrogen and boron, with the reaction shown in eq (3) indicating enhanced nucleophilicity at the secondary *N*-atom.

**Scheme 4:**
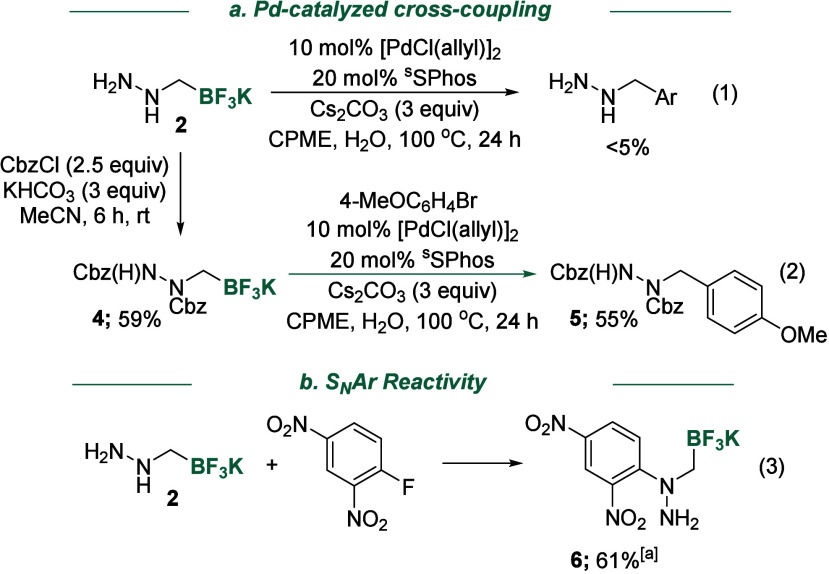
Preliminary Reactivity Study of Hydrazine **2**

a Yield estimated by NMR spectroscopy relative to 1,3,5-(MeO)_3_C_6_H_3_ standard.

Nitrogen based heterocycles are ubiquitous in the chemical sciences and constitute small molecule pharmaceuticals, agrochemicals as well as functional materials. With specific regard to pharmaceuticals, there is a growing interest in the use of pyrazoles in drug discovery^8^ and these compounds are represented in the top 10 of most frequent nitrogen heterocycles in FDA approved drugs in the past 10 years.^9^ We became particularly interested in *N*-benzyl substituted pyrazoles as a prominent subclass of these heteroaromatic systems. These compounds offer a broad range of biological activities (Scheme 5a) and therefore methods to access these substructures are highly sought after. These compounds are normally synthesized by the *N*-alkylation of the parent pyrazoles, and this can lead to regioselectivity issues in unsymmetrical pyrazole substrates.^10^ Moreover, the required alkylating agents are often not commercially available, and as established mutagens, their synthesis and handling can be relatively onerous.

**Scheme 5:**
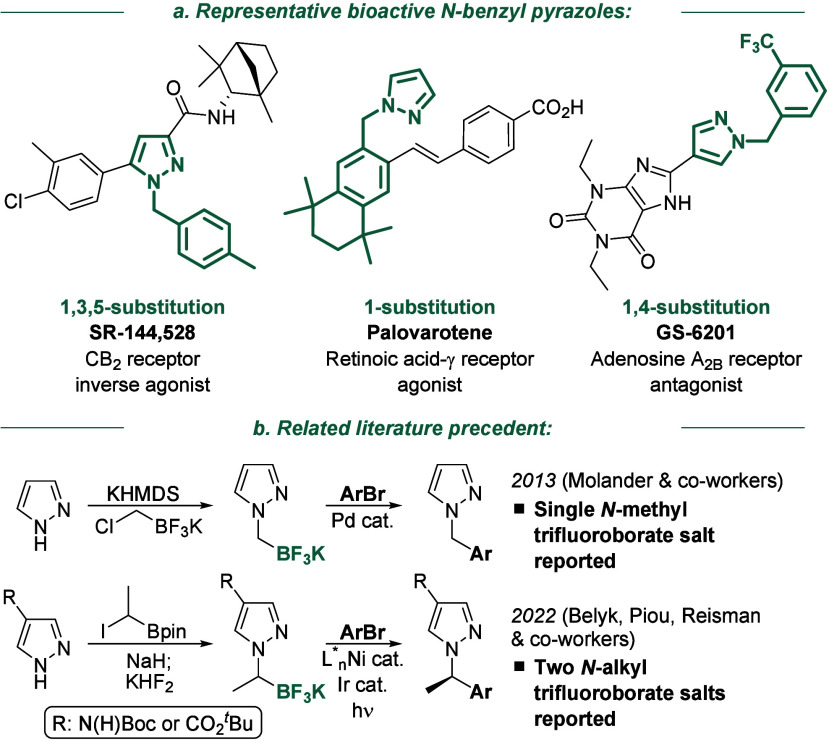
Synthetic Routes to N-Benzylpyrazoles and Their Use As Pharmaceuticals

We anticipated that it would be feasible to access these compounds through the cross-coupling of pyrazole *N*-methyltrifluoroborate salts, that would in turn be accessible from the hydrazines **2** and **3** through condensation reactions. A search of the literature highlighted a paucity of available methods to access these compounds, with those that had been devised offering a narrow scope of exemplars (Scheme 5b).^7,11^ Accordingly, we investigated the potential of hydrazine trifluoroborate salts to access these heterocyclic products.

As outlined in Scheme 6, the reaction of **2** with symmetrical 1,3-diketones smoothly generated the corresponding 3,5-disubstituted pyrazole *N*-methyltrifluoroborate salts, although the reactions evidently slowed as more sterically hindered substrates were used. The chemistry was successfully extended to trisubstituted diketones to generate 3,4,5-trisubstituted pyrazole salts with **12** and **13** bearing a distal ester group representing an attractive difunctional scaffold. We next explored the use of unsymmetrical diketones to evaluate the regioselectivity of the condensation reaction, and in these cases the pyrazoles were characterized as regioisomeric mixtures. As expected, pyrazoles **14–16** gave poor levels of regiocontrol while **17** and **18** showed that significant steric and electronic differences in the substrate substituents could deliver useful regioselectivities. We explored the potential of this method to make bicyclic pyrazoles and were pleased to find that both **19** and **20** could be successfully prepared. Notably in this case, the reactions required both an acid catalyst and excess of hydrazine salt to achieve full conversion of the starting materials and the products were isolated as α-ammoniumtrifluoroborate internal salts^12^ whose structures were confirmed by X-ray crystallographic analysis. Finally, ketoaldehydes/dialdehydes also reacted effectively with the products isolated in good to high yields, with **23** and **24**, offering further synthetic versatility through the ester functionality. Overall, hydrazine salts **2** and **3** proved to be generally effective precursors to pyrazole *N*-methyltrifluoroborate salts, offering these products with a range of substitution patterns.

**Scheme 6:**
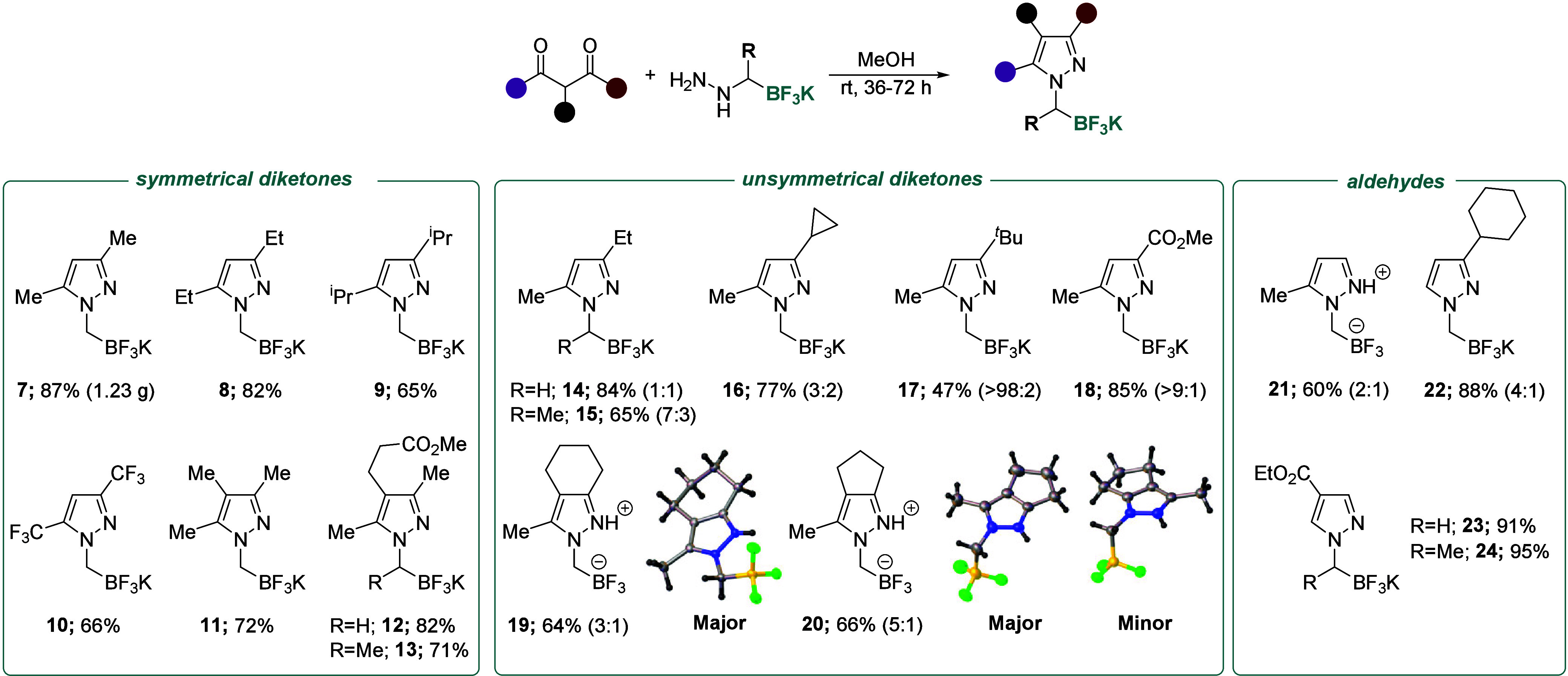
Scope of Pyrazole Synthesis from 1,3-Dicarbonyl Substrates

We envisaged that ynones could address the limitations posed by sterically similar unsymmetrical 1,3-dicarbonyl substrates with regard to regiocontrol, and we set out to explore the condensation reaction of our hydrazine salts with these compounds. As shown in Scheme 7, hex-3-yn-2-one generated pyrazole **25** with significantly improved regioselectivity as compared to the corresponding reaction with hexan-2,4-dione (c.f. **14** in Scheme 6). Isomeric pyrazoles **26** and **27** could be generated with useful levels of regiocontrol, and the method could be extended to pyrazoles **28–30** with excellent regioselectivities being observed in each case. These studies revealed a clear pattern of reactivity that allows synthetic routes to be designed with predictable regiocontrol; the secondary *N*-atom of the hydrazine salt undergoing conjugate addition to the alkyne while the primary amine moiety condenses on the ketone.

**Scheme 7:**
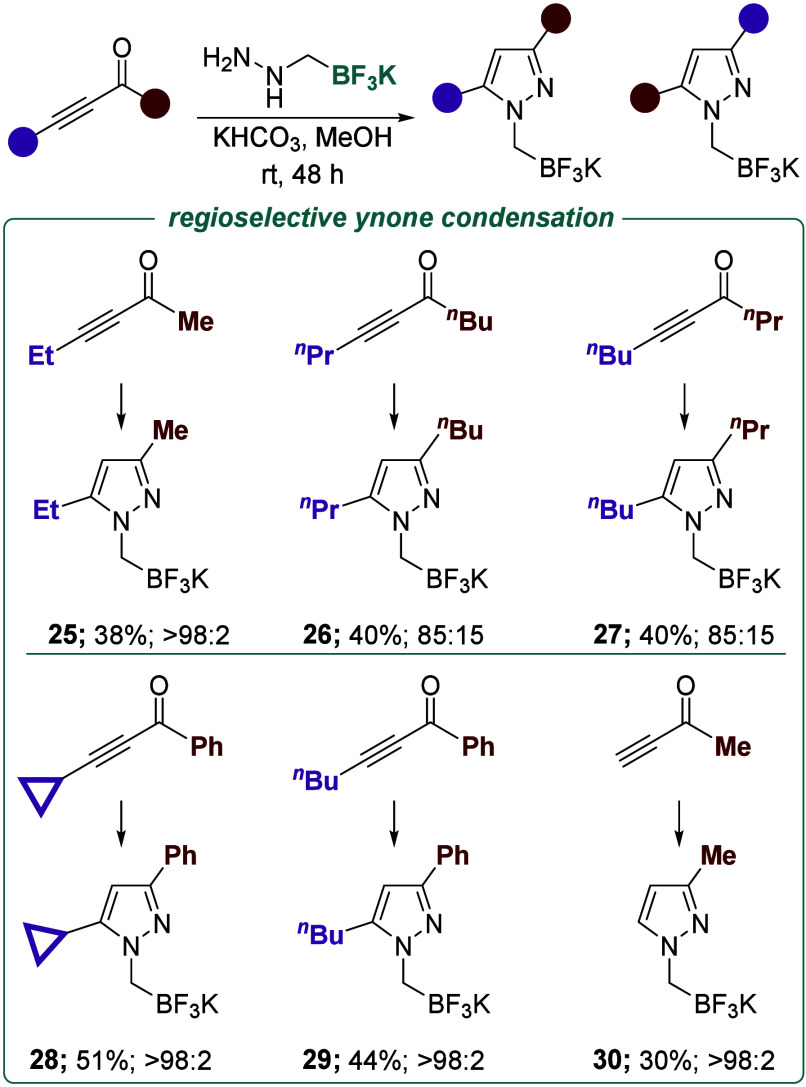
Scope of Pyrazole Synthesis from Ynone Substrates

With a robust method for the synthesis of *N*-pyrazolylmethyltrifluoroborates in hand, we wanted to confirm the potential of these intermediates in further downstream chemistry. As shown in Scheme 8, we were able to prepare bifunctional intermediate **31** by bromination of the pyrazole ring (eq 1). This reaction proceeded smoothly without impacting the trifluoroborate moiety providing testament to the tolerance of this group to oxidizing reagents. In addition, **7** underwent a light promoted three-component alkyl Petasis Boron–Mannich reaction^13^ to generate morpholine derivative **32**. Regarding our primary objective to devise a new approach to *N*-benzyl substituted pyrazoles, we took the opportunity to exploit this method for the synthesis of the palovarotene scaffold. Palovarotene (Sohonos) is currently prescribed for the treatment of heterotopic ossification and fibrodysplasia ossificans progressiva.^14^ We envisaged that the *N*-pyrazolylmethyl-trifluoroborate salts would offer a convenient and regiocontrolled means for generating pyrazole analogs of this compound by means of a late-stage cross-coupling step. In the event, we were able to rapidly assemble the required stilbene core structure and pleasingly, this underwent Pd-catalyzed coupling with pyrazoles **17** and **28** to generate palovarotene derivatives **35** and **36**.

**Scheme 8:**
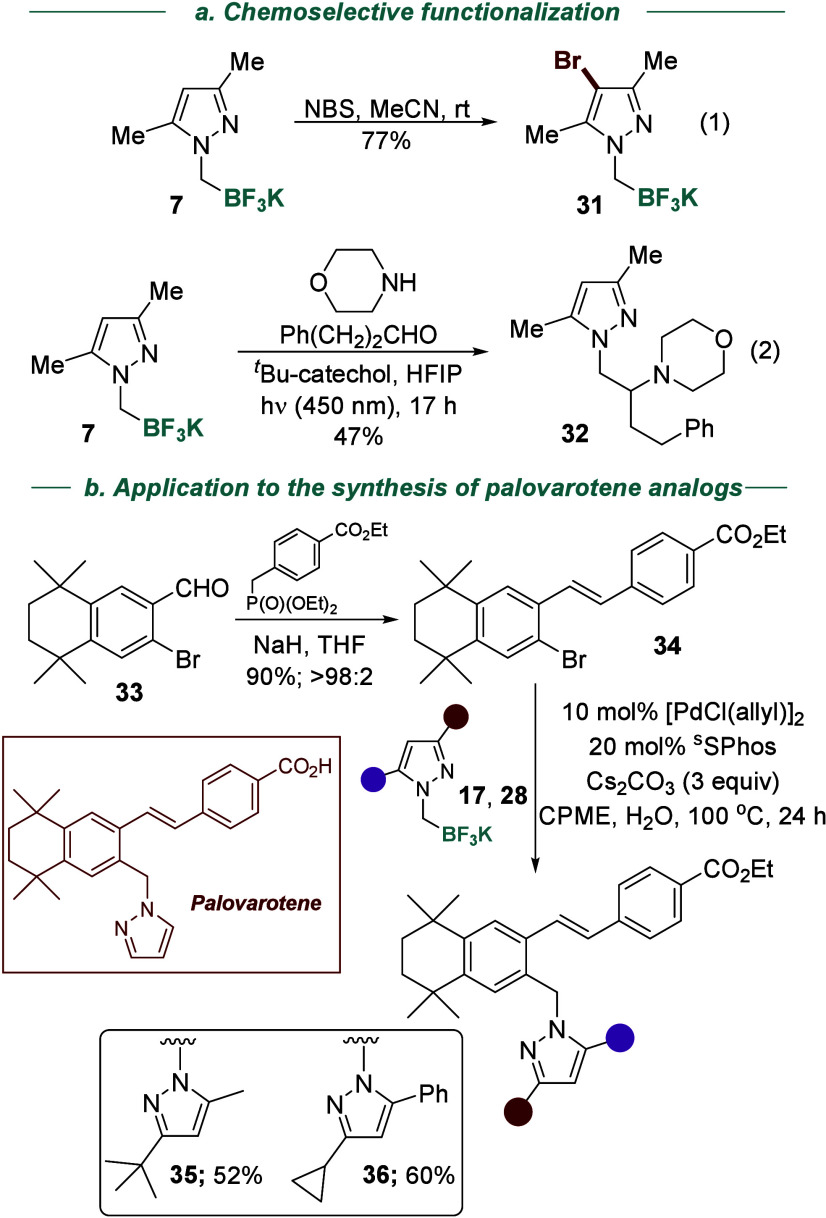
Employment of Trifluoro­(*N*-methyl)­pyrazolo)­borates in Synthesis

In conclusion, we describe a novel class of hydrazine trifluoroborate salts that are easily prepared on gram scale as air stable free-flowing solids. These compounds can be exploited through the high nucleophilicity of the hydrazine moiety while maintaining the trifluoroborate for downstream chemistry. These compounds allow useful pyrazole building blocks to be generated with regiocontrol, offering new opportunities to produce a range of analogs of these high value heterocycles. Further investigation of the synthetic potential of this class new class of reagents is underway and will be reported in due course.

## Supplementary Material



## Data Availability

The data underlying this study are available in the published article and its Supporting Information.

## Notes

Experimental procedures and characterization of the compounds obtained (PDF)
