# Supplementary material for: Synthesis and Reactions of Hydrazine Trifluoroborate Salts
Source: Org Lett. 2026 Jul 27;28(31):10134–8. doi: 10.1021/acs.orglett.6c02953 (PMC13459557; doi:10.1021/acs.orglett.6c02953)
Supplement: Supplementary file 1 [file ol6c02953_si_001.pdf]

Supporting Information

Synthesis and Reactions of Hydrazine Trifluoroborate Salts

Lorenzo Ballerini,[ǂ,#] Wenhao Zhao,[ǂ] Anika Tarasewicz,[#] María Méndez,[#] and Joseph P. A. Harrity*,[ǂ]

[ǂ]Division of Chemistry, School of Mathematical and Physical Sciences, University of Sheffield, Sheffield, S3 7HF, U.K.

[#] Integrated Drug Discovery, R&D, Sanofi Aventis Deutschland GmbH, 65926 Frankfurt am Main, Germany.

j.harrity@sheffield.ac.uk

Table of Contents

**General Information**	**S3**
**Experimental Section**	**S4**
**NMR Spectra**	**S23**
**Regiochemical Assignments**	**S86**
**X-ray crystallography Data**	**S109**

General Information

Unless otherwise indicated, all reactions were conducted using inert atmosphere conditions using solvents as obtained directly from the suppliers. A DrySyn block combined with a temperature probe was used as the heating source, where required.

Bruker AVI HD 400 (400 MHz), Bruker AVI 400 (400 MHz), or Bruker AMX-400 (400 MHz) were used to record the ^{1}H spectra data. Chemical shift values (δ) are given in ppm relative to the residual solvent for ^{1}H NMR ($CDCl_3$: δ 7.26 ppm, MeOD-d^4: δ 3.31 ppm, DMSO-d^6: δ 2.50 ppm) and relative to internal resonance for ^{13}C NMR ($CDCl_3$: δ 77.16 ppm, MeOD-d^4: δ 49.00 ppm, DMSO-d^6: δ 39.52 ppm). In experimental results, the data are presented as follow: chemical shift, multiplicity (s = singlet, d = doublet, t = triplet, q= quartet, br = broad, m = multiplet), coupling constant (Hz), integration. ^{13}C NMR signals for boronate compounds are generally incomplete, with the carbon directly attached to boron not being observed due to the quadrupole effect at boron. Bruker AVI HD 400 was used to record ^{19}F NMR (376.5 MHz) and ^{11}B NMR (128 MHz) spectra.

High-resolution mass spectra (HRMS) recorded for accurate mass analysis, were performed on a Micromass LCT operating in electrospray mode (TOF, ESI+, ESI–). Aluminium-backed plates pre-coated with silica (0.2 mm, Merck 60 F254) are used to perform thin layer chromatography (TLC). They were developed by standard visualizing agents: potassium permanganate or UV light. Silica gel (Merck 40-63 µm) or Florisil® (60-100 mesh) were used to perform flash chromatography. Gallenkamp melting point apparatus was used to record melting points and these are uncorrected.

Synthesis of Hydrazide Salts

Potassium trifluoro(*N*-methylhydrazino)borate (2)

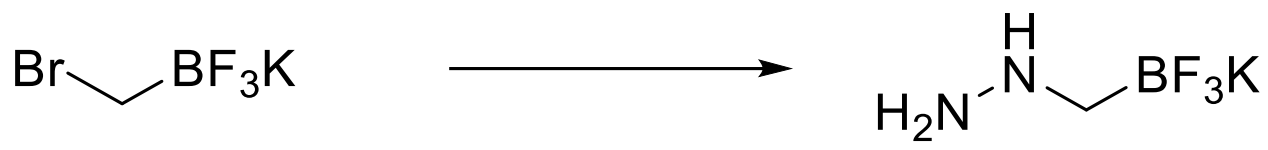

Hydrazine in THF (1 M, 50 mL, 50 mmol, 5 equiv.; **CARE:** toxic, can form explosive vapor-air mixtures), triethylamine (2.8 mL, 20 mmol, 2 equiv.) and potassium trifluoro(*N*-bromomethyl)borate **1a** (2 g, 10 mmol, 1 equiv.) were solubilized in MeOH (80 mL). The reaction mixture was left to stir overnight and then concentrated *in vacuo*. The crude product was purified by dissolving in the minimum of methanol and precipitating the product with diethyl ether to afford the title compound **2** (1.14 g, 75% yield). It was sometimes necessary to perform a second reprecipitation to obtain the desired product free of hydrazine contamination.

^{1}H NMR (400 MHz, MeOD-d^4) δ 2.19 (q, *J* = 5.5 Hz, 2H, CH_2); **^{1}H NMR (400 MHz, DMSO-d^6)** δ 1.91 (q, *J* = 5.5 Hz, 2H, CH_2); **^{19}F NMR (377 MHz, MeOD-d^4)** δ -145.9 (q, *J* = 53.0 Hz); **^{19}F NMR (377 MHz, DMSO-d^6)** δ -139.3 (q, *J* = 53.0 Hz); **^{11}B NMR (128 MHz, MeOD-d^4)** δ 2.30 (q, *J* = 53.0 Hz); **^{11}B NMR (128 MHz, DMSO-d^6)** δ 2.17 (q, *J* = 53.0 Hz).

HRMS (ESI$^-$) $CH_5N_2BF_3^-$ requires (M-K)$^-$ 113.0498, found 113.0499.

M.P. 130-133 °C.

Potassium 1-trifluoro(bromoethyl)borate (1b)

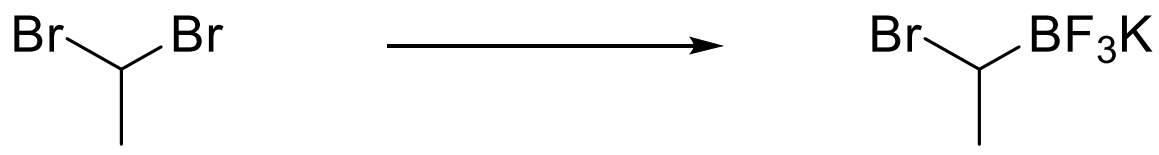

To a solution of dibromoethane (1.76 mL, 25 mmol) and triisopropylborate (5.2 mL, 23 mmol) in THF (35 mL) was added n-BuLi (2.5 M soln in hexanes, 8.5 mL, 21 mmol) dropwise at -78 °C under N_2. The solution was vigorously stirred for 1 h. KHF_2 (4.9 g, 63 mmol) was added in one portion, followed by the dropwise addition of H_2O (15 mL). The reaction mixture was warmed to rt and left stirring overnight. The suspension was then concentrated under reduced pressure and the resulting solid was suspended in hot acetone and filtered through Celite. The filtrate was concentrated and the resulting solid was washed with DCM to remove residual dibromoethane to afford the product **1b** as a white amorphous powder (2.7 g, 60% yield).

^{1}H NMR (400 MHz, MeOD-d^4) δ 3.20 (br, 1H, CH), 1.55 (d, *J* = 7.5 Hz, 3H, CH_3); **^{19}F NMR (377 MHz, MeOD-d^4)** δ -152.5 (q, *J* = 54.0 Hz); **^{11}B NMR (128 MHz, MeOD-d^4)** δ 3.1 (q, *J* = 54.0 Hz).

HRMS (ESI$^-$) $C_2H_4{}^{79}BrBF_3^-$ requires (M-K)$^-$ 174.9547, found 174.9546.

Potassium 1-trifluoro(*N*-ethylhydrazino)borate (3)

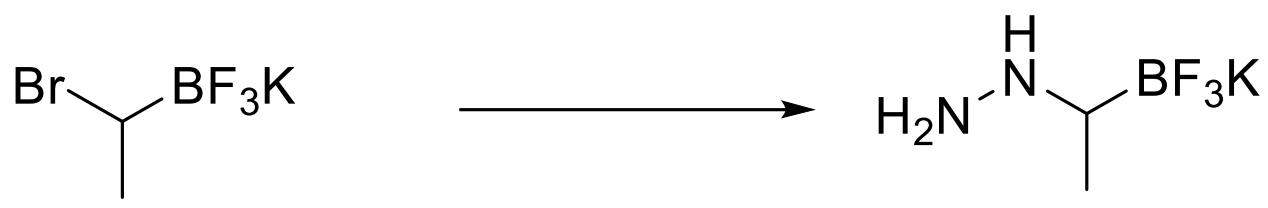

Hydrazine in THF (1 M, 50 mL, 50 mmol, 5 equiv.; **CARE:** toxic, can form explosive vapor-air mixtures), triethylamine (2.8 mL, 20 mmol, 2 equiv.) and potassium 1-trifluoro(bromoethyl)borate **1b** (2.15 g, 10 mmol, 1 equiv.) were left to stir overnight. After filtration, the solid was collected and the crude product was washed several times with acetonitrile to remove hydrazine, affording the desired product as a sticky white solid (996 mg, 60% yield).

^{1}H NMR (400 MHz, MeOD-d^{4}) δ 2.28 (dq, *J* = 7.0, 4.0 Hz, 1H, CH), 1.55 (d, *J* = 7.5 Hz, 3H, CH_3); **^{13}C NMR (101 MHz, MeOD**-d^{4}**)** δ 10.1; **^{19}F NMR (377 MHz, MeOD-d^{4})** δ -150.7 (br); **^{11}B NMR (128 MHz, MeOD-d^{4})** δ 2.7 (q, *J* = 53.0 Hz).

HRMS (ESI$^-$) $C_2H_7N_2BF_3^-$ requires (M-K)$^-$ 127.0654, found 127.0657.

Synthesis of potassium 1-trifluoro(1-methyl)borate 1,2-bis(phenylmethyl) 1,2-hydrazinedicarboxylate (4)

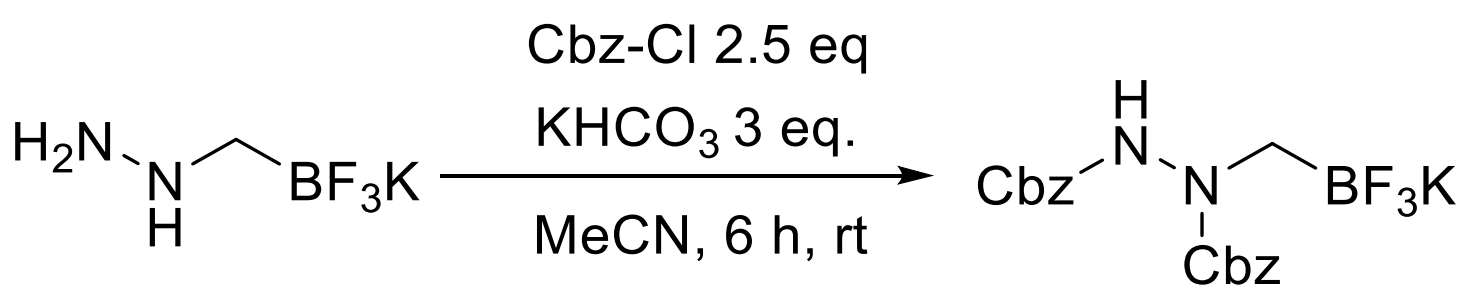

Potassium trifluoro(*N*-methylhydrazino)borate **(2)** (152 mg, 1 mmol), benzyl carbonochloridate (426 mg, 2.5 mmol, 2.5 equiv.), and $KHCO_3$ (300 mg, 3 mmol, 3 equiv.) were dissolved in MeCN (10 mL) and stirred at rt for 6 hours. The reaction mixture was concentrated *in vacuo*, dissolved in acetone and filtered to remove the base. The filtrate was concentrated *in vacuo* and the resulting oil was washed with ether (4 x 10 mL) to remove soluble impurities. The resulting residue was triturated with ether-hexane to afford the product **4** as white solid (254 mg, 59% yield).

^{1}H NMR (400 MHz, MeOD-d^{4}) δ 7.46 – 7.00 (m, 10H), 5.28 – 4.95 (m, 4H), 2.91 – 2.57 (br, 2H). **^{19}F NMR (376 MHz, MeOD-d^{4})** δ -144.5 (br). **^{11}B NMR (128 MHz, MeOD-d^{4})** δ 3.6 (br). **^{13}C NMR (101 MHz, MeOD-d^{4})** δ 159.1, 158.2, 138.1, 137.8, 129.4, 129.4, 129.0, 128.8, 128.7, 128.5, 68.5, 67.9. **HRMS** (ESI$^-$) $C_{17}H_{17}BF_3N_2O_4$ requires (M-K)$^-$ 381.1239, found 381.1239.

Synthesis of 1-(4-methoxyphenyl methyl)-1,2-bis(phenylmethyl) 1,2-hydrazinedicarboxylate (5)

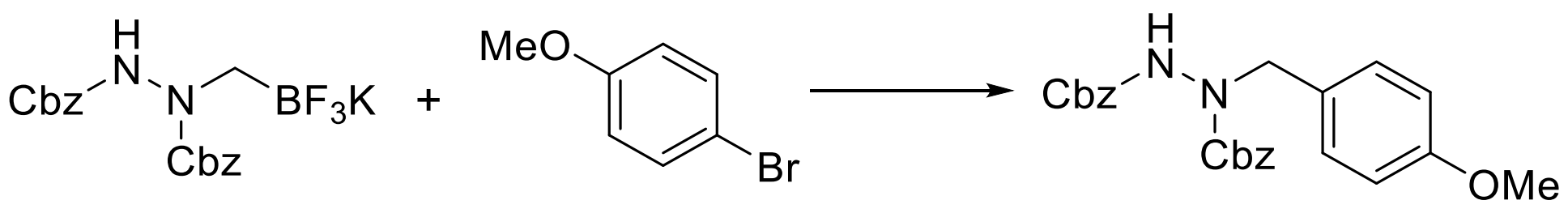

A 5 mL round bottom flask equipped with a stirrer bar was charged with **4** (103 mg, 0.25 mmol, 1.39 equiv.), Cs_2CO_3 (176 mg, 0.54 mmol, 3 equiv), 1-bromo-4-methoxybenzene (34 mg, 0.18 mmol, 1 equiv), $[PdCl(allyl)]_2$ (3.2 mg, 0.009 mmol, 10 mol%), and ^{S}SPhos (9.5 mg, 0.018 mmol, 10 mol%). CPME (1.8 mL) and H_2O (0.4 mL) were added by syringe, and the reaction mixture was stirred and heated at 100 °C for 24 h under N_2. The reaction mixture was then cooled to rt and diluted with H_2O (3 mL), and the product extracted with EtOAc (3 ×10 mL). The combined organic layers were dried over $MgSO_4$, filtered through Celite, and concentrated in vacuo. The product **5** isolated via column chromatography (silica, hexanes: EtOAc =3:1) as pale yellow oil (42 mg, 55% yield).

^{1}H NMR (400 MHz, MeOD) δ 7.45 – 7.07 (m, 13H), 6.94 – 6.70 (m, 2H), 5.31 – 4.98 (m, 4H), 4.60 (s, 2H), 3.76 (s, 3H). **^{13}C NMR (101 MHz, MeOD-d^4)** δ 160.8, 158.1, 137.7 (x2C), 131.0, 129.7, 129.5 (x2C), 129.3, 129.1, 128.8, 128.2, 128.0, 114.8, 69.0, 68.1, 65.2, 55.6. **HRMS** (ESI$^+$) $C_{24}H_{24}N_2O_5Na^+$ requires $(M+Na)^+$ 443.1584, found 443.1583.

Synthesis of 1-(2,4-dinitrophenyl)-1-((trifluoroboraneyl)methyl)hydrazine, potassium salt (6)

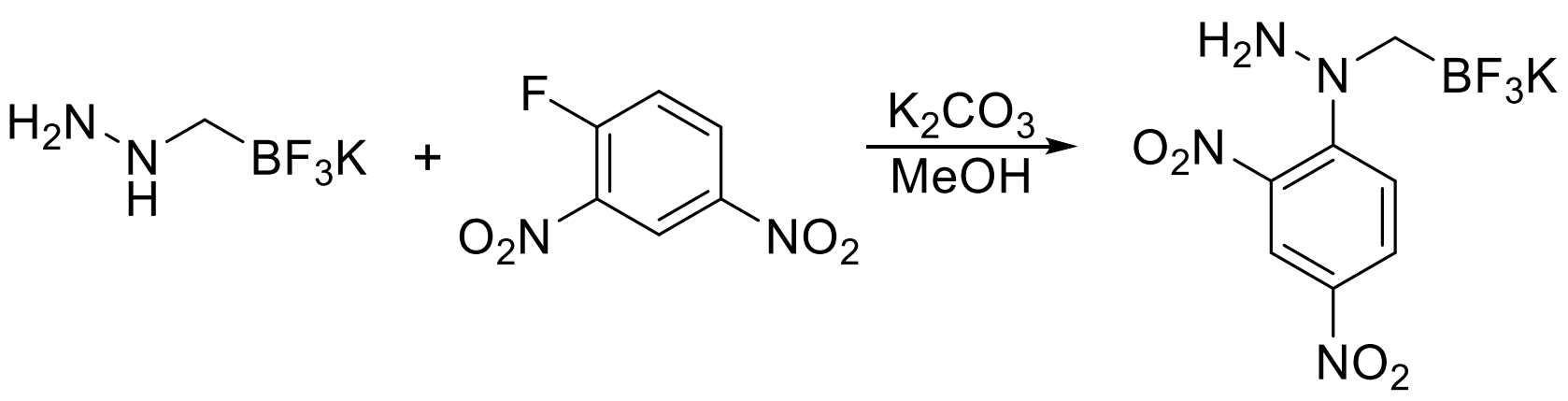

A glass vial was charged with trifluoro(N-methylhydrazino)borate **2** (22 mg, 0.145 mmol, 2.2 equiv.), 2,4-dinitrofluorobenzene (8.2 μL, 0.065 mmol, 1 equiv) and K_2CO_3 (9.2 mg, 0.065 mmol, 1 equiv.) in MeOH (0.3 mL) as stirred at room temperature for 2 hours. After concentrating on a rotary evaporator under reduced pressure, the yield of the resulting orange solid was calculated using 1,3,5-trimethoxybenzene as an internal standard (61%).

^{1}H NMR (400 MHz, DMSO-d^6) δ 8.22 (d, J = 2.5 Hz, 1H), 7.97 (dd, J = 9.5, 2.5 Hz, 1H), 6.95 (d, J = 9.5 Hz, 1H), 4.70 (brs, 2H), 2.70 (q, J = 5.0 Hz, 2H). **^{13}C NMR (101 MHz, DMSO-**

d[6]) δ 148.0, 134.2, 131.7, 126.3, 122.8, 114.7. **^{19}F NMR (377 MHz, DMSO-d[6])** δ -137.8 (br); **^{11}B NMR (128 MHz, DMSO-d[6])** δ 2.89 (q, *J* = 44.5 Hz).

HRMS (ESI$^-$) $C_7H_7BF_3N_4O_4^-$ requires $(M-K)^-$ 279.0512, found 279.0512.

Synthesis of Potassium Trifluoro(*N*-methylpyrazolyl)borate Salts

General procedure A

Potassium trifluoro(*N*-methylhydrazino)borate (**2**) (106 mg, 0.7 mmol, 1 equiv.) and dicarbonyl substrate **(**0.7 mmol, 1 equiv.) were dissolved in MeOH (10 mL). The reaction mixture was stirred at rt over the stated time. The reaction mixture was concentrated *in vacuo* and the product was precipitated from DCM and collected by filtration.

General procedure B

Potassium 1-trifluoro(*N*-ethylhydrazino)borate **(2)** (33 mg, 0.2 mmol, 1 equiv.) and dicarbonyl substrate (0.8 mmol, 4 equiv.) were dissolved in MeOH (10 mL). $KHCO_3$ (25 mg, 0.2 mmol, 1 equiv.) was added and the reaction mixture was stirred at rt over the stated time. The reaction mixture was concentrated *in vacuo* and filtered in acetone to remove the base. The product was precipitated from DCM or acetone with hexanes and collected by filtration.

General procedure C

Potassium trifluoro(*N*-methylhydrazino)borate (**2**) (61 mg, 0.4 mmol, 1 equiv.) and ynone substrate (1.6 mmol, 4 equiv.) were dissolved in MeOH (10 mL). $KHCO_3$ (40 mg, 0.4 mmol, 1 equiv.) was added and the reaction mixture was stirred at rt over the stated time. The reaction mixture was concentrated *in vacuo*, suspended in acetone or acetonitrile and filtered to remove the base. The filtrate was concentrated *in vacuo* and resulting solid was re-dissolved in the stated solvent, filtered and then concentrated *in vacuo* to afford the product.

Synthesis of potassium 3,5-dimethyl-(trifluoro(1-methylpyrazolyl)borate (7)

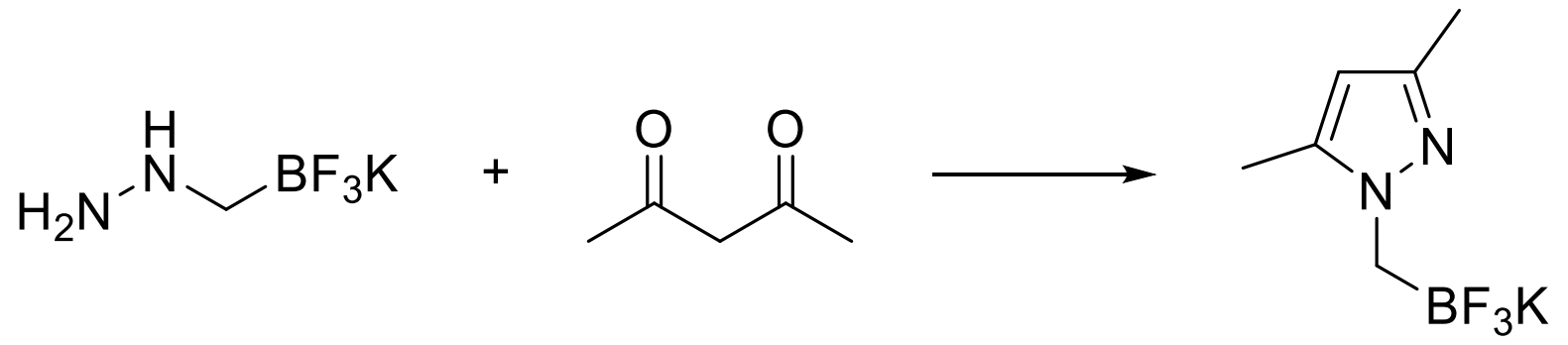

Following a scaled-up General procedure A using potassium trifluoro(*N*-methylhydrazino)borate (**2**) (1.00 g, 6.6 mmol) and acetyl acetone (658 mg, 6.6 mmol), the

reaction mixture was stirred for 2 days and the product **7** isolated as an amorphous white solid (1.23 g, 87% yield).

^{1}H NMR (400 MHz, CDCl$_3$) δ 6.07 (s, 1H, CH), 3.36 (q, *J* = 5.0 Hz, 2H, CH$_2$), 2.40 (s, 3H, CH$_3$), 2.36 (s, 3H, CH$_3$); **^{13}C NMR (101 MHz, CDCl$_3$)** δ 145.2, 142.6, 106.8, 11.4, 11.1; **^{19}F NMR (377 MHz, CDCl$_3$)** δ -142.7 (q, *J* = 52.5 Hz); **^{11}B NMR (128 MHz, CDCl$_3$)** δ 2.7 (q, *J* = 52.5 Hz).

HRMS (ESI$^-$) $C_6H_9BF_3N_2^-$ requires (M-K)$^-$ 177.0811, found 177.0825.

Synthesis of potassium 3,5-diethyl-(trifluoro(1-methylpyrazolyl)borate (8)

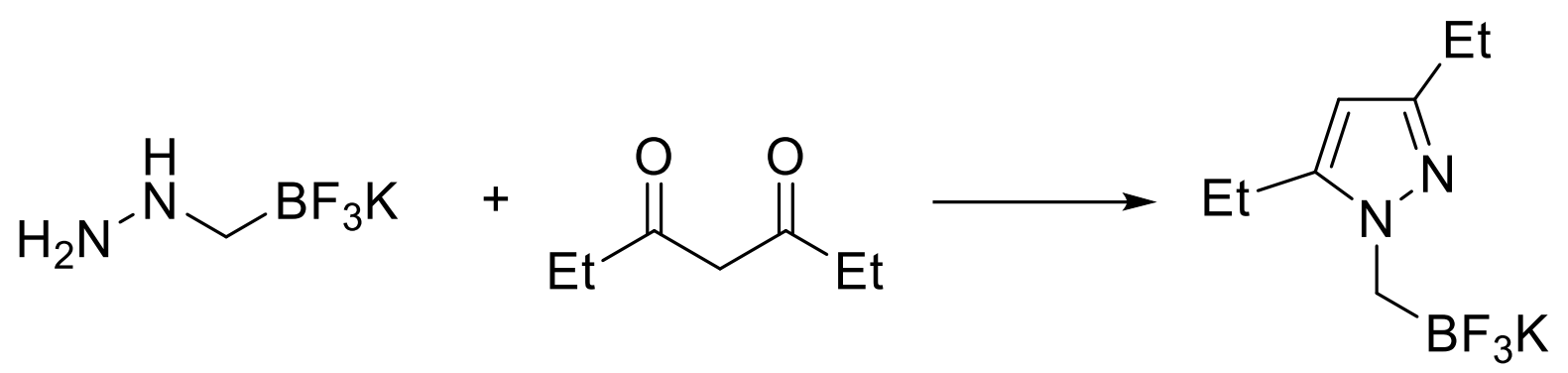

Following General procedure A using heptane-3,5-dione (90 mg, 0.7 mmol), the reaction mixture was stirred for 36 hours and the product **8** isolated as an amorphous pale yellow solid (140 mg, 82% yield).

^{1}H NMR (400 MHz, CDCl$_3$) δ 5.99 (s, 1H, CH), 3.32 (q, *J* = 5.0 Hz, 2H, CH$_2$), 2.66 (q, *J* = 7.5 Hz, 2H, CH$_2$), 2.64 (q, *J* = 7.5 Hz, 2H, CH$_2$), 1.26 (t, *J* = 7.5 Hz, 3H, CH$_3$), 1.22 (t, *J* = 7.5 Hz, 3H, CH$_3$); **^{13}C NMR (101 MHz, CDCl$_3$)** δ 149.1, 149.0, 102.5, 19.4, 18.8, 12.9, 11.9; **^{19}F NMR (377 MHz, CDCl$_3$)** δ -143.1 (br); **^{11}B NMR (128 MHz, CDCl$_3$)** δ 3.1 (br).

HRMS (ESI$^-$) $C_8H_{13}BF_3N_2^-$ requires (M-K)$^-$ 205.1124, found 205.1132.

Synthesis of potassium 3,5-diisopropyl-(trifluoro(1-methylpyrazolyl)borate (9)

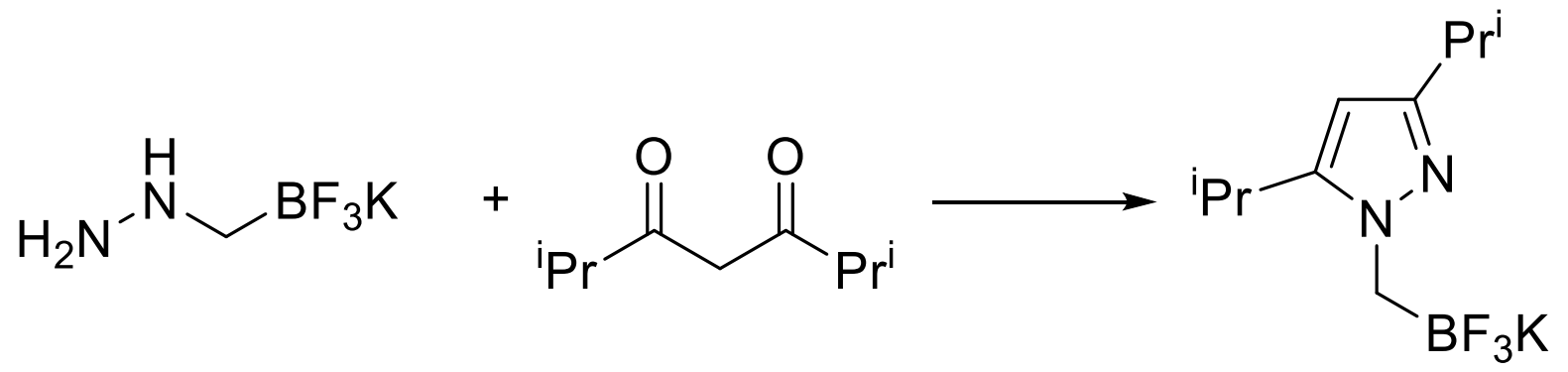

Following General procedure A using 2,6-dimethylheptane-3,5-dione (109 mg, 0.7 mmol), the reaction mixture was stirred for 2 days and the product **9** isolated as an amorphous pale yellow oil solid (125 mg, 65% yield).

^{1}H NMR (400 MHz, CDCl$_3$) δ 5.93 (s, 1H, CH), 3.36 (q, *J* = 5.0 Hz, 2H, CH$_2$), 3.07 (sept, *J* = 7.0 Hz, 1H, CH), 2.97 (sept, *J* = 7.0 Hz, 1H, CH), 1.22 (app t, *J* = 7.0 Hz, 12H, CH$_3$); **^{13}C NMR (101 MHz, CDCl$_3$)** δ 153.7, 153.2, 99.0, 26.3, 25.1, 22.2, 21.9; **^{19}F NMR (377 MHz, CDCl$_3$)** δ -143.2 (br); **^{11}B NMR (128 MHz, CDCl$_3$)** δ 3.2 (br).

HRMS (ESI$^-$) $C_{10}H_{17}BF_3N_2^-$ requires $(M-K)^-$ 233.1437, found 233.1452.

Synthesis of potassium 3,5-trifluoromethyl-(trifluoro(1-methylpyrazolyl)borate (10)

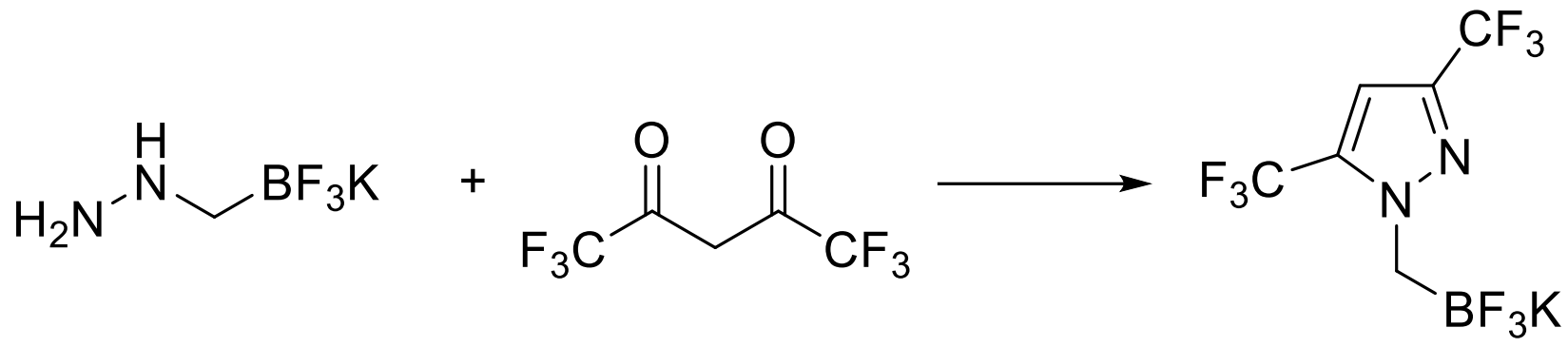

Following a scaled-up General procedure A using potassium trifluoro(*N*-methylhydrazino)borate (**2**) (303 mg, 2 mmol, 1 equiv.) and 1,1,1,5,5,5-hexafluoropentane-2,4-dione (416 mg, 2 mmol), the reaction mixture was stirred for 60 hours and the product **10** isolated as an amorphous pale yellow solid (430 mg, 66% yield).

^{1}H NMR (400 MHz, DMSO-d^6) δ 7.18 (s, 1H, CH), 3.33 (q, J = 4.5 Hz, 2H, CH_2); **^{13}C NMR (101 MHz, DMSO-d^6)** δ 138.4 (q, J = 38.0 Hz), 132.0 (q, J = 39.0 Hz), 121.6 (q, J = 268.0 Hz), 120.0 (q, J = 269.0 Hz), 105.7 (q, J = 2.0 Hz); **^{19}F NMR (377 MHz, DMSO-d^6)** δ -58.5 (s, CF_3), -60.2 (s, CF_3), -141.6 (q, J = 50.0 Hz, BF_3K); **^{11}B NMR (128 MHz, DMSO-d^6)** δ 2.1 (q, J = 50.0 Hz).

HRMS (ESI$^-$) $C_6H_3BF_9N_2^-$ requires $(M-K)^-$ 285.0246, found 285.0251.

Synthesis of potassium 3,4,5-trimethyl-(trifluoro(1-methylpyrazolyl)borate (11)

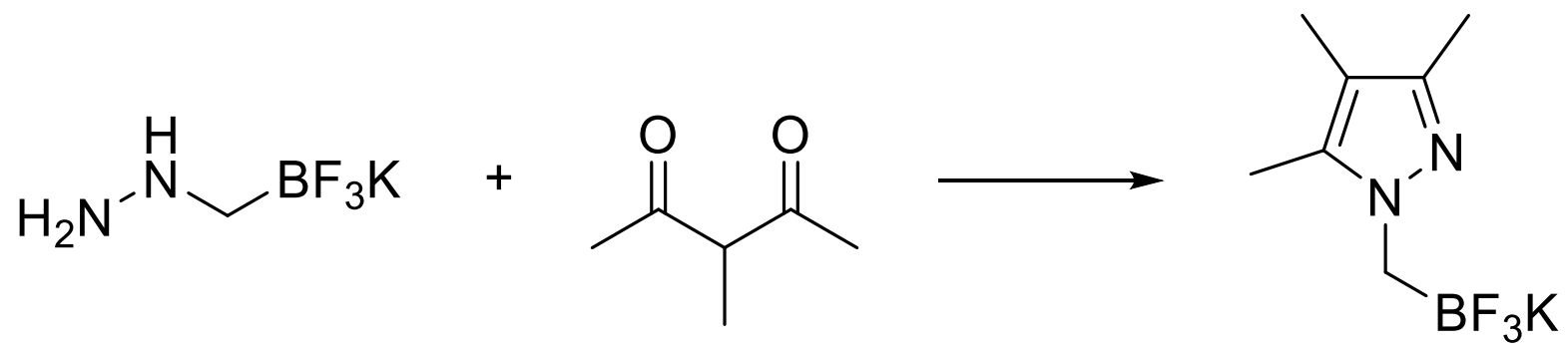

Following General procedure A using 3-methylpentane-2,4-dione (80 mg, 0.7 mmol), the reaction mixture was stirred for 36 hours and the product **11** isolated as an amorphous pale yellow solid (115 mg, 72% yield).

^{1}H NMR (400 MHz, $CDCl_3$) δ 3.34 (q, J = 5.0 Hz, 2H, CH_2), 2.27 (s, 3H, CH_3), 2.25 (s, 3H, CH_3), 1.94 (s, 3H, CH_3); **^{13}C NMR (101 MHz, $CDCl_3$)** δ 142.5, 140.1, 113.3, 9.7, 9.6, 7.6; **^{19}F NMR (377 MHz, $CDCl_3$)** δ -143.1 (br); **^{11}B NMR (128 MHz, $CDCl_3$)** δ 2.9 (br).

HRMS (ESI$^-$) $C_7H_{11}BF_3N_2^-$ requires $(M-K)^-$ 191.0967, found 191.0971.

Synthesis of potassium methyl 3,5-dimethyl-(trifluoro(1-methylpyrazolyl-4-propanoate)borate (12)

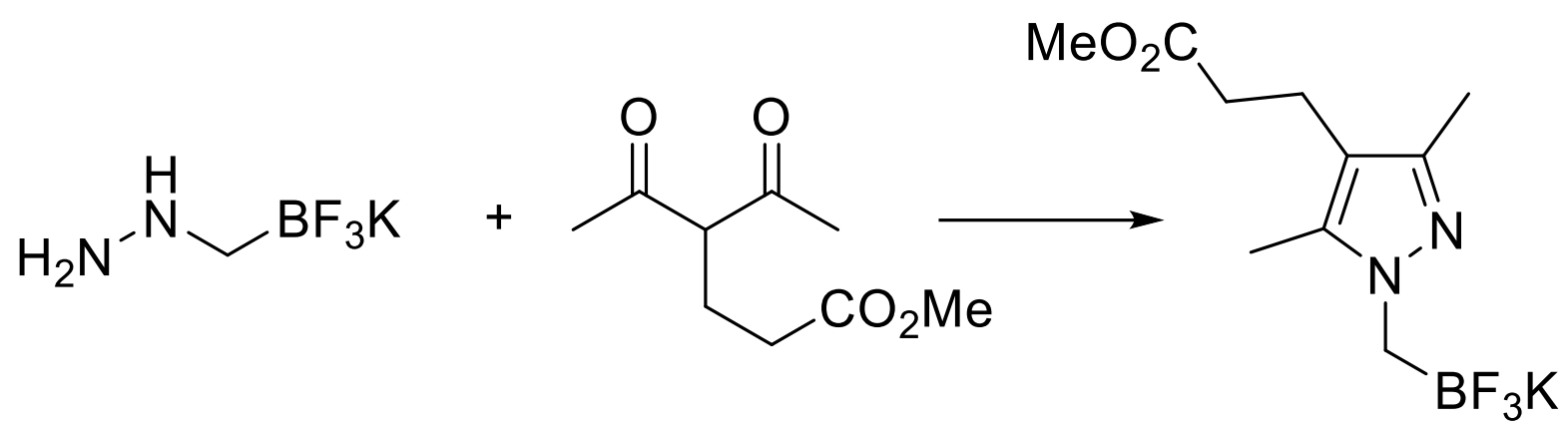

Following a modification of General procedure A using potassium trifluoro(*N*-methylhydrazino)borate (**2**) (106 mg, 0.7 mmol, 2 equiv.) and methyl 4-acetyl-5-oxohexanoate (65 mg, 0.35 mmol) in MeOH (10 mL), the reaction mixture was stirred for 2 days and the product **12** isolated as an amorphous yellow solid (86 mg, 82% yield).

^{1}H NMR (400 MHz, $CDCl_3$) δ 3.65 (s, 3H), 3.29 (q, *J* = 5.0 Hz, 2H, CH_2), 2.68 (t, *J* = 7.5 Hz, 2H, CH_2), 2.44 (t, *J* = 7.5 Hz, 2H, CH_2), 2.24 (s, 3H), 2.23 (s, 3H); **^{13}C NMR (101 MHz, $CDCl_3$)** δ 172.8, 141.2, 141.1, 115.2, 51.8, 34.1, 18.4, 9.9, 9.5; **^{19}F NMR (377 MHz, $CDCl_3$)** δ -143.3 (br); **^{11}B NMR (128 MHz, $CDCl_3$)** δ 3.4 (br).

HRMS (ESI^-) $C_{10}H_{15}BF_3N_2O_2^-$ requires $(M\text{-}K)^-$ 263.1179, found 263.1192.

Synthesis of potassium methyl 3,5-dimethyl-(1-trifluoro(1-ethylpyrazolyl-4-propanoate)borate (13)

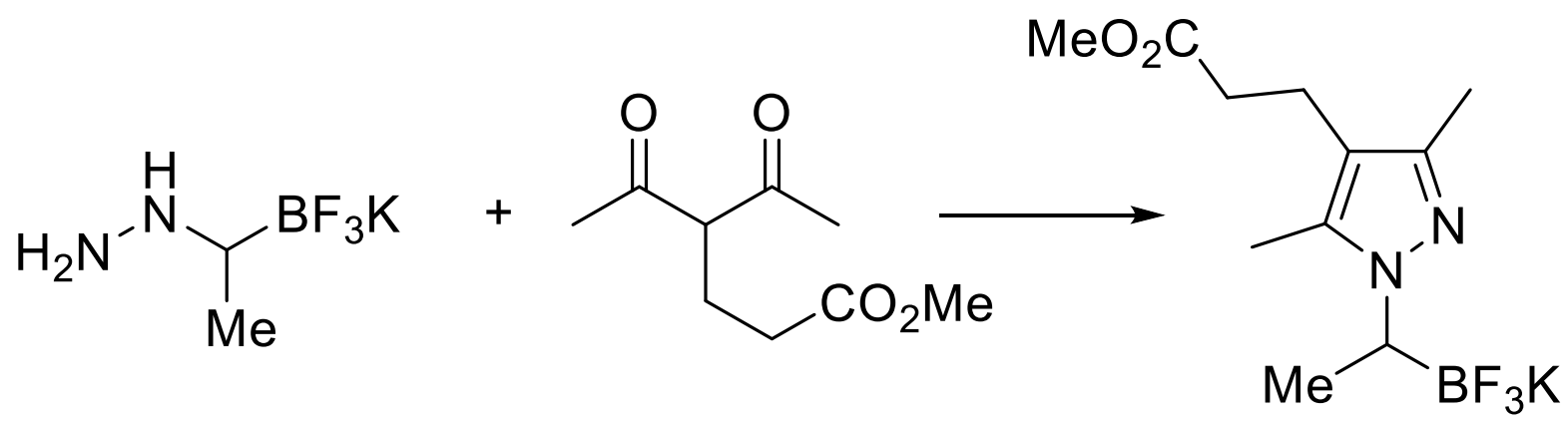

Following General procedure B using methyl 4-acetyl-5-oxohexanoate (149 mg, 0.8 mmol), the reaction mixture was stirred for 2 days and the product **13** isolated as an amorphous yellow solid (45 mg, 71% yield).

^{1}H NMR (400 MHz, $CDCl_3$) δ 3.65 (s, 3H, CH_3), 3.59 (m, 1H, CH), 2.69 (t, *J* = 7.5 Hz, 2H, CH_2), 2.45 (t, *J* = 7.5 Hz, 2H, CH_2), 2.26 (s, 3H, CH_3), 2.25 (s, 3H, CH_3), 1.29 (d, *J* = 7.5 Hz, 3H, CH_3); **^{13}C NMR (101 MHz, $CDCl_3$)** δ 172.8, 134.5, 116.0, 113.0, 52.0, 34.1, 18.4, 16.7, 10.1, 9.6; **^{19}F NMR (377 MHz, $CDCl_3$)** δ -146.9 (br); **^{11}B NMR (128 MHz, $CDCl_3$)** δ 3.1 (br)

HRMS (ESI^-) $C_{11}H_{17}BF_3N_2O_2^-$ requires $(M\text{-}K)^-$ 277.1335, found 277.1344.

Synthesis of potassium 3-ethyl 5-methyl-(trifluoro(1-methylpyrazolyl)borate (14a) and potassium 3-methyl 5-ethyl-(trifluoro(1-methylpyrazolyl)borate (14b)

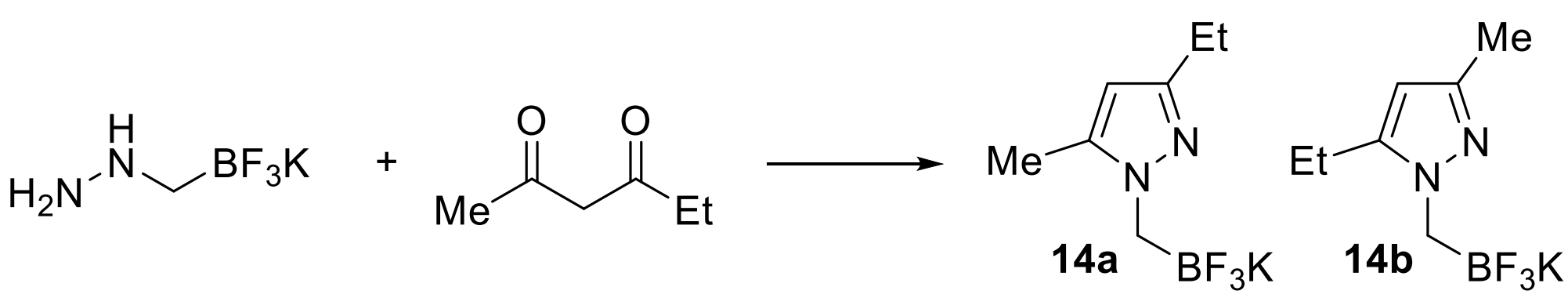

Following General procedure A using hexane-2,4-dione (114 mg, 1 mmol), the reaction mixture was stirred for 3 days and the products **14a,b** isolated as an amorphous pale yellow solid (194 mg, 84% yield, r.r. = 1:1).

^{1}H NMR (400 MHz, CDCl$_3$) δ 5.90 (s, 0.5H, CH^{A}), 5.89 (s, 0.5H, CH^{B}), 3.35 – 3.26 (m, 2H, CH$_2$^{A} and CH$_2$^{B}), 2.65-2.54 (m, 2H, CH$_2$^{A} and CH$_2$^{B}), 2.25 (s, 1.5H, CH$_3$^{A}), 2.22 (s, 1.5H, CH$_3$^{B}), 1.23 – 1.13 (m, 6H, CH$_3$^{A} and CH$_3$^{B}); **^{13}C NMR (101 MHz, CDCl$_3$)** δ 148.6 (A), 148.2 (B), 142.9 (B), 142.7 (A), 103.9 (A), 103.6 (B), 41.7 (A and B, br), 19.1 (A), 18.5 (B), 12.8 (A), 11.8 (B), 11.2 (B), 11.0 (A); **^{19}F NMR (377 MHz, CDCl$_3$)** δ -143.8 (m, A and B); **^{11}B NMR (128 MHz, CDCl$_3$)** δ 2.9 (m, A and B).

HRMS (ESI$^-$) $C_7H_{11}BF_3N_2^-$ requires (M-K)$^-$ 191.0967, found 191.0971 and 191.0970.

Synthesis of potassium 3-ethyl 5-methyl-(1-trifluoro(1-ethylpyrazolyl)borate (15 major) and potassium 3-methyl 5-ethyl-(1-trifluoro(1-ethylpyrazolyl)borate (15 minor)

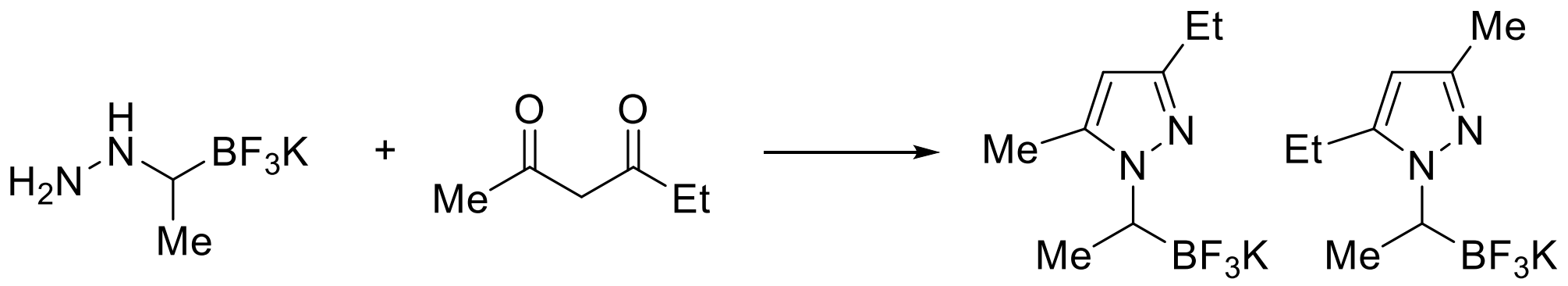

Following General procedure B using hexane-2,4-dione (91 mg, 0.8 mmol), the reaction mixture was stirred for 2 days and the products **15 major/minor** isolated as an amorphous yellow solid (32 mg, 65% yield, r.r. = 7:3).

^{1}H NMR (400 MHz, CDCl$_3$) 5.91 (s, 0.7H, CH, major), 5.87 (s, 0.3H, CH, minor), 3.54 (br, 1H, CH, major and minor), 2.66-2.54 (m, 2H, CH$_2$, major and minor), 2.26 (s, 2.1H, CH$_3$, major), 2.21 (s, 0.9H, CH$_3$, minor), 1.37-1.15 (m, 6H, CH$_3$, major and minor); **^{13}C NMR (101 MHz, CDCl$_3$)** δ 149.9 (x2 C), 142.9, 142.8, 104.1, 104.0, 19.7, 18.8, 16.9, 16.6, 13.0, 12.5, 12.1, 11.4; **^{19}F NMR (377 MHz, CDCl$_3$)** δ -147.0 (br, mixture); **^{11}B NMR (128 MHz, CDCl$_3$)** δ 4.0 (br, mixture).

HRMS (ESI$^-$) $C_8H_{13}BF_3N_2^-$ requires (M-K)$^-$ 205.1124, found 205.1133.

Synthesis of potassium 3-cyclopropyl 5-methyl(trifluoro(1-methylpyrazolyl)borate (16 major) and potassium 3-methyl 5-cyclopropyl(trifluoro(1-methylpyrazolyl)borate (16 minor)

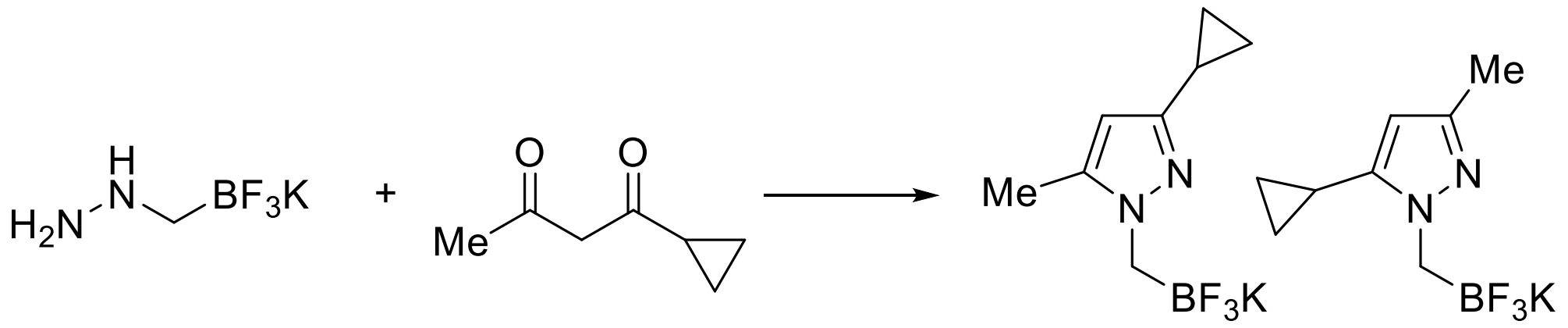

Following General procedure A using 1-cyclopropylbutane-1,3-dione (88 mg, 0.7 mmol), the reaction mixture was stirred for 5 days and the products **16 major/minor** isolated as an amorphous pale yellow solid (130 mg, 77% yield, r.r. = 3:2).

^{1}H NMR (400 MHz, CDCl$_3$) δ 5.68 (s, 1H, CH, major and minor), 3.48 (q, J = 5.0 Hz, 0.8H, CH$_2$ minor), 3.33 (q, J = 5.0 Hz, 1.2H, CH$_2$, major), 2.26 (s, 3H, CH$_3$, major and minor), 1.98 – 1.82 (m, 1H, CH, major and minor), 1.12 – 1.03 (m, 0.8H, CH$_2$, minor), 1.03 – 0.94 (m, 1.2H, CH$_2$, major), 0.76 – 0.63 (m, 2H, CH$_2$, major and minor). **^{13}C NMR (101 MHz, CDCl$_3$)** major/minor δ 150.7, 149.8, 144.5, 142.9, 102.7, 101.8, 11.4, 11.2, 9.3, 8.0, 6.9, 6.3.**^{19}F NMR (377 MHz, CDCl$_3$)** δ -143.3 (br, minor), -143.5 (br, major); **^{11}B NMR (128 MHz, CDCl$_3$)** δ 3.1 (br, major and minor).

HRMS (ESI$^-$) $C_8H_{11}BF_3N_2^-$ requires (M-K)$^-$ 203.0967, found 203.0975.

Synthesis of potassium 3-*tert*-butyl-5-methyl(trifluoro(1-methylpyrazolyl)borate (17)

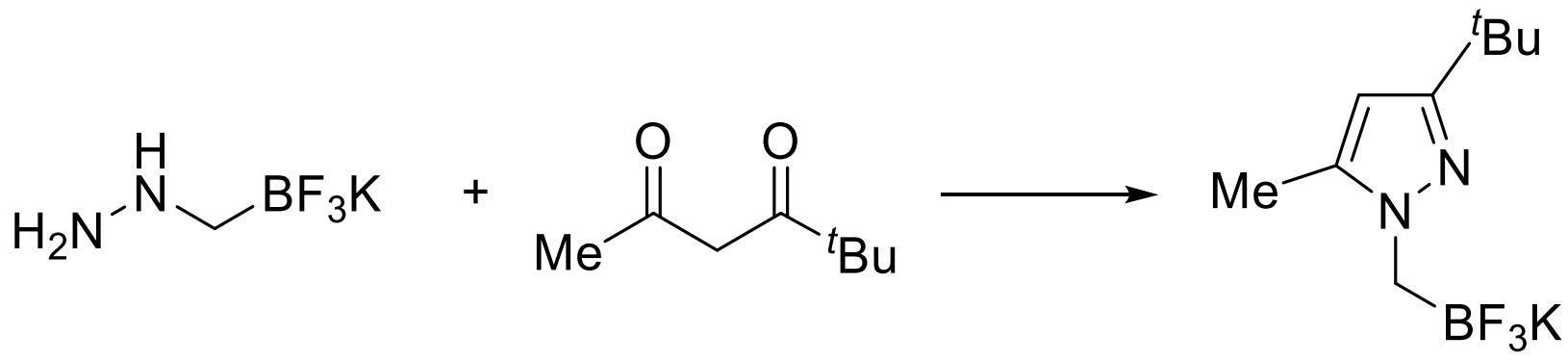

Following General procedure A using potassium trifluoro(*N*-methylhydrazino)borate (**2**) (30.4 mg, 0.2 mmol) and 5,5-dimethylhexane-2,4-dione (85.3 mg, 0.6 mmol, 3 equiv.), the reaction mixture was stirred for 5 days. The mixture was concentrated, dissolved in CH$_2$Cl$_2$, filtered and the filtrate was concentrated *in vacuo*. The product **17** isolated as a pale yellow oil (24 mg, 47% yield, r.r. > 98:2).

^{1}H NMR (400 MHz, CDCl$_3$) δ 5.91 (s, 1H, CH), 3.34 (q, J = 5.5 Hz, 2H, CH$_2$), 2.25 (s, 3H, CH$_3$), 1.25 (s, 9H, CH$_3$); **^{13}C NMR (101 MHz, CDCl$_3$)** δ 156.4, 142.5, 102.5, 31.6, 30.0, 11.4; **^{19}F NMR (377 MHz, CDCl$_3$)** δ -143.3 (br); **^{11}B NMR (128 MHz, CDCl$_3$)** δ 3.4 (br).

HRMS (ESI$^-$) $C_9H_{15}BF_3N_2^-$ requires (M-K)$^-$ 219.1280, found 219.1290.

Synthesis of potassium 3-carboxymethyl 5-methyl(trifluoro(1-methylpyrazolyl)borate (18)

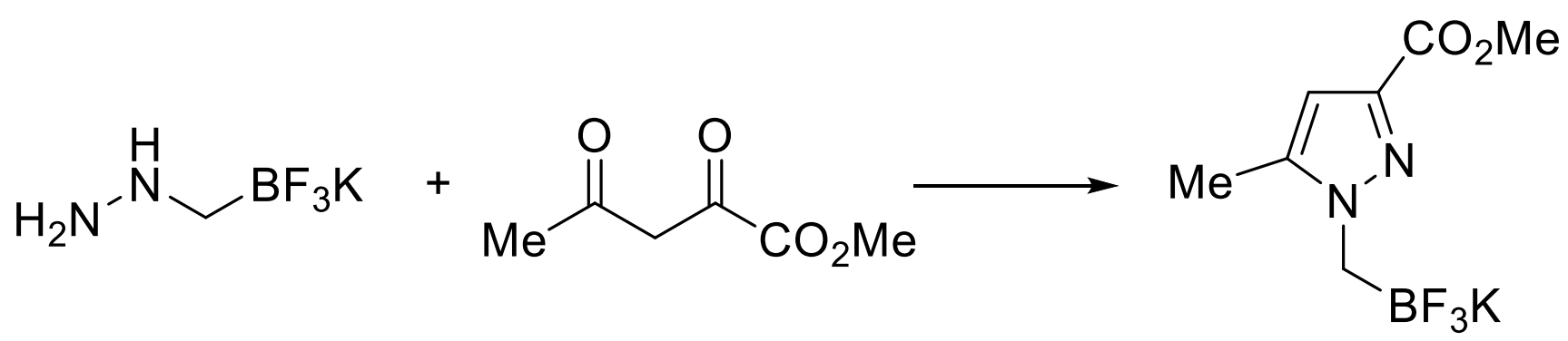

Following a modification of General procedure A using potassium trifluoro(*N*-methylhydrazino)borate (**2**) (106 mg, 0.7 mmol, 2 equiv.) and methyl 2,4-dioxopentanoate (50

mg, 0.35 mmol) in MeOH (10 mL), the reaction mixture was stirred for 3 days and the product **18** isolated as an amorphous pale yellow solid (77 mg, 85% yield, r.r. = >90:10). Data described for the major isomer only.

^{1}H NMR (400 MHz, DMSO-d^6) δ 6.37 (s, 1H, CH), 3.72 (s, 3H, OCH_3), 3.10 (q, J = 5.0 Hz, 2H, CH_2), 2.19 (s, 3H, CH_3); **^{13}C NMR (101 MHz, DMSO-d^6)** δ 162.9, 138.7, 138.2, 106.9, 50.9, 11.0; **^{19}F NMR (377 MHz, DMSO-d^6)** δ -139.9 (br); **^{11}B NMR (128 MHz, DMSO-d^6)** δ 2.6 (br).

HRMS (ESI$^-$) $C_7H_9BF_3N_2O_2^-$ requires $(M\text{-}K)^-$ 221.0709, found 221.0718.

Synthesis of trifluoro((3-methyl-4,5,6,7-tetrahydro-2*H*-indazol-1-ium-2-yl)methyl)borate (19 major) and trifluoro((3-methyl-4,5,6,7-tetrahydro-1*H*-indazol-2-ium-1-yl)methyl)borate (19 minor)

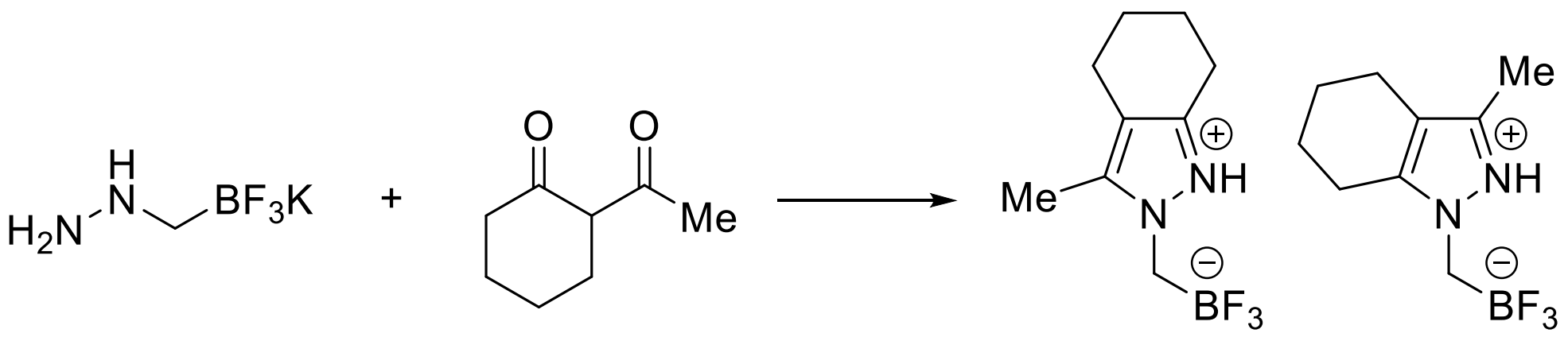

Following a modification of General procedure A using potassium trifluoro(*N*-methylhydrazino)borate (**2**) (61 mg, 0.4 mmol, 2 equiv.) and 2-acetylcyclohexan-1-one (28 mg, 0.2 mmol) in MeOH (10 mL). 5 Drops of concentrated HCl were added and the reaction mixture was stirred for 3 days. The products **19 major/minor** were isolated as an orange solid (28 mg, 64% yield, r.r. = 1:3). Recrystallization from $CHCl_3$ to generate a clean sample of the major isomer for X-ray crystallography. **M.P.** 118 °C (dec.).

^{1}H NMR (400 MHz, $CDCl_3$) δ 12.18 (s, 1H, NH, major and minor), 3.37-3.29 (m, 2H, CH_2, major and minor), 2.74 (t, J = 6.0 Hz, 1.5H, CH_2, major), 2.65 (t, J = 6.0 Hz, 0.5H, CH_2, minor), 2.44-2.38 (m, 2H, CH_2, major and minor), 2.32 (s, 0.75H, CH_3, minor), 2.26 (s, 2.25H, CH_3, major), 1.92 – 1.72 (m, 4H, major and minor); **^{13}C NMR (101 MHz, $CDCl_3$)** major and minor δ 144.8, 142.4, 141.6, 139.1, 115.9, 115.7, 22.2, 22.1, 21.7, 21.6, 21.0, 20.8, 19.4, 19.3, 9.6, 9.4; **^{19}F NMR (377 MHz, $CDCl_3$)** δ -142.6 (br, major and minor); **^{11}B NMR (128 MHz, $CDCl_3$)** δ 2.8 (q, J = 47.0 Hz, major and minor).

HRMS (ESI$^-$) $C_9H_{13}BF_3N_2^-$ requires $(M\text{-}H)^-$ 217.1124, found 217.1133.

Synthesis of trifluoro((3-methyl-5,6-dihydrocyclopenta[c]pyrazol-1-ium-2(*4H*)-yl)methyl)borate (20 major) and trifluoro((3-methyl-5,6-dihydrocyclopenta[c]pyrazol-2-ium-1(*4H*)-yl)methyl)borate (20 minor)

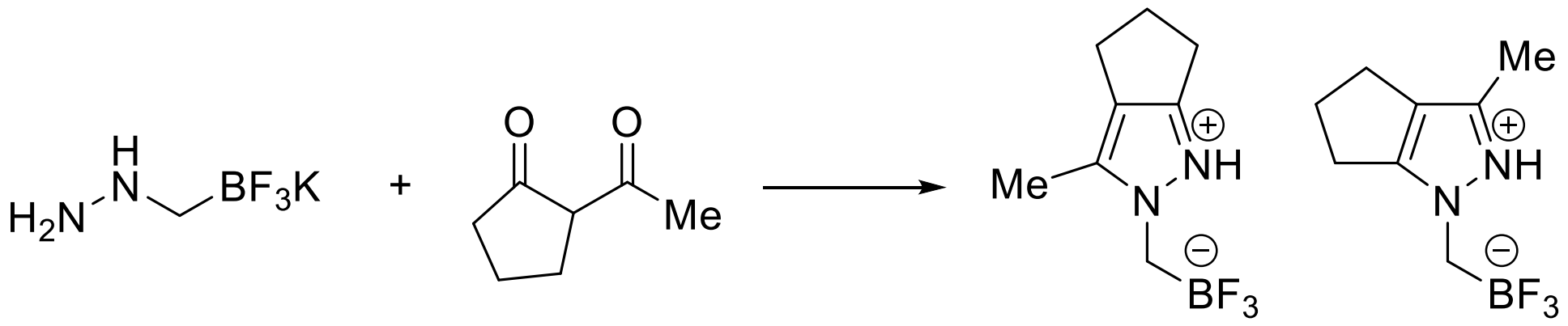

Following a modification of General procedure A using potassium trifluoro(*N*-methylhydrazino)borate (**2**) (61 mg, 0.4 mmol, 2 equiv.) and 2-acetylcyclopentan-1-one (25 mg, 0.2 mmol) in MeOH (10 mL). 5 Drops of concentrated HCl were added and the reaction mixture was stirred for 3 days. The products **20 major/minor** were isolated as an orange crystalline solid as a major/minor mixture (27 mg, 66% yield, r.r. = 1:5).

^{1}H NMR (400 MHz, CDCl$_3$) δ 12.02 (br, 1H, NH, major and minor), 3.38-3.34 (m, 2H, CH_2, major and minor), 2.93 (t, *J* = 7.5 Hz, 0.4H, CH_2, minor), 2.84 (t, *J* = 7.5 Hz, 1.6H, CH_2, major), 2.65 (t, *J* = 7.0 Hz, 2H, CH_2, major and minor), 2.60 – 2.49 (m, 2H, CH_2, major and minor), 2.34 (s, 2.55H, CH_3, major), 2.30 (s, 0.45H, CH_3, minor); **^{13}C NMR (101 MHz, CDCl$_3$)** major/minor δ 155.8, 151.9, 138.7, 136.5, 126.3, 125.4, 29.5, 29.3, 24.9, 24.0, 22.9, 22.6, 10.5, 10.2; **^{19}F NMR (377 MHz, CDCl$_3$)** δ -142.4 (br, minor), -143.2 (br, major); **^{11}B NMR (128 MHz, CDCl$_3$)** δ 2.7 (q, *J* = 53.0 Hz, major and minor).

HRMS (ESI$^-$) $C_8H_{12}BF_3N_2^-$ requires (M-H)$^-$ 203.0967, found 203.0971.

Synthesis of 3-methyl(trifluoro(1-methylpyrazolyl)borate (21 major) and 5-methyl(trifluoro(1-methylpyrazolyl)borate (21 minor)

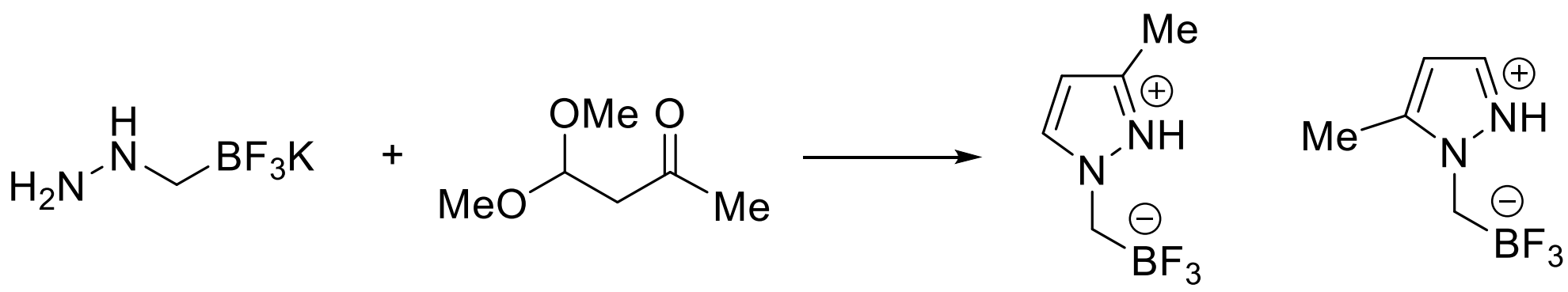

Following a modification of General procedure A using potassium trifluoro(*N*-methylhydrazino)borate (**2**) (61 mg, 0.4 mmol, 1 equiv.) and 4,4-dimethoxybutan-2-one (53 mg, 0.4 mmol) in MeOH (10 mL). 5 Drops of concentrated HCl were added and the reaction mixture was stirred for 2 days. The products **21 major/minor** were isolated as a pale orange solid (40 mg, 60% yield, r.r. = 1:2).

^{1}H NMR (400 MHz, CDCl$_3$) δ 12.82 (s, 0.35H, NH, minor), 12.73 (s, 0.65H, NH, major), 7.7 (d, *J* = 2.0 Hz, 0.65H, CH, major), 7.59 (s, 0.35H, CH, minor), 6.34 (d, *J* = 2.0 Hz, 0.65H, CH, major), 6.26 (s, 0.35H, CH, minor), 3.55 (q, *J* = 4.5 Hz, 0.75H, CH_2, minor), 3.46 (q, *J* = 4.0 Hz, 1.25H, CH_2, major), 2.45 (s, 1H, minor), 2.42 (s, 2H, major); **^{13}C NMR (101 MHz, CDCl$_3$)**

δ 144.8, 144.1, 135.6, 131.1, 107.3, 106.6, 11.5, 11.2; **^{19}F NMR (377 MHz, $CDCl_3$)** δ -142.6 (br, major), -143.5 (br, minor); **^{11}B NMR (128 MHz, $CDCl_3$)** δ 2.7 (br, major and minor). **HRMS** (ESI$^-$) $C_5H_7BF_3N_2^-$ requires $(M-H)^-$ 163.0654, found 163.0659.

Synthesis of potassium 3-cyclohexyl(trifluoro(1-methylpyrazolyl)borate (22 major) and potassium 5-cyclohexyl(trifluoro(1-methylpyrazolyl)borate (22 minor)

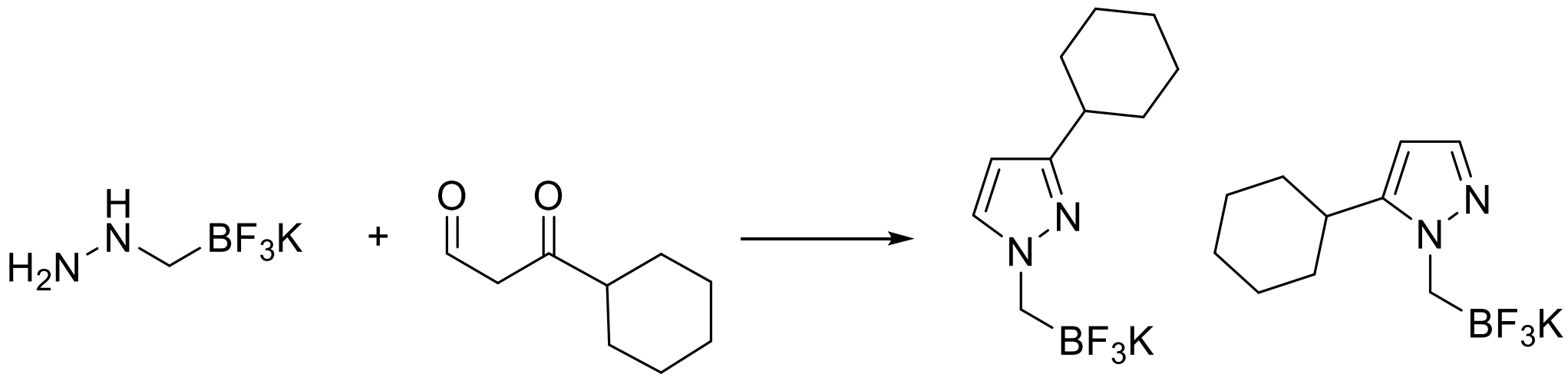

Following a modification of General procedure A using potassium trifluoro(*N*-methylhydrazino)borate (**2**) (106 mg, 0.7 mmol, 1 equiv.) and 3-cyclohexyl-3-oxopropanal (116 mg, 0.7 mmol) in MeOH (10 mL). 5 Drops of concentrated HCl were added and the reaction mixture was stirred for 2 days. The products **22 major/minor** were isolated as a pale yellow amorphous solid (167 mg, 88% yield, r.r. = 4:1).

^{1}H NMR (400 MHz, $CDCl_3$) δ 7.68 (d, J = 2.5 Hz, 1H, CH, major and minor), 6.24 (d, J = 3.0 Hz, 0.2H, CH, minor), 6.15 (d, J = 2.5 Hz, 0.8H, CH, major), 3.43 (m, 2H, CH_2, major and minor), 2.80 – 2.64 (m, 1H, CH, major and minor), 1.99 – 1.63 (m, 5H, major and minor), 1.43 – 1.14 (m, 5H, major and minor); **^{13}C NMR (101 MHz, $CDCl_3$)** 153.5, 151.8, 134.2, 132.7, 103.0, 102.9, 35.6, 34.5, 32.5, 31.6, 25.9, 25.8, 25.6; **^{19}F NMR (377 MHz, $CDCl_3$)** δ -142.8 (br, minor), -143.7 (br, major); **^{11}B NMR (128 MHz, $CDCl_3$)** δ 2.7 (br, major and minor) **HRMS** (ESI$^-$) $C_{10}H_{15}BF_3N_2^-$ requires $(M-K)^-$ 231.1280, found 231.1287.

Synthesis of potassium 4-carboxyethyl(trifluoro(1-methylpyrazolyl)borate (23)

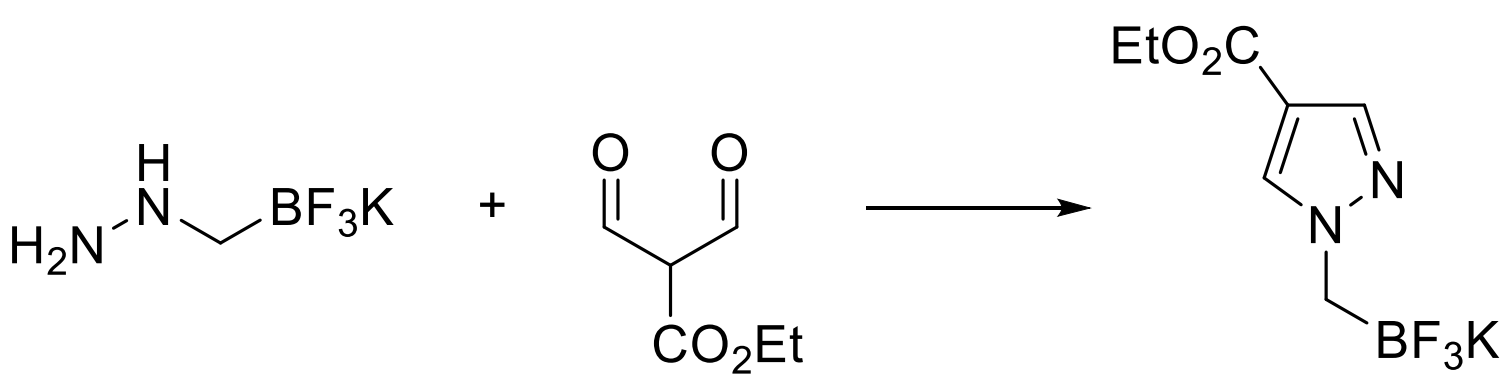

Following a modified General procedure A using potassium trifluoro(*N*-methylhydrazino)borate (**2**) (76 mg, 0.5 mmol) and ethyl 2-formyl-3-oxopropanoate (73 mg, 0.5 mmol), the reaction mixture was stirred for 3 days and the product **23** isolated as an amorphous yellow solid (108 mg, 91% yield).

^{1}H NMR (400 MHz, DMSO-d^6) δ 7.98 (s, 1H, CH), 7.70 (s, 1H, CH), 4.17 (q, J = 7.0 Hz, 2H, CH_2), 3.09 (q, J = 4.5 Hz, 2H, CH_2), 1.24 (t, J = 7.0 Hz, 3H, CH_3); **^{13}C NMR (101 MHz,**

DMSO-d[6]) δ 162.7, 138.4, 132.8, 112.6, 59.4, 14.4; **^{19}F NMR (377 MHz, DMSO-d[6])** δ -141.0 (br); **^{11}B NMR (128 MHz, DMSO-d[6])** δ 2.8 (q, *J* = 54.5 Hz).

HRMS (ESI$^-$) $C_7H_9BF_3N_2O_2^-$ requires $(M-K)^-$ 221.0709, found 221.0715.

Synthesis of potassium 4-carboxyethyl-(1-trifluoro(1-ethylpyrazolyl)borate (24)

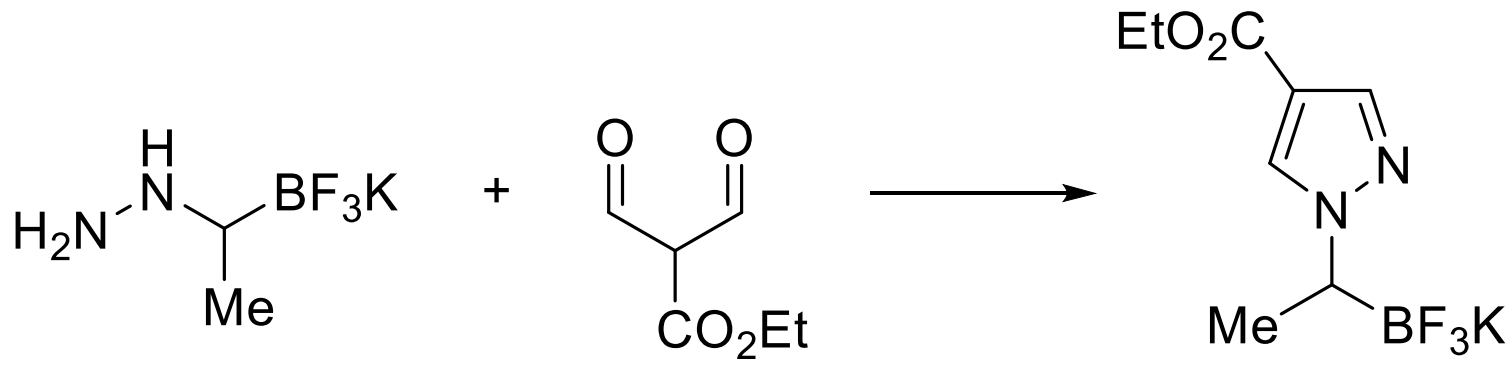

Following General procedure F using ethyl 2-formyl-3-oxopropanoate (115 mg, 0.8 mmol), the reaction mixture was stirred for 2 days and the product **24** isolated as an amorphous yellow solid (52 mg, 95% yield).

^{1}H NMR (400 MHz, DMSO-d[6]) δ 7.99 (s, 1H, CH), 7.68 (s, 1H, CH), 4.17 (q, *J* = 7.0 Hz, 1H, CH_2), 3.25 (br, 1H, CH), 1.25 (t, *J* = 7.0 Hz, 3H, CH_3), 1.17 (d, *J* = 7.5 Hz, 3H, CH_3); **^{13}C NMR (101 MHz, DMSO-d[6])** δ 162.8, 138.4, 131.9, 112.2, 59.2, 18.5, 14.3; **^{19}F NMR (377 MHz, DMSO-d[6])** δ -145.9 (br); **^{11}B NMR (128 MHz, DMSO-d[6])** δ 3.1 (m).

HRMS (ESI$^-$) $C_8H_{11}BF_3N_2O_2^-$ requires $(M-K)^-$ 235.0866, found 235.0873.

Synthesis of potassium 3-methyl 5-ethyl-(trifluoro(1-methylpyrazolyl)borate (25)

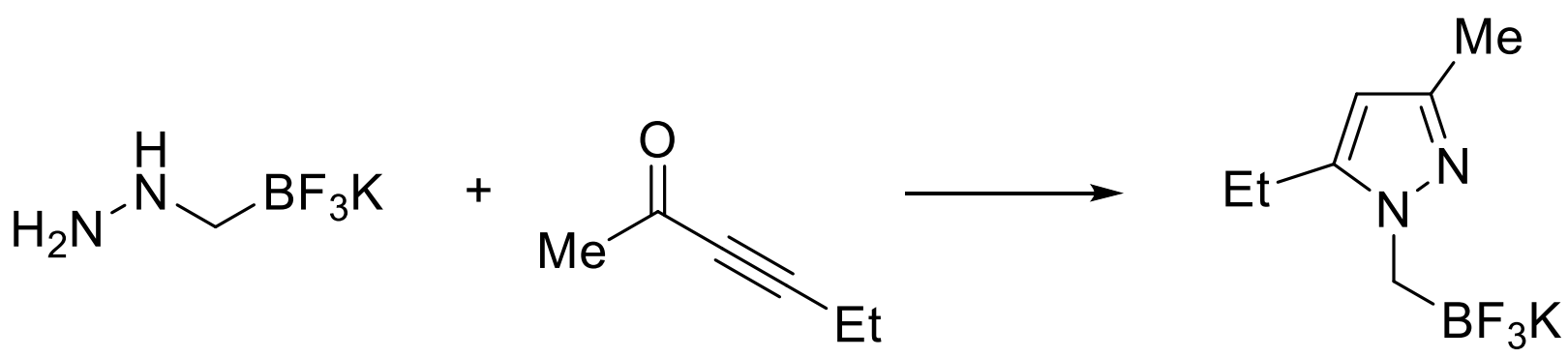

Following general procedure C using 3-hexyn-2-one (154 mg, 1.6 mmol), the reaction mixture was stirred for 2 days. The mixture was concentrated, dissolved in acetone, filtered and the filtrate was concentrated *in vacuo*. This process was repeated and the resulting solid was washed with ether to afford the product **25** as a pale yellow solid (35 mg, 38% yield, r.r. > 98:2).

^{1}H NMR (400 MHz, MeOD-d[4]) δ 5.74 (s, 1H), 3.17 (q, *J* = 5.5 Hz, 2H), 2.61 (q, *J* = 7.5 Hz, 2H), 2.15 (s, 3H), 1.20 (t, *J* = 7.5 Hz, 3H); **^{13}C NMR (101 MHz, MeOD-d[4])** δ 146.7, 145.7, 102.6, 19.7, 13.1, 13.0; **^{19}F NMR (377 MHz, MeOD-d[4])** δ -145.6 (br); **^{11}B NMR (128 MHz, MeOD-d[4])** δ 3.2 (q, *J* = 55.0 Hz).

HRMS (ESI$^-$) $C_7H_{11}BF_3N_2^-$ requires $(M-K)^-$ 191.0967, found 191.0977.

Synthesis of potassium 3-*n*-butyl 5-*n*-propyl-(trifluoro(1-methylpyrazolyl)borate (26) and potassium 3-*n*-propyl 5-*n*-butyl-(trifluoro(1-methylpyrazolyl)borate (27)

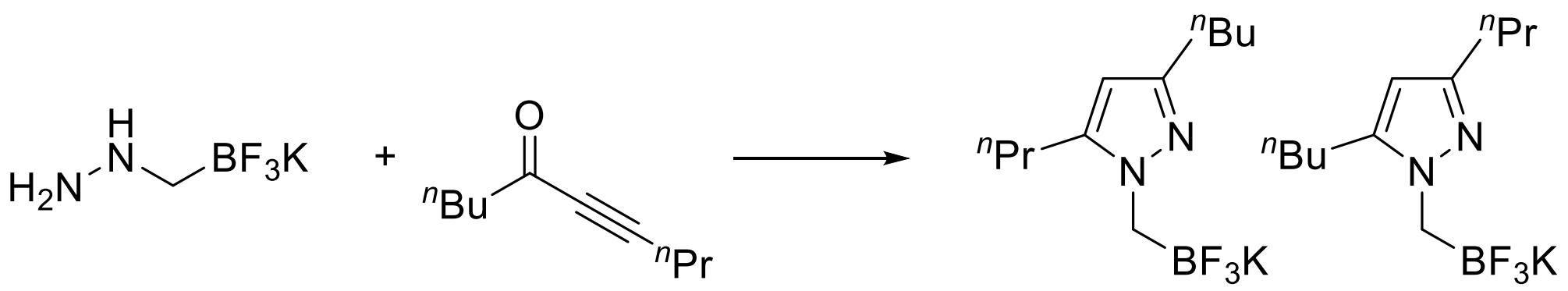

Following general procedure C using dec-6-yn-5-one (61 mg, 0.4 mmol), the reaction mixture was stirred for 2 days. The mixture was concentrated and extracted with CH_2Cl_2 (3 x 10 mL) and the extract was concentrated *in vacuo*. The solid was washed with minimal cold hexane and the solid was dried *in vacuo* to afford the product **26** and **27** isolated as a white solid (45.5 mg, 40% yield, r.r. = 85:15).

^{1}H NMR (400 MHz, CDCl$_3$) major δ 5.67 (s, 1H), 3.08 (s, 2H), 2.44 (t, *J* = 8.0 Hz, 2H), 2.28 (t, *J* = 8.5 Hz, 2H), 1.59 – 1.49 (m, 2H), 1.47 – 1.38 (m, 2H), 1.30 – 1.21 (m, 2H), 0.94 (t, *J* = 7.5 Hz, 3H), 0.85 (t, *J* = 7.0 Hz, 3H); **^{13}C NMR (101 MHz, CDCl$_3$)** major δ 150.5, 143.9, 100.8, 32.0, 27.7, 27.6, 22.6, 21.5, 13.9 ×2C; **^{19}F NMR (377 MHz, CDCl$_3$)** δ -142.9 (br). **^{11}B NMR (128 MHz, CDCl$_3$)** δ 0.3 (br).

HRMS (ESI$^-$) $C_{11}H_{19}BF_3N_2^-$ requires (M-K)$^-$ 247.1593, found 247.1602.

Synthesis of potassium 3-*n*-propyl 5-*n*-butyl-(trifluoro(1-methylpyrazolyl)borate (27) and potassium 3-*n*-butyl 5-*n*-propyl-(trifluoro(1-methylpyrazolyl)borate (26)

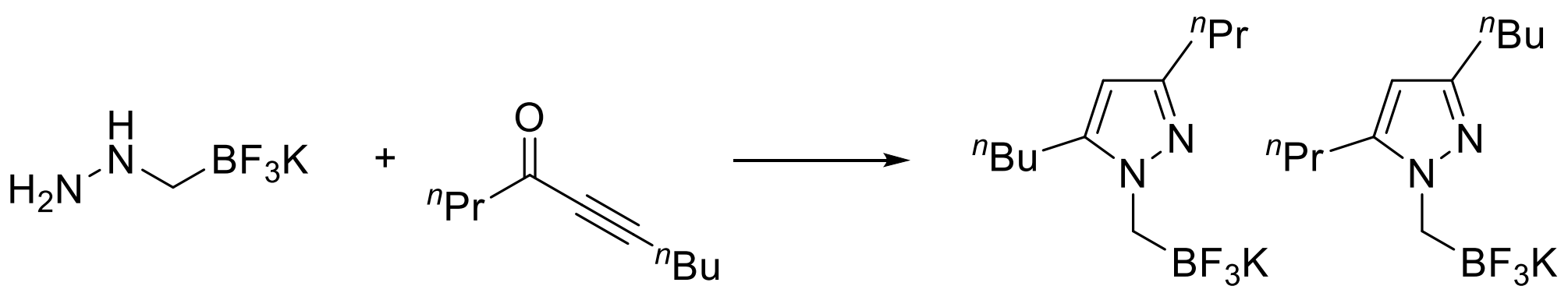

Following general procedure C using dec-5-yn-4-one (61 mg, 0.4 mmol), the reaction mixture was stirred for 2 days. The mixture was concentrated and extracted with CH_2Cl_2 (3 x 10 mL) and the extract was concentrated *in vacuo*. The solid was washed with minimal cold hexane and the solid was dried *in vacuo* to afford the product **27** and **26** as a white solid (45 mg, 40% yield, r.r. = 85:15).

^{1}H NMR (400 MHz, CDCl$_3$) major δ 5.65 (s, 1H), 3.07 (q, *J* = 4.5 Hz, 2H), 2.45 (t, *J* = 7.5 Hz, 2H), 2.25 (t, *J* = 7.5 Hz, 2H), 1.57 – 1.39 (m, 4H), 1.38 – 1.27 (m, 2H), 0.89 (t, *J* = 7.5 Hz, 3H), 0.82 (t, *J* = 7.5 Hz, 3H); **^{19}F NMR (377 MHz, CDCl$_3$)** δ -142.7 (br); **^{11}B NMR (128 MHz, CDCl$_3$)** δ 0.3 (br); **^{13}C NMR (101 MHz, CDCl$_3$)** major δ 150.5, 144.3, 101.0, 30.5, 30.0, 25.5, 23.1, 22.6, 14.1, 14.0.

HRMS (ESI$^-$) $C_{11}H_{19}BF_3N_2^-$ requires (M-K)$^-$ 247.1593, found 247.1593.

Synthesis of potassium 3-phenyl 5-cyclopropyl-(trifluoro(1-methylpyrazolyl)borate (28)

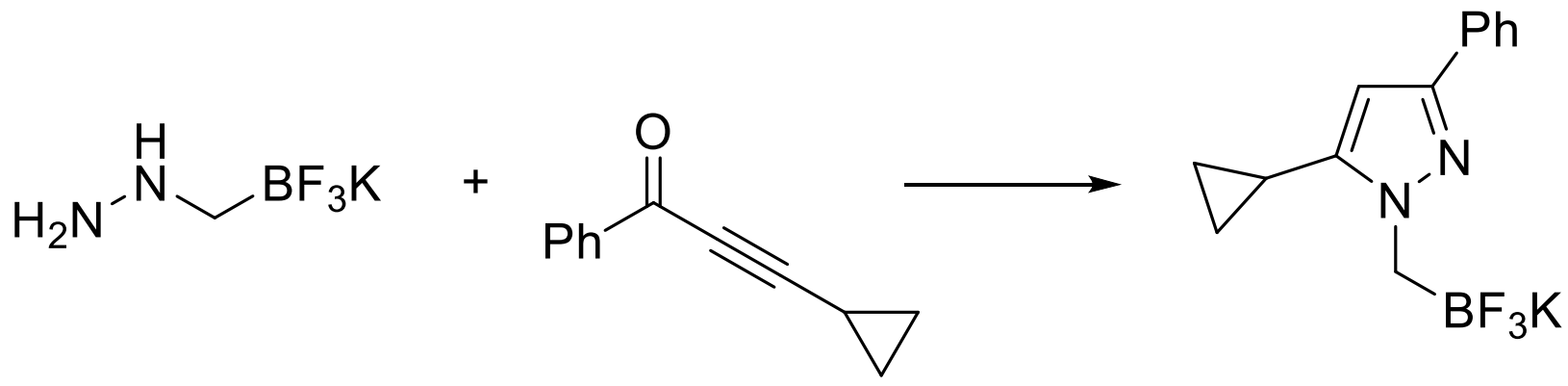

Following general procedure C using potassium trifluoro(*N*-methylhydrazino)borate (**2**) (152 mg, 1 mmol), 3-cyclopropyl-1-phenylprop-2-yn-1-one (681 mg, 4 mmol), and $KHCO_3$ (100 mg, 1 mmol) in MeOH (25 mL) the reaction mixture was stirred for 2 days. The mixture was concentrated, dissolved in acetone, filtered and the filtrate was concentrated *in vacuo*. The resulting solid was washed with hexane (3 x 10 mL) to afford the product **28** as a yellow solid (155.4 mg, 51% yield, r.r. > 98:2).

^{1}H NMR (400 MHz, MeOD-d^4) δ 7.72 – 7.65 (m, 2H), 7.34 – 7.28 (m, 2H), 7.23 – 7.16 (m, 1H), 6.08 (s, 1H), 3.47 (q, *J* = 5.5 Hz, 2H), 2.10 – 2.00 (m, 1H), 0.98 – 0.91 (m, 2H), 0.68 – 0.61 (m, 2H); **^{13}C NMR (101 MHz, MeOD-d^4)** δ 149.5, 147.8, 135.7, 129.4, 127.8, 126.4, 99.1, 7.5, 7.4; **^{19}F NMR (377 MHz, MeOD-d^4)** δ -145.3 (s); **^{11}B NMR (128 MHz, MeOD-d^4)** δ 3.4 (br).

HRMS (ESI$^-$) $C_{13}H_{13}BF_3N_2^-$ requires (M-K)$^-$ 265.1124, found 265.1127.

Synthesis of potassium 3-phenyl 5-*n*-butyl-(trifluoro(1-methylpyrazolyl)borate (29)

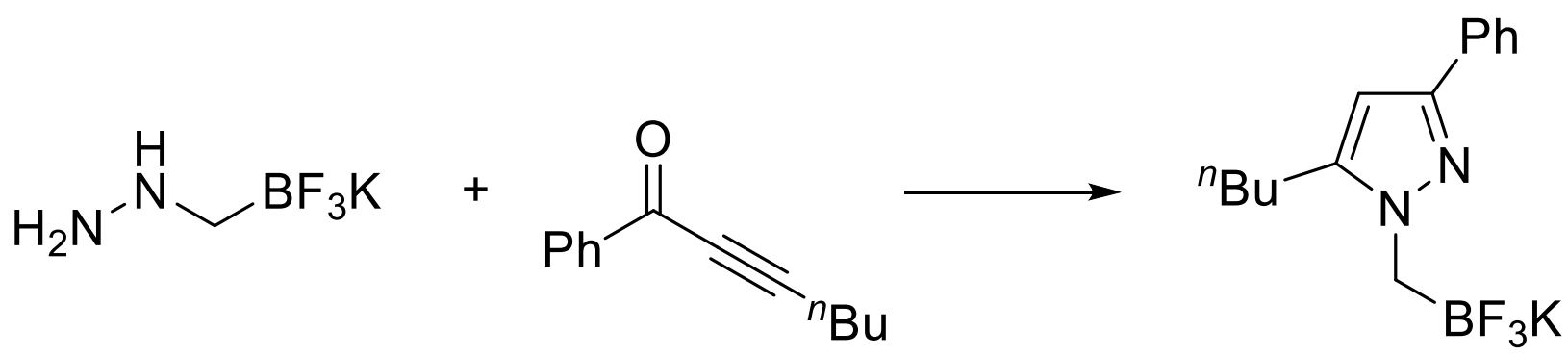

Following general procedure C using 1-phenylhept-2-yn-1-one (298 mg, 1.6 mmol), the reaction mixture was stirred for 2 days. The mixture was concentrated, dissolved in acetone, filtered and the filtrate was concentrated *in vacuo*. The resulting solid was washed with hexane (3 x 10 mL) to afford the product **29** as a yellow solid (57 mg, 44% yield, r.r. > 98:2).

^{1}H NMR (400 MHz, DMSO-d^6) δ 7.68 (d, *J* = 7.5 Hz, 2H), 7.31 (t, *J* = 7.5 Hz, 2H), 7.16 (t, *J* = 7.5 Hz, 1H), 6.27 (s, 1H), 3.08 (q, *J* = 5.0 Hz, 2H), 2.59 (t, *J* = 7.5 Hz, 2H), 1.63 – 1.51 (m, 2H), 1.43 – 1.31 (m, 2H), 0.92 (t, *J* = 7.5 Hz, 3H). **^{13}C NMR (101 MHz, DMSO-d^6)** δ 145.8, 143.0, 134.9, 128.3, 126.0, 124.4, 99.4, 30.1, 24.7, 22.1, 13.9; **^{19}F NMR (377 MHz, DMSO-d^6)** δ -139.4 (br); **^{11}B NMR (128 MHz, DMSO-d^6)** δ 2.9 (br).

HRMS (ESI$^-$) $C_{14}H_{17}BF_3N_2^-$ requires (M-K)$^-$ 281.1437, found 281.1445.

Synthesis of potassium 3-methyl-(trifluoro(1-methylpyrazolyl)borate (30)

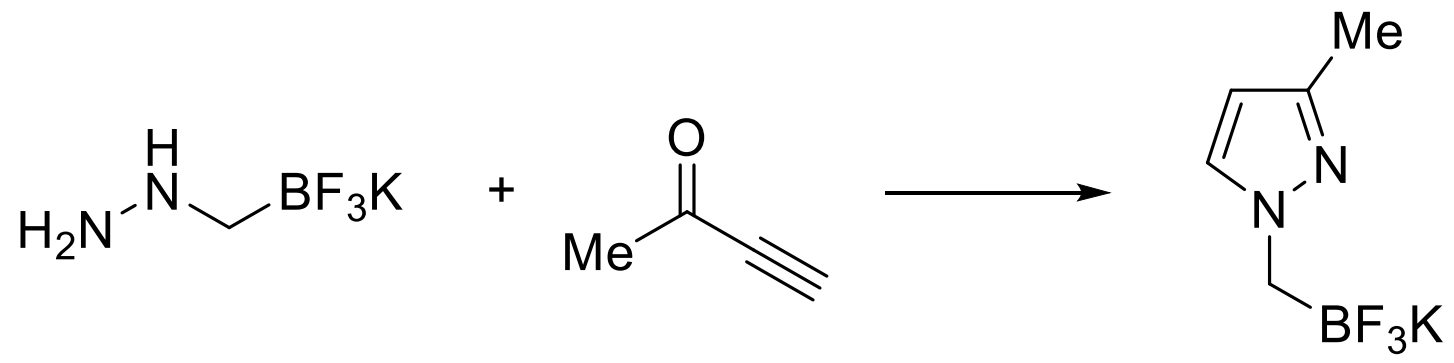

Following general procedure C using potassium trifluoro(*N*-methylhydrazino)borate (**2**) (152 mg, 1 mmol), 3-butyn-2-one (272 mg, 4 mmol), and $KHCO_3$ (100 mg, 1 mmol) in MeOH (25 mL) the reaction mixture was stirred for 2 days. The mixture was concentrated, dissolved in acetonitrile, filtered and the filtrate was concentrated *in vacuo*. The resulting solid was washed with CH_2Cl_2 (3 x 10 mL) to afford a solid that was dried *in vacuo* to afford the product **30** as a pale brown solid (60 mg, 30% yield, r.r. > 98:2).

^{1}H NMR (400 MHz, DMSO-d^6) δ 7.36 (s, 1H), 5.77 (s, 1H), 2.94 (q, J = 5.0 Hz, 2H), 2.07 (s, 3H) ; **^{13}C NMR (101 MHz, DMSO-d^6)** δ 144.0, 129.7, 102.6, 13.4; **^{19}F NMR (377 MHz, DMSO-d^6)** δ -140.2 (q, J = 50.0 Hz); **^{11}B NMR (128 MHz, DMSO-d^6)** δ 3.0 (q, J = 55.5 Hz).
HRMS (ESI$^-$) $C_5H_7BF_3N_2^-$ requires (M-K)$^-$ 163.0654, found 163.0657.

Functionalization Reactions

Synthesis of potassium 4-bromo 3,5-dimethyl-(trifluoro(1-methylpyrazolyl)borate (31)

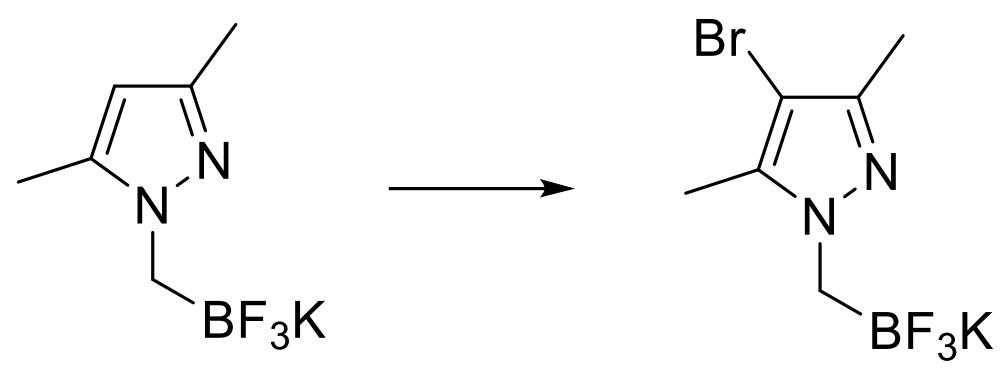

N-Bromosuccinimide (NBS) (71 mg, 0.4 mmol, 1 equiv.) was dissolved in MeCN (2 mL) and 3,5-dimethyl-1-((trifluoro-λ^4-boranyl)methyl)-1*H*-pyrazole, potassium salt (86.4 mg, 0.4 mmol, 1 equiv.) was added. The reaction mixture was stirred at room temperature for 1 hour. The reaction mixture was transferred into a larger vessel and concentrated *in vacuo* and resulting residue was dissolved in CH_2Cl_2 and the solid removed by filtration. The solid was washed with CH_2Cl_2 (3 × 10 mL), dried and collected to afford the product **31** as a white solid (91 mg, 77% yield).

^{1}H NMR (400 MHz, DMSO-d^6) δ 3.00 (q, J = 5.0 Hz, 2H), 2.09 (s, 3H), 2.00 (s, 3H); **^{13}C NMR (101 MHz, DMSO-d^6)** δ 140.8, 135.1, 90.4, 11.9, 10.2; **^{19}F NMR (377 MHz, DMSO-d^6)** δ -139.7 (br); **^{11}B NMR (128 MHz, DMSO-d^6)** δ 2.7 (br).
HRMS (ESI$^-$) $C_6H_8{}^{79}BrBF_3N_2^-$ requires (M-K)$^-$ 254.9921, found 254.9931.

Synthesis of 4-[1-[(3,5-dimethylpyrazol-1-yl)methyl]-3-phenyl-propyl]morpholine (32)

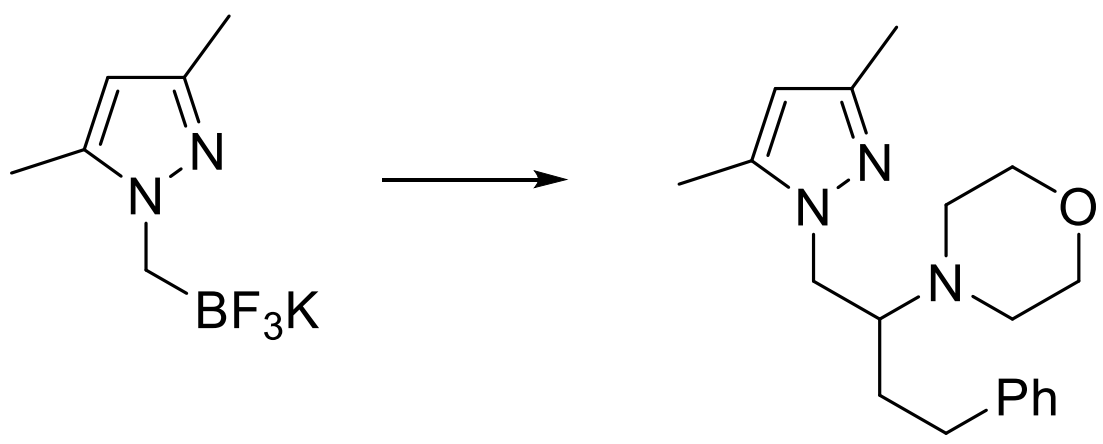

A glass vial was charged with potassium 3,5-dimethyl-(trifluoro(1-methylpyrazolyl)borate (68.2 mg, 0.3 mmol), *tert*-butyl catechol (50.0 mg, 0.3 mmol), 3-phenylpropanal (26.3 µL, 0.2 mmol), morpholine (26.0 µL, 0.3 mmol) and HFIP (1 mL). The headspace of the tube was purged with a gentle stream of argon for approximately 30 seconds. The vial was closed and irradiated under 450 nm for 17 h (the color of the reaction mixture turned from yellow to colorless). The reaction mixture was concentrated on a rotary evaporator under reduced pressure, and the crude was purified on a small pad of silica using heptane:EtOAc from 3:1 to 1:2 to afford the product **32** as a pale orange solid (29 mg, 47% yield).

^{1}H NMR (400 MHz, $CDCl_3$) δ 7.31 – 7.20 (m, 3H), 7.19 – 7.09 (m, 2H), 5.77 (s, 1H), 4.14 (dd, J = 14.0, 6.5 Hz, 1H), 3.86 (dd, J = 14.0, 7.5 Hz, 1H), 3.68 (qt, J = 11.0, 4.5 Hz, 4H), 3.10 – 3.01 (m, 1H), 2.75 – 2.50 (m, 6H), 2.27 – 2.13 (m, 6H), 1.90 – 1.77 (m, 1H), 1.67 – 1.53 (m, 1H).**^{13}C NMR (101 MHz, $CDCl_3$)** δ 147.6, 142.1, 139.2, 128.5, 128.5, 126.0, 105.3, 67.5, 63.4, 48.9, 48.2, 32.8, 30.4, 13.6, 11.4.

HRMS (ESI$^+$) $C_{19}H_{28}N_3O^+$ requires $(M+H)^+$ 314.2232, found 314.2235.

Synthesis of ethyl 4-[(E)-2-(3-bromo-5,5,8,8-tetramethyl-5,6,7,8-tetrahydronaphthalen-2-yl)ethenyl]benzoate (34)

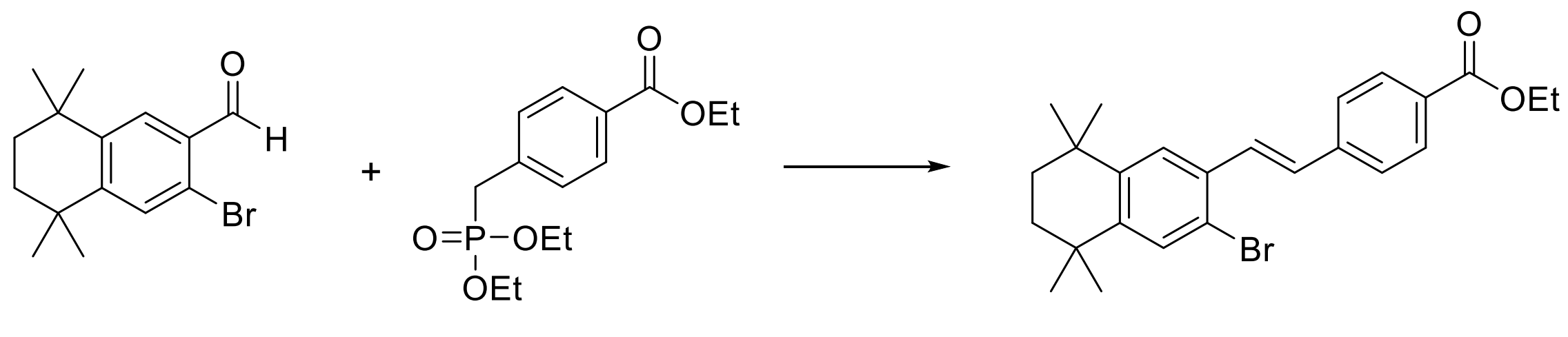

A 25 mL two-neck round bottom flask equipped with a stirrer bar was charged with phosphonate (345 mg, 1.15 mmol, 1.15 eq.) in dry THF (15 mL). NaH (70 mg, 2.9 mmol, 2.9 eq.) was added at 0 °C followed by aldehyde **33** (295 mg, 1 mmol, 1 eq.). The reaction mixture was stirred at 0 °C for 10 mins and then at room temperature for 3 hours under N_2. The reaction mixture was quenched with H_2O (5 mL) and extracted with EtOAc (3 × 20 mL). The combined extracts were dried ($MgSO_4$) and concentrated in vacuo. The cude material was purified by

column chromatography (hexanes: EtOAc =95:5) to produce the title compound **34** as colorless oil (395 mg, 90% yield).

^{1}H NMR (400 MHz, CDCl$_3$) δ 8.24 – 7.86 (m, 2H), 7.70 – 7.56 (m, 3H), 7.57 – 7.46 (m, 2H), 7.01 (d, *J* = 16.0 Hz, 1H), 4.40 (q, *J* = 7.0 Hz, 2H), 1.70 (s, 4H), 1.42 (t, *J* = 7.0 Hz, 3H), 1.34 (s, 6H), 1.29 (s, 6H). **^{13}C NMR (101 MHz, CDCl$_3$)** δ 166.5, 147.6, 144.9, 141.7, 133.8, 131.2, 130.2, 130.1, 129.6, 129.4, 126.6, 125.1, 121.7, 61.1, 35.0, 34.9, 34.6, 34.4, 31.9, 31.8, 14.5.

HRMS (ESI$^+$) $C_{25}H_{30}BrO_2$ requires $(M+H)^+$ 441.1429, found 441.1427.

Synthesis of ethyl 4-[(E)-2-{3-[(3-tert-butyl-5-methyl-1H-pyrazol-1-yl)methyl]-5,5,8,8-tetramethyl-5,6,7,8-tetrahydronaphthalen-2-yl}ethenyl]benzoate (35)

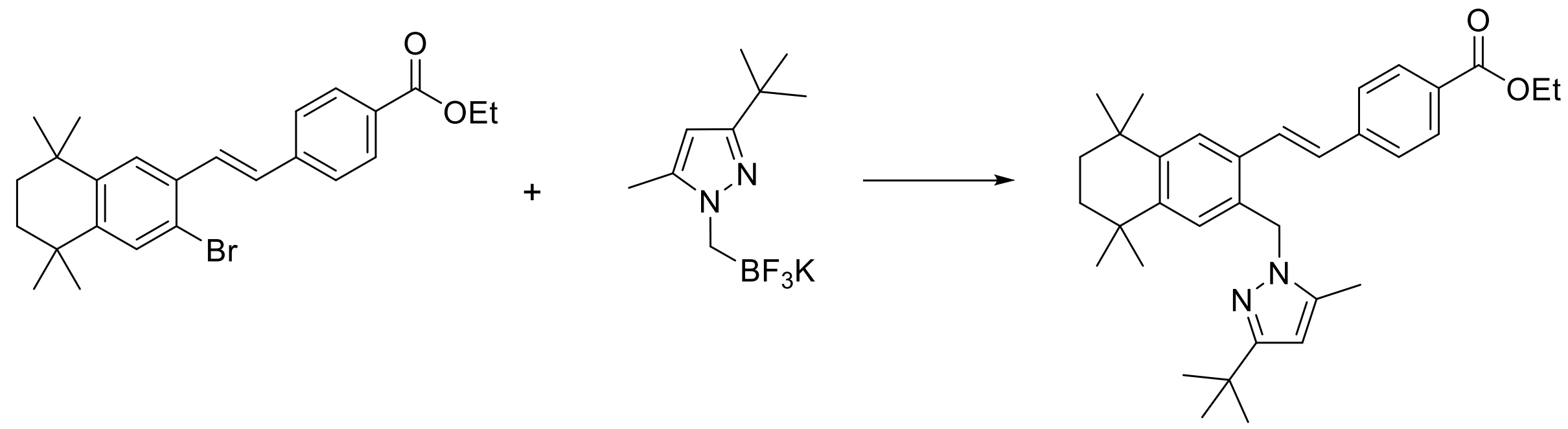

A 10 mL round bottom flask equipped with a stirrer bar was charged with potassium 3-*tert*-butyl-5-methyl(trifluoro(1-methylpyrazolyl)borate (38.7 mg, 0.15 mmol, 1.25 equiv), Cs_2CO_3 (98 mg, 0.3 mmol, 3 equiv), ethyl 4-[(E)-2-(3-bromo-5,5,8,8-tetramethyl-5,6,7,8-tetrahydronaphthalen-2-yl)ethenyl]benzoate (52 mg, 0.12 mmol, 1 equiv), $[PdCl(allyl)]_2$ (3.6 mg, 0.01 mmol, 10 mol %), and ^{S}SPhos (10.6 mg, 0.02 mmol, 20 mol %). CPME (1 mL) and H_2O (0.25 mL) were added by syringe, and the reaction mixture was stirred and heated at 100 °C for 24 h under N_2. The reaction mixture was then cooled to rt and diluted with H_2O (3 mL), extracted with EtOAc (5 × 10 mL). The combined organics were dried ($MgSO_4$), filtered through Celite, and concentrated in vacuo. The product **35** isolated via column chromatography (hexanes: EtOAc = 9:1) as yellow oil (31.7 mg, 52% yield).

^{1}H NMR (400 MHz, CDCl$_3$) δ 8.07 – 8.00 (m, 2H), 7.69 – 7.57 (m, 3H), 7.53 (s, 1H), 6.96 (d, *J* = 16.0 Hz, 1H), 6.57 (s, 1H), 5.93 (s, 1H), 5.36 (s, 2H), 4.40 (q, *J* = 7.0 Hz, 2H), 2.13 (s, 3H), 1.67 (s, 4H), 1.42 (t, *J* = 7.0 Hz, 3H), 1.36 – 1.26 (m, 15H), 1.17 (s, 6H). **^{13}C NMR (101 MHz, CDCl$_3$)** δ 166.6, 161.0, 145.5, 144.6, 142.1, 139.3, 132.8, 132.3, 130.1, 129.3, 129.2, 128.5, 126.6, 126.4, 124.2, 102.5, 61.0, 51.6, 35.2, 35.0, 34.2 (x3C), 32.0, 31.7, 30.8, 14.5, 11.6.

HRMS (ESI$^+$) $C_{34}H_{45}N_2O_2$ requires $(M+H)^+$ 513.3476, found 513.3489.

Synthesis of ethyl 4-[(E)-2-{3-[(5-cyclopropyl-3-phenyl-1H-pyrazol-1-yl)methyl]-5,5,8,8-tetramethyl-5,6,7,8-tetrahydronaphthalen-2-yl}ethenyl]benzoate (36)

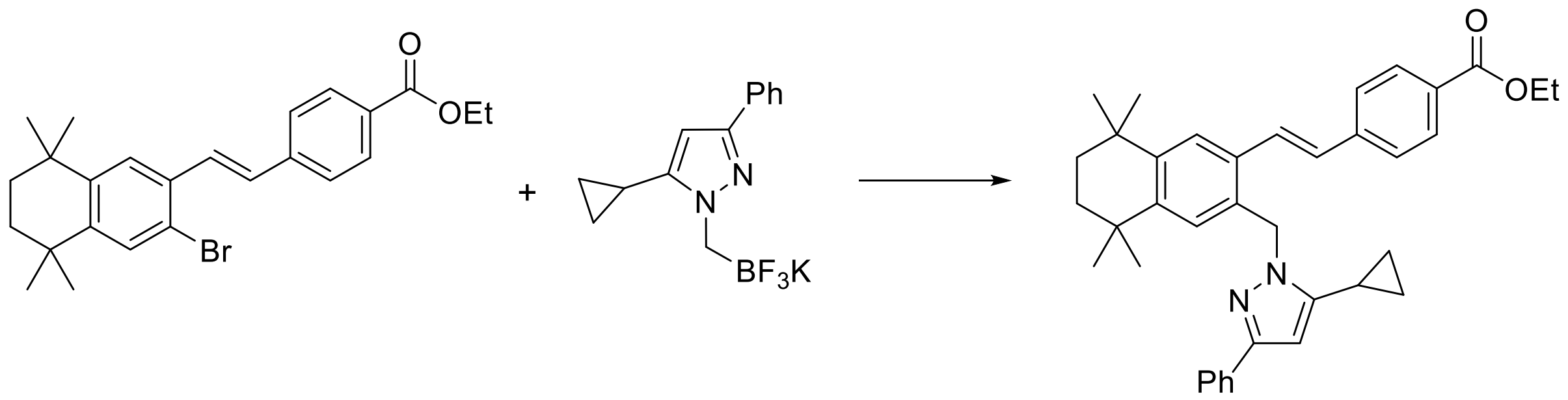

A 10 mL round bottom flask equipped with a stirrer bar was charged with potassium 3-phenyl 5-cyclopropyl-(trifluoro(1-methylpyrazolyl)borate (45.6 mg, 0.15 mmol, 1.5 equiv), Cs_2CO_3 (98 mg, 0.3 mmol, 3 equiv), ethyl 4-[(*E*)-2-(3-bromo-5,5,8,8-tetramethyl-5,6,7,8-tetrahydronaphthalen-2-yl)ethenyl]benzoate (44.1 mg, 0.1 mmol, 1 equiv), $[PdCl(allyl)]_2$ (3.6 mg, 0.01 mmol, 10 mol %), and ^{S}SPhos (10.6 mg, 0.02 mmol, 20 mol %). CPME (1 mL) and H_2O (0.25 mL) were added by syringe, and the reaction mixture was stirred and heated at 100 °C for 24 h under N_2. The reaction mixture was then cooled to rt and diluted with H_2O (3 mL), extracted with EtOAc (5 × 10 mL). The combined organics were dried ($MgSO_4$), filtered through Celite, and concentrated in vacuo. The product **36** isolated via column chromatography (hexanes: EtOAc = 9:1) as yellow oil (33.2 mg, 60% yield).

^{1}H NMR (400 MHz, CDCl$_3$) δ 7.93 (d, *J* = 8.5 Hz, 2H), 7.81 – 7.72 (m, 3H), 7.59 – 7.55 (m, 3H), 7.40 – 7.35 (m, 2H), 7.31 – 7.27 (m, 1H), 7.00 – 6.91 (m, 2H), 6.19 (s, 1H), 5.58 (s, 2H), 4.38 (q, *J* = 7.0 Hz, 2H), 1.68 (s, 4H), 1.63 – 1.55 (m, 1H), 1.42 (t, *J* = 7.0 Hz, 3H), 1.33 (s, 6H), 1.20 (s, 6H), 0.91 – 0.81 (m, 2H), 0.69 – 0.61 (m, 2H). **^{13}C NMR (101 MHz, CDCl$_3$)** δ 166.6, 150.0, 147.1, 145.4, 144.9, 142.2, 133.8, 133.3, 132.0, 130.1, 129.2, 129.0, 128.8, 128.6, 127.5, 127.4, 126.6, 125.6, 124.3, 100.5, 61.0, 52.7, 35.2, 35.0, 34.31, 34.30, 32.0, 31.8, 14.5, 7.3, 6.8.

HRMS (ESI$^+$) $C_{38}H_{43}N_2O_2$ requires $(M+H)^+$ 559.3319, found 559.3307.

NMR Spectra

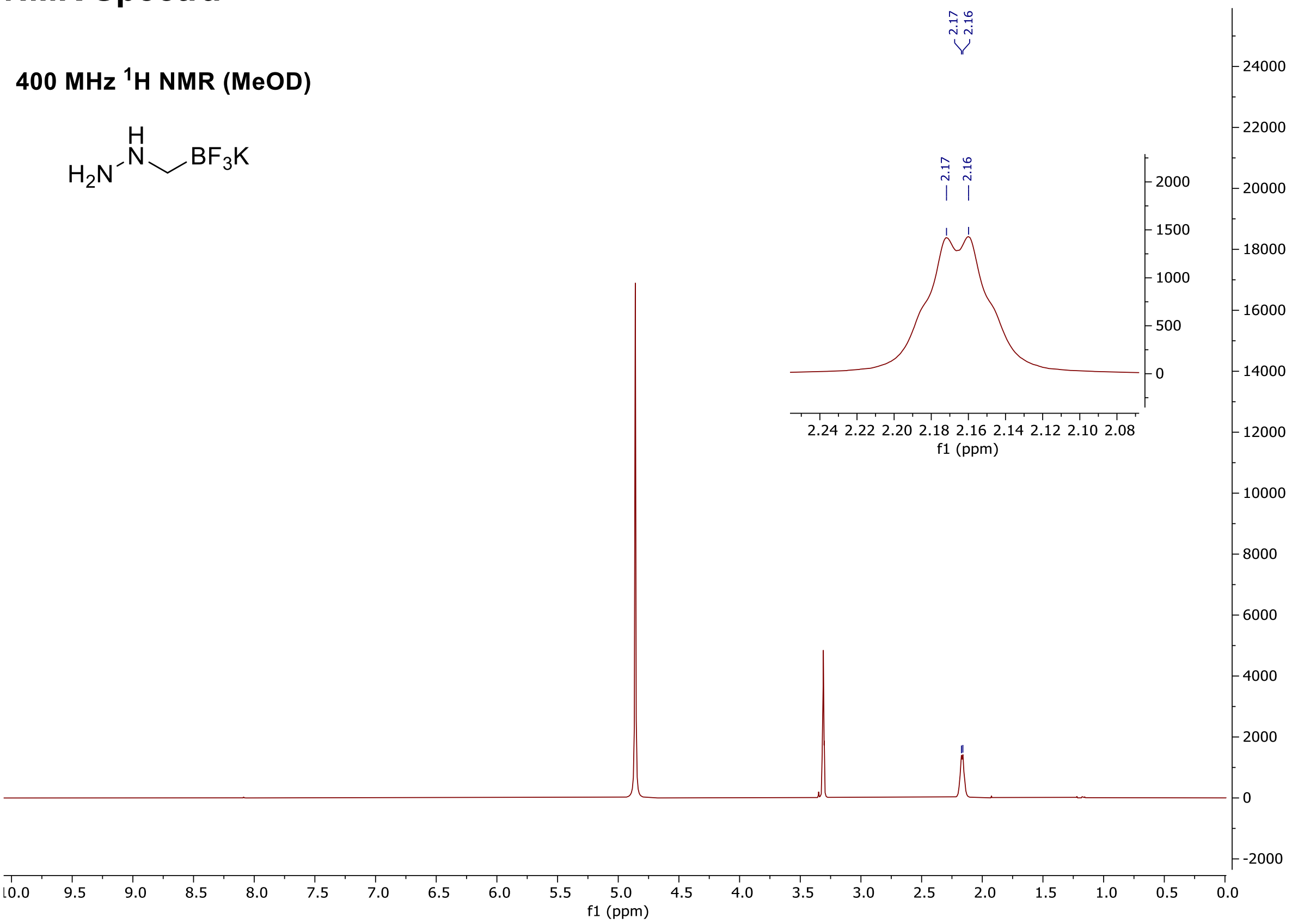
400 MHz 1H NMR (MeOD)
H2N-NH-CH2-BF3K
2.17
2.16
f1 (ppm)

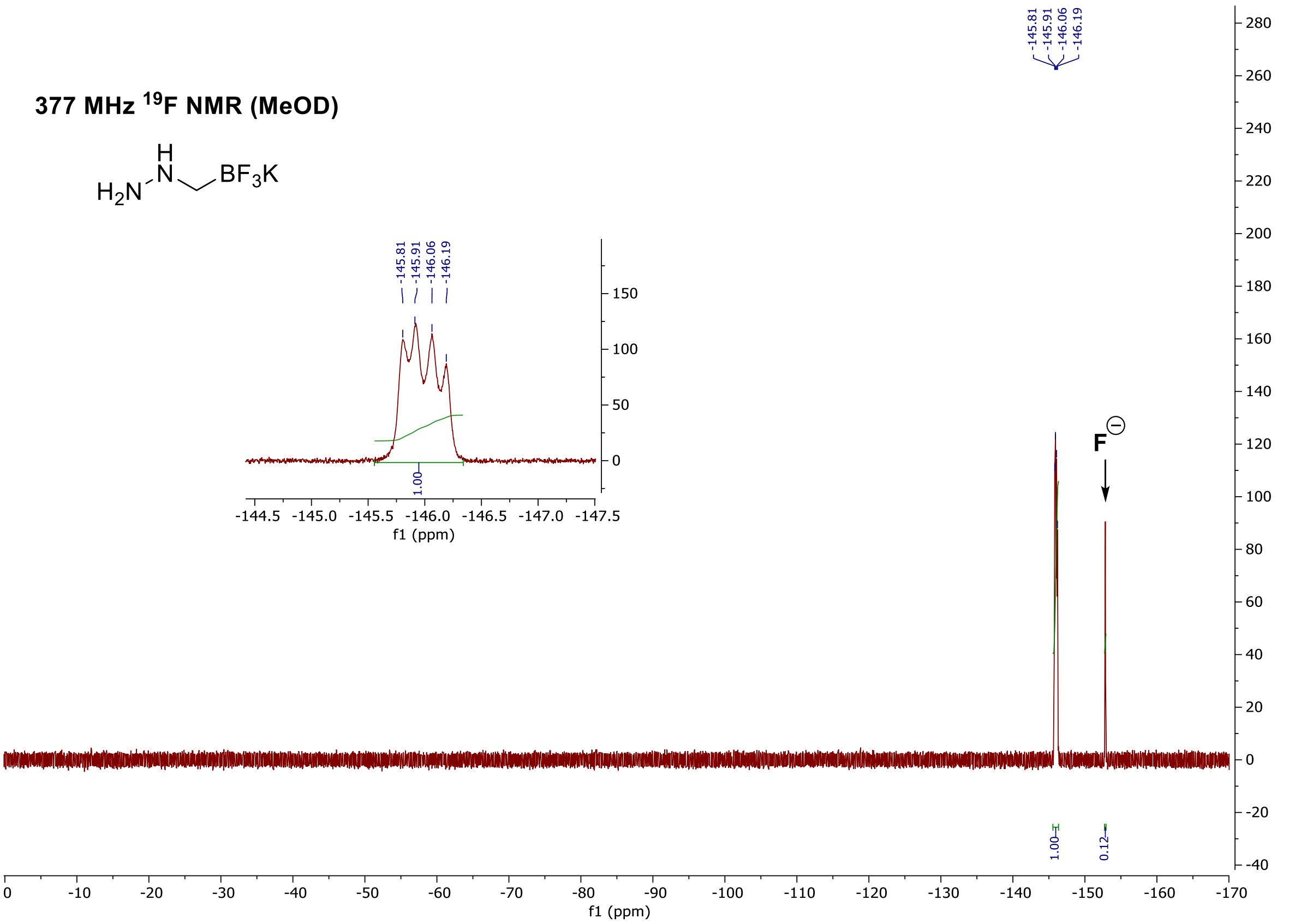
377 MHz 19F NMR (MeOD)
H2N-NH-CH2-BF3K
-145.81
-145.91
-146.06
-146.19
F⊖
1.00
0.12
f1 (ppm)

128 MHz ^{11}B NMR (MeOD)

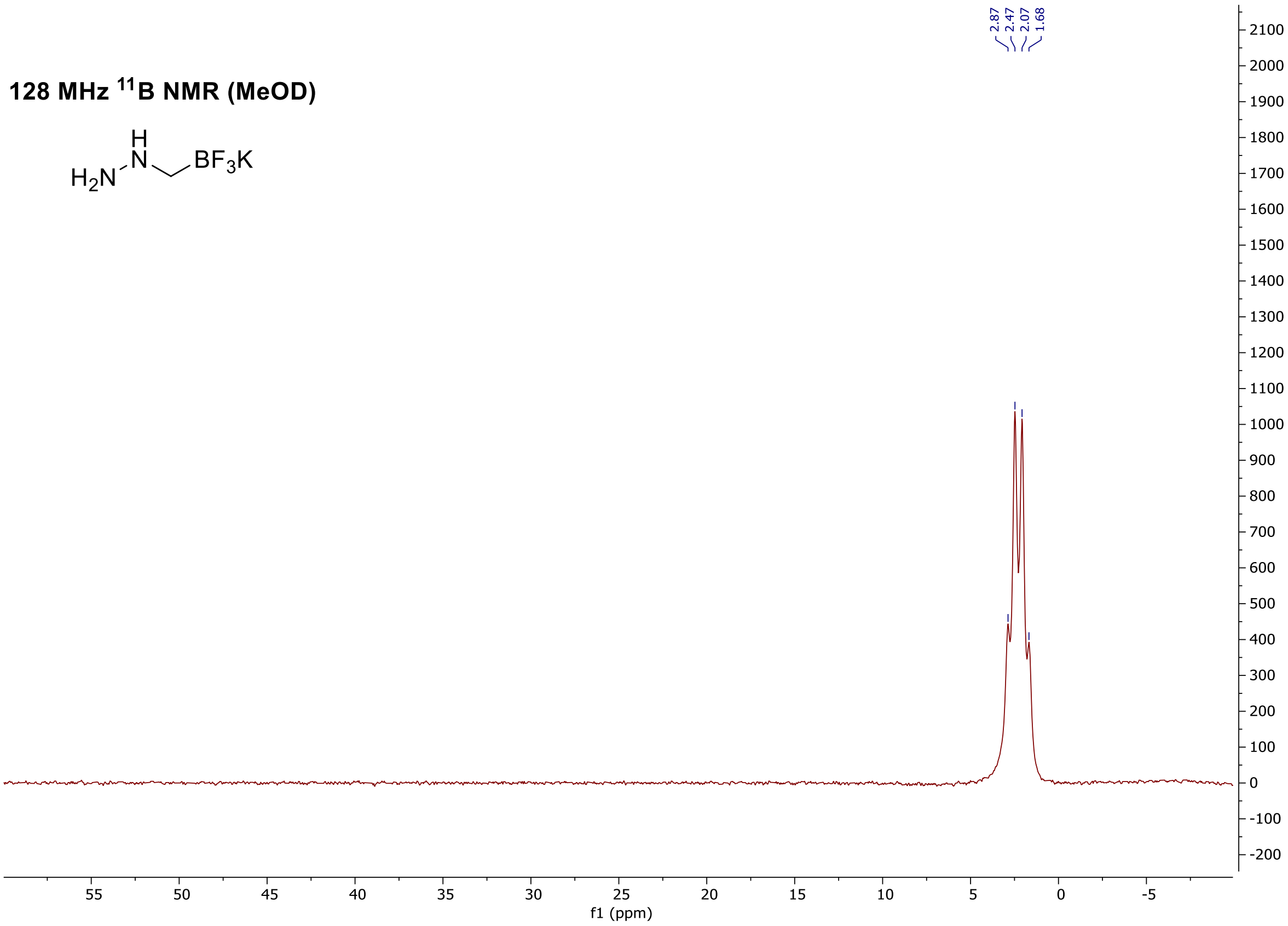

400 MHz ^{1}H NMR (MeOD)

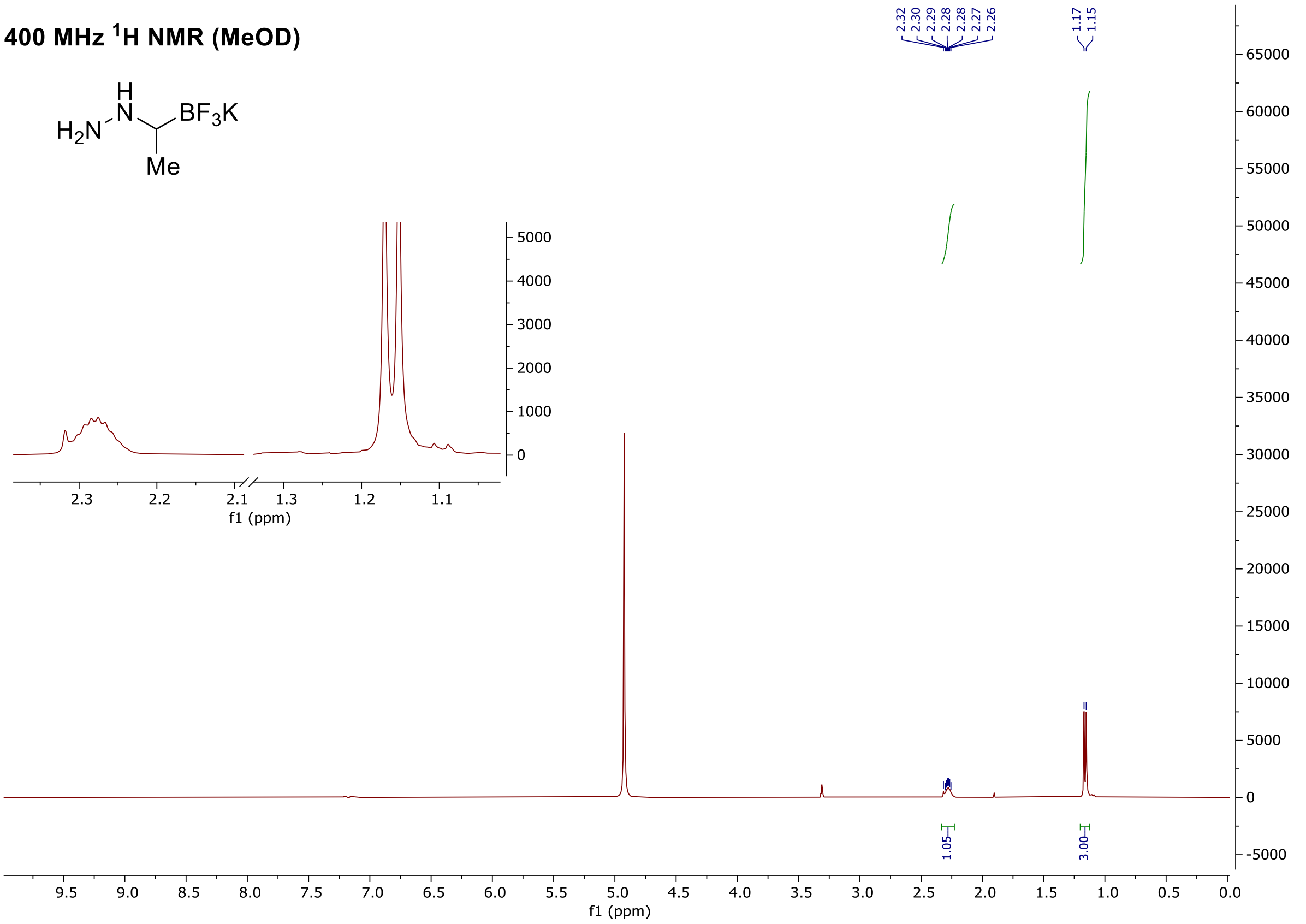

101 MHz {^{1}H}^{13}C NMR (MeOD)

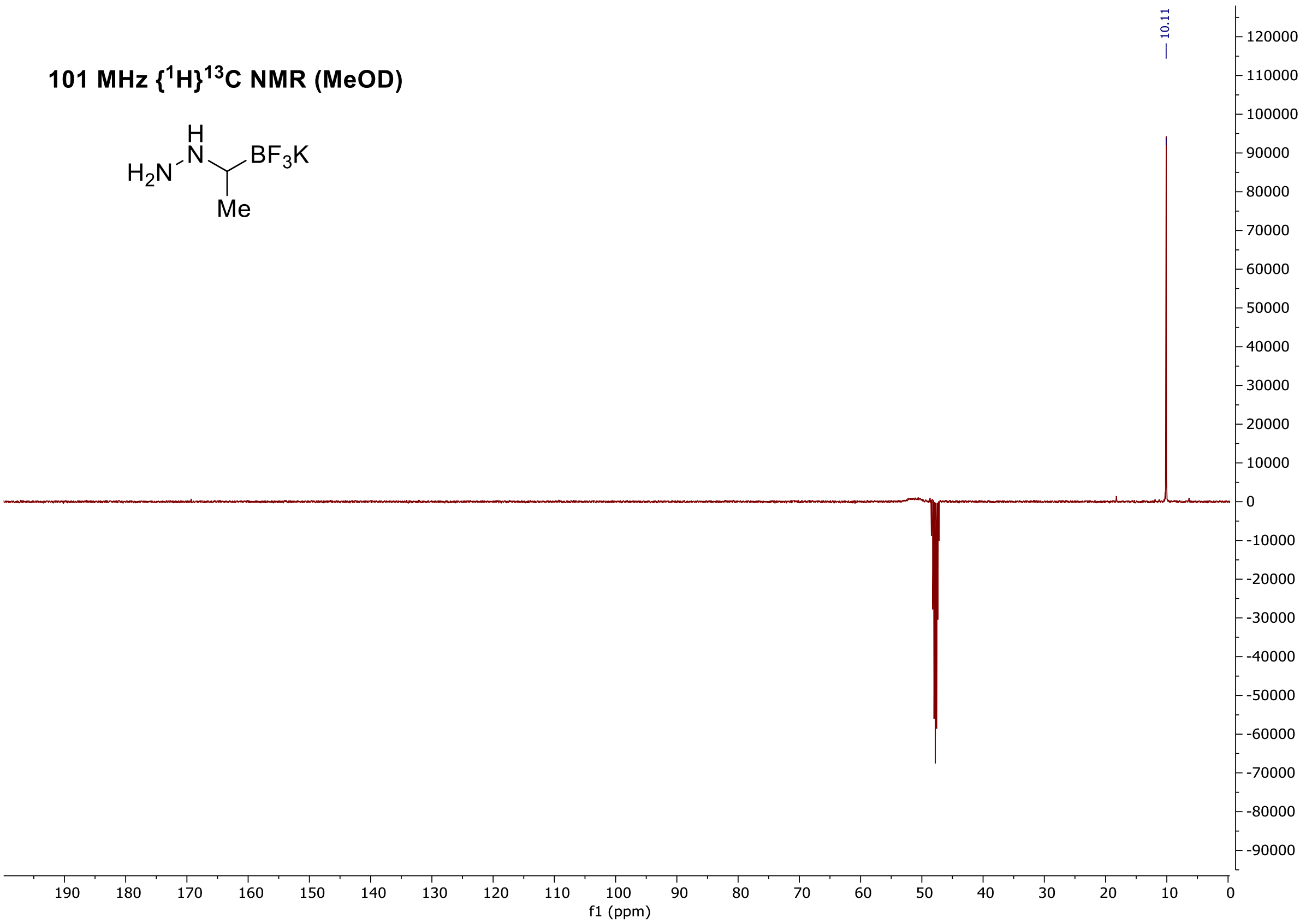

377 MHz ^{19}F NMR (MeOD)

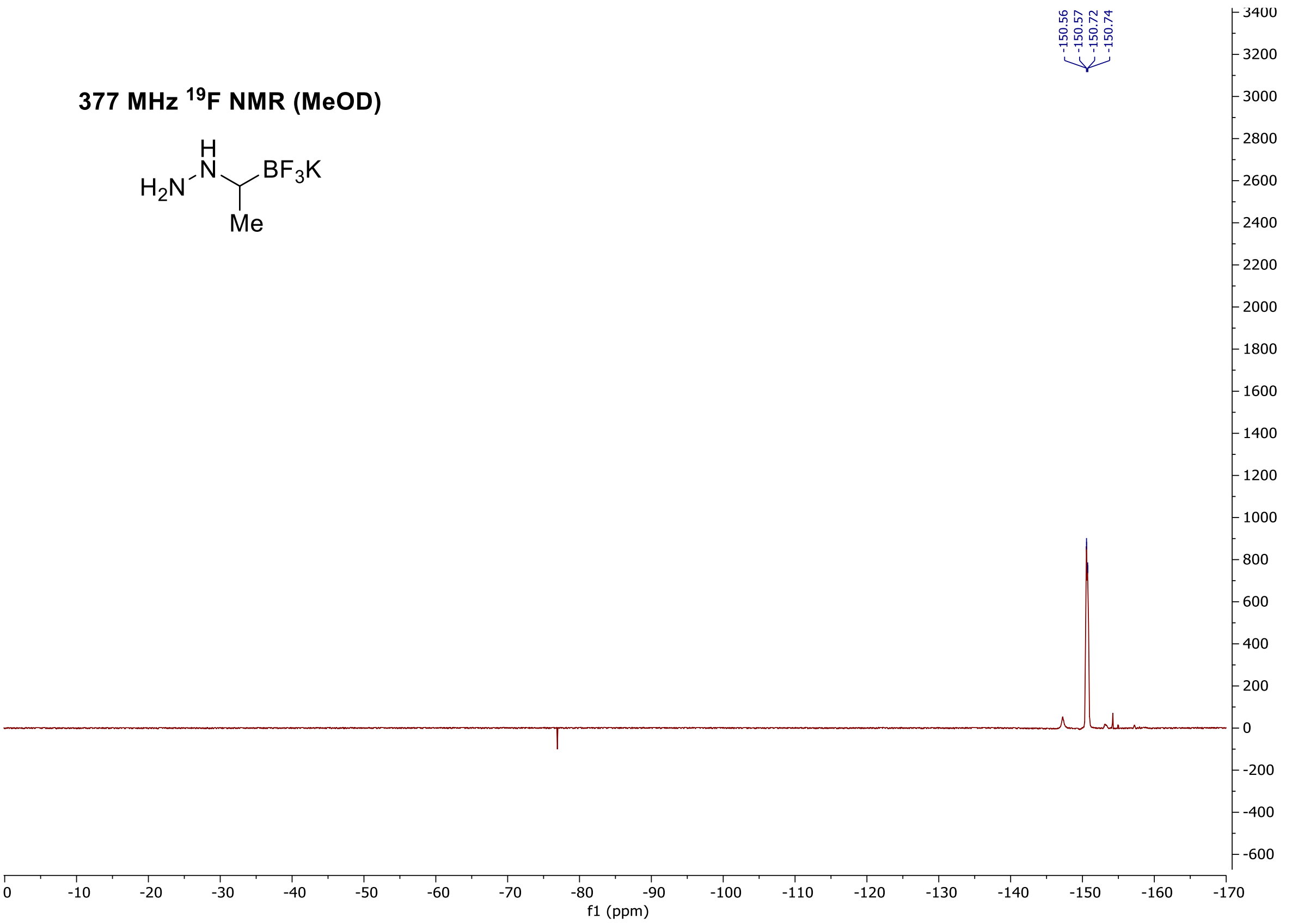

128 MHz ^{11}B NMR (MeOD)

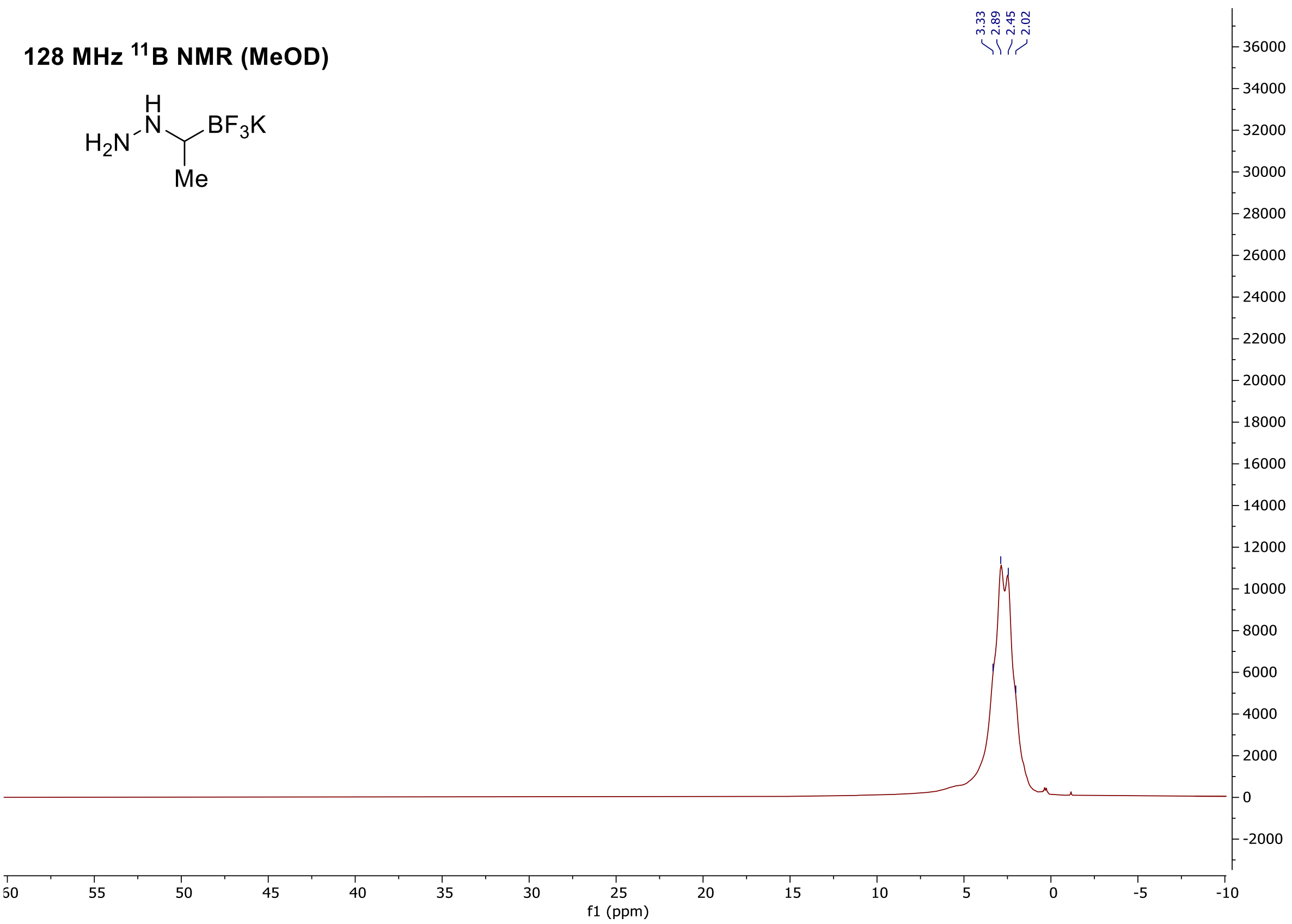

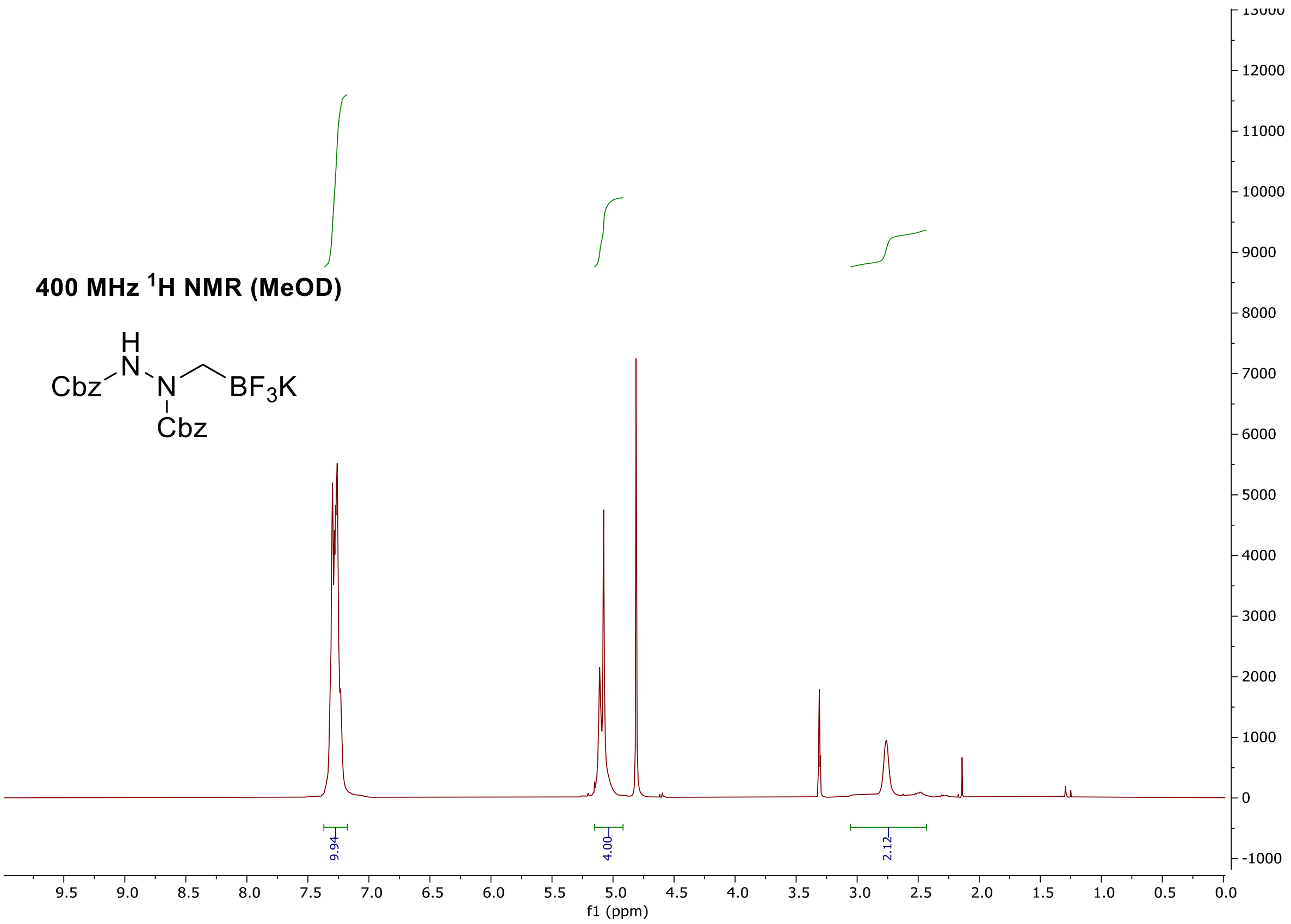
400 MHz ¹H NMR (MeOD)
Cbz
N
H
N
BF3K
Cbz
9.94
4.00
2.12
f1 (ppm)

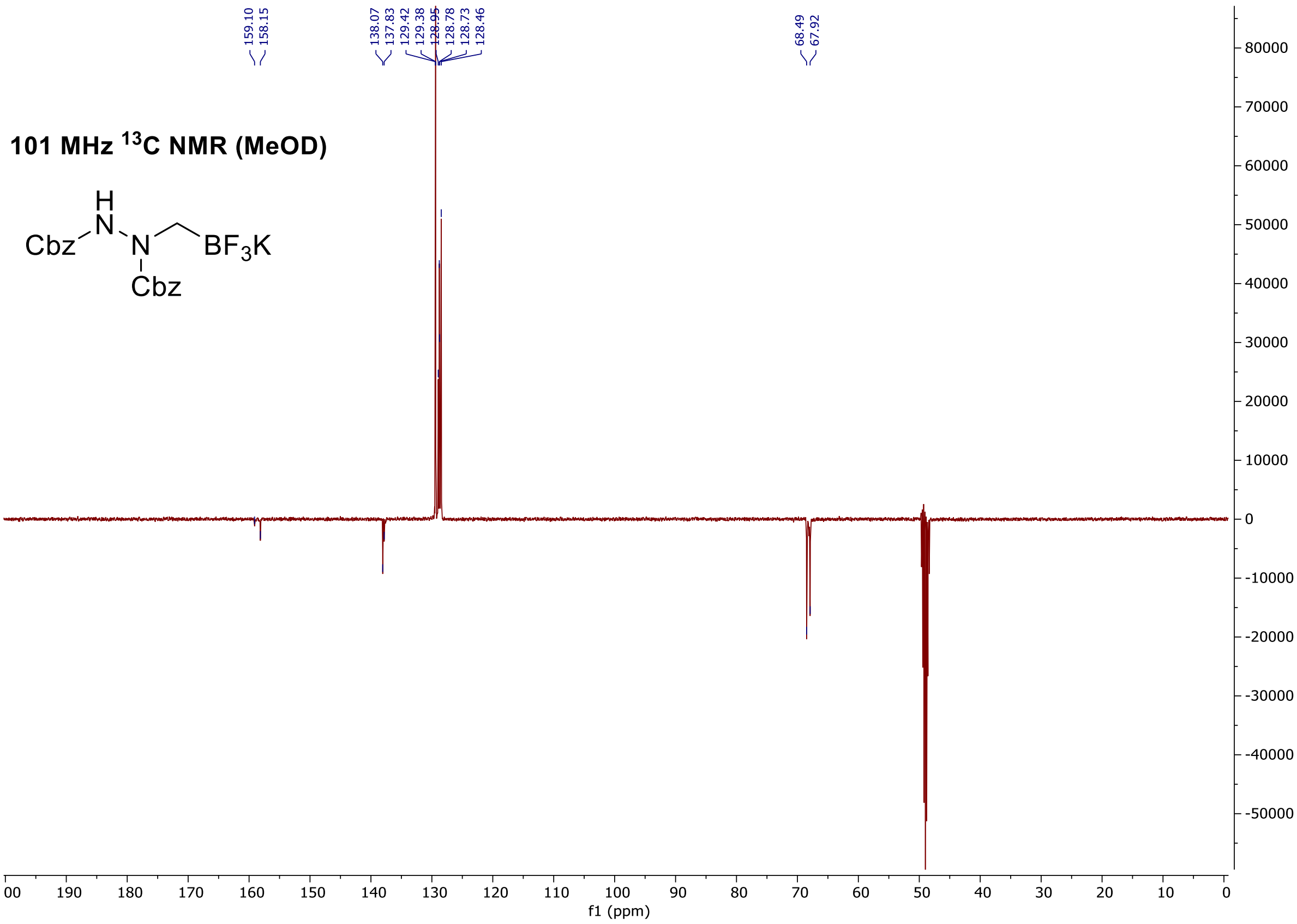
101 MHz ¹³C NMR (MeOD)
159.10
158.15
138.07
137.83
129.42
129.38
128.95
128.78
128.73
128.46
68.49
67.92
Cbz
N
H
N
BF3K
Cbz
f1 (ppm)

376 MHz ^{19}F NMR (MeOD)

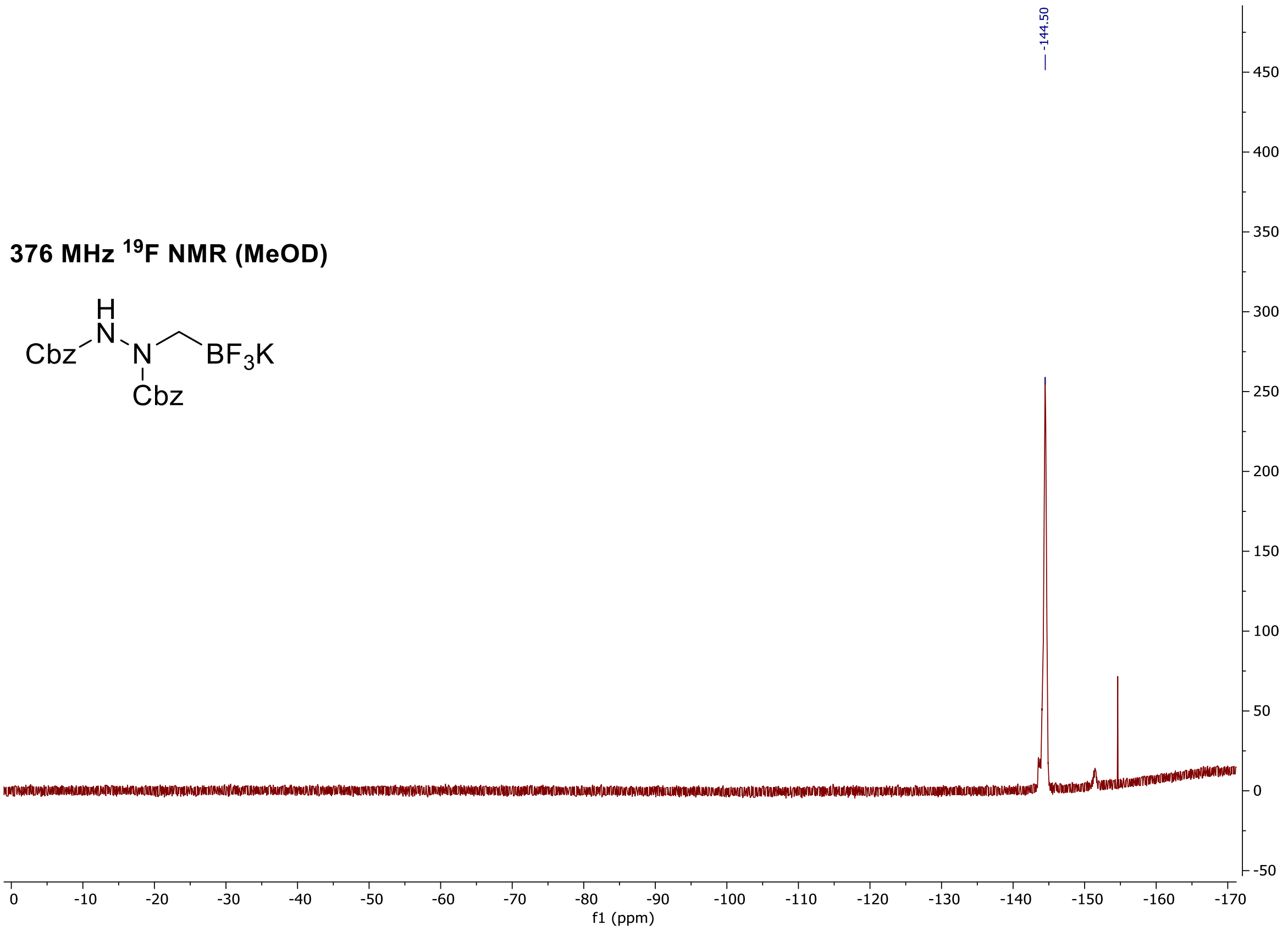

128 MHz ^{11}B NMR (MeOD)

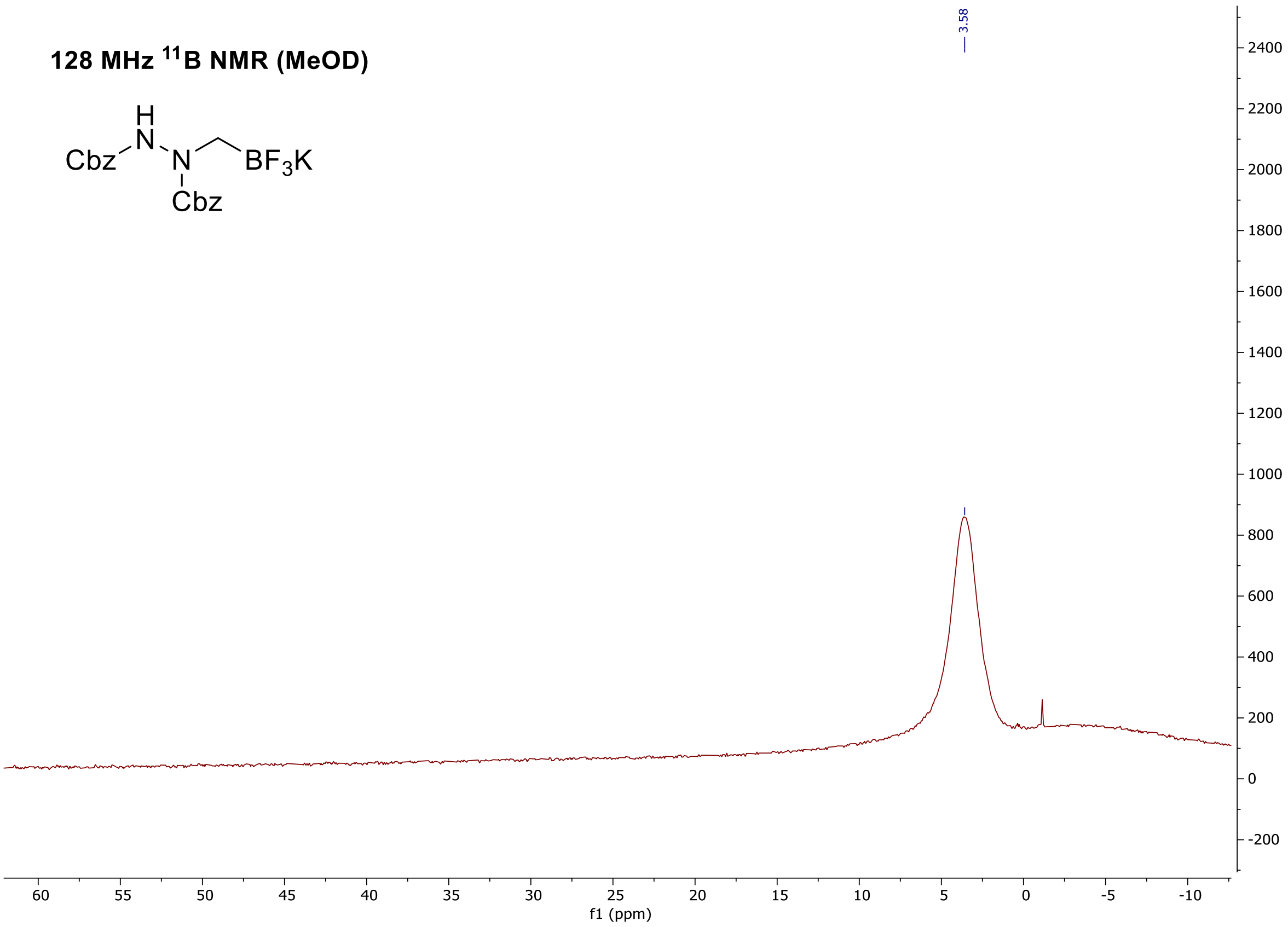

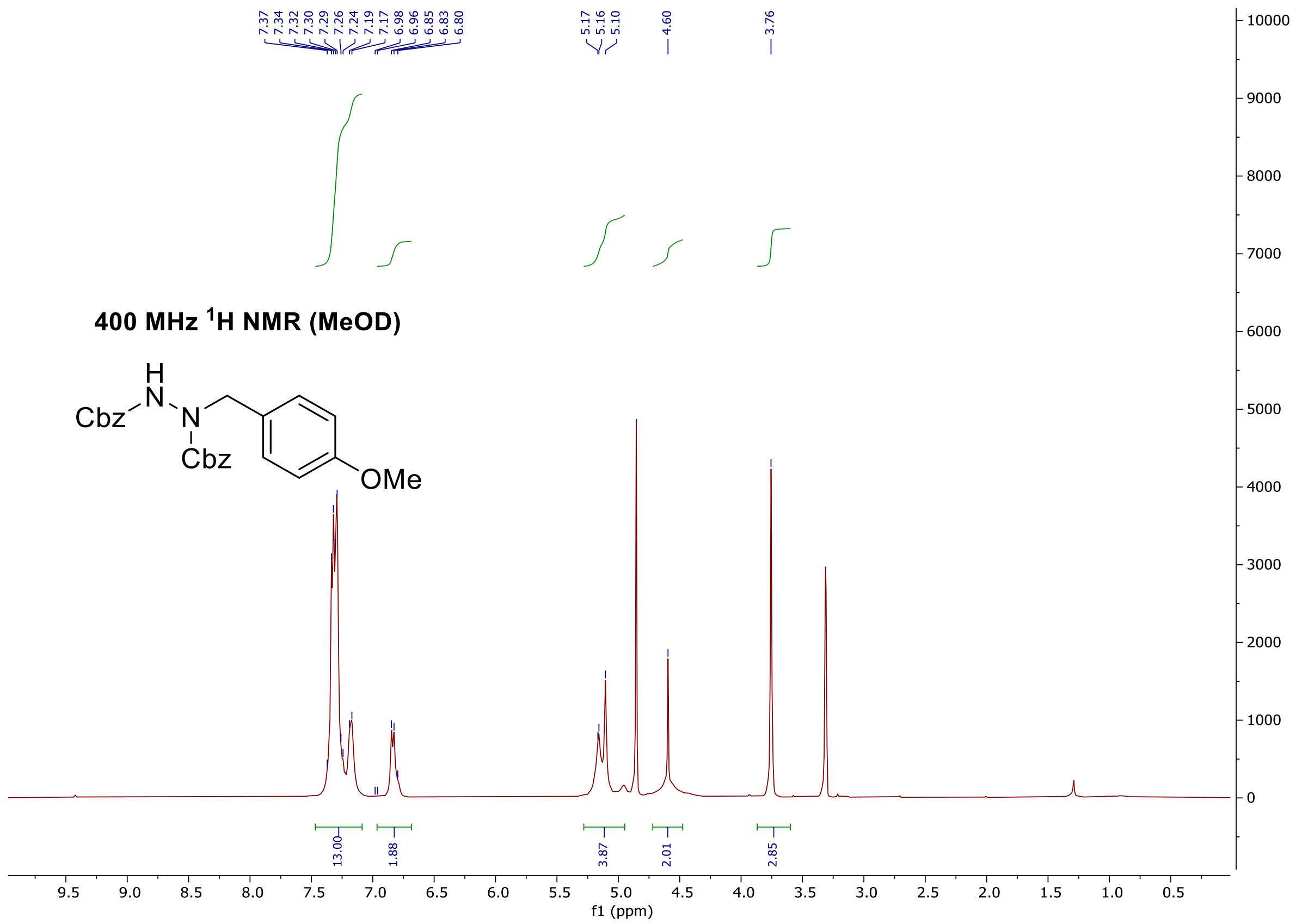
400 MHz ¹H NMR (MeOD)
Cbz
N
H
N
Cbz
OMe
7.37
7.34
7.32
7.30
7.29
7.26
7.24
7.19
7.17
6.98
6.96
6.85
6.83
6.80
5.17
5.16
5.10
4.60
3.76
13.00
1.88
3.87
2.01
2.85
f1 (ppm)

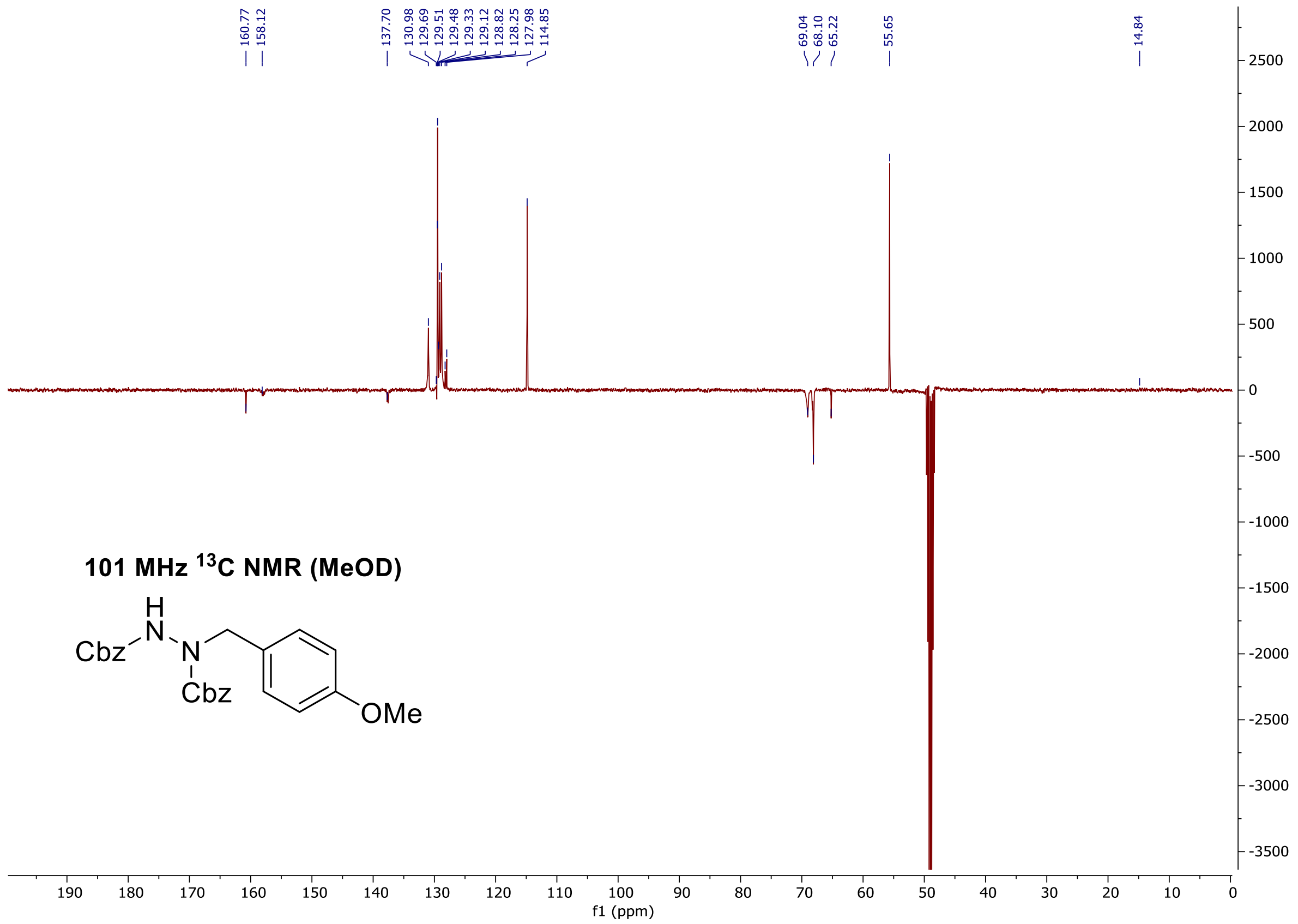
101 MHz ¹³C NMR (MeOD)
Cbz
N
H
N
Cbz
OMe
160.77
158.12
137.70
130.98
129.69
129.51
129.48
129.33
129.12
128.82
128.25
127.98
114.85
69.04
68.10
65.22
55.65
14.84
f1 (ppm)

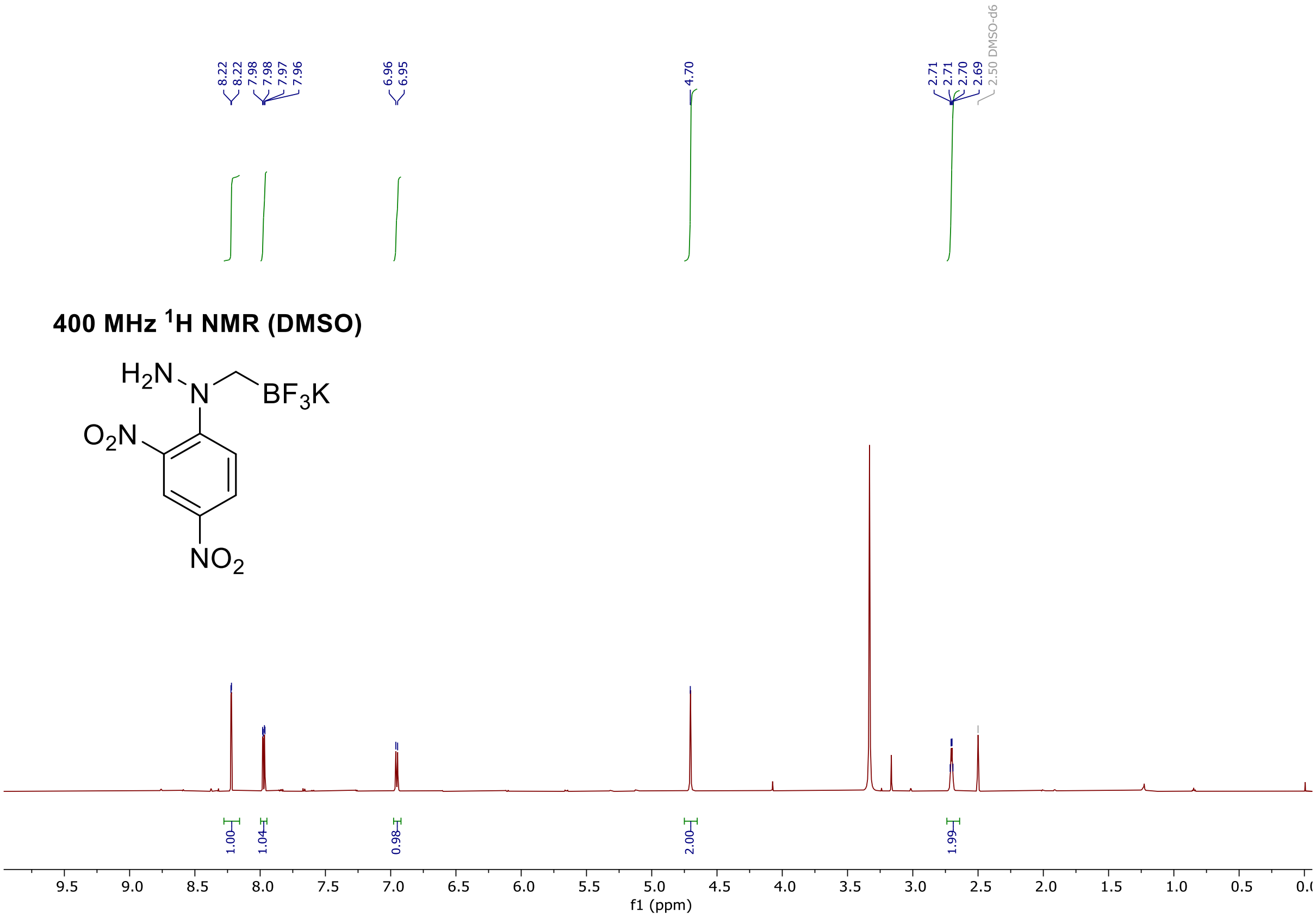
8.22
8.22
7.98
7.98
7.97
7.96
6.96
6.95
4.70
2.71
2.71
2.70
2.69
2.50 DMSO-d6
400 MHz ^{1}H NMR (DMSO)
H_2N
N
BF_3K
O_2N
NO_2
1.00
1.04
0.98
2.00
1.99
9.5
9.0
8.5
8.0
7.5
7.0
6.5
6.0
5.5
5.0
4.5
4.0
3.5
3.0
2.5
2.0
1.5
1.0
0.5
f1 (ppm)

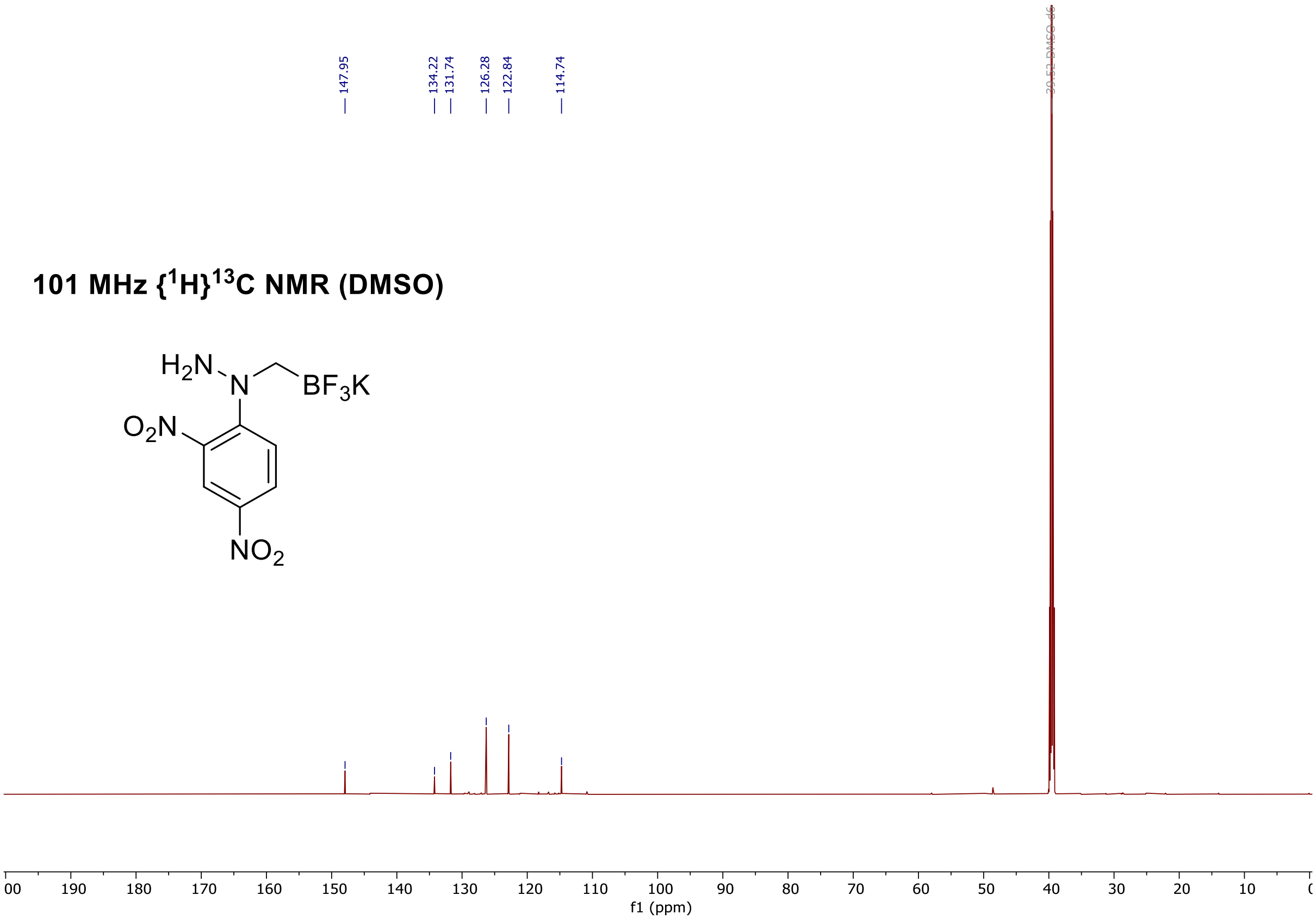
147.95
134.22
131.74
126.28
122.84
114.74
101 MHz {^{1}H}^{13}C NMR (DMSO)
H_2N
N
BF_3K
O_2N
NO_2
190
180
170
160
150
140
130
120
110
100
90
80
70
60
50
40
30
20
10
f1 (ppm)

377 MHz ^{19}F NMR (DMSO)

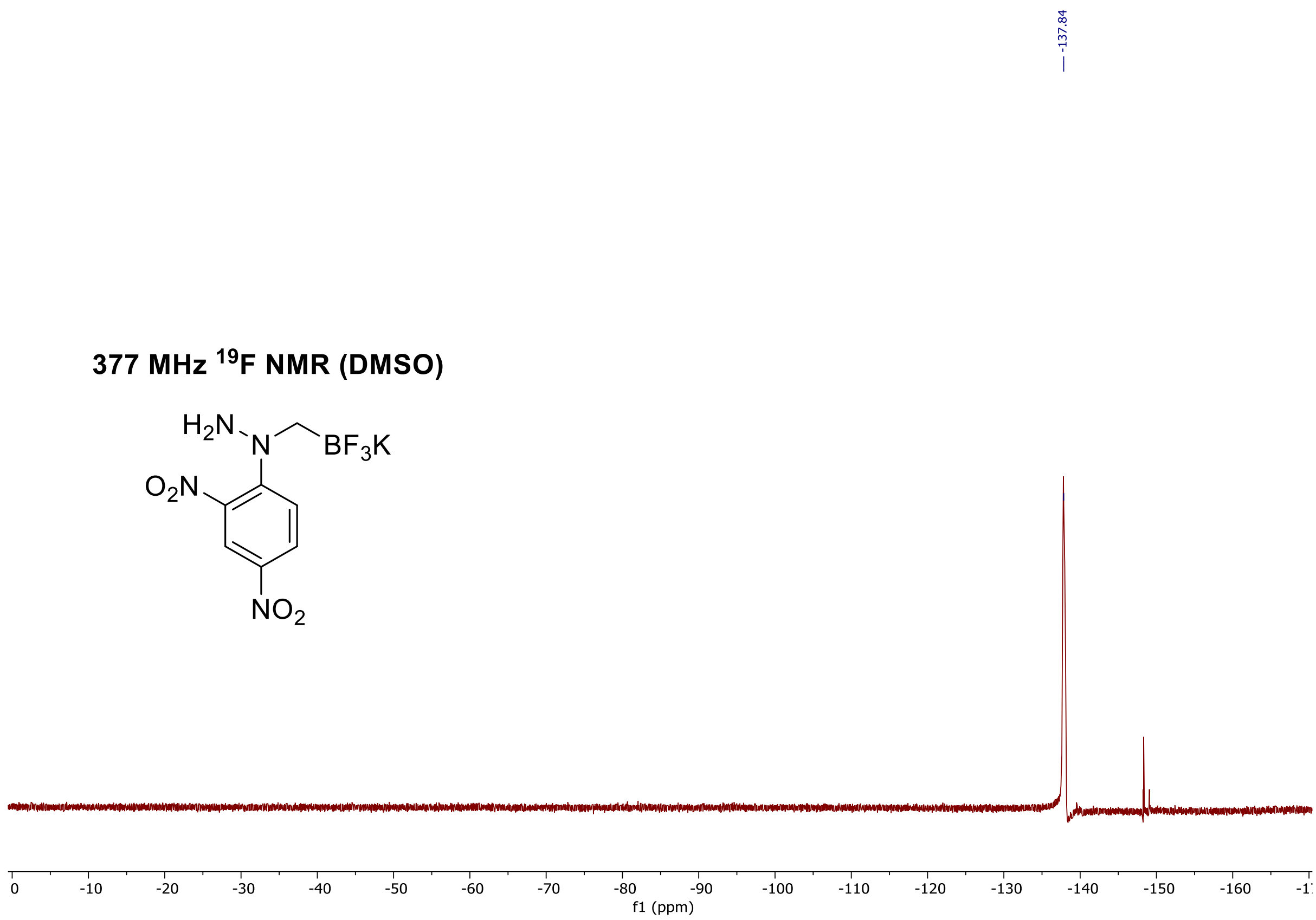

128 MHz ^{11}B NMR (DMSO)

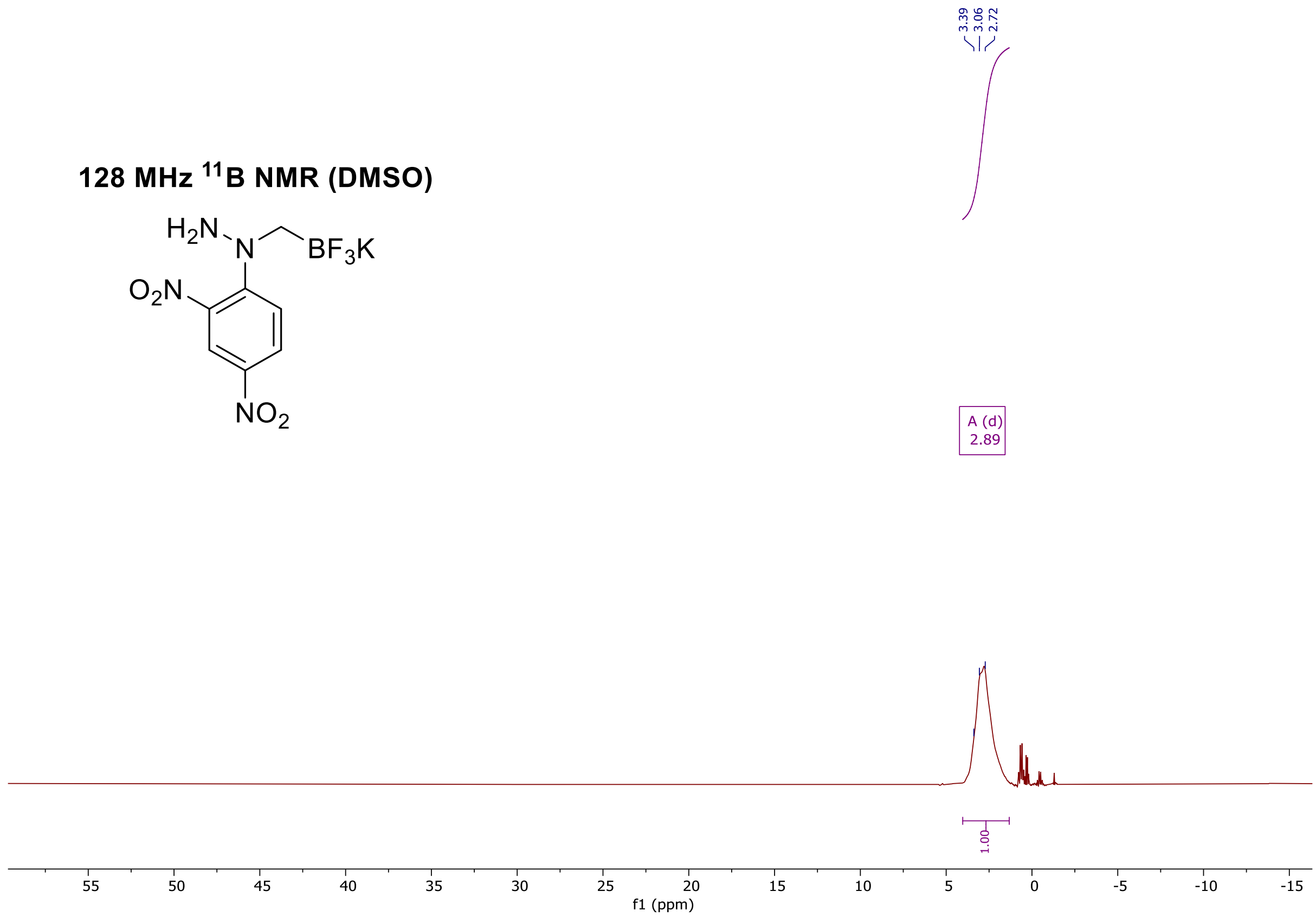

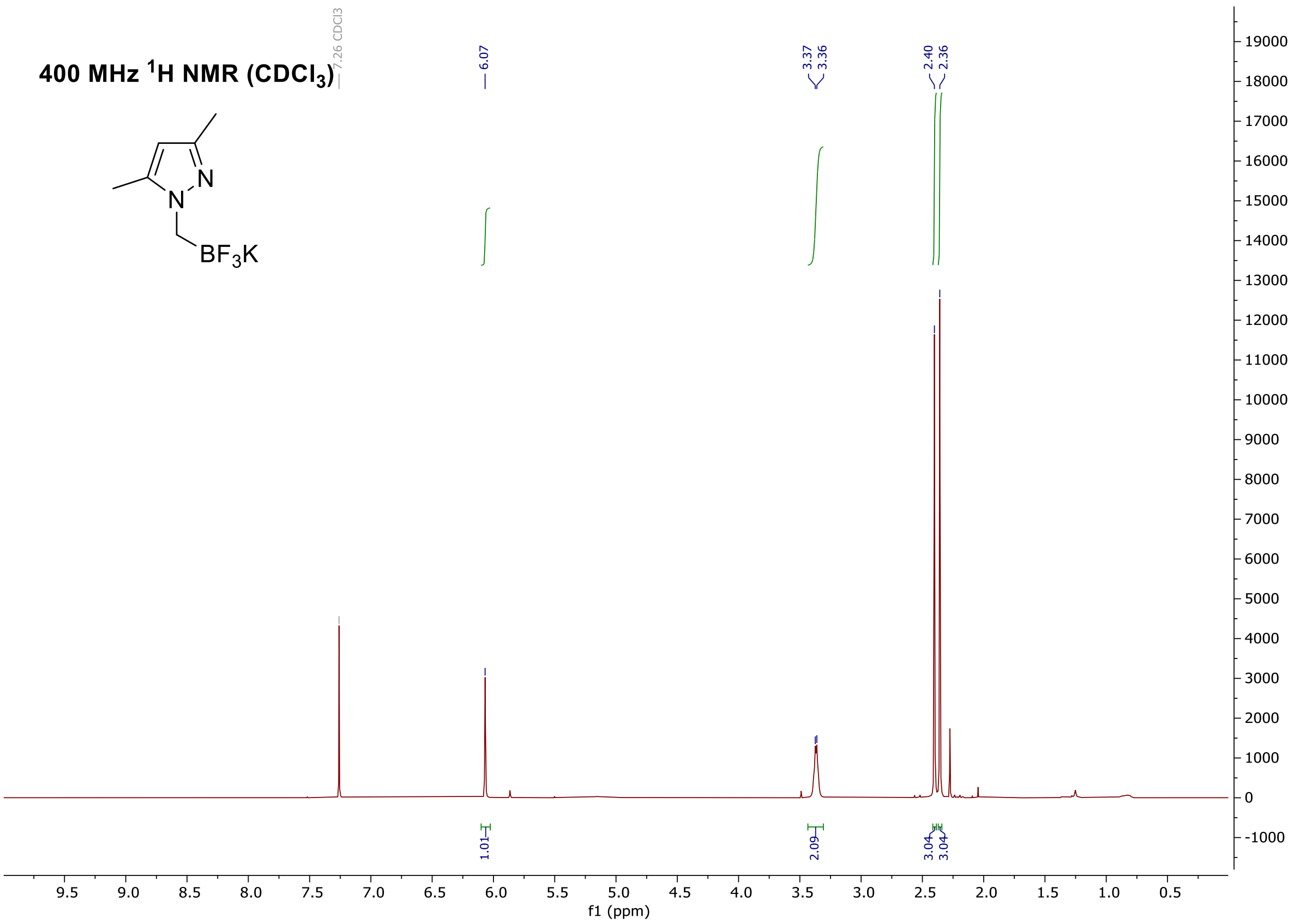
400 MHz ¹H NMR (CDCl₃)
BF₃K
N
N
7.26 CDCl3
6.07
3.37
3.36
2.40
2.36
1.01
2.09
3.04
3.04
f1 (ppm)

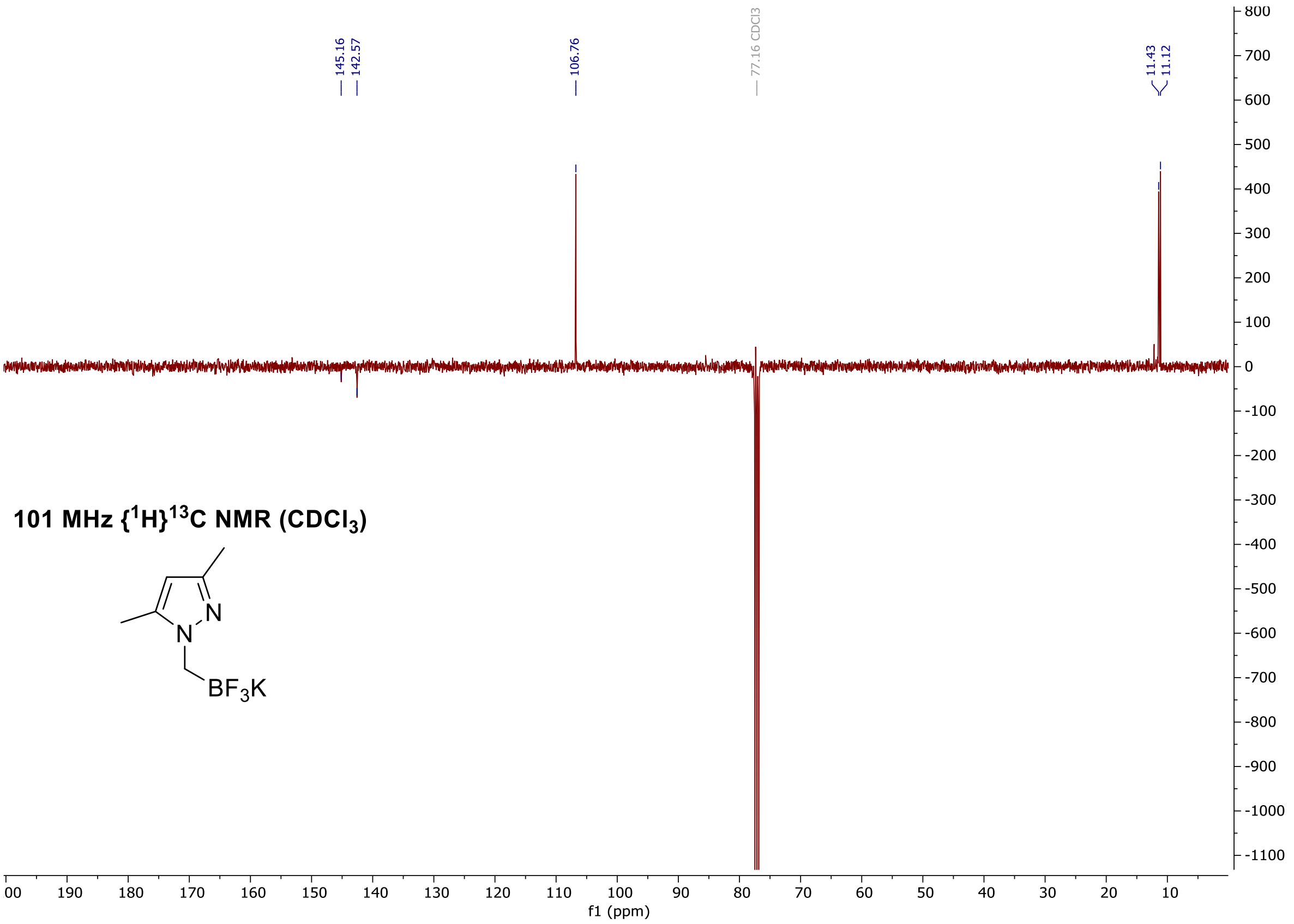
101 MHz {¹H}¹³C NMR (CDCl₃)
BF₃K
N
N
145.16
142.57
106.76
77.16 CDCl3
11.43
11.12
f1 (ppm)

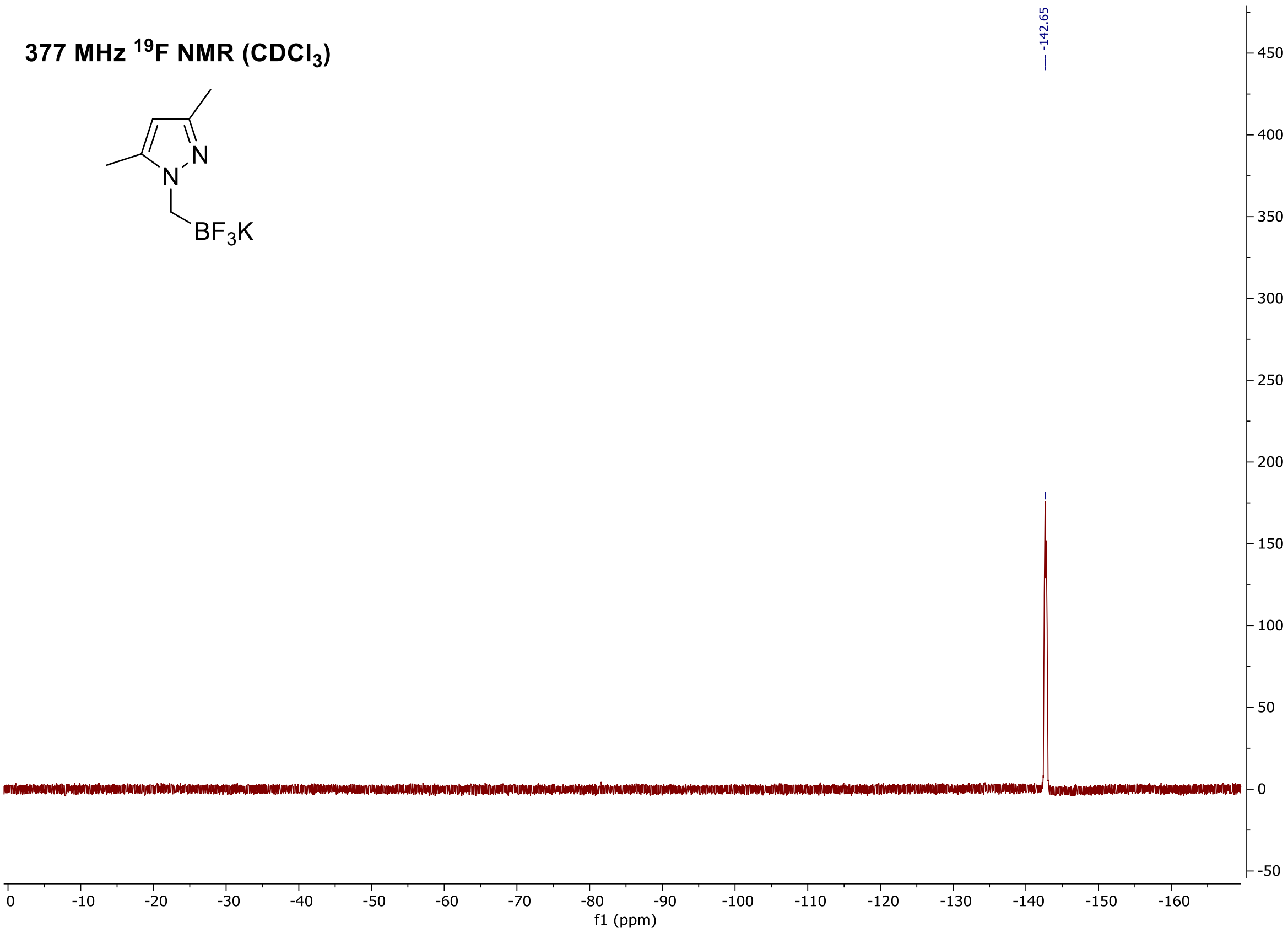
377 MHz 19F NMR (CDCl3)
BF3K
N
N
-142.65
450
400
350
300
250
200
150
100
50
0
-50
0 -10 -20 -30 -40 -50 -60 -70 -80 -90 -100 -110 -120 -130 -140 -150 -160
f1 (ppm)

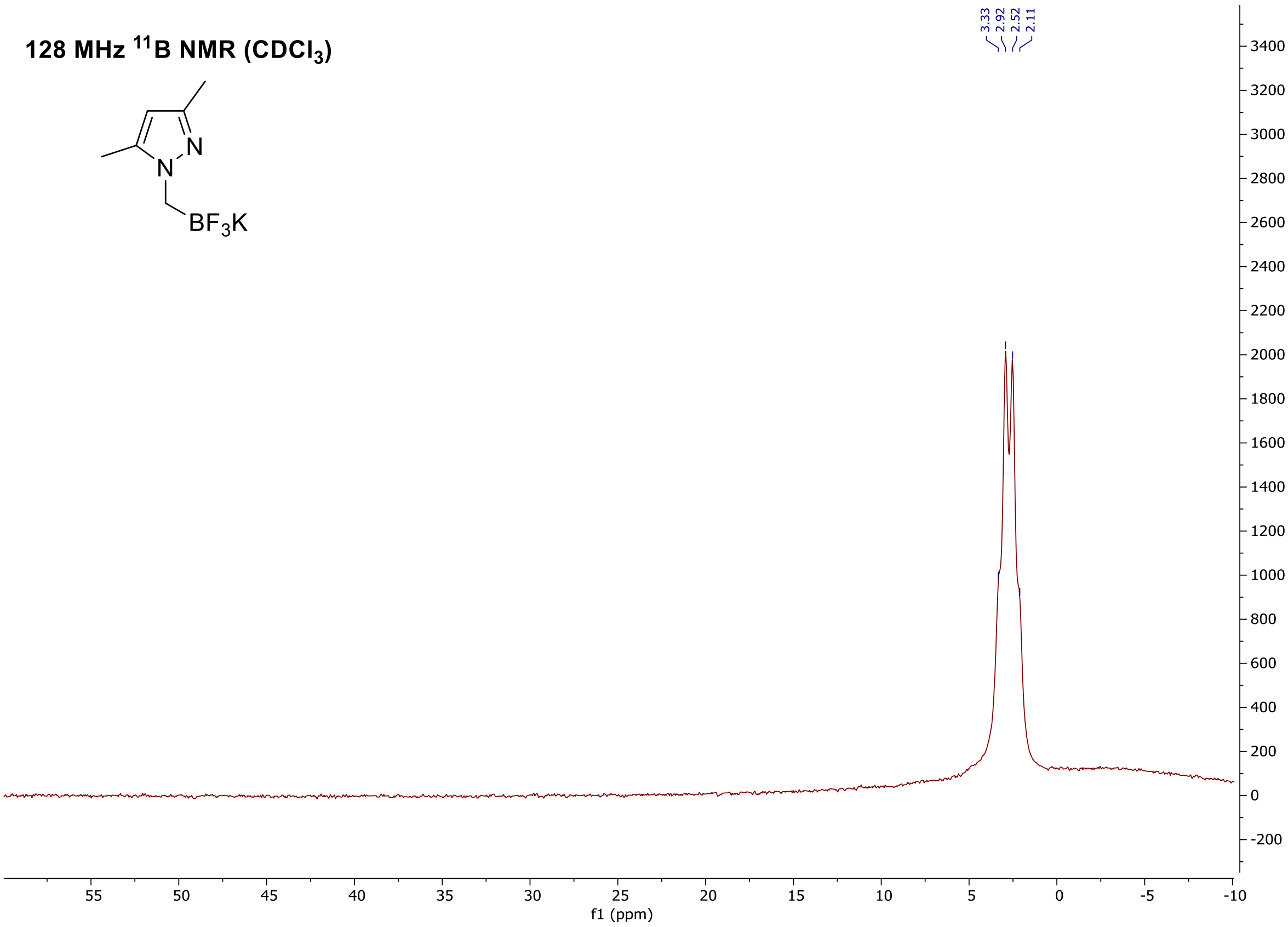
128 MHz 11B NMR (CDCl3)
BF3K
N
N
3.33
2.92
2.52
2.11
3400
3200
3000
2800
2600
2400
2200
2000
1800
1600
1400
1200
1000
800
600
400
200
0
-200
55 50 45 40 35 30 25 20 15 10 5 0 -5 -10
f1 (ppm)

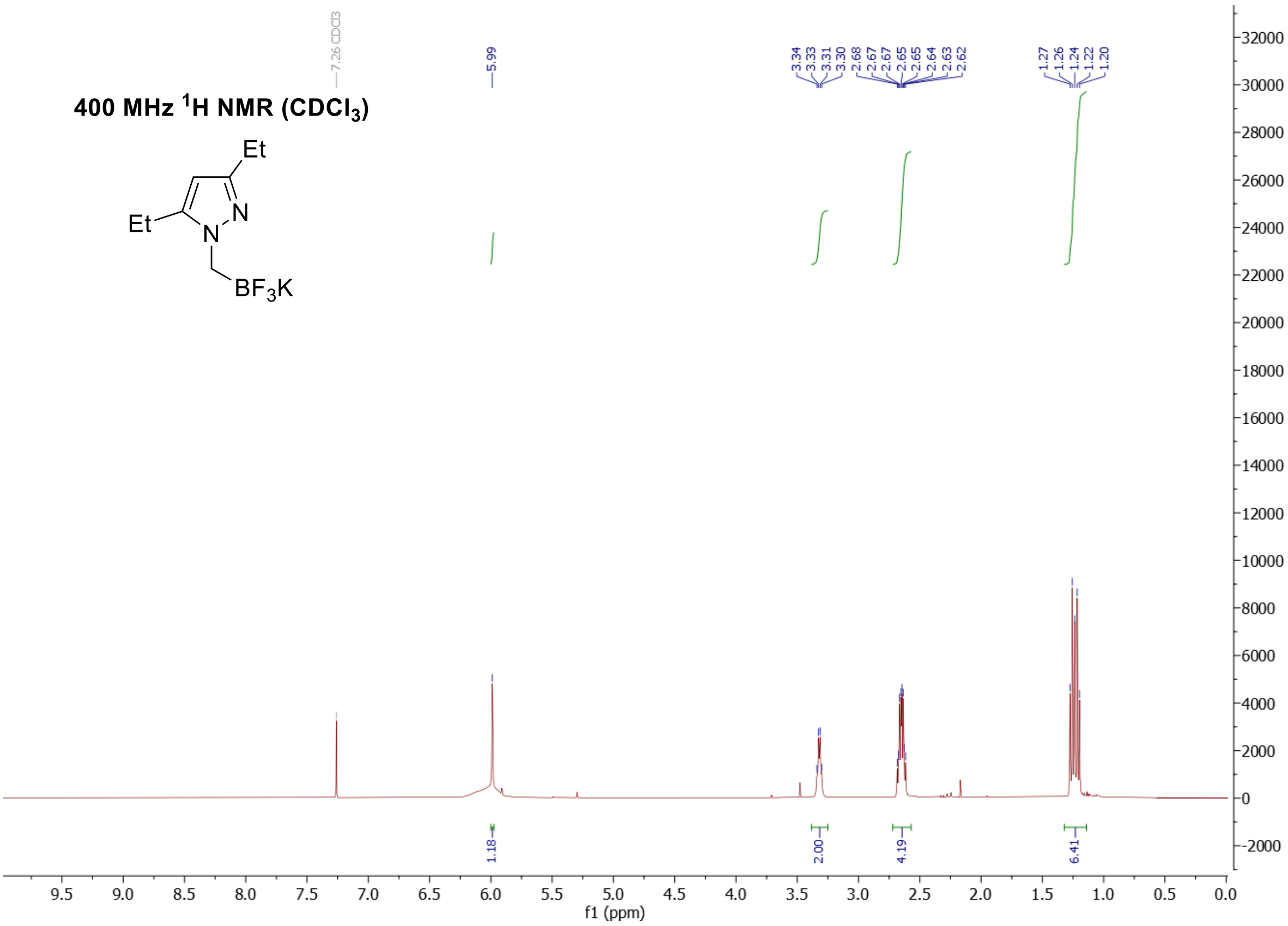
400 MHz 1H NMR (CDCl3)
Et
Et
N
N
BF3K
7.26 CDCl3
5.99
3.34
3.33
3.31
3.30
2.68
2.67
2.67
2.65
2.65
2.64
2.63
2.62
1.27
1.26
1.24
1.22
1.20
1.18
2.00
4.19
6.41
f1 (ppm)

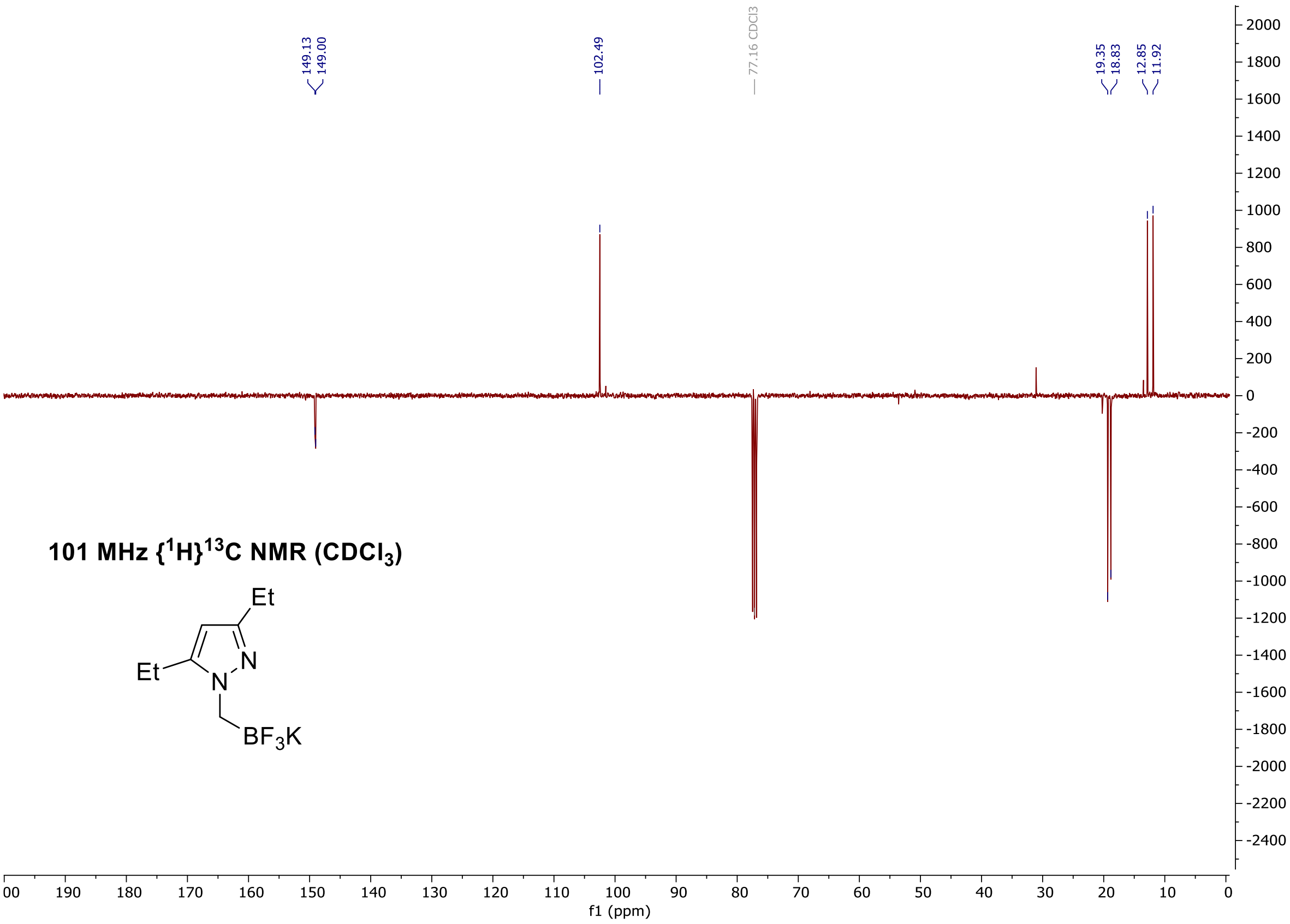
149.13
149.00
102.49
77.16 CDCl3
19.35
18.83
12.85
11.92
101 MHz {1H}13C NMR (CDCl3)
Et
Et
N
N
BF3K
f1 (ppm)

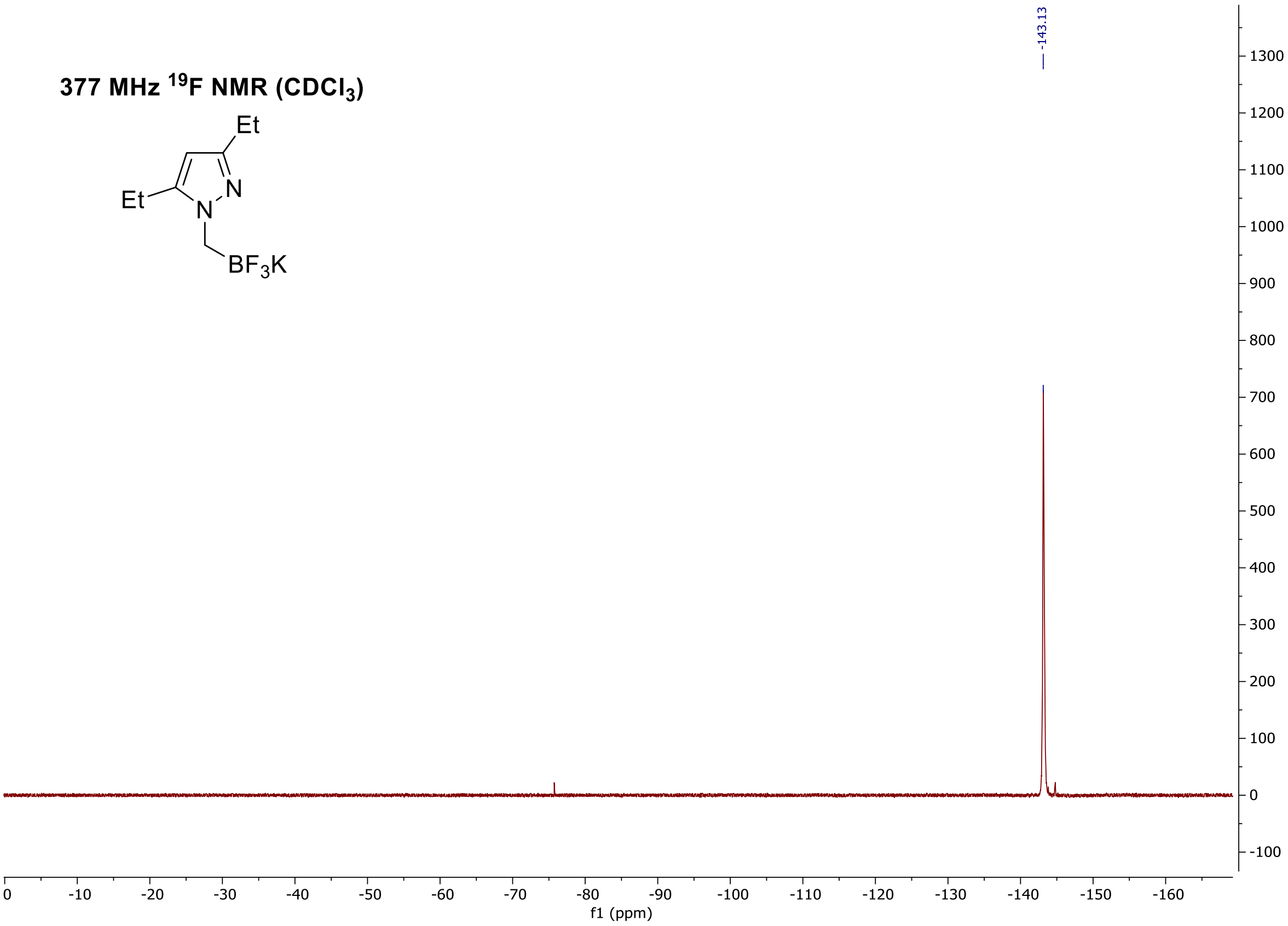
377 MHz ^{19}F NMR ($CDCl_3$)
Et
Et
N
N
BF_3K
-143.13
f1 (ppm)

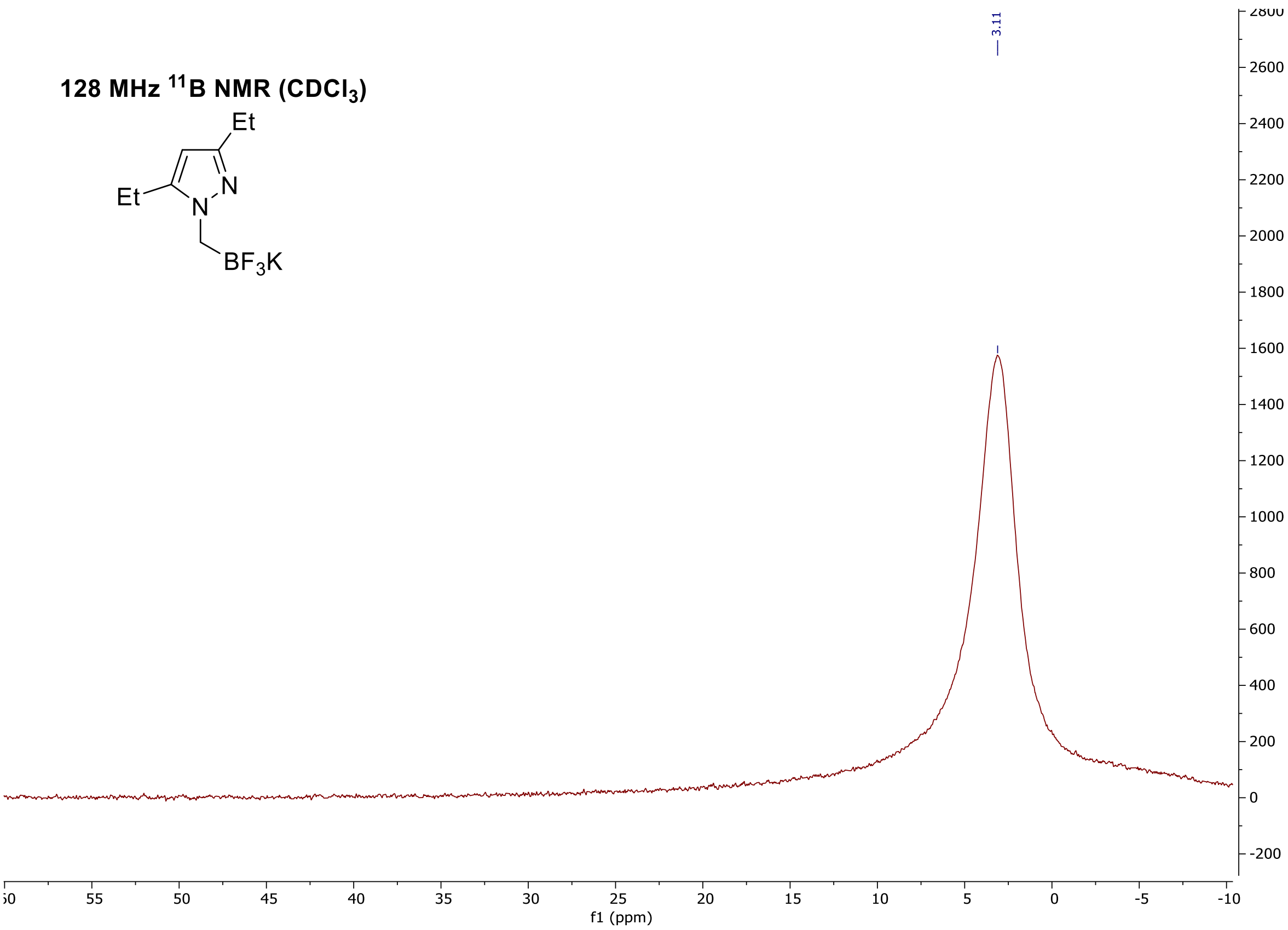
128 MHz ^{11}B NMR ($CDCl_3$)
Et
Et
N
N
BF_3K
3.11
f1 (ppm)

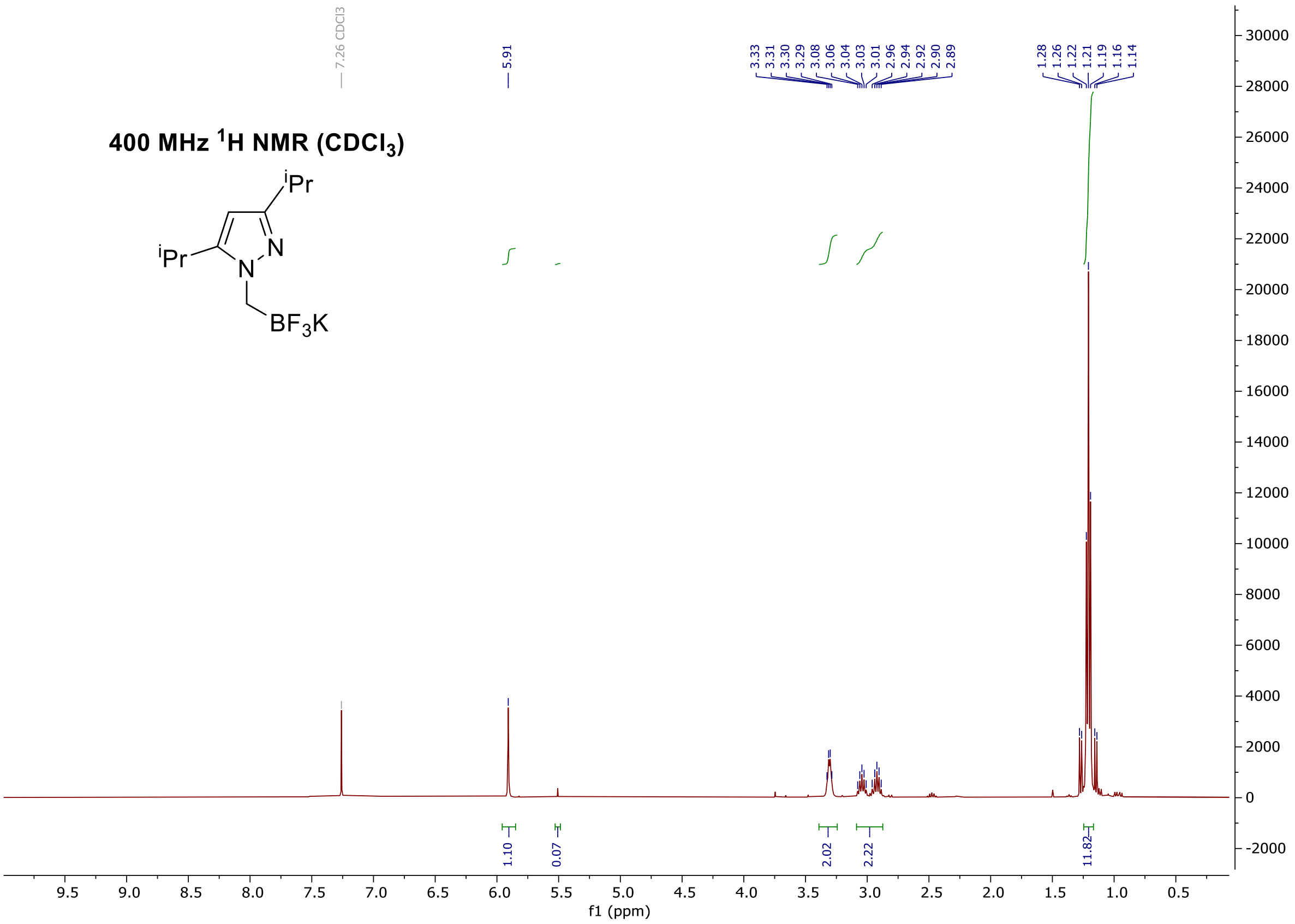
400 MHz ¹H NMR (CDCl₃)
iPr
iPr
N
N
BF₃K
7.26 CDCl3
5.91
3.33 3.31 3.30 3.29 3.08 3.06 3.04 3.03 3.01 2.96 2.94 2.92 2.90 2.89
1.28 1.26 1.22 1.21 1.19 1.16 1.14
1.10
0.07
2.02
2.22
11.82
f1 (ppm)

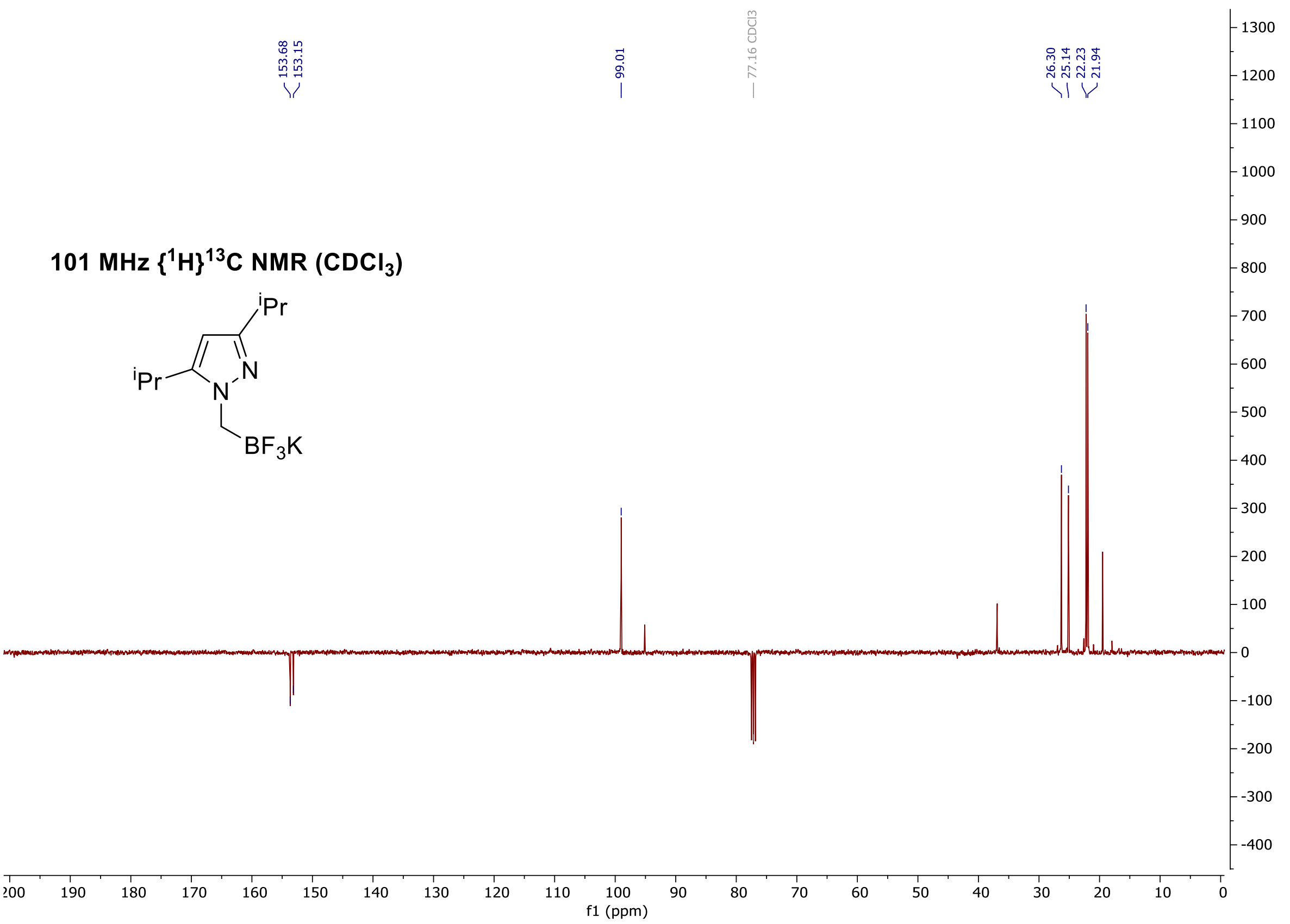
101 MHz {¹H}¹³C NMR (CDCl₃)
iPr
iPr
N
N
BF₃K
153.68 153.15
99.01
77.16 CDCl3
26.30 25.14 22.23 21.94
f1 (ppm)

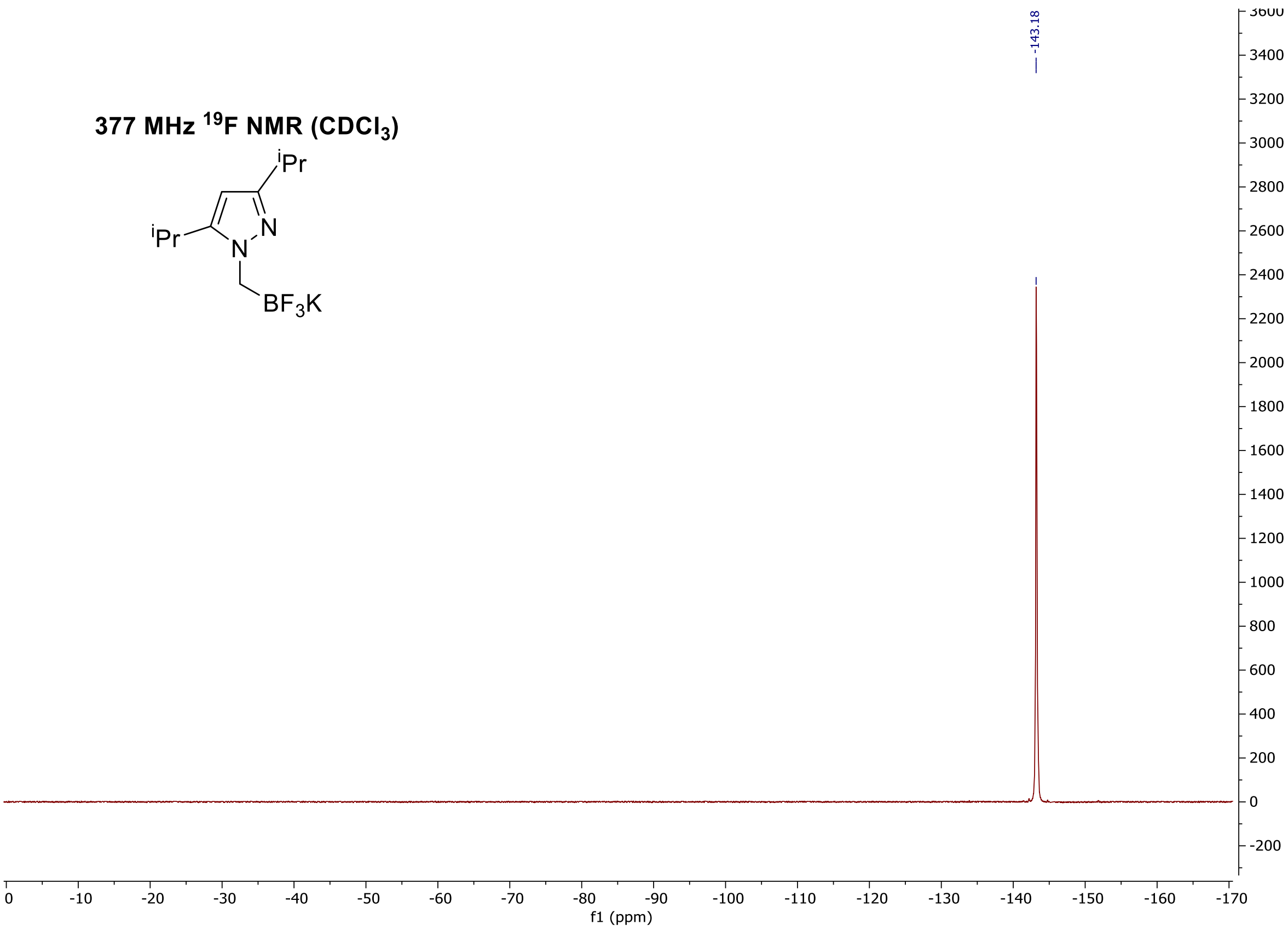
377 MHz 19F NMR (CDCl3)
iPr
iPr
N
N
BF3K
-143.18
f1 (ppm)

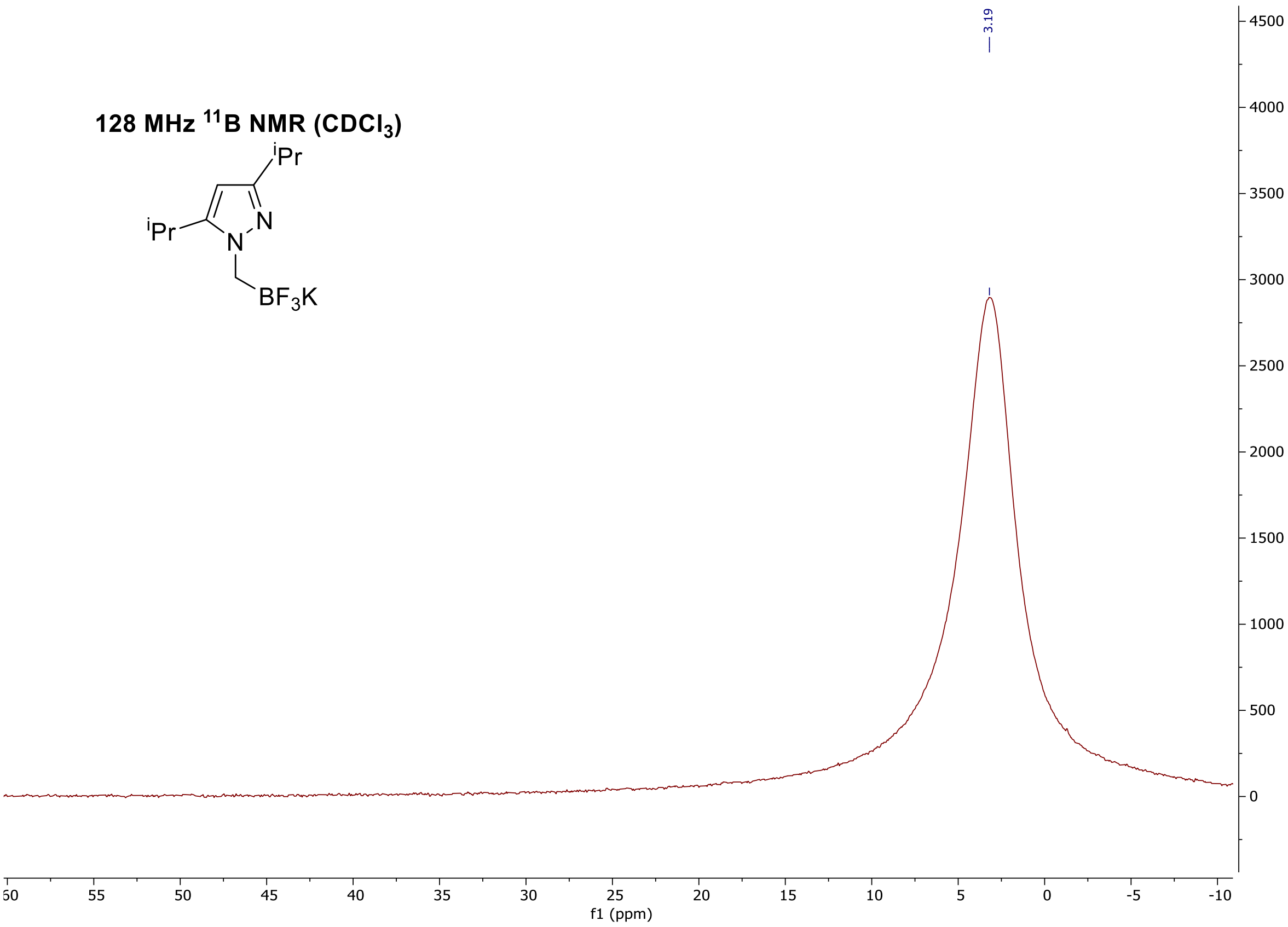
128 MHz 11B NMR (CDCl3)
iPr
iPr
N
N
BF3K
3.19
f1 (ppm)

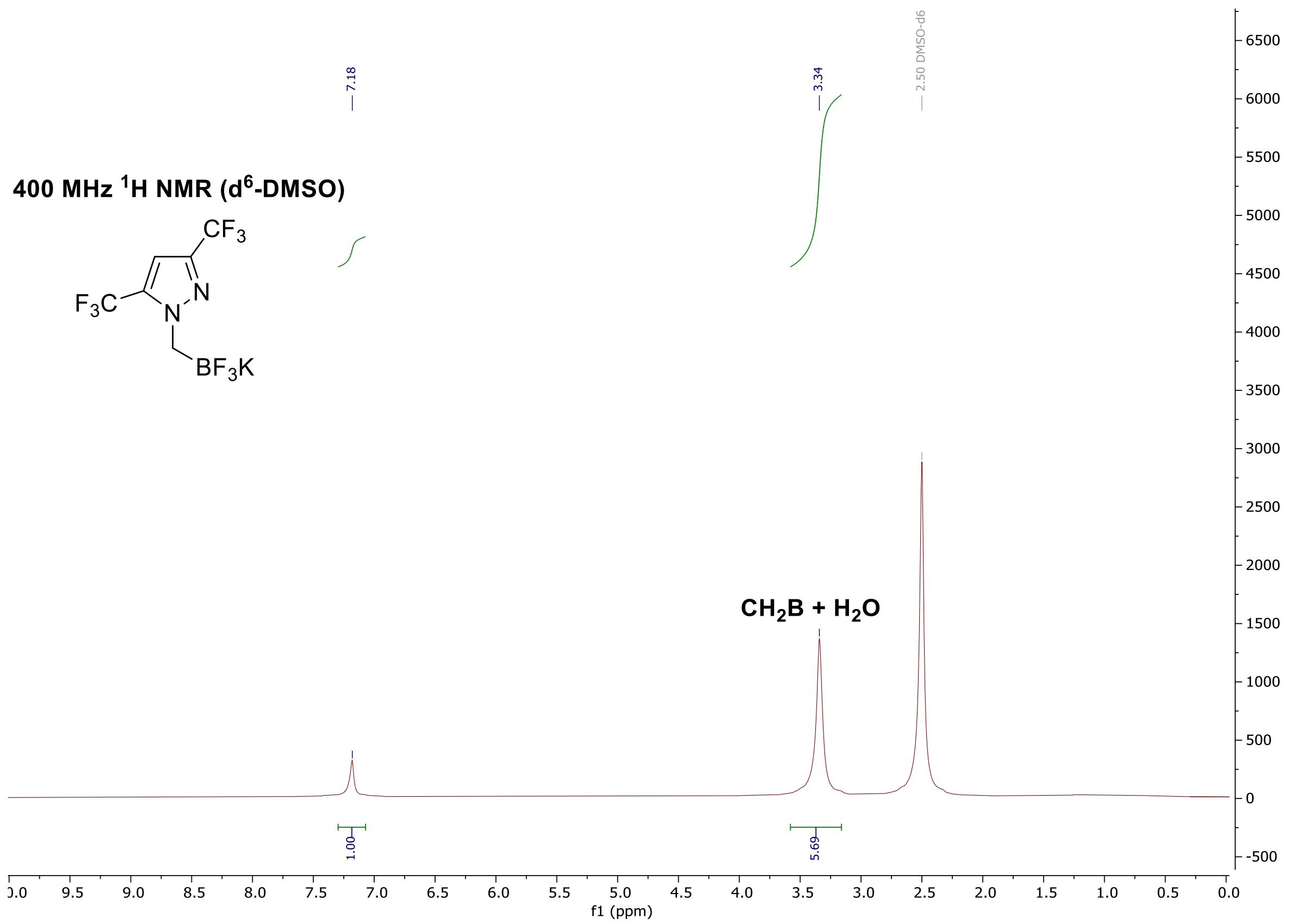
400 MHz ¹H NMR (d⁶-DMSO)
CF3
F3C
N
N
BF3K
7.18
3.34
2.50 DMSO-d6
CH2B + H2O
1.00
5.69
f1 (ppm)

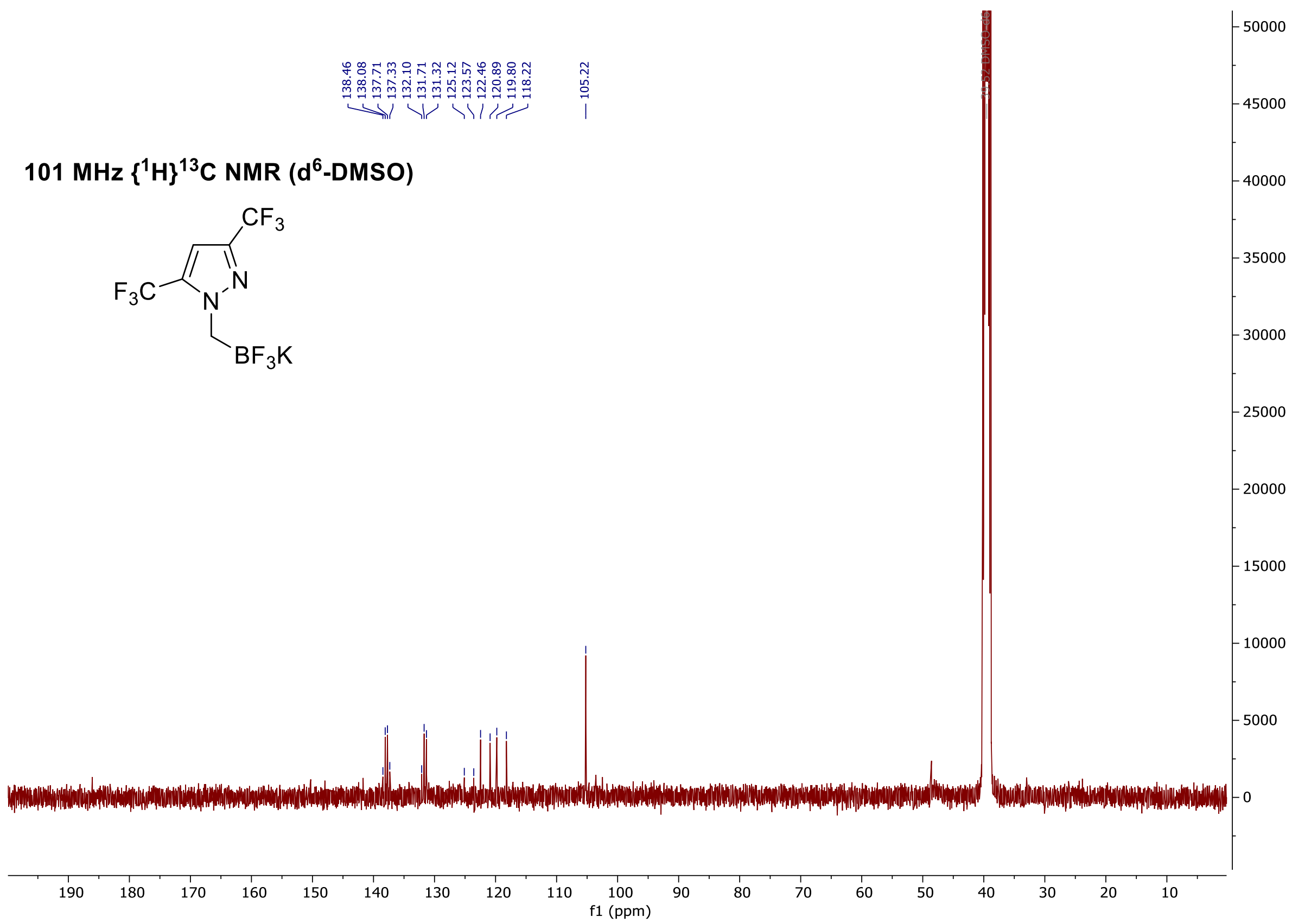
101 MHz {¹H}¹³C NMR (d⁶-DMSO)
CF3
F3C
N
N
BF3K
138.46
138.08
137.71
137.33
132.10
131.71
131.32
125.12
123.57
122.46
120.89
119.80
118.22
105.22
f1 (ppm)

377 MHz ^{19}F NMR (d^{6}-DMSO)

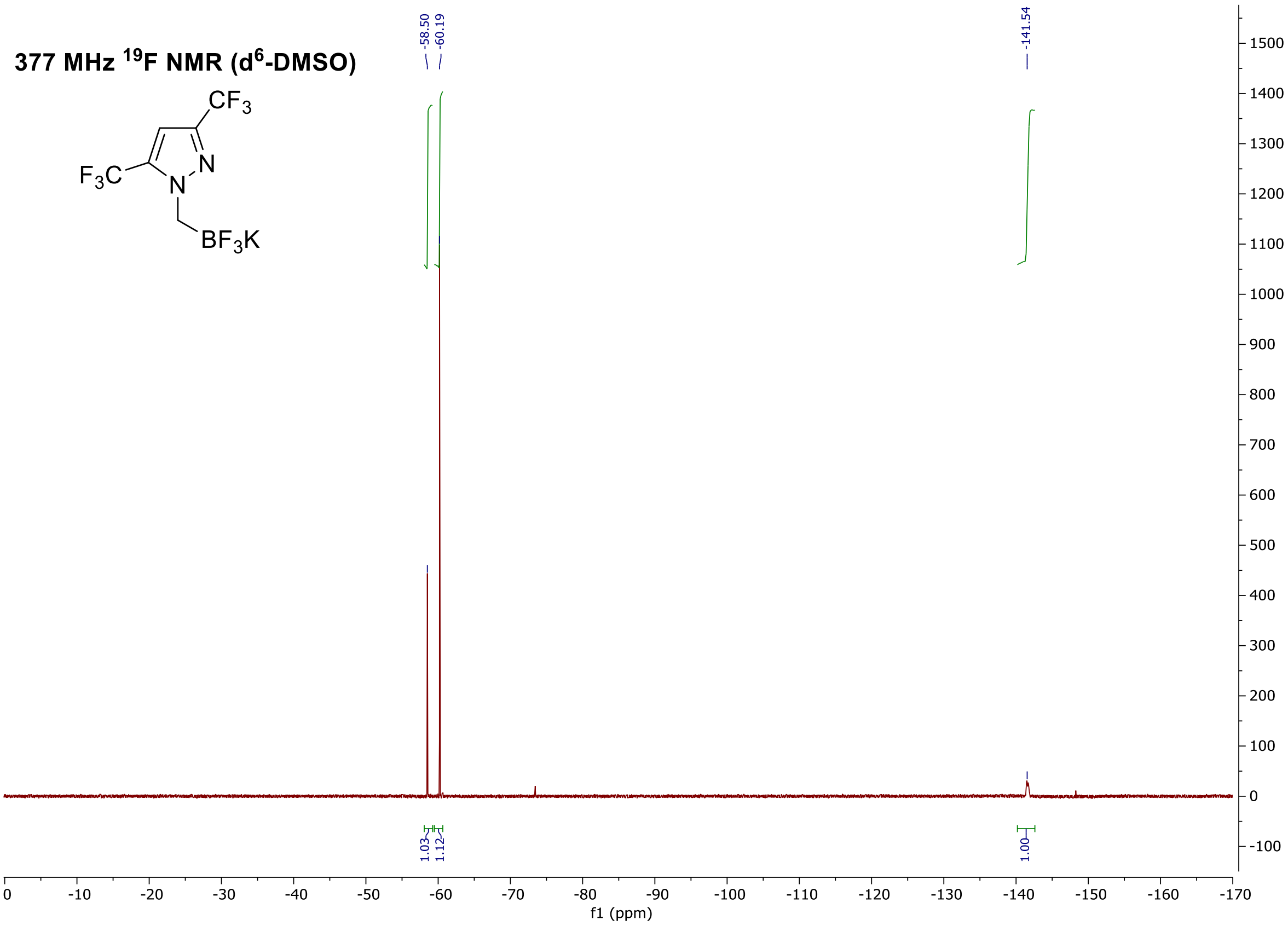

128 MHz ^{11}B NMR (d^{6}-DMSO)

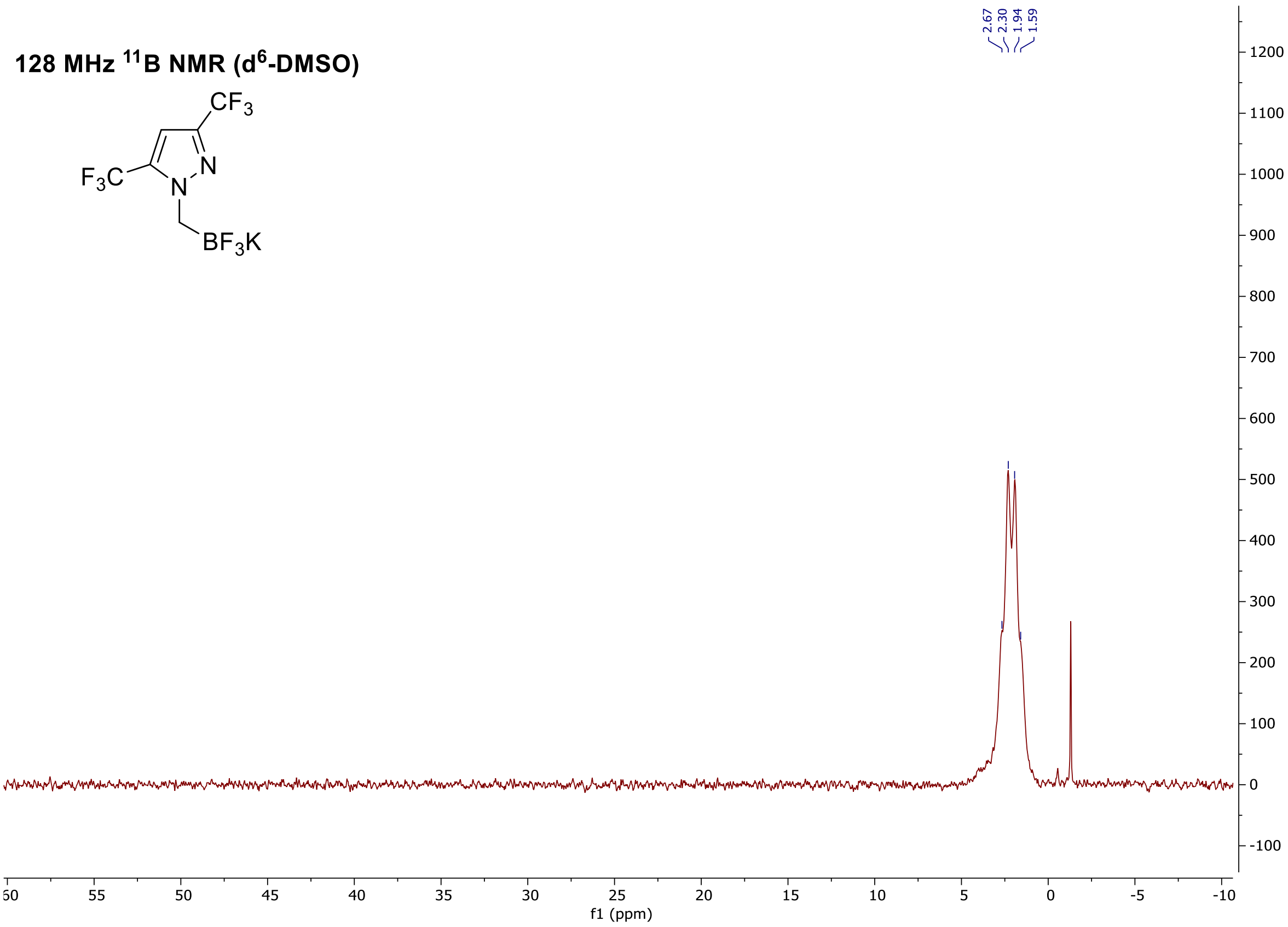

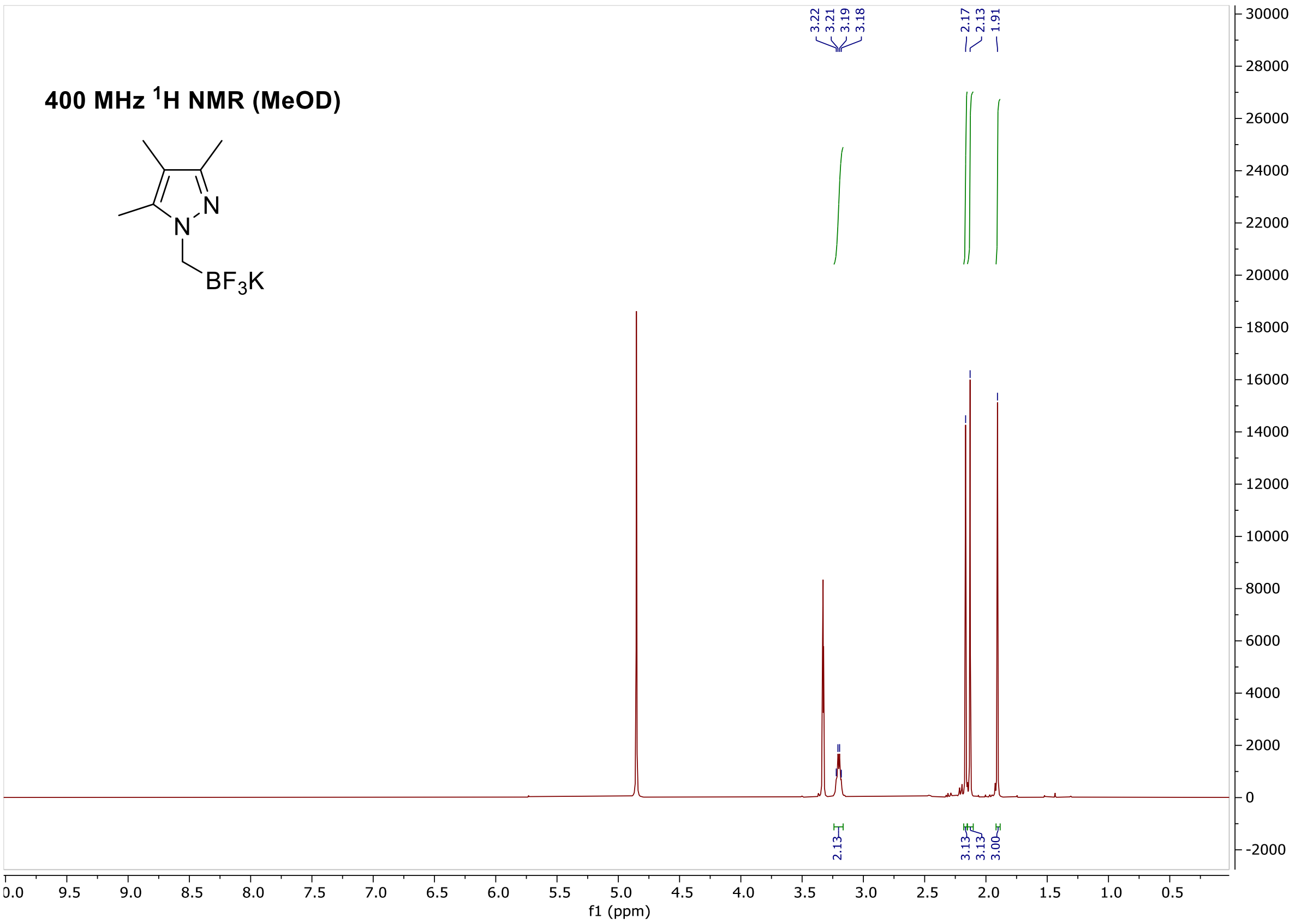
400 MHz 1H NMR (MeOD)
BF3K
N
N
3.22
3.21
3.19
3.18
2.17
2.13
1.91
2.13
3.13
3.13
3.00
f1 (ppm)

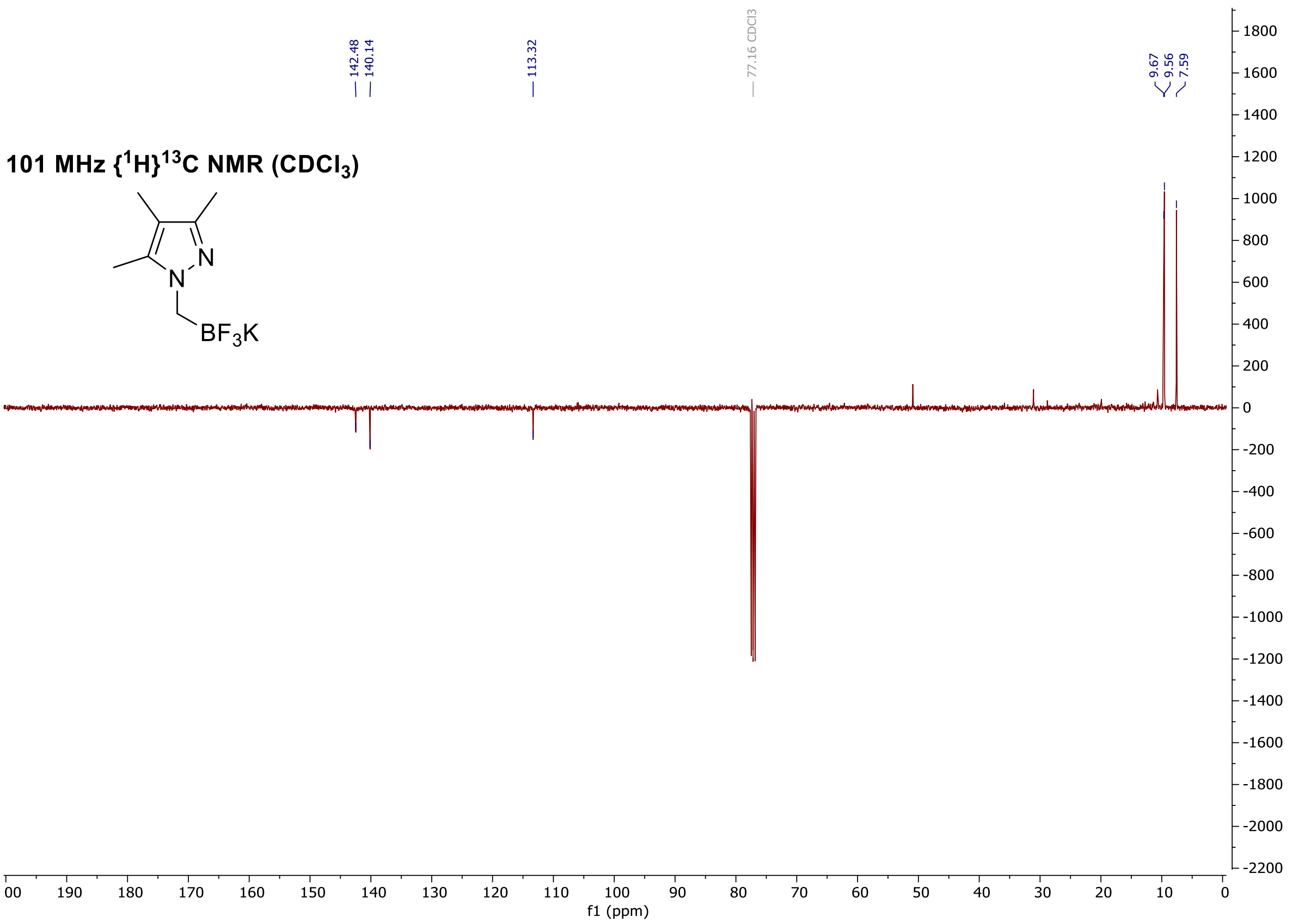
101 MHz {1H}13C NMR (CDCl3)
BF3K
N
N
142.48
140.14
113.32
77.16 CDCl3
9.67
9.56
7.59
f1 (ppm)

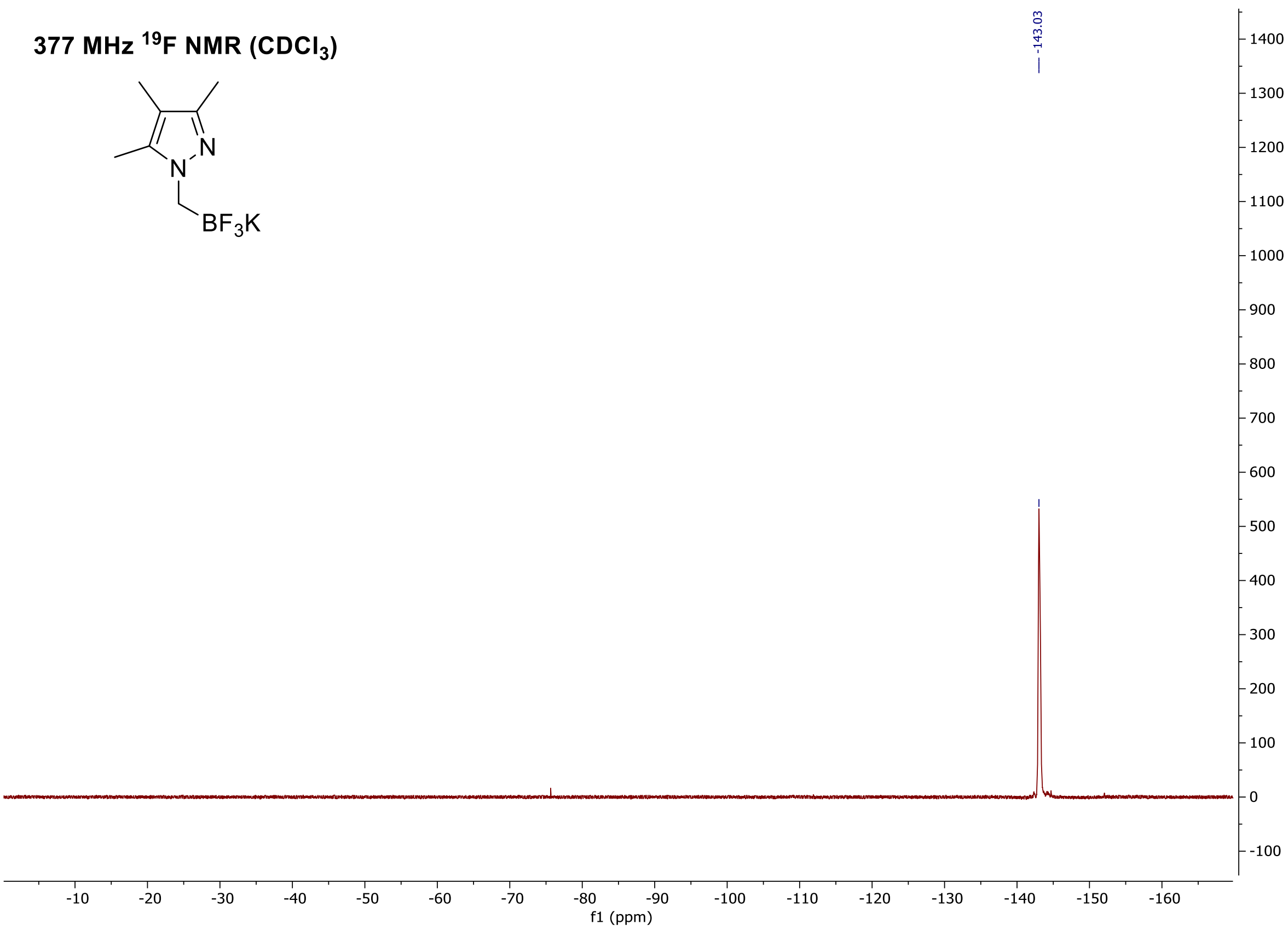
377 MHz ^{19}F NMR ($CDCl_3$)
N
N
BF_3K
-143.03
f1 (ppm)

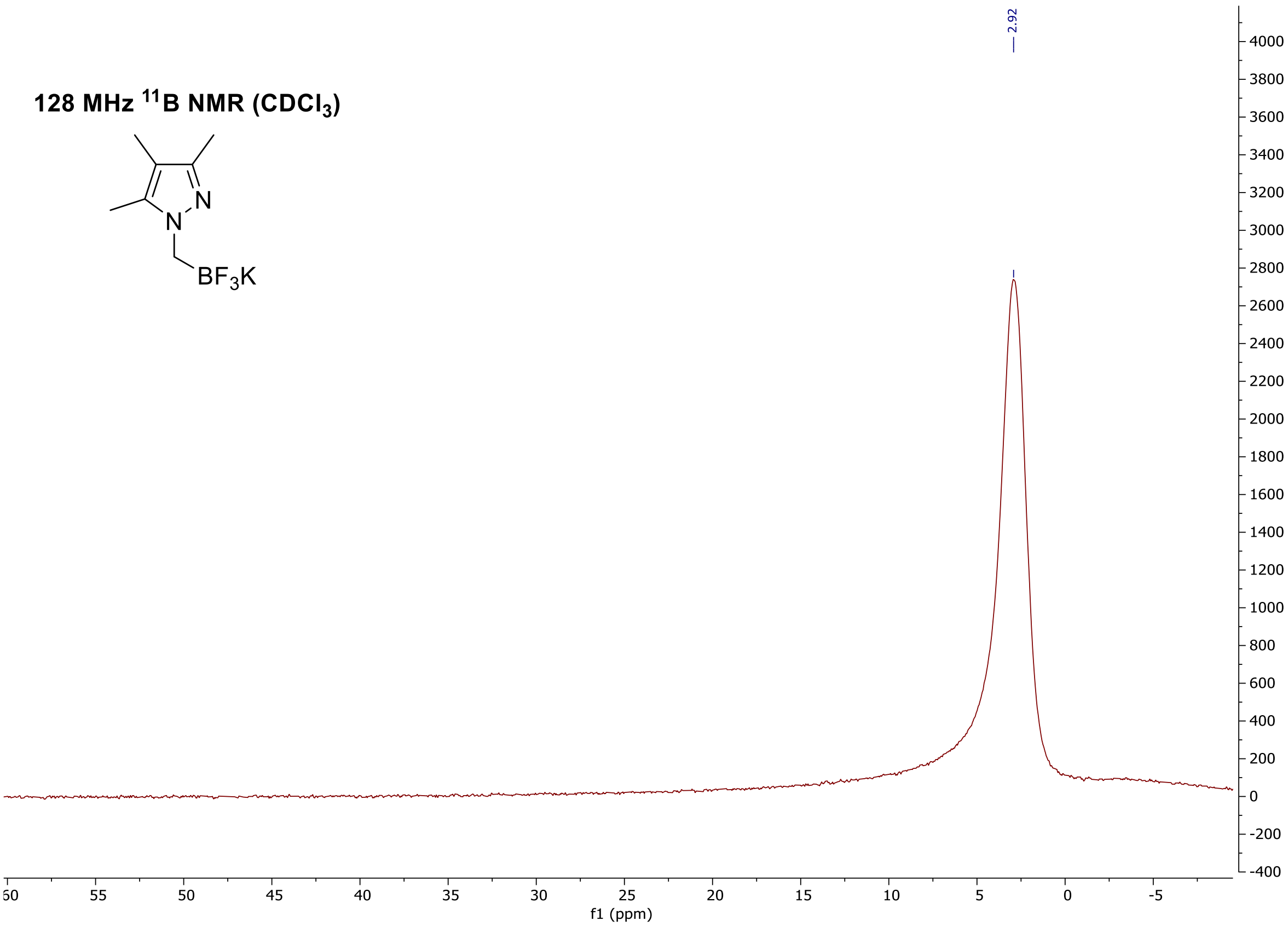
128 MHz ^{11}B NMR ($CDCl_3$)
N
N
BF_3K
2.92
f1 (ppm)

400 MHz ^{1}H NMR ($CDCl_3$)

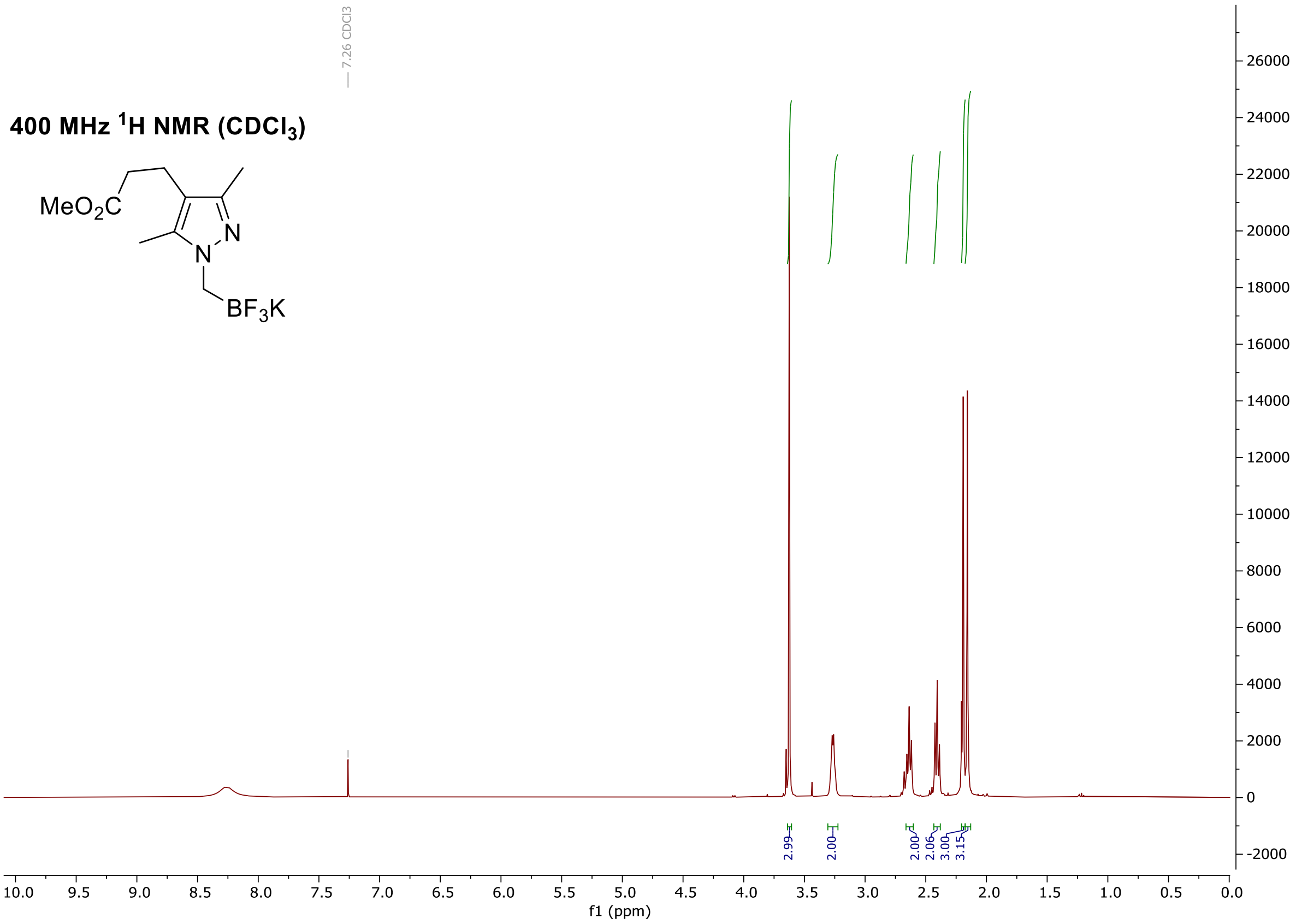

101 MHz {^{1}H}^{13}C NMR ($CDCl_3$)

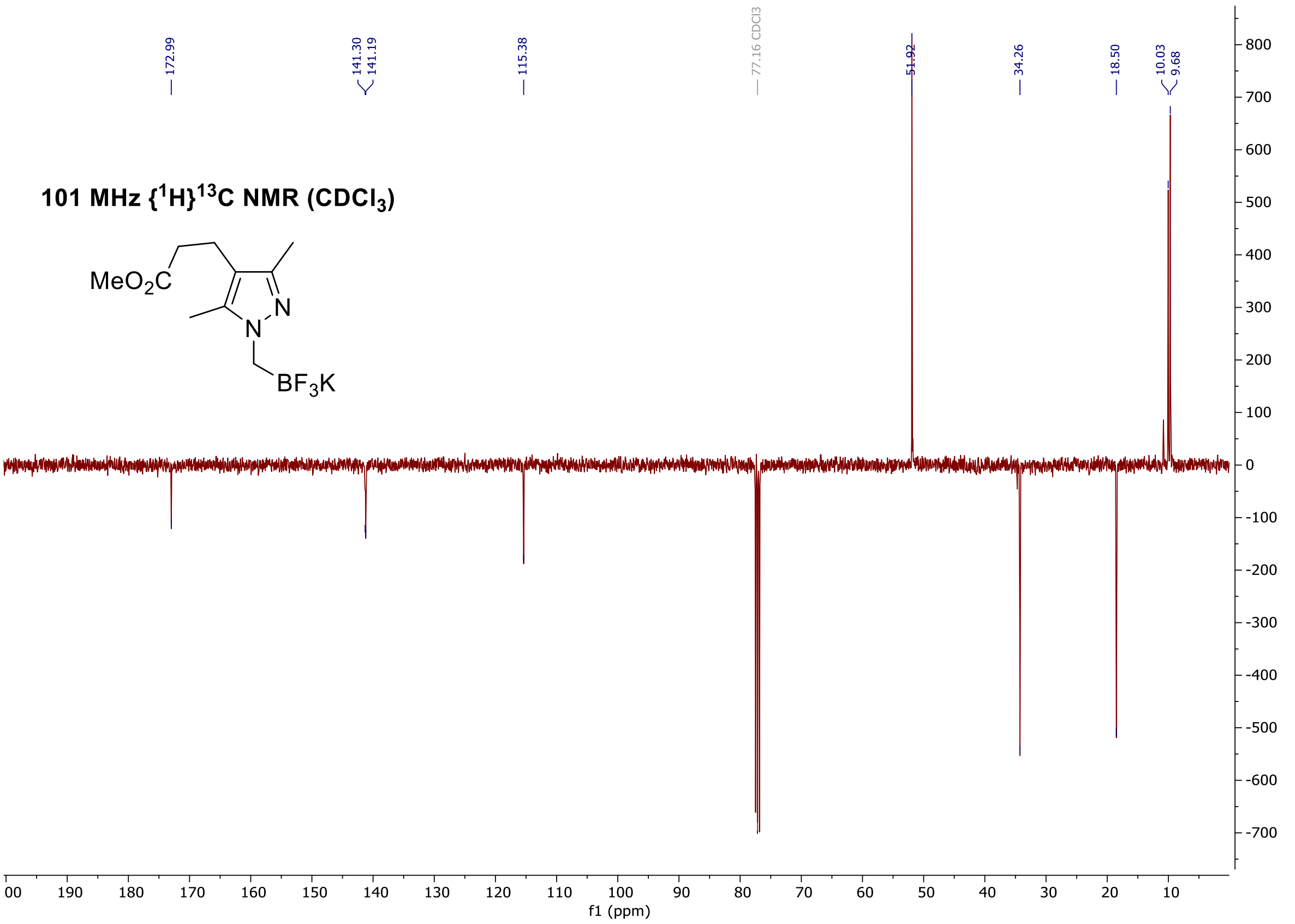

377 MHz ^{19}F NMR ($CDCl_3$)

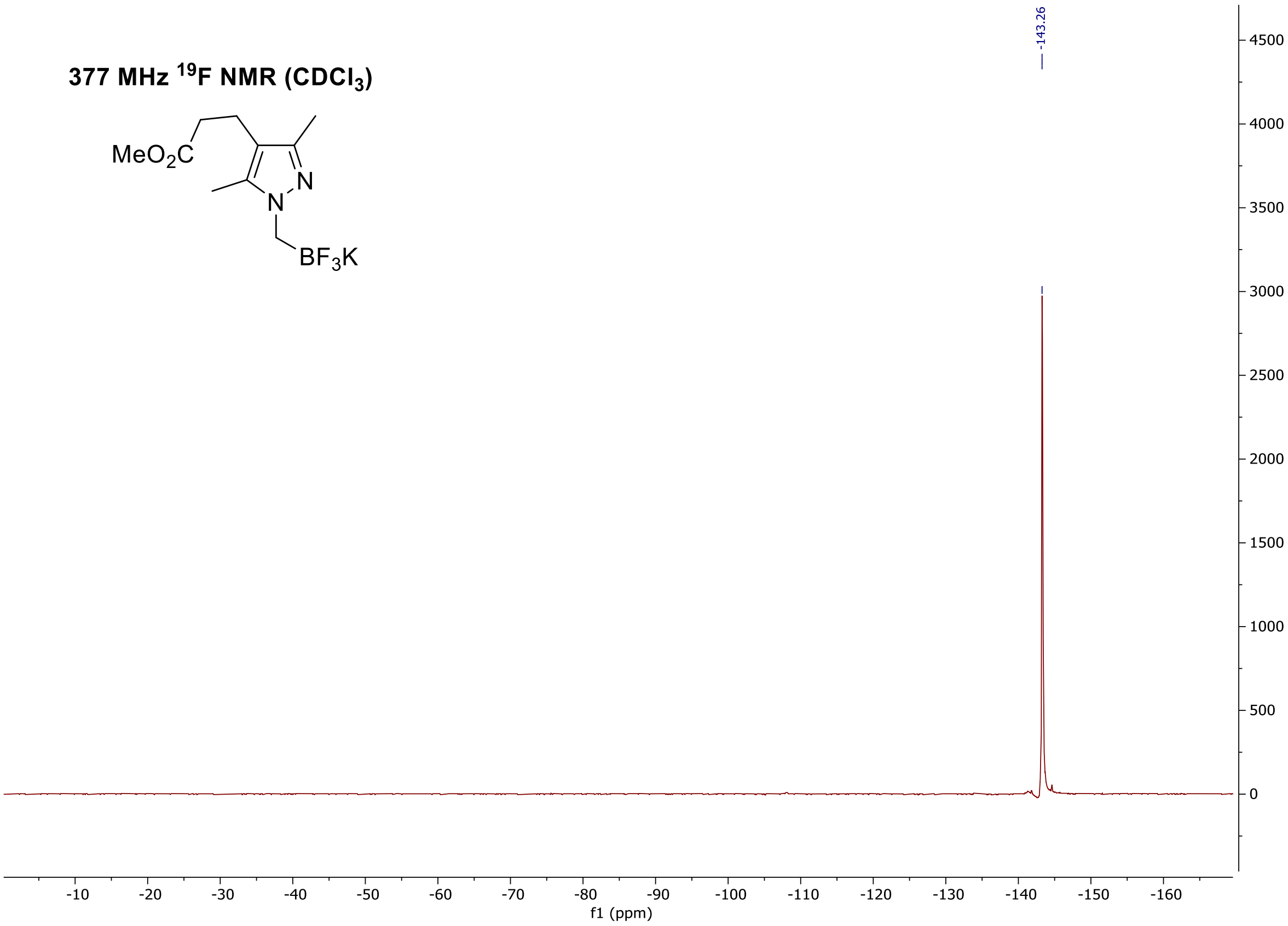

128 MHz ^{11}B NMR ($CDCl_3$)

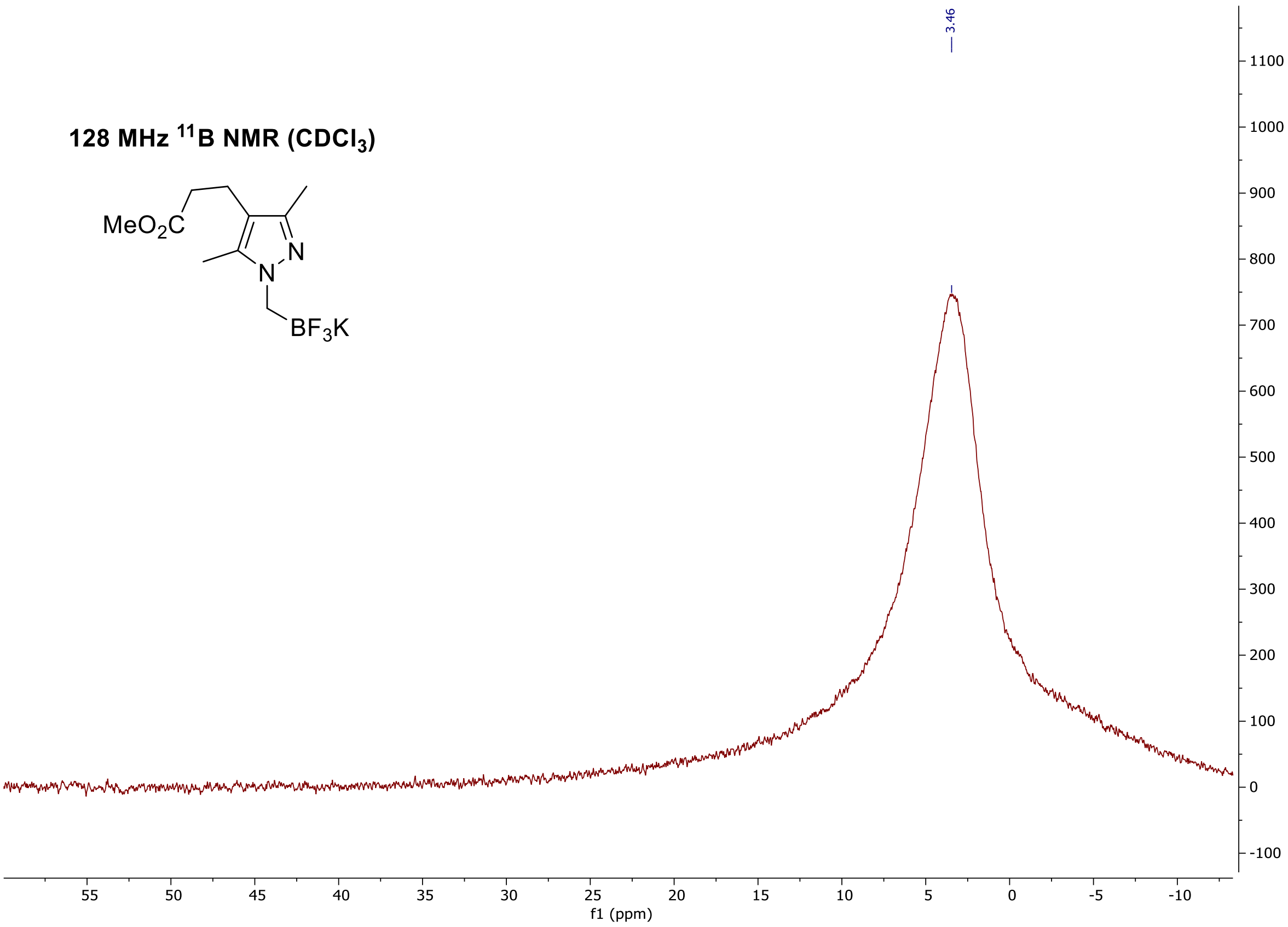

400 MHz ^{1}H NMR ($CDCl_3$)

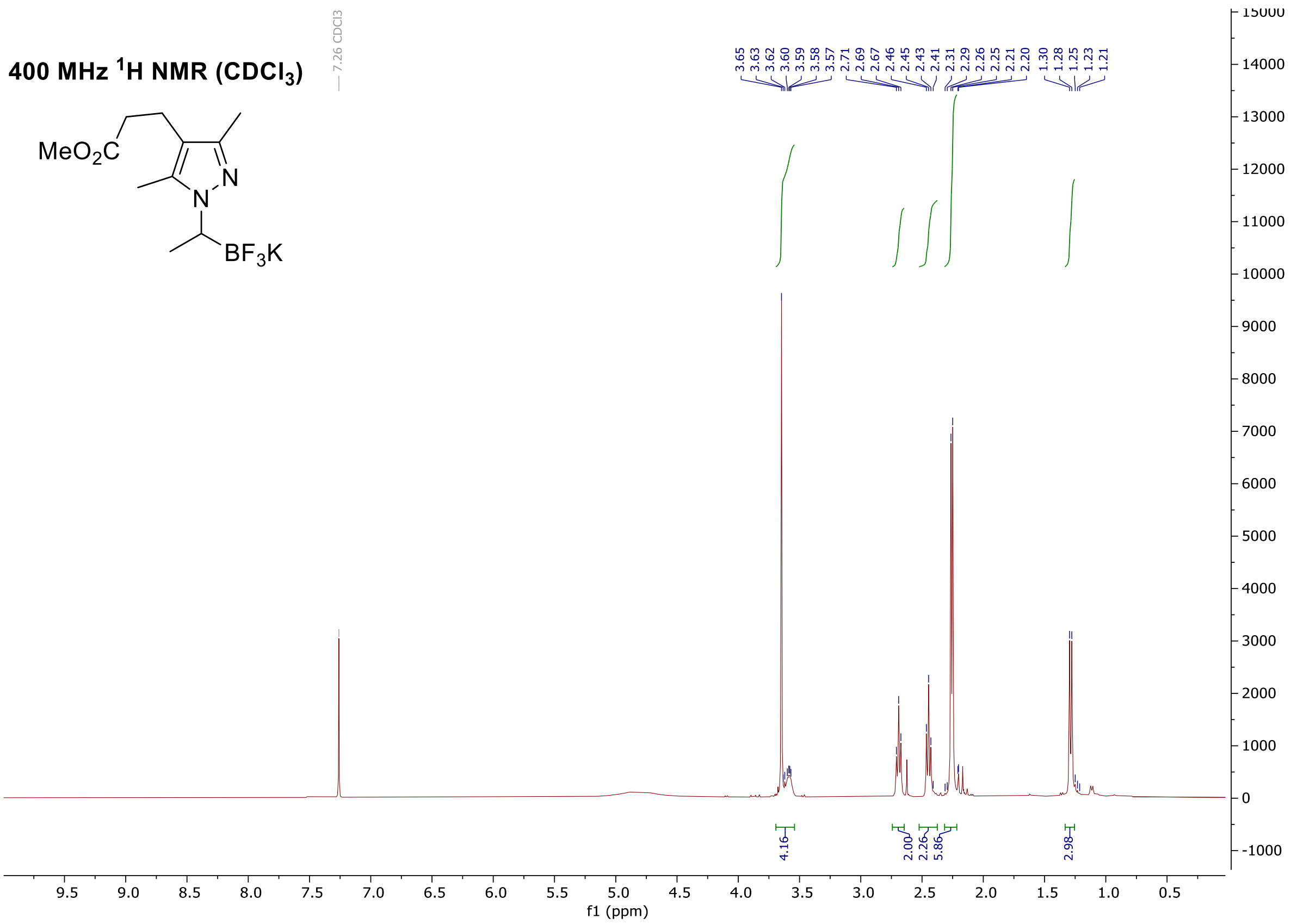

101 MHz {^{1}H}^{13}C NMR ($CDCl_3$)

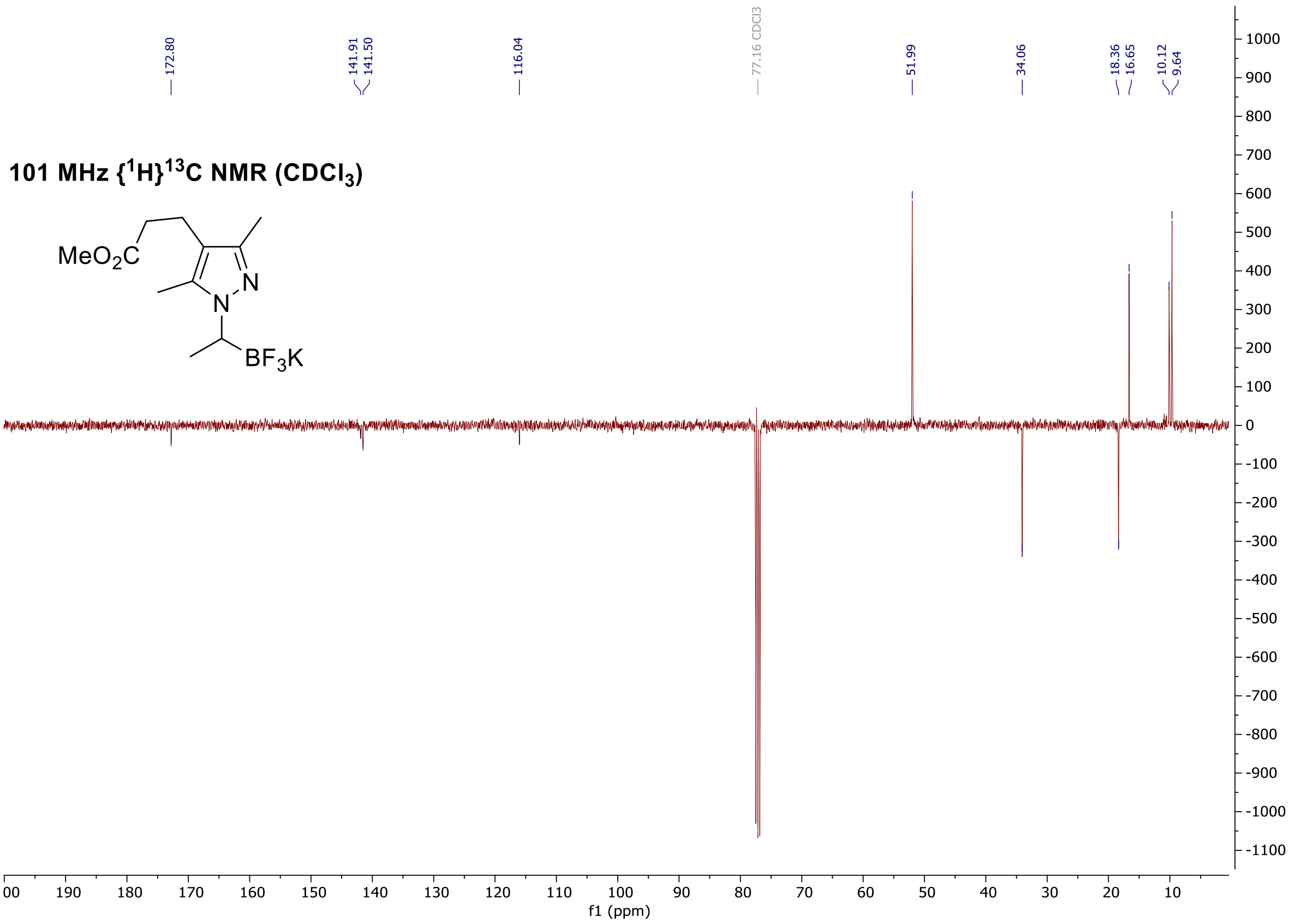

377 MHz ^{19}F NMR ($CDCl_3$)

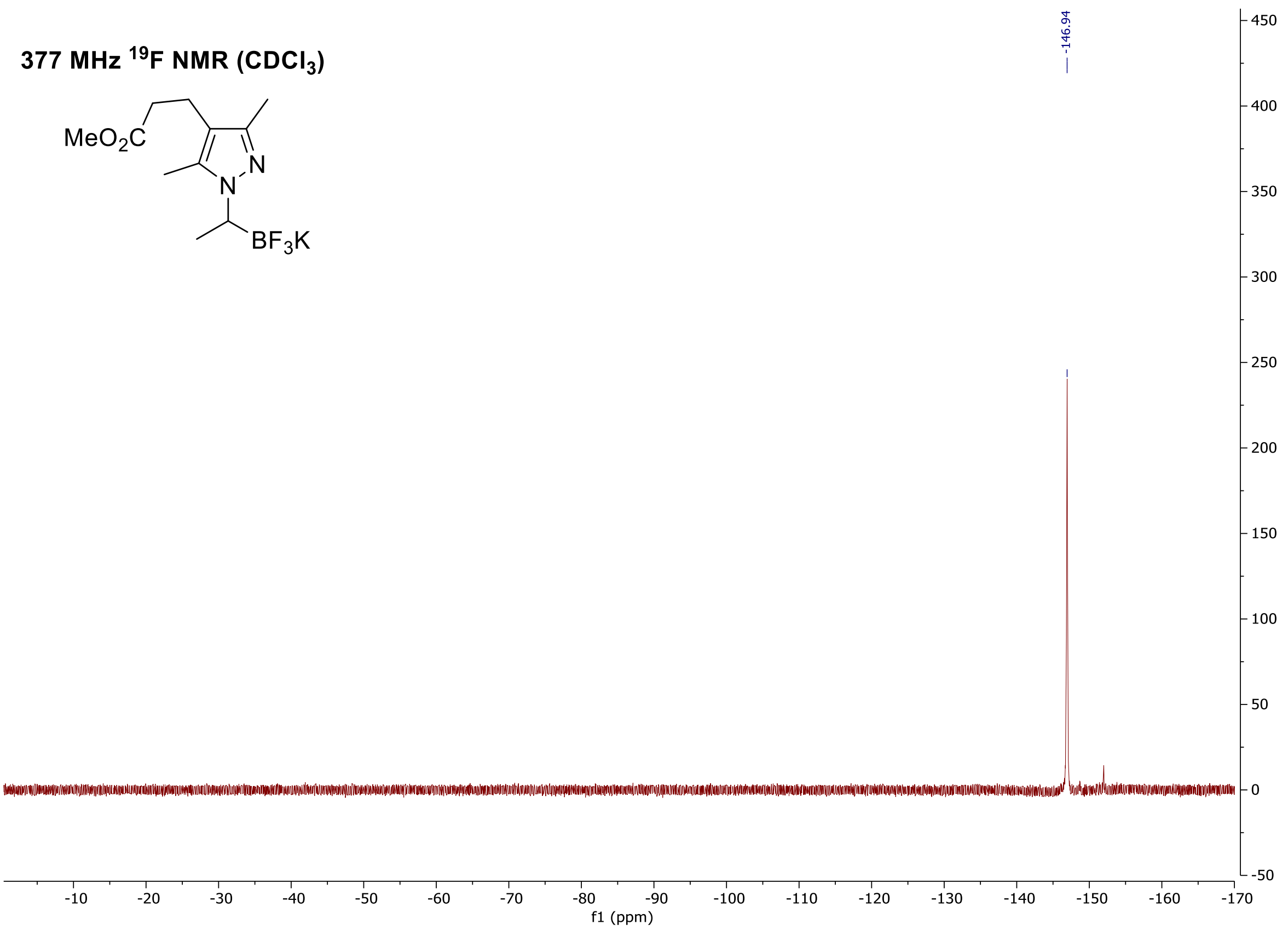

128 MHz ^{11}B NMR ($CDCl_3$)

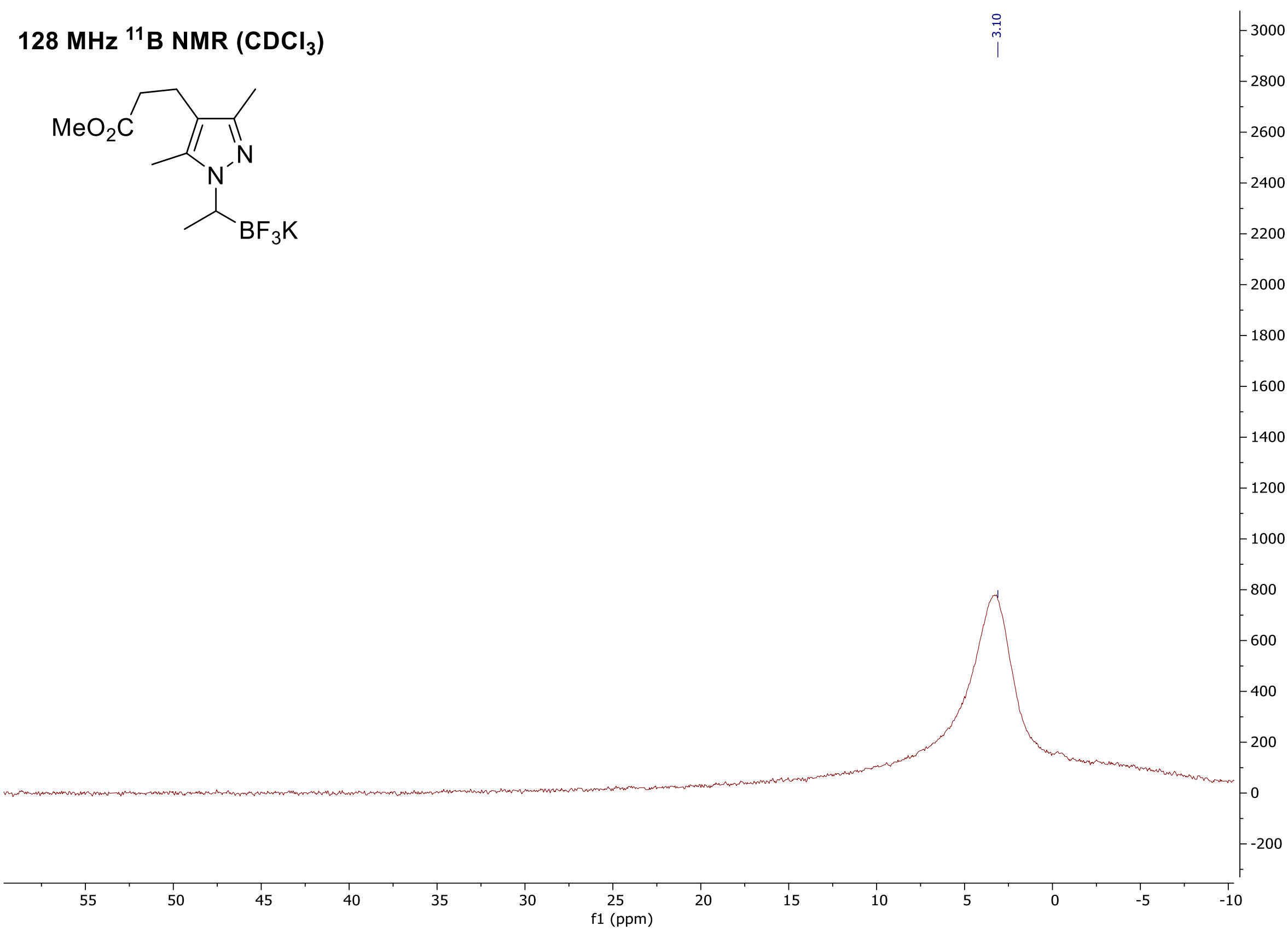

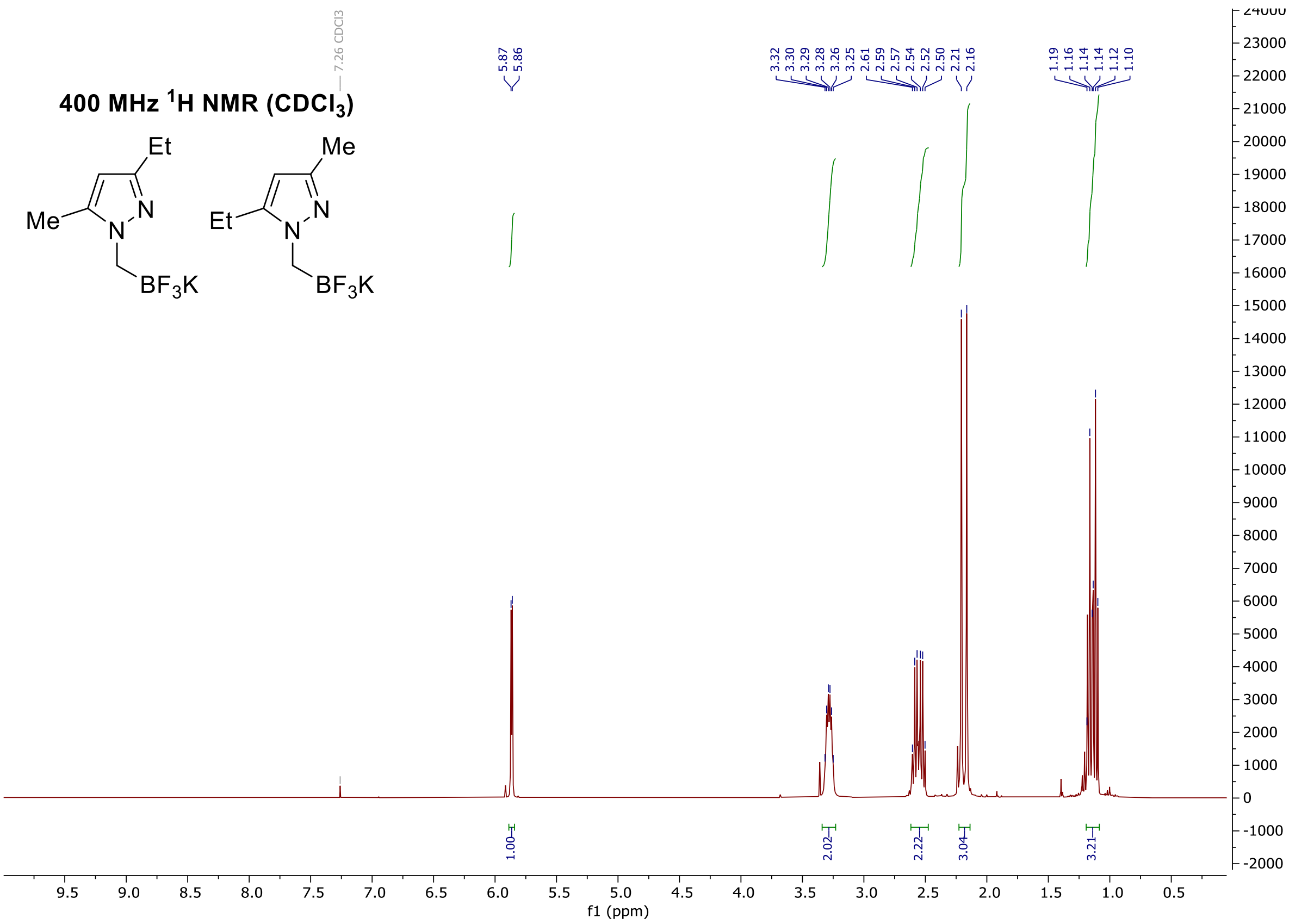
400 MHz ¹H NMR (CDCl3)
Et
Me
N
N
BF3K
7.26 CDCl3
5.87
5.86
3.32
3.30
3.29
3.28
3.26
3.25
2.61
2.59
2.57
2.54
2.52
2.50
2.21
2.16
1.19
1.16
1.14
1.14
1.12
1.10
1.00
2.02
2.22
3.04
3.21
f1 (ppm)

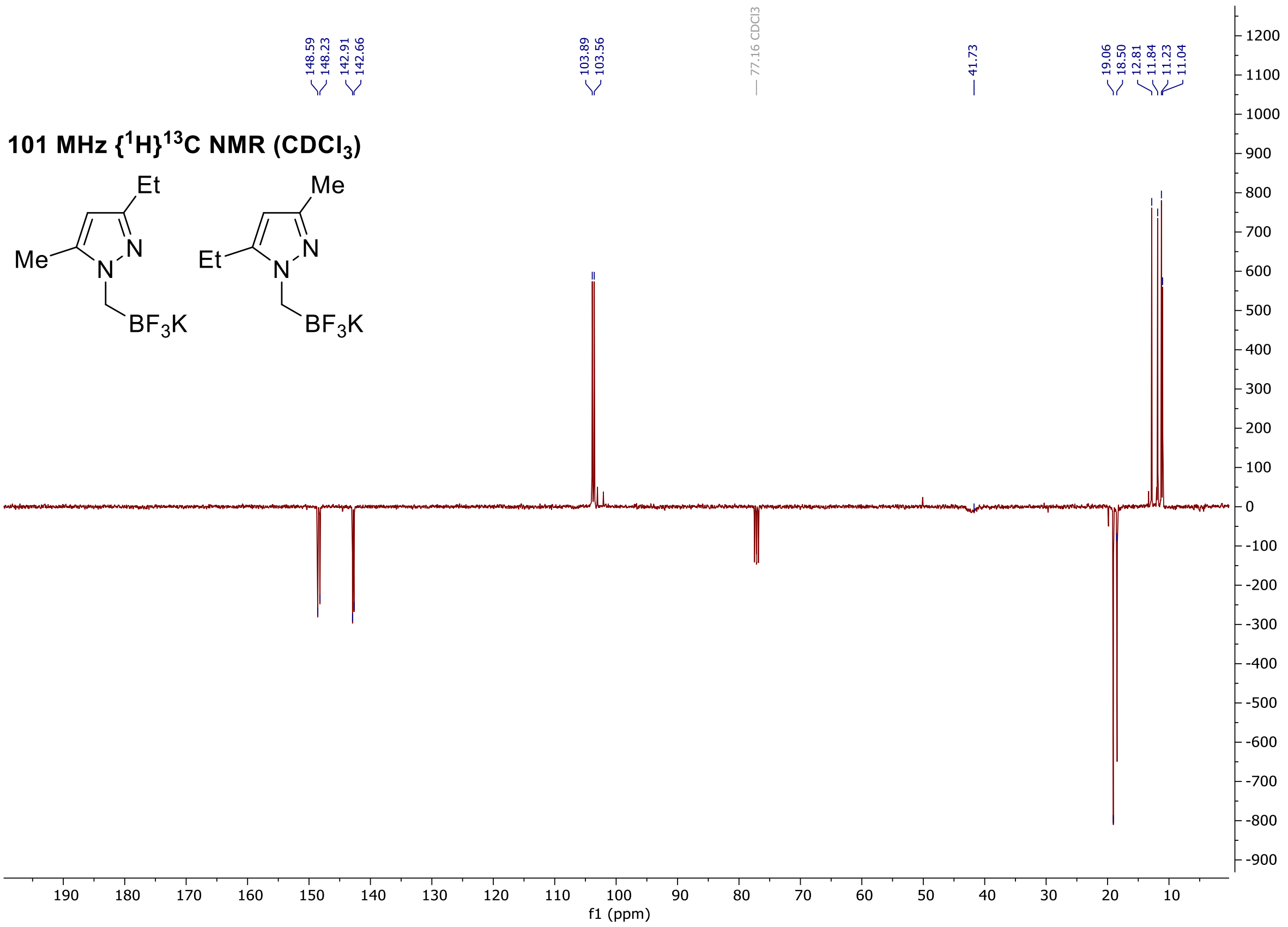
101 MHz {¹H}¹³C NMR (CDCl3)
Et
Me
N
N
BF3K
148.59
148.23
142.91
142.66
103.89
103.56
77.16 CDCl3
41.73
19.06
18.50
12.81
11.84
11.23
11.04
f1 (ppm)

377 MHz ^{19}F NMR ($CDCl_3$)

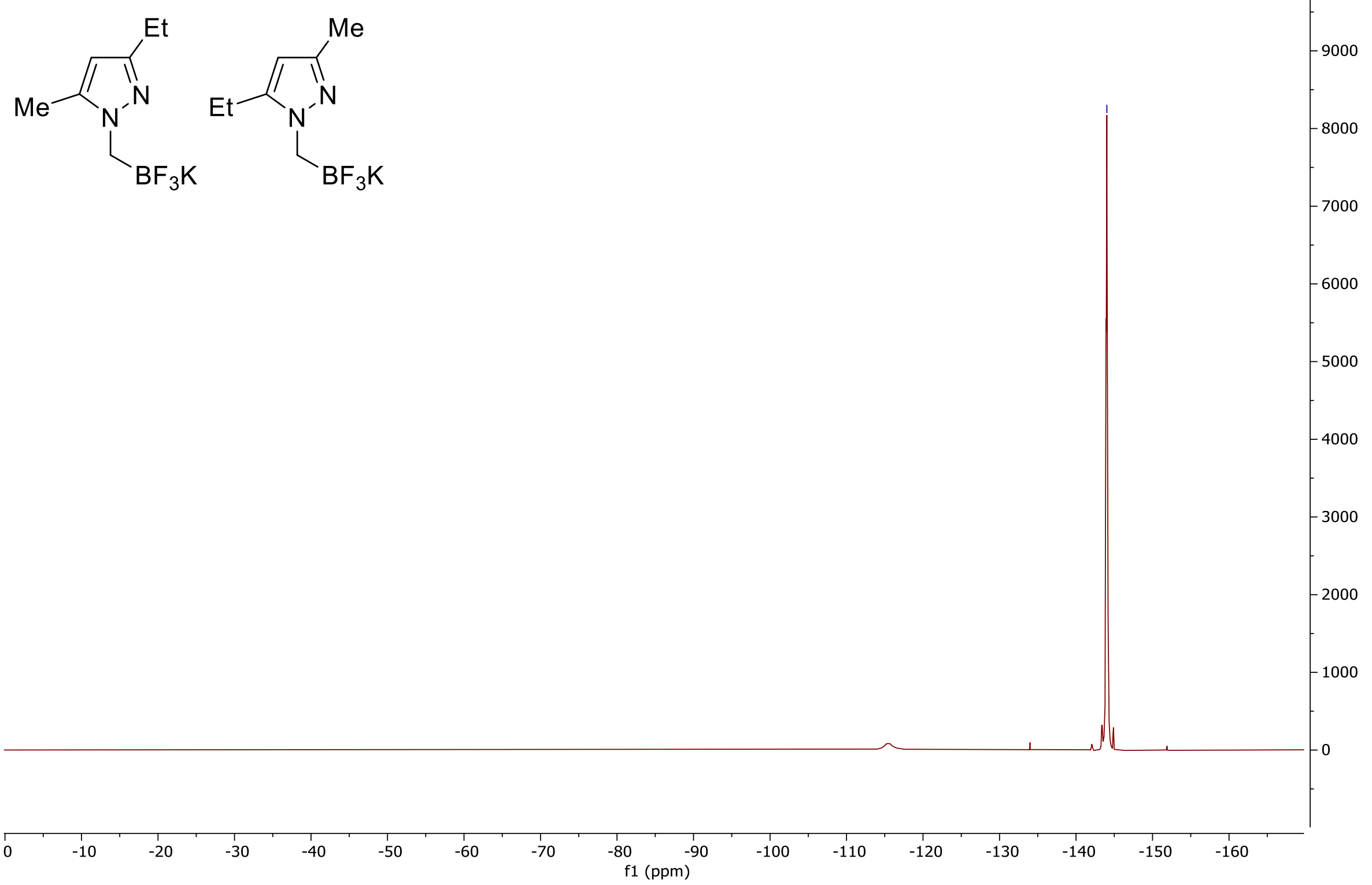

128 MHz ^{11}B NMR ($CDCl_3$)

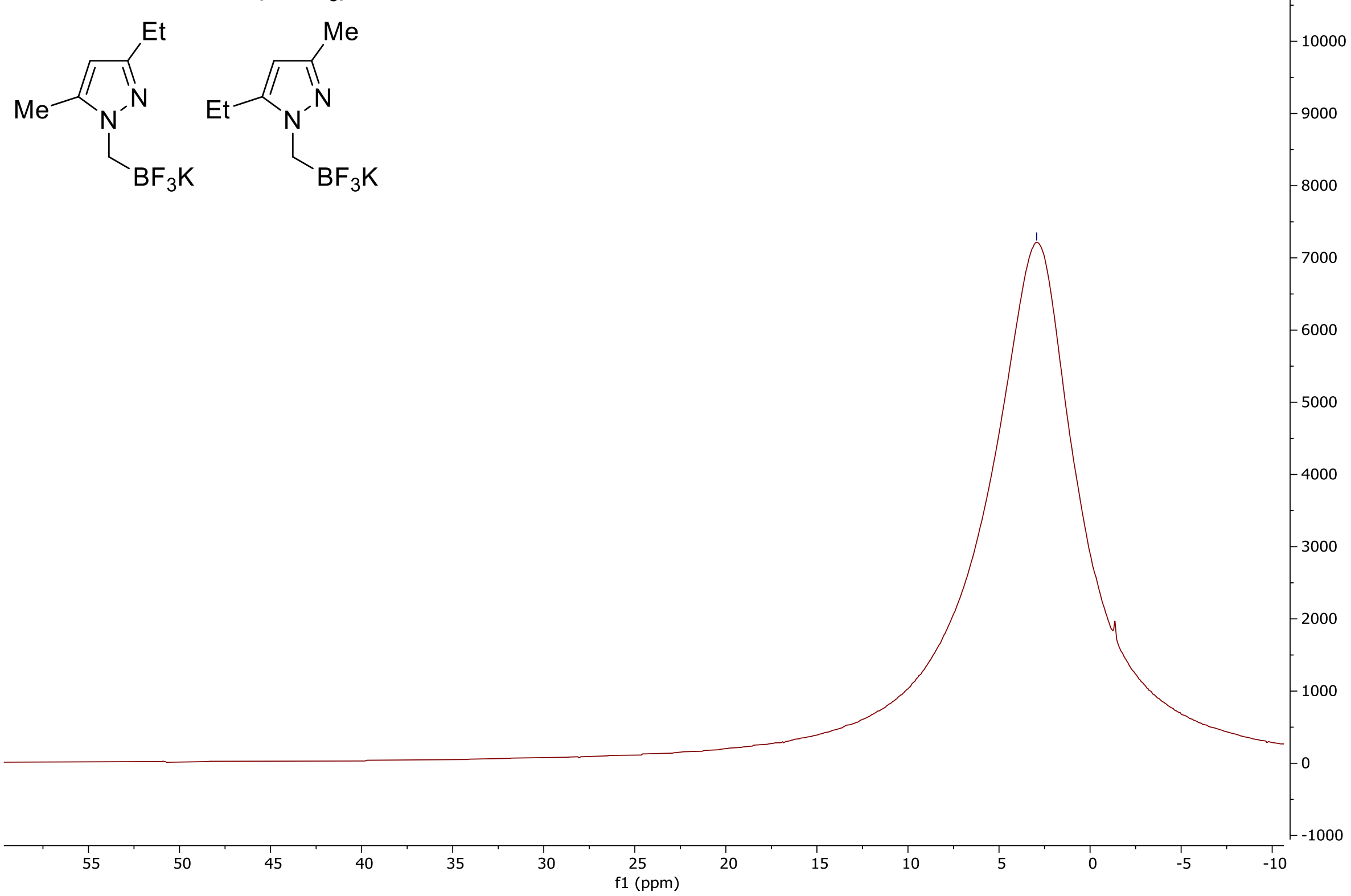

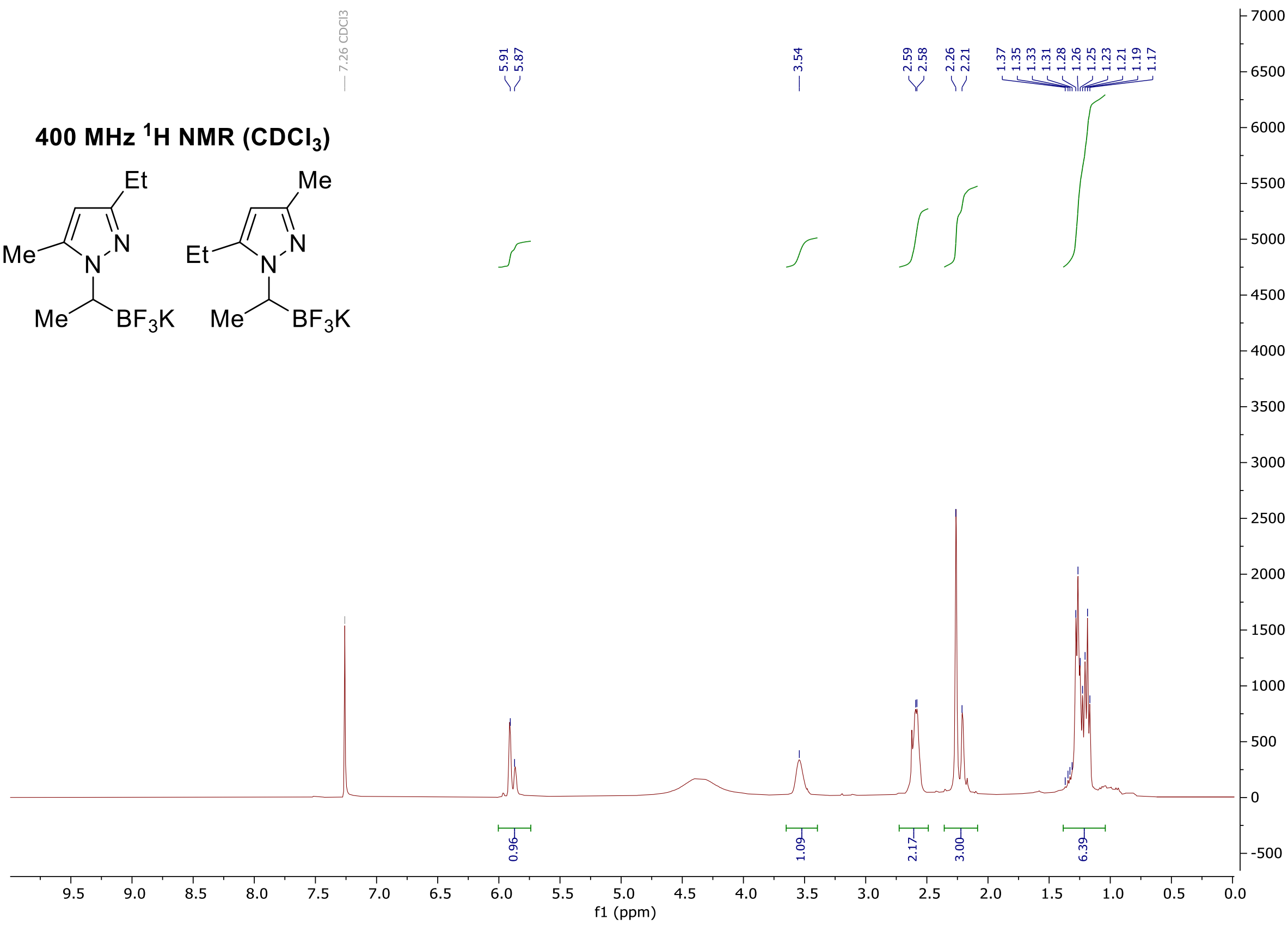
400 MHz 1H NMR (CDCl3)
7.26 CDCl3
5.91
5.87
3.54
2.59
2.58
2.26
2.21
1.37
1.35
1.33
1.31
1.28
1.26
1.25
1.23
1.21
1.19
1.17
0.96
1.09
2.17
3.00
6.39
f1 (ppm)

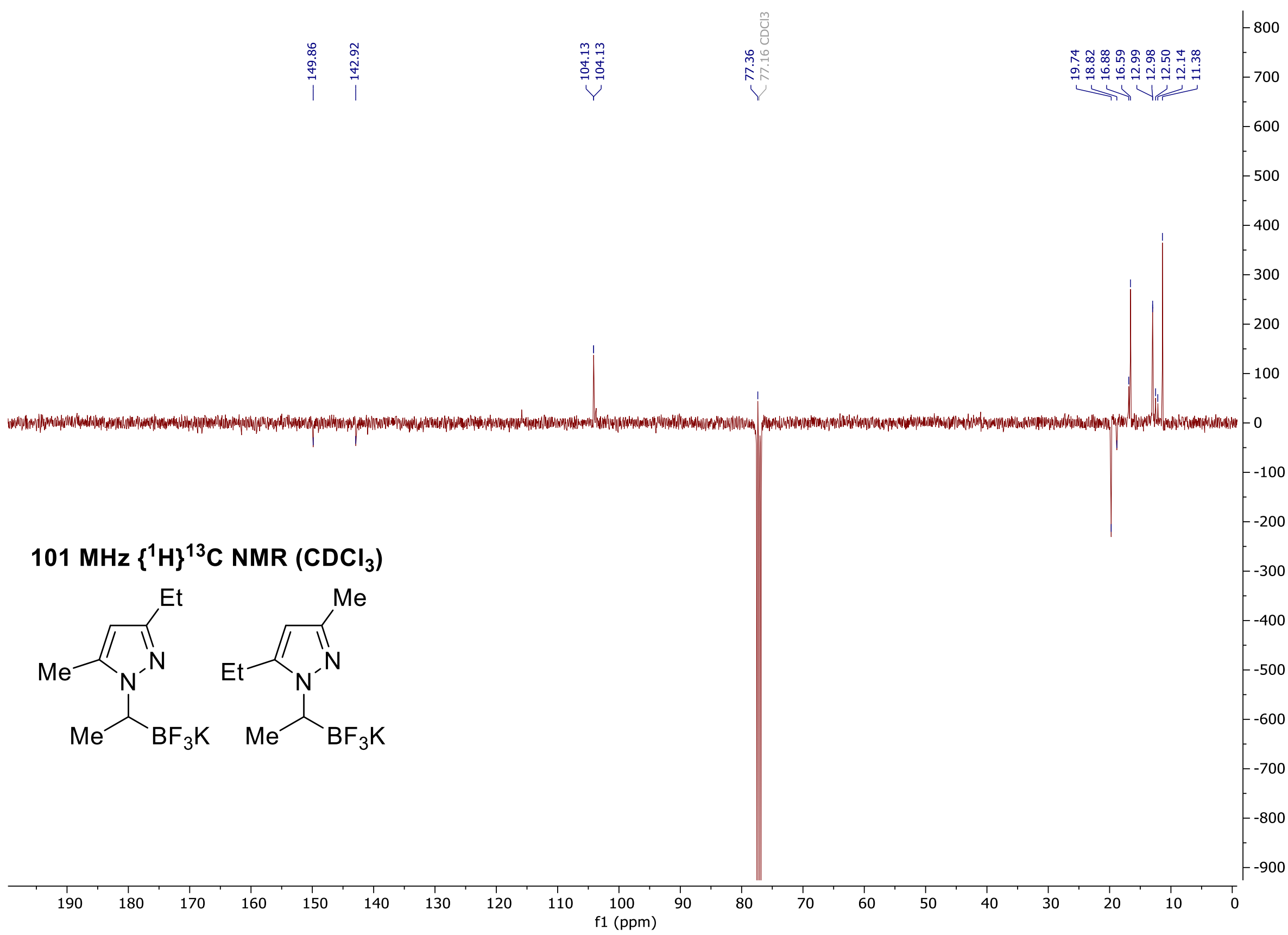
101 MHz {1H}13C NMR (CDCl3)
149.86
142.92
104.13
104.13
77.36
77.16 CDCl3
19.74
18.82
16.88
16.59
12.99
12.98
12.50
12.14
11.38
f1 (ppm)

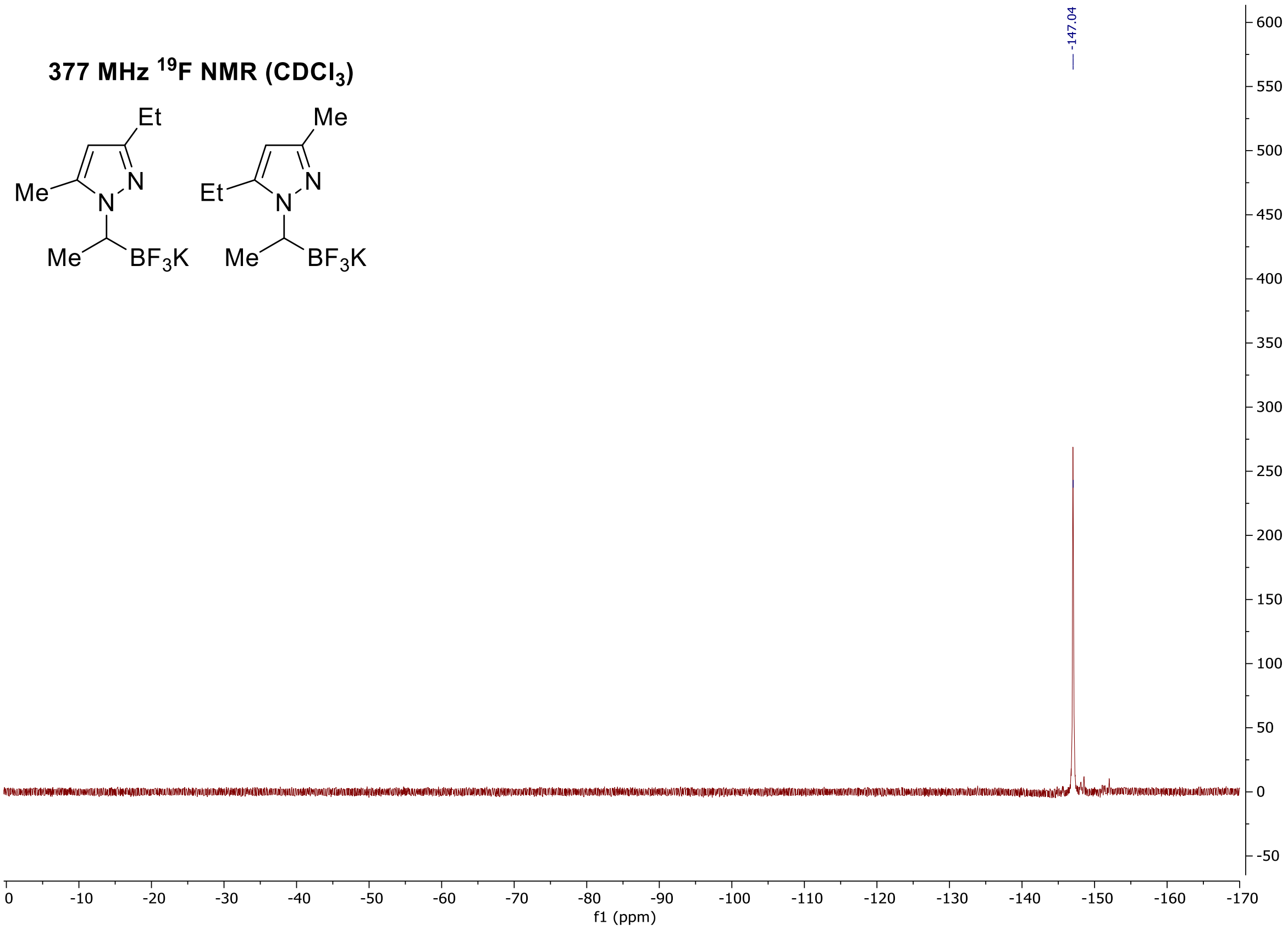
377 MHz 19F NMR (CDCl3)
Et
Me
Me
N
N
Et
N
N
Me
BF3K
Me
BF3K
-147.04
f1 (ppm)

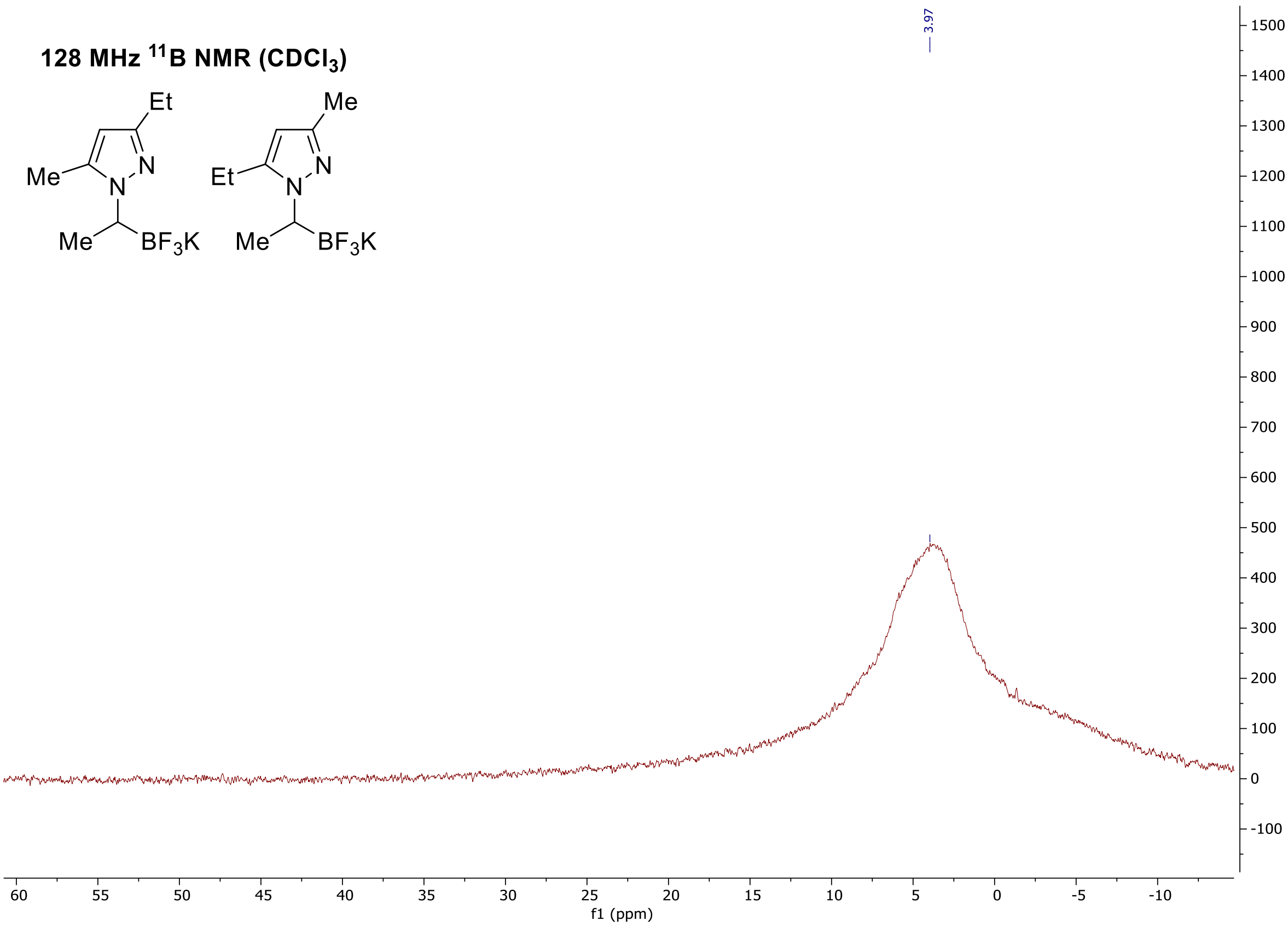
128 MHz 11B NMR (CDCl3)
Et
Me
Me
N
N
Et
N
N
Me
BF3K
Me
BF3K
3.97
f1 (ppm)

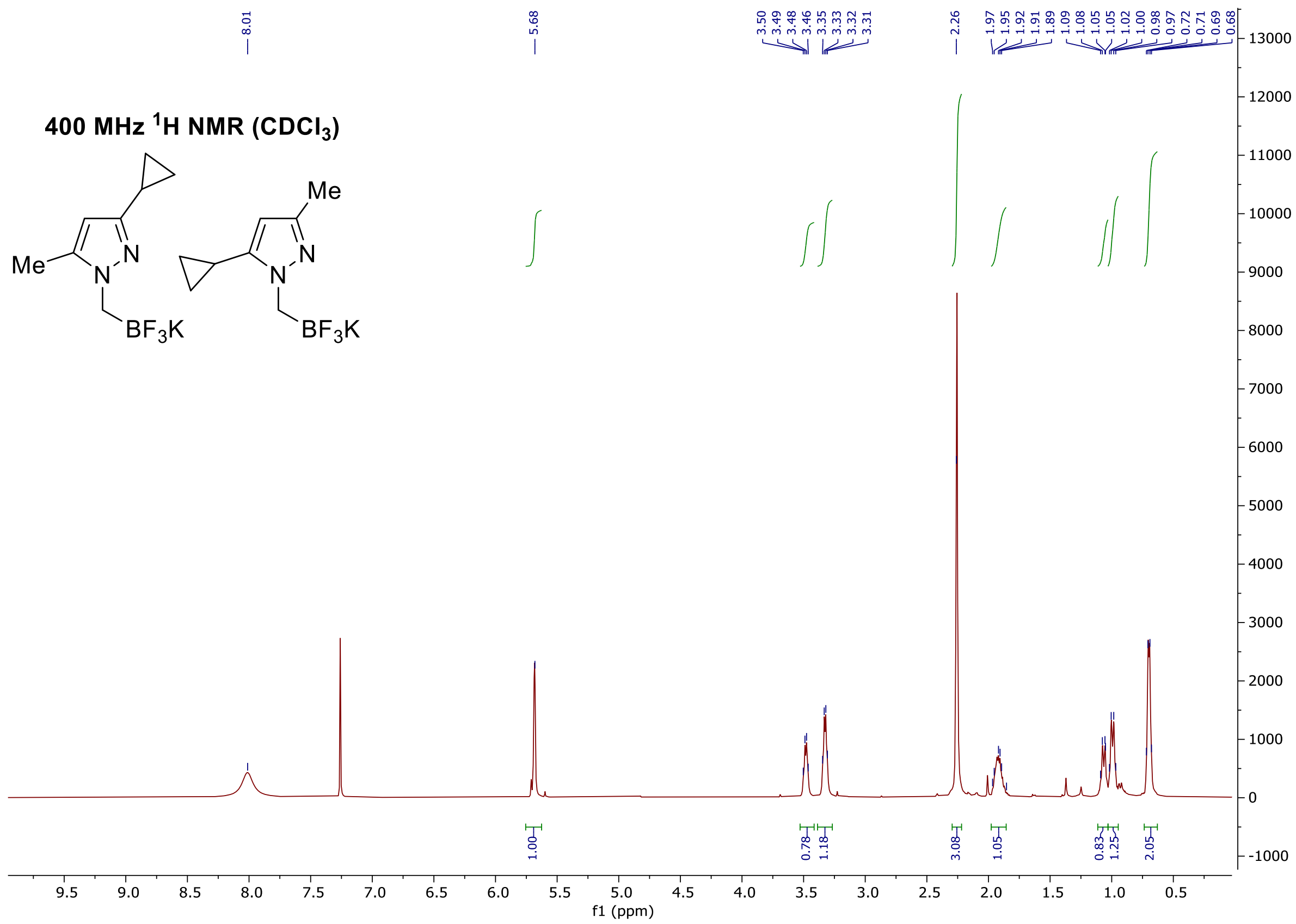
400 MHz ¹H NMR (CDCl₃)
Me
N
N
BF₃K
f1 (ppm)

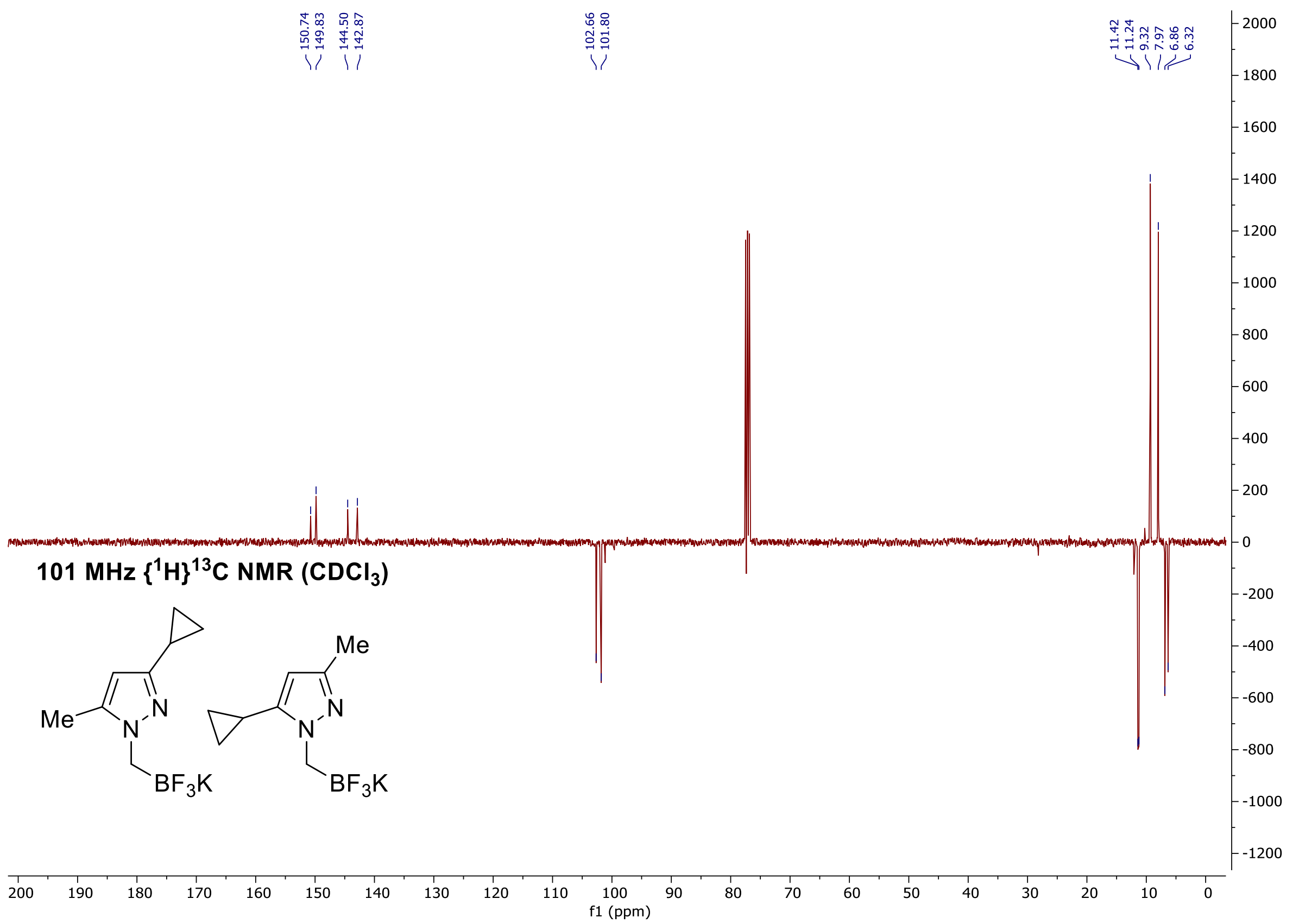
101 MHz {¹H}¹³C NMR (CDCl₃)
Me
N
N
BF₃K
f1 (ppm)

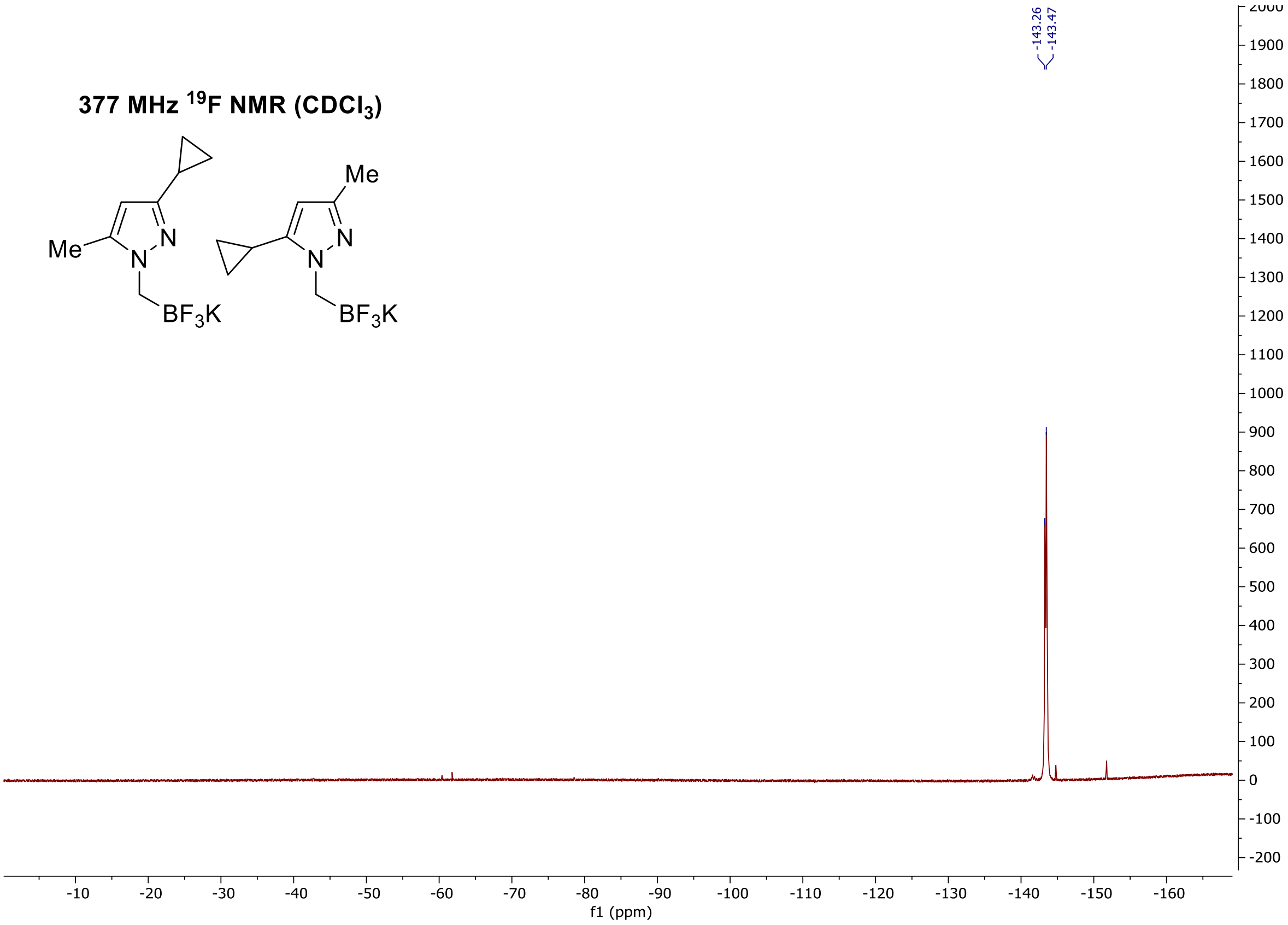
377 MHz 19F NMR (CDCl3)
Me
N
N
BF3K
-143.26
-143.47
f1 (ppm)

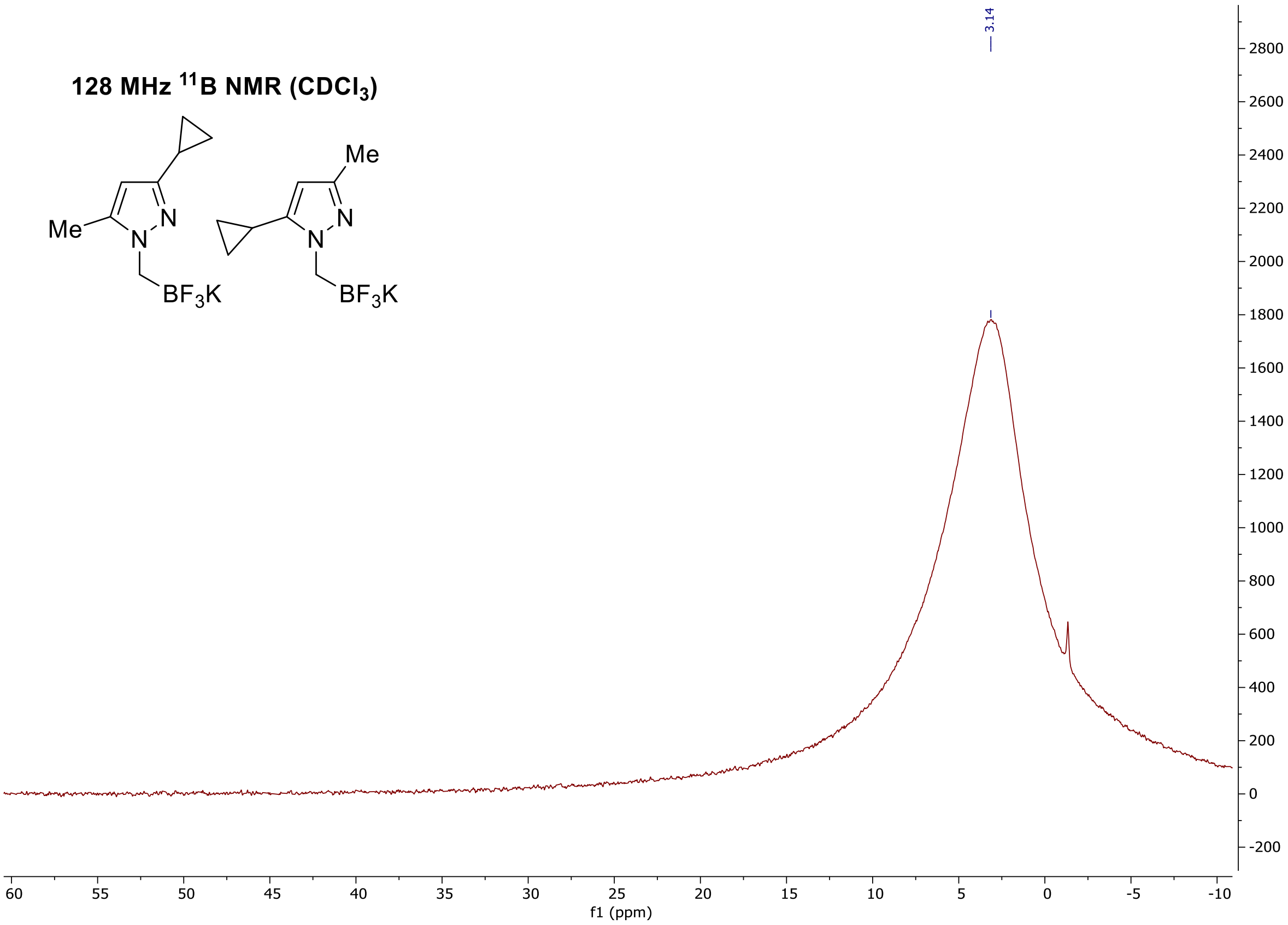
128 MHz 11B NMR (CDCl3)
Me
N
N
BF3K
3.14
f1 (ppm)

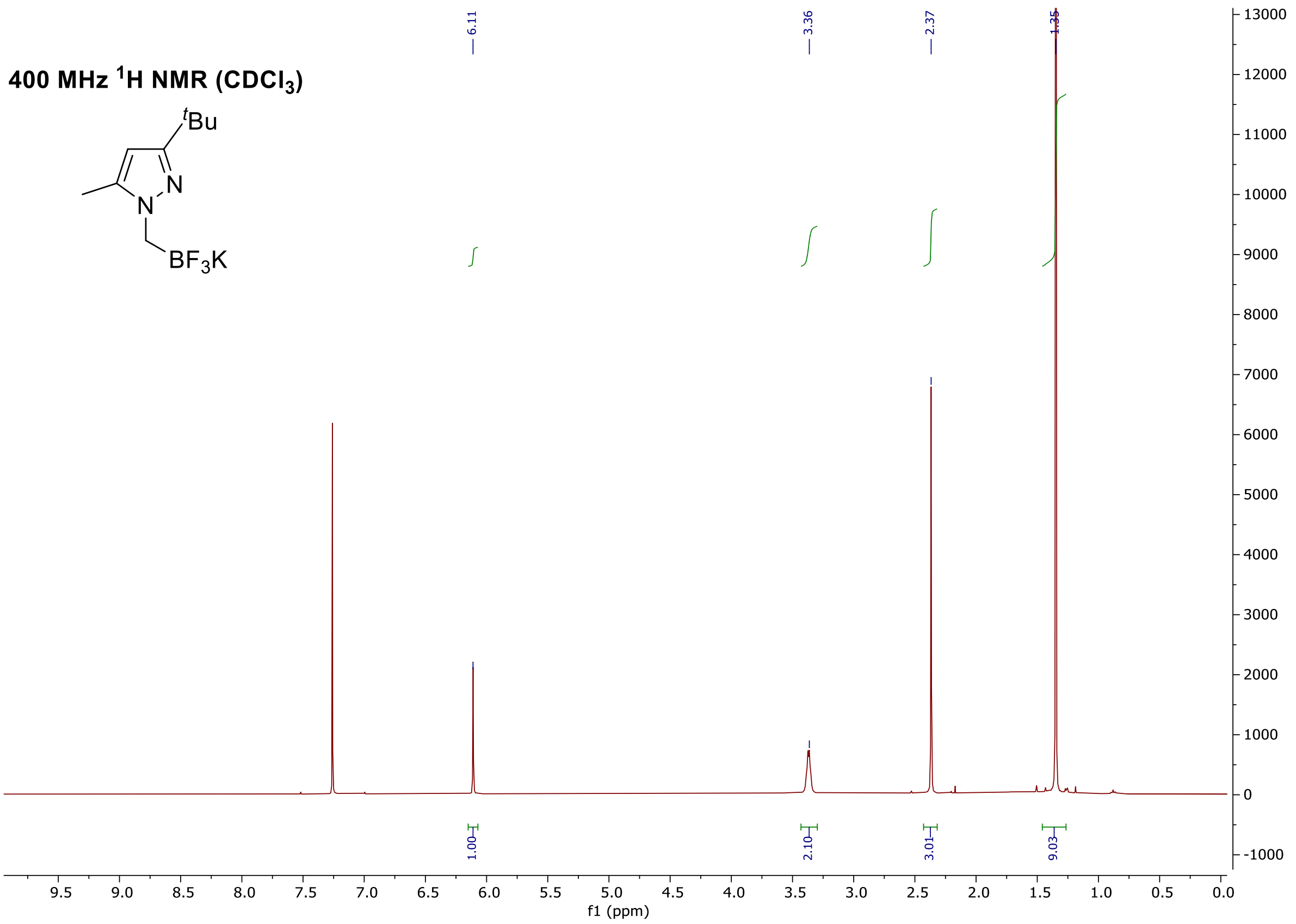

400 MHz ^1H NMR ($CDCl_3$)
^{t}Bu
N
N
BF_3K
6.11
3.36
2.37
1.35
1.00
2.10
3.01
9.03
f1 (ppm)

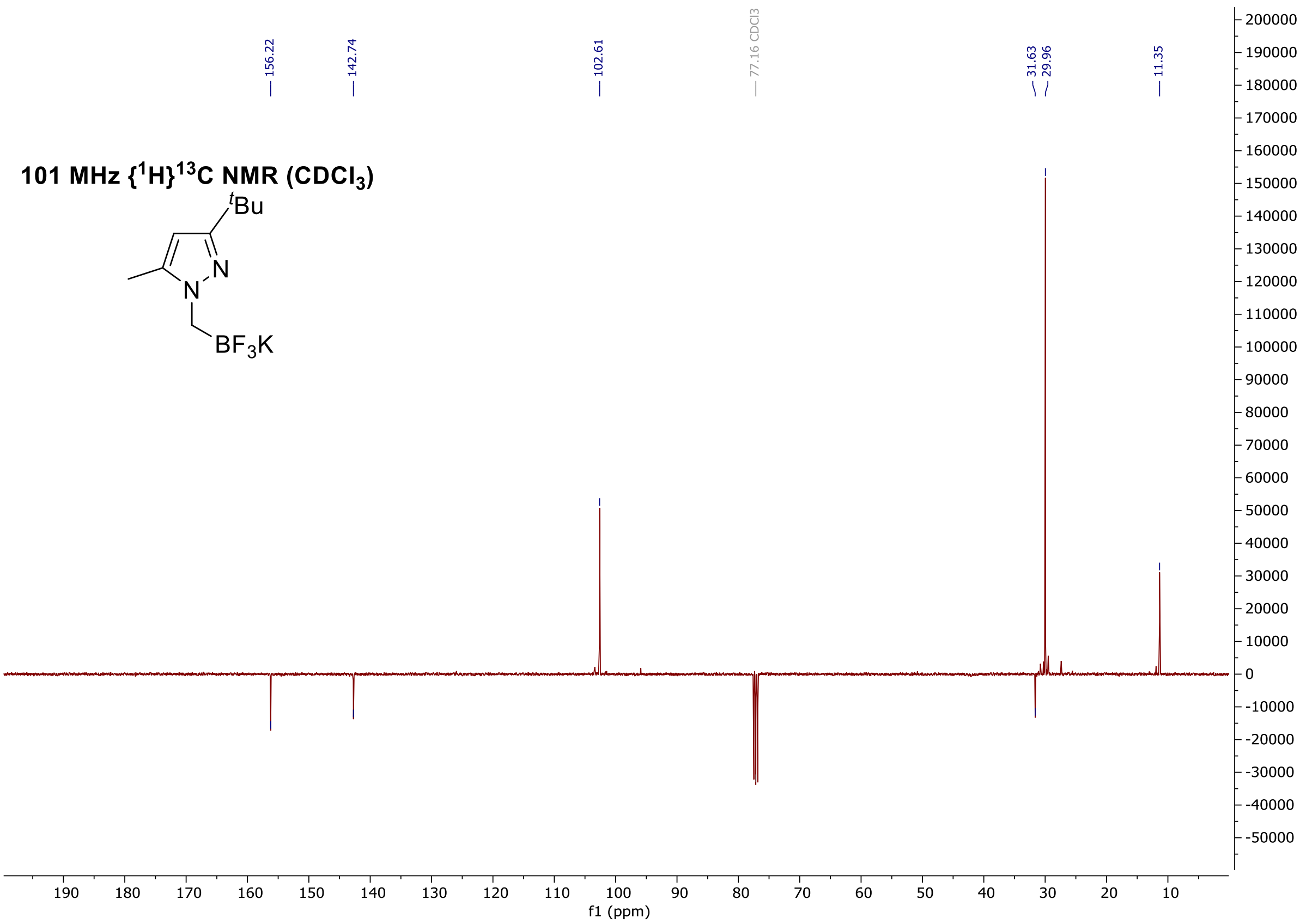

101 MHz {^1H}^{13}C NMR ($CDCl_3$)
^{t}Bu
N
N
BF_3K
156.22
142.74
102.61
77.16 CDCl3
31.63
29.96
11.35
f1 (ppm)

377 MHz ^{19}F NMR ($CDCl_3$)

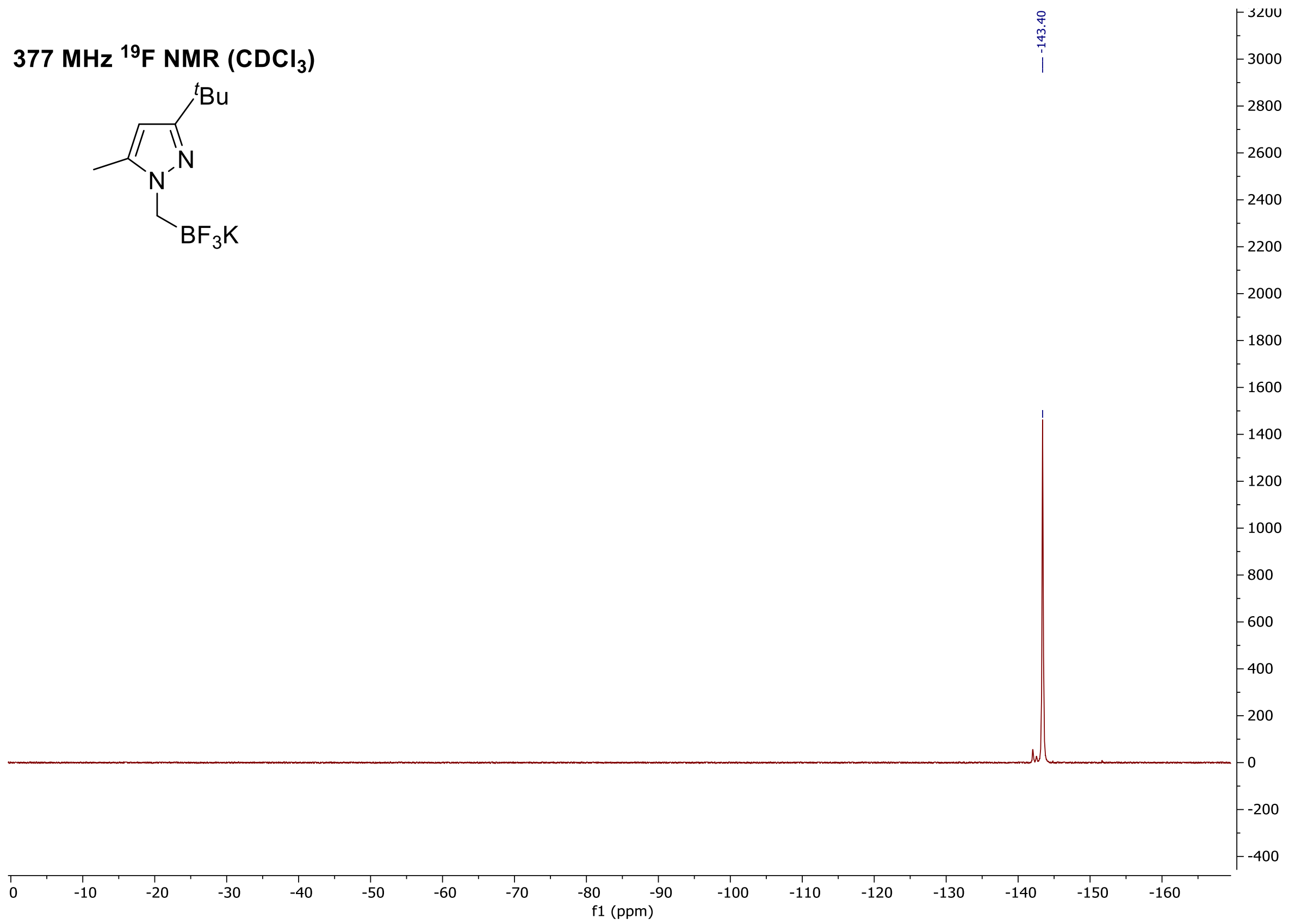

128 MHz ^{11}B NMR ($CDCl_3$)

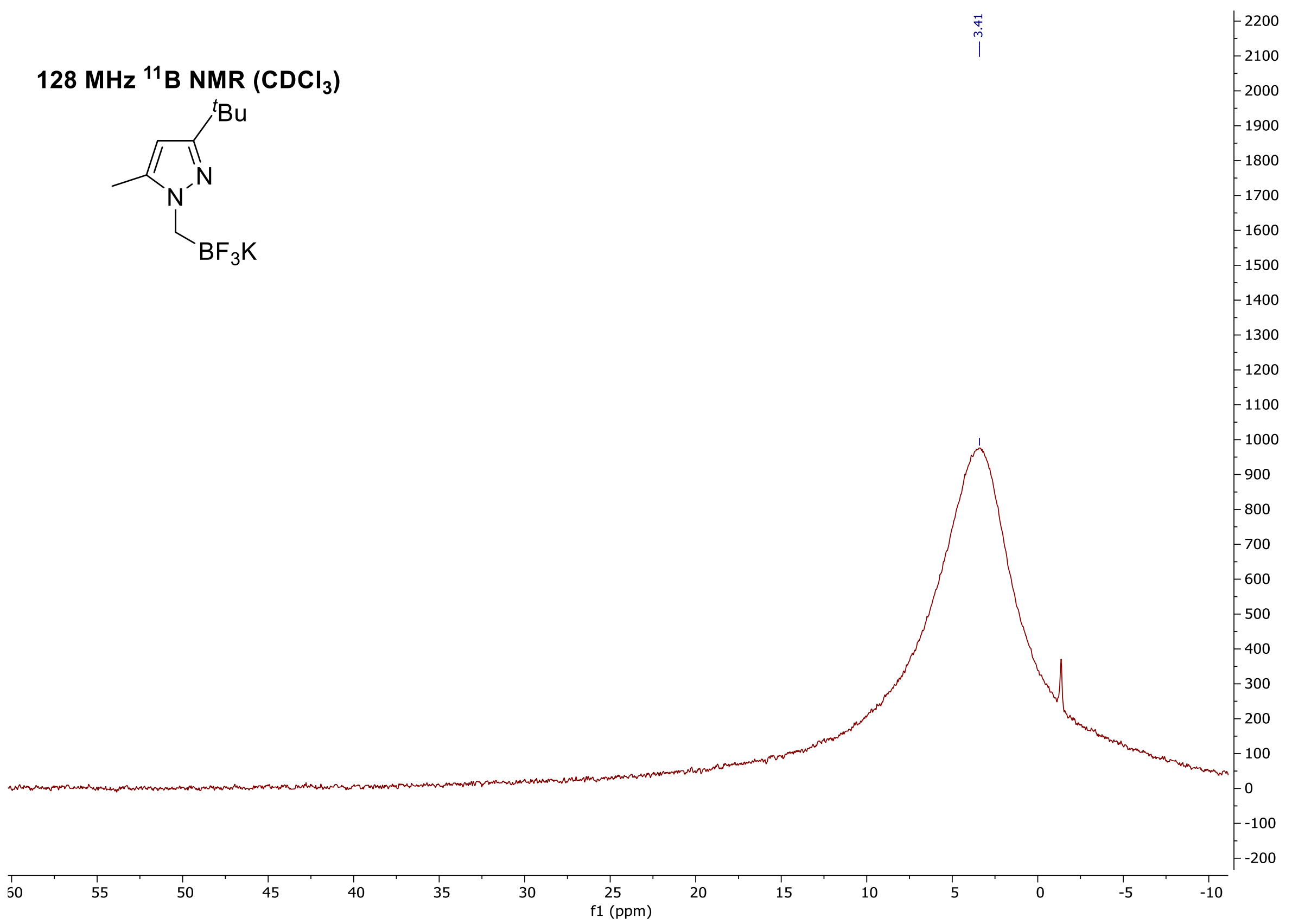

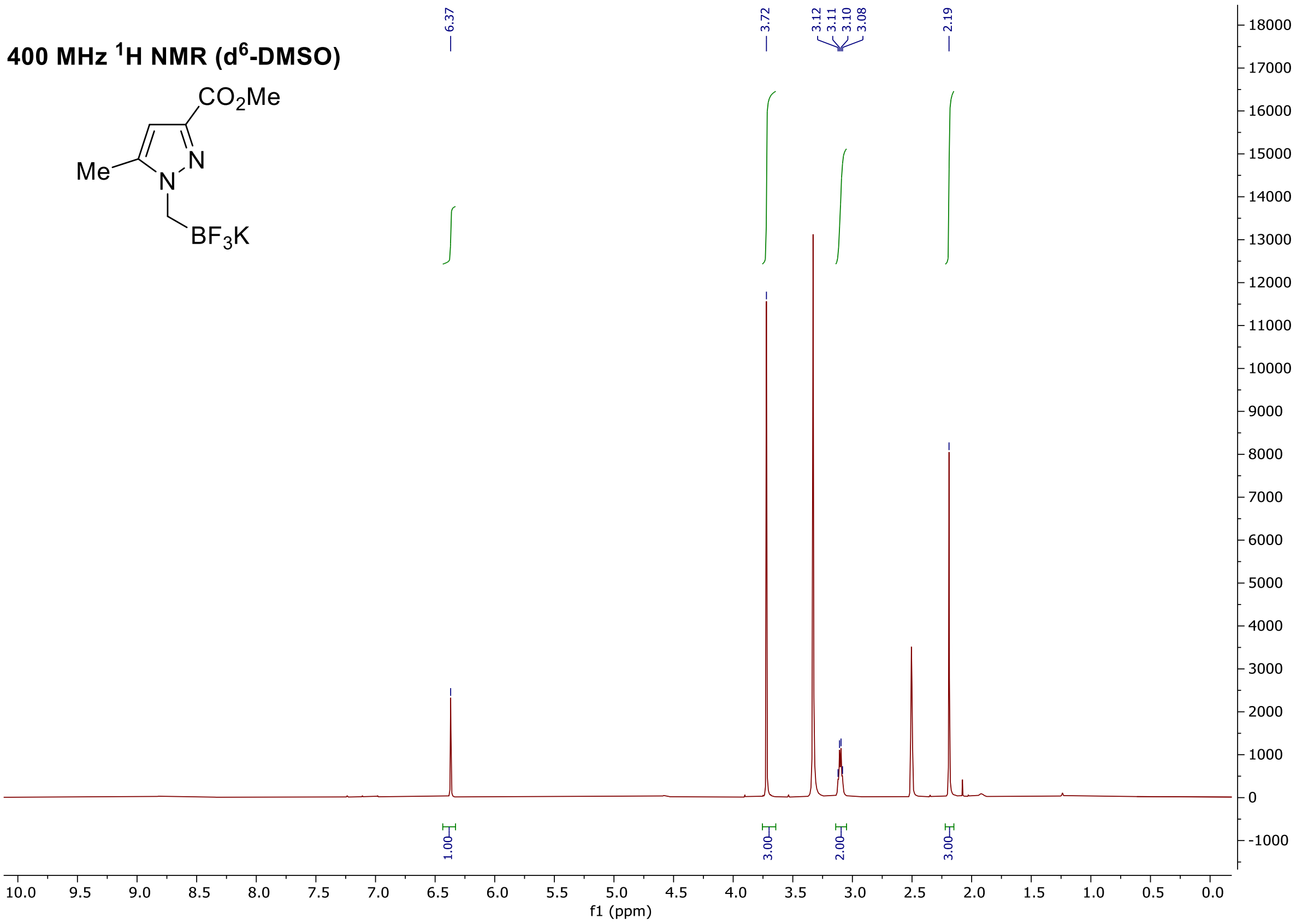
400 MHz ^{1}H NMR (d^6-DMSO)
CO2Me
Me
N
N
BF3K
6.37
3.72
3.12
3.11
3.10
3.08
2.19
1.00
3.00
2.00
3.00
f1 (ppm)

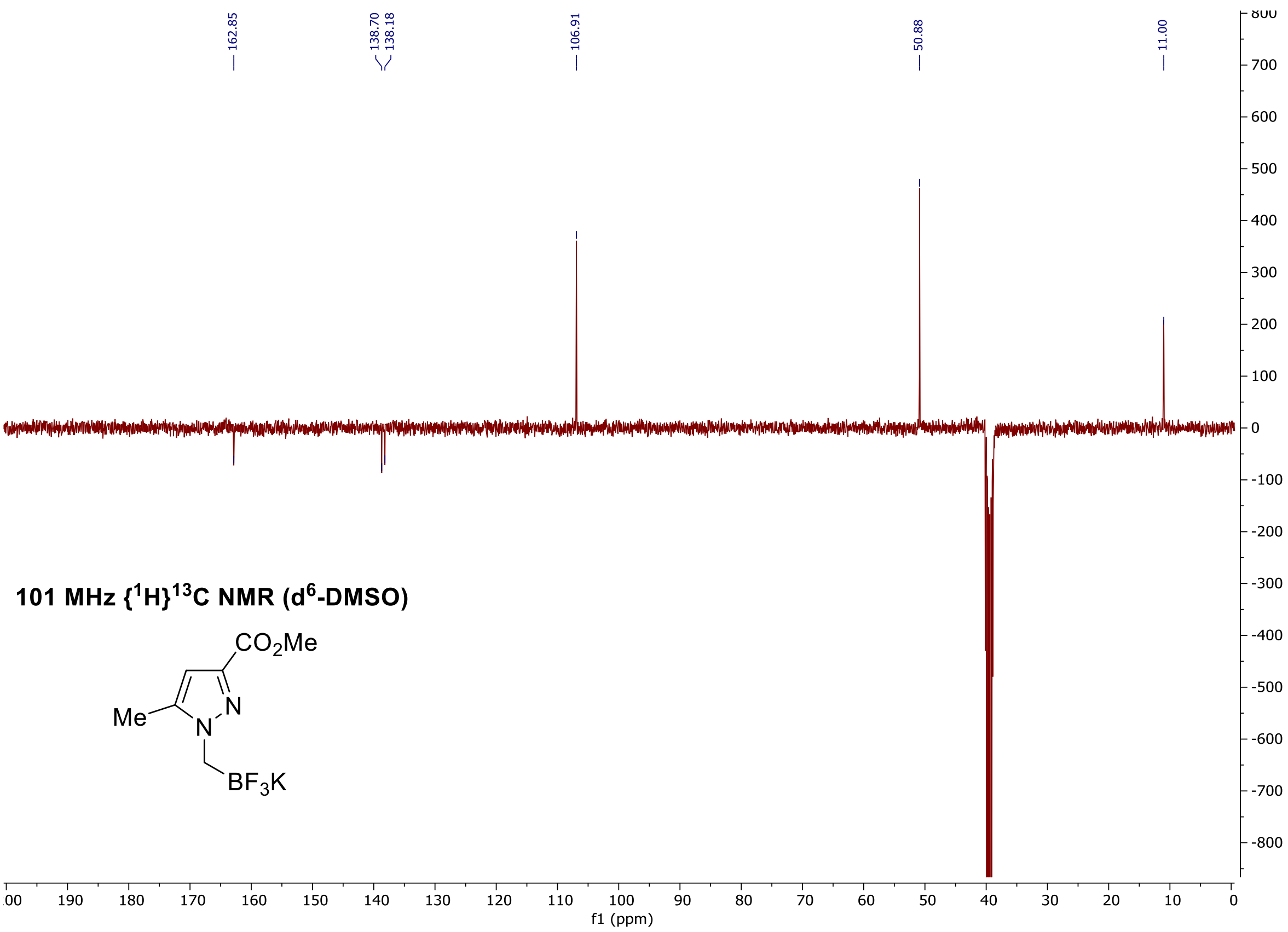
162.85
138.70
138.18
106.91
50.88
11.00
101 MHz {^{1}H}^{13}C NMR (d^6-DMSO)
CO2Me
Me
N
N
BF3K
f1 (ppm)

377 MHz ^{19}F NMR (d^6-DMSO)

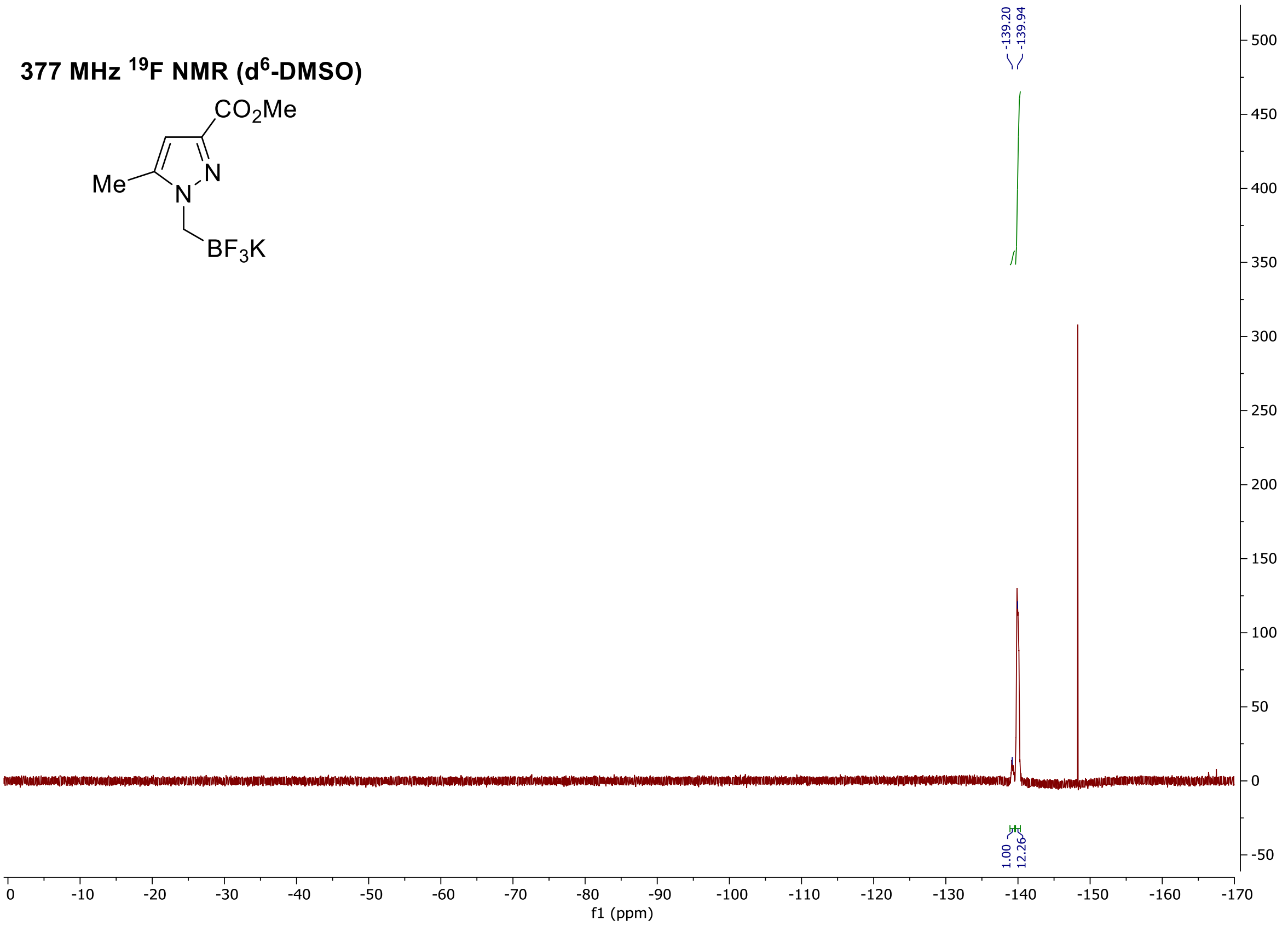

128 MHz ^{11}B NMR (d^6-DMSO)

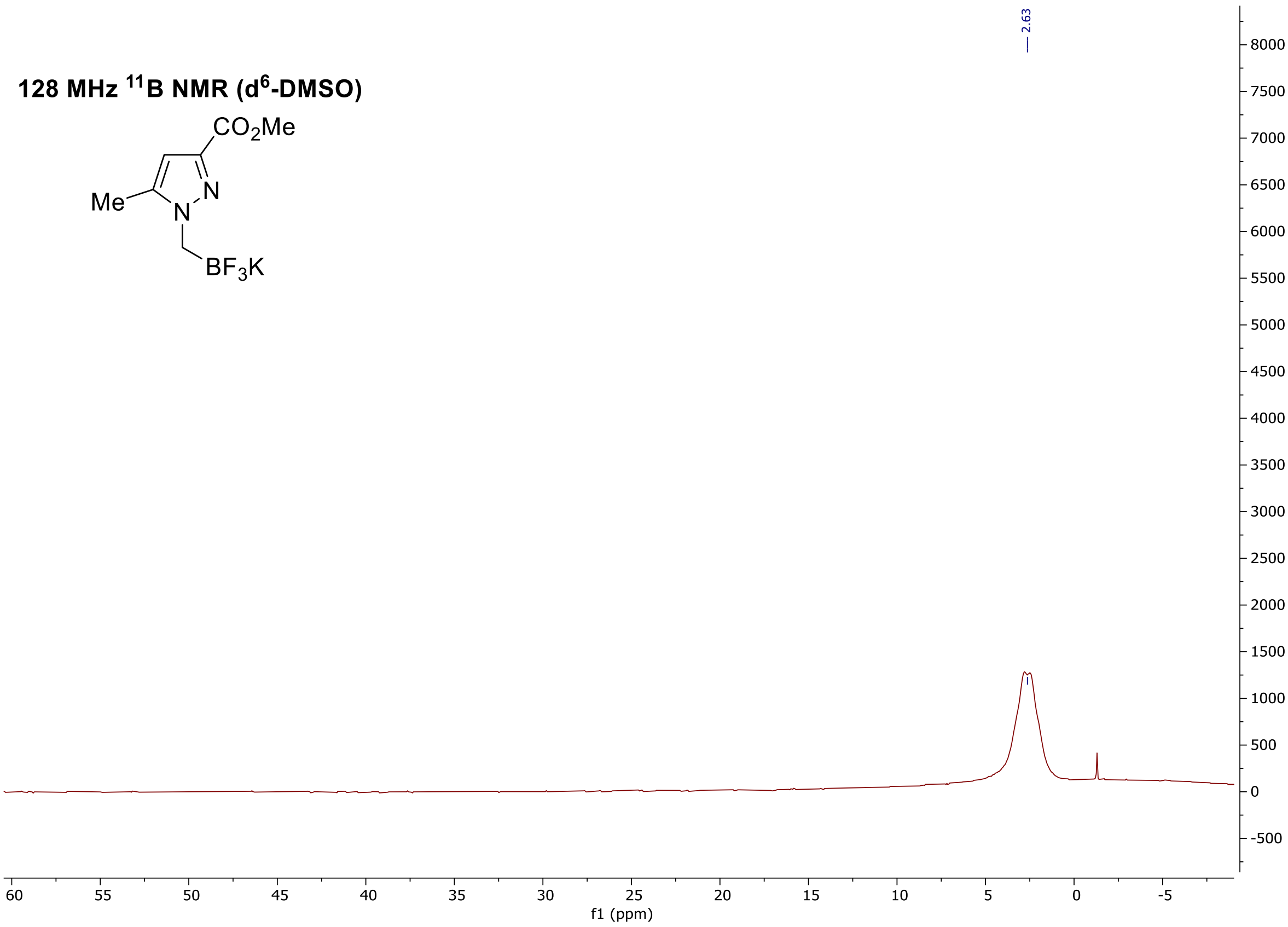

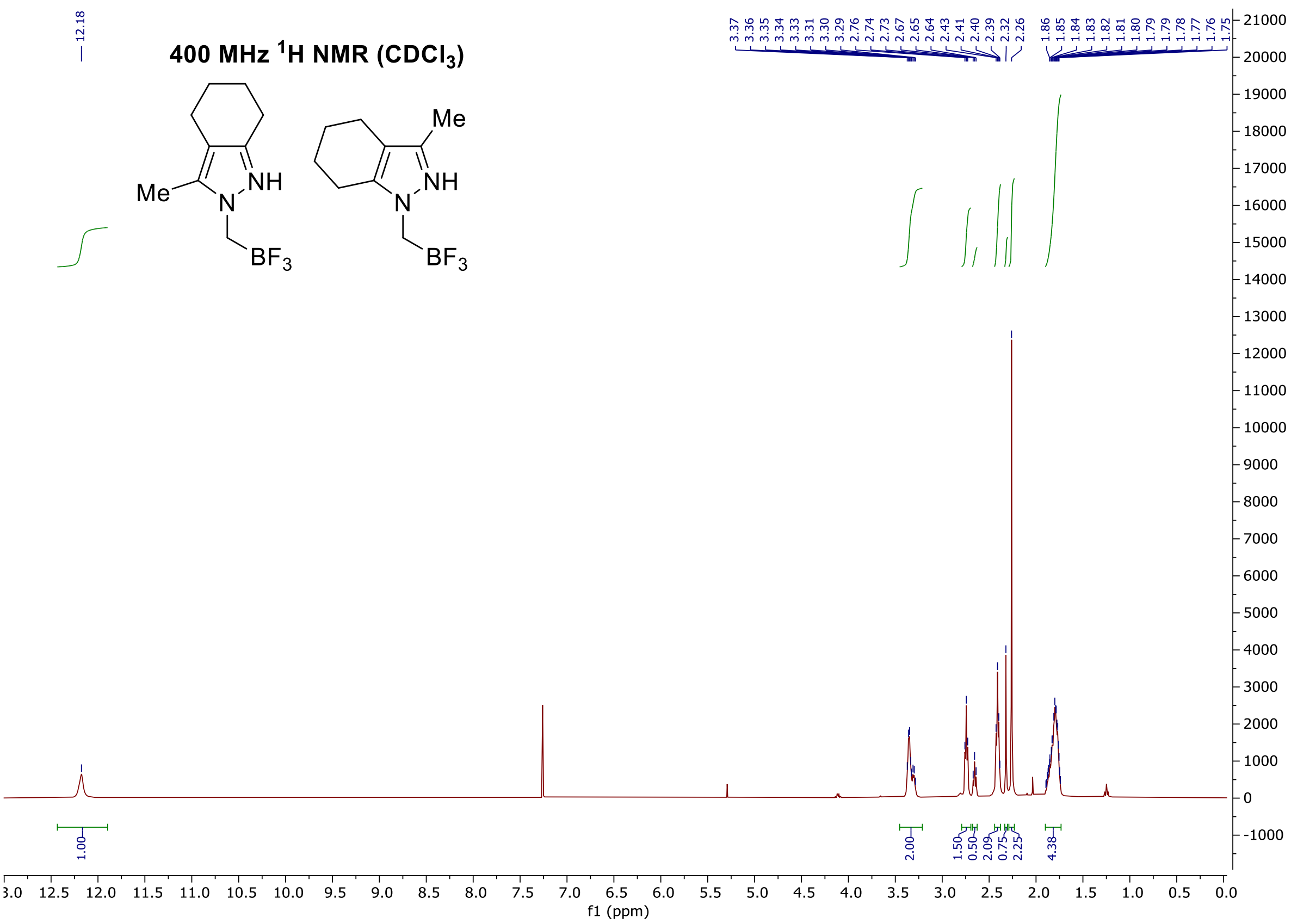
400 MHz ¹H NMR (CDCl₃)
Me
NH
N
BF₃
f1 (ppm)

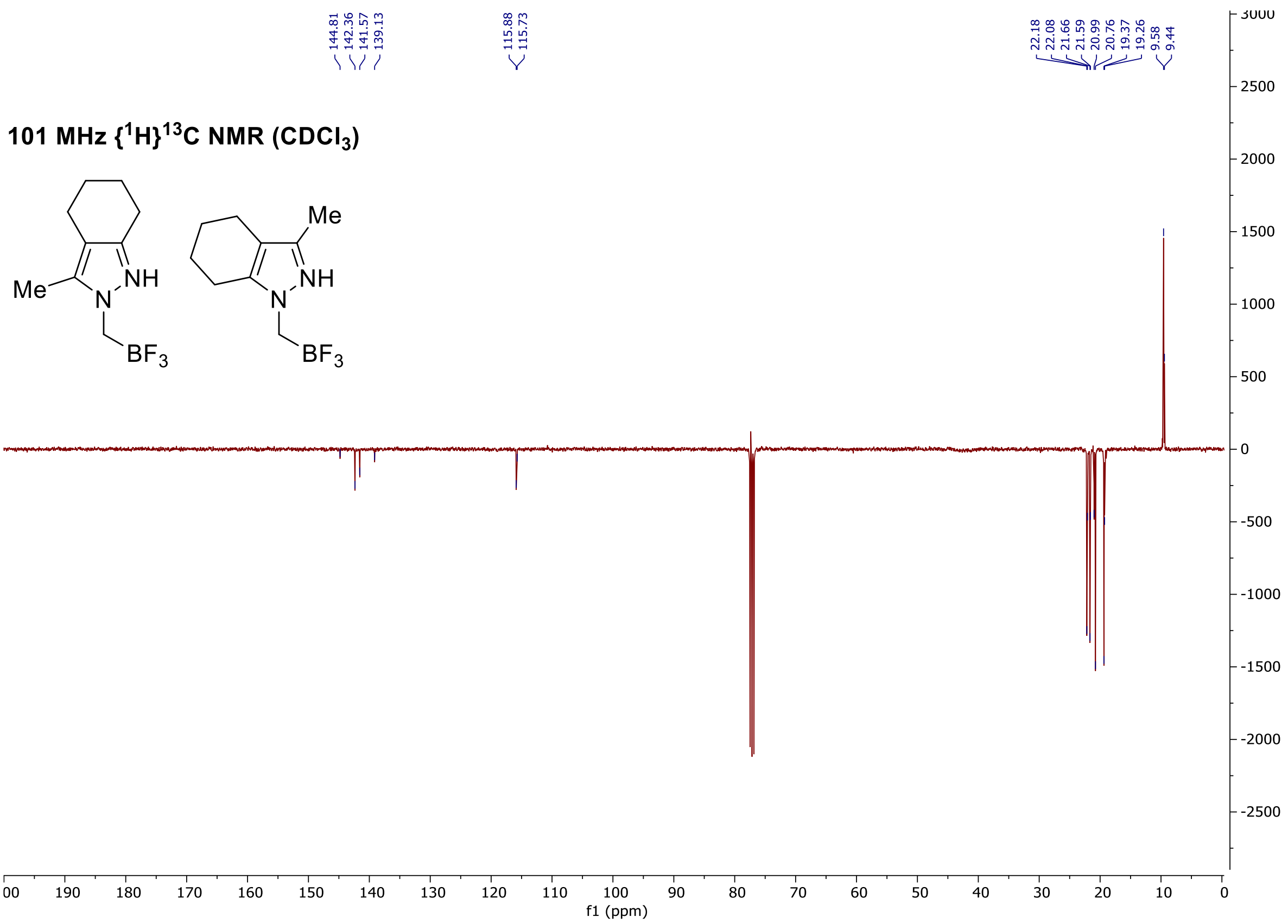
101 MHz {¹H}¹³C NMR (CDCl₃)
Me
NH
N
BF₃
f1 (ppm)

377 MHz ^{19}F NMR ($CDCl_3$)

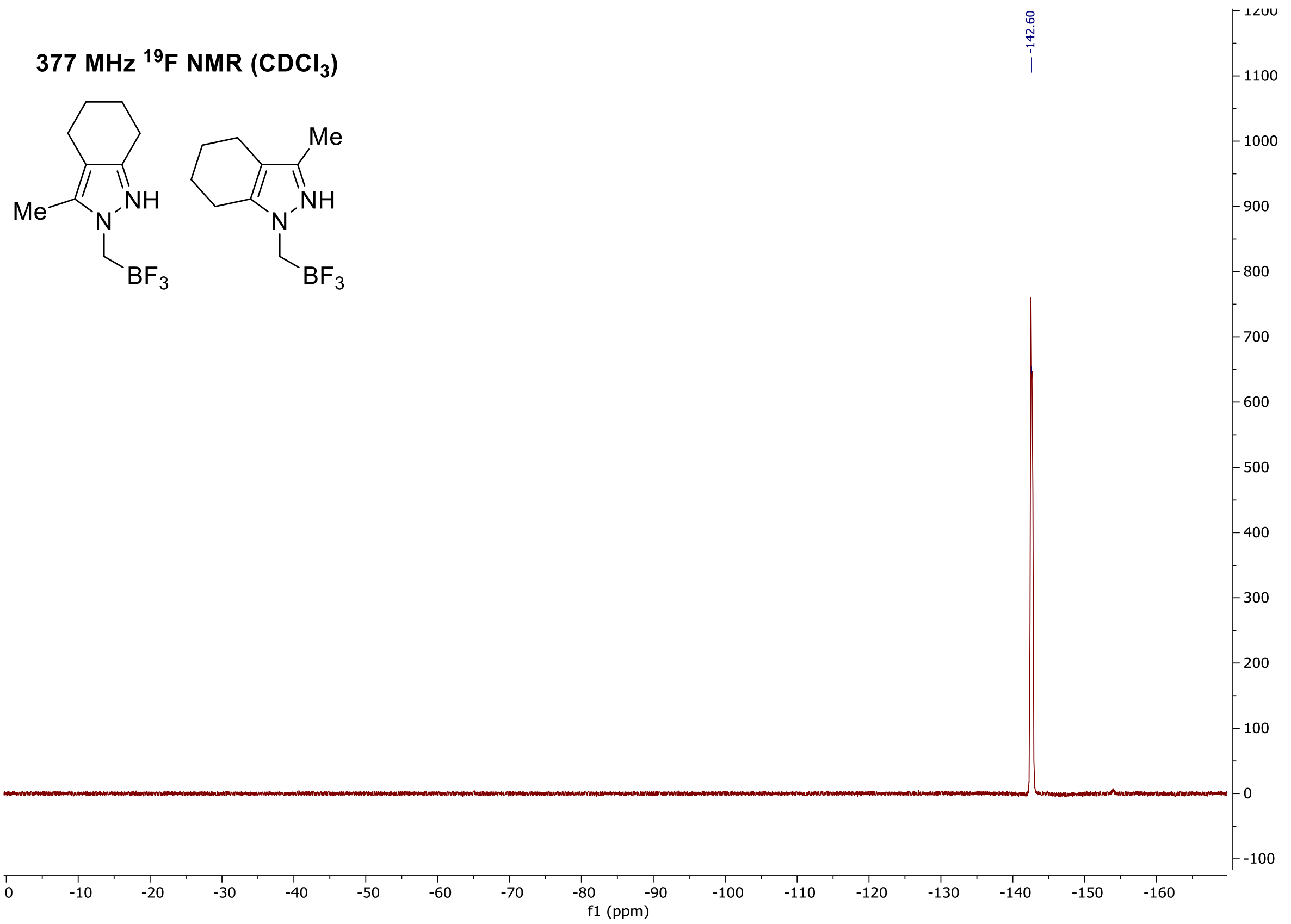

128 MHz ^{11}B NMR ($CDCl_3$)

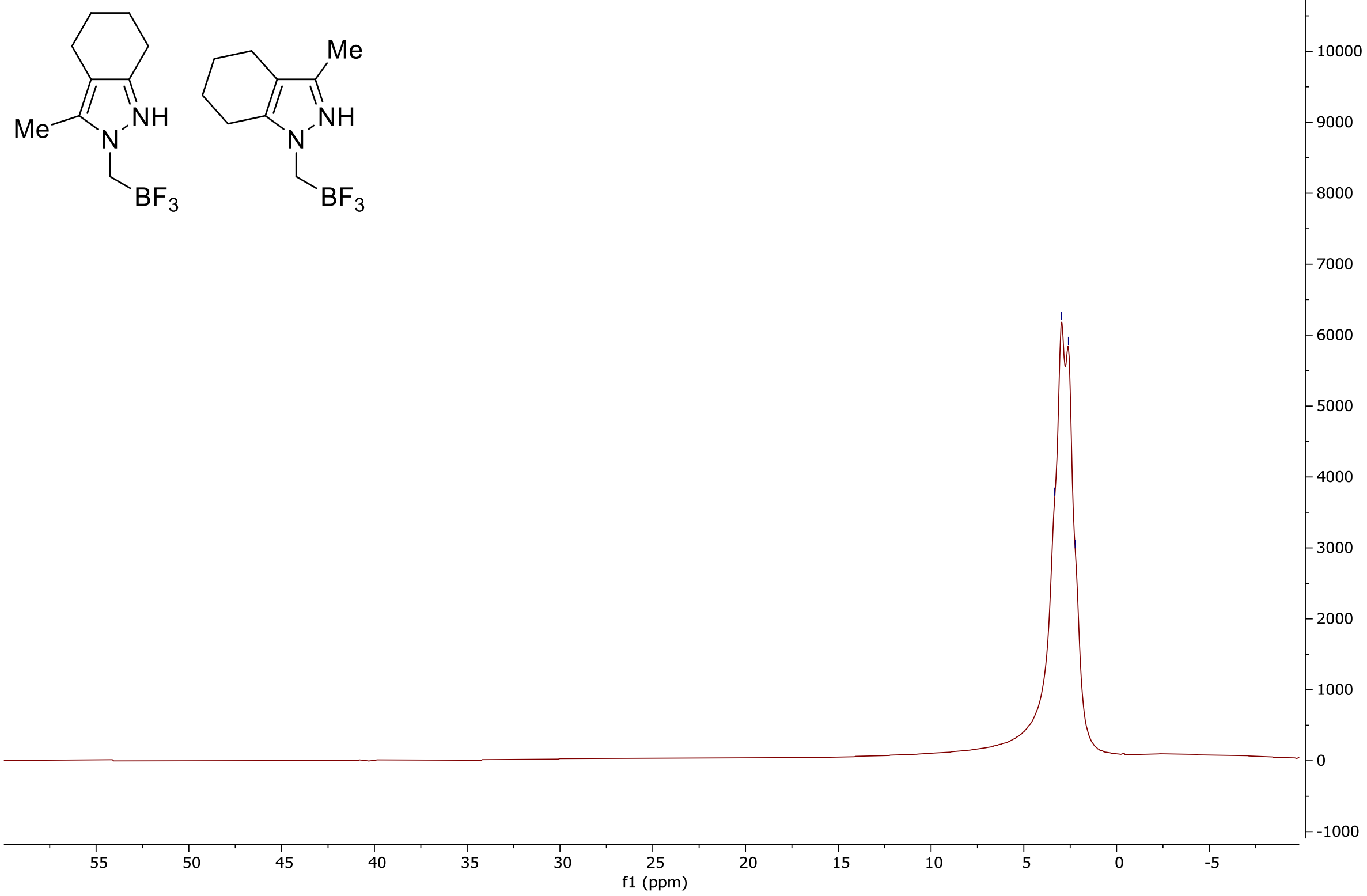

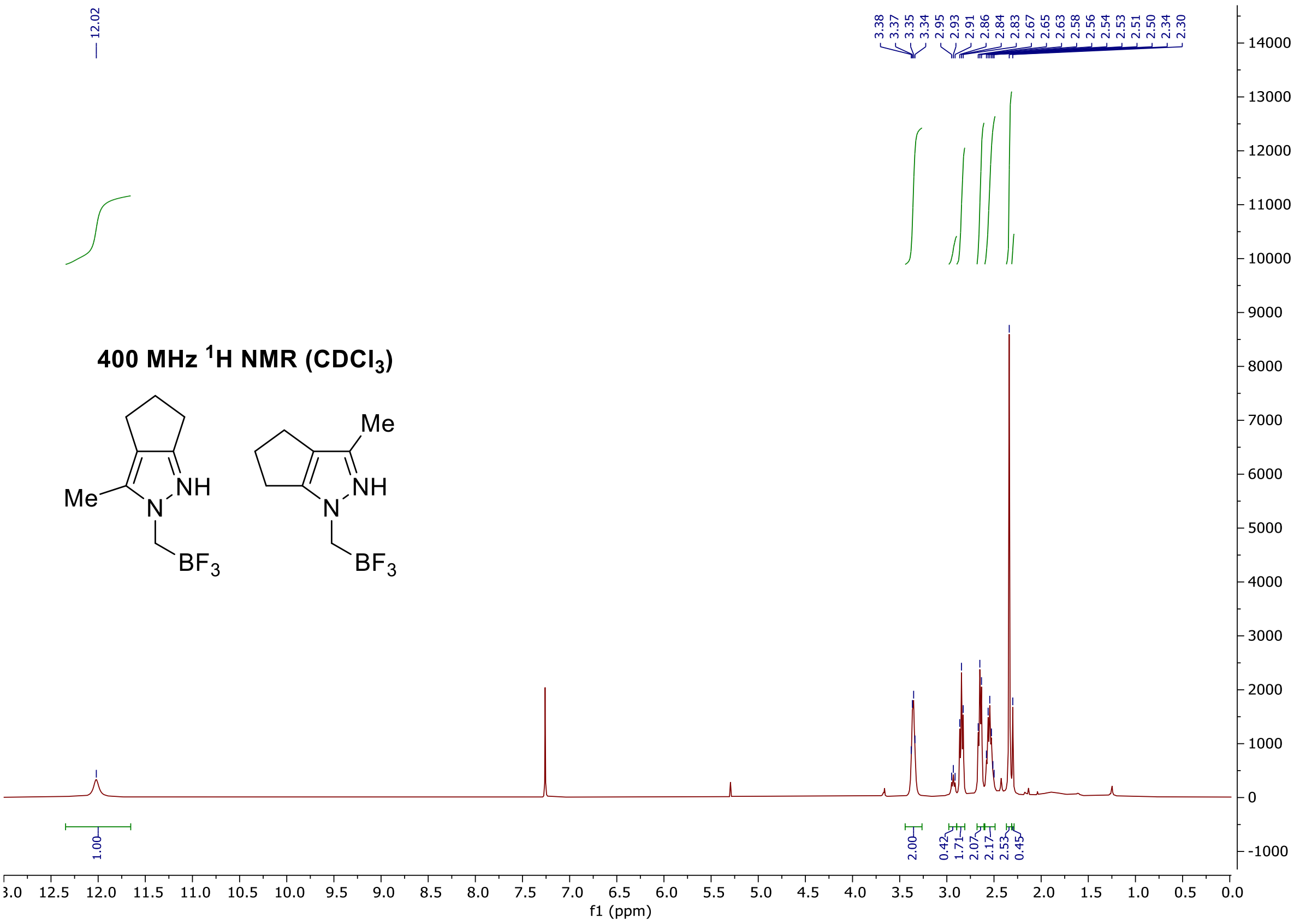
400 MHz ¹H NMR (CDCl₃)
Me
NH
N
BF₃
f1 (ppm)

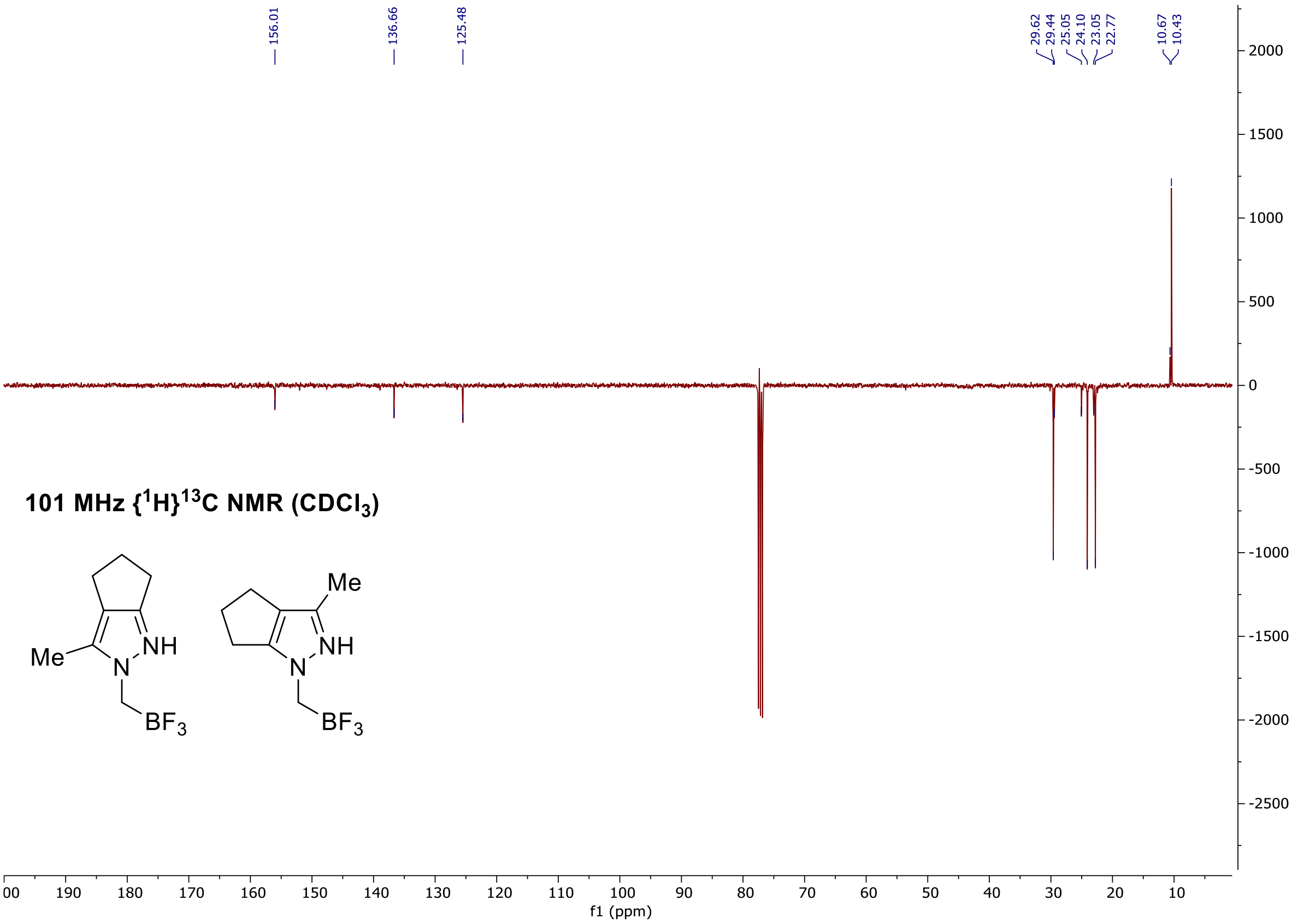
101 MHz {¹H}¹³C NMR (CDCl₃)
Me
NH
N
BF₃
f1 (ppm)

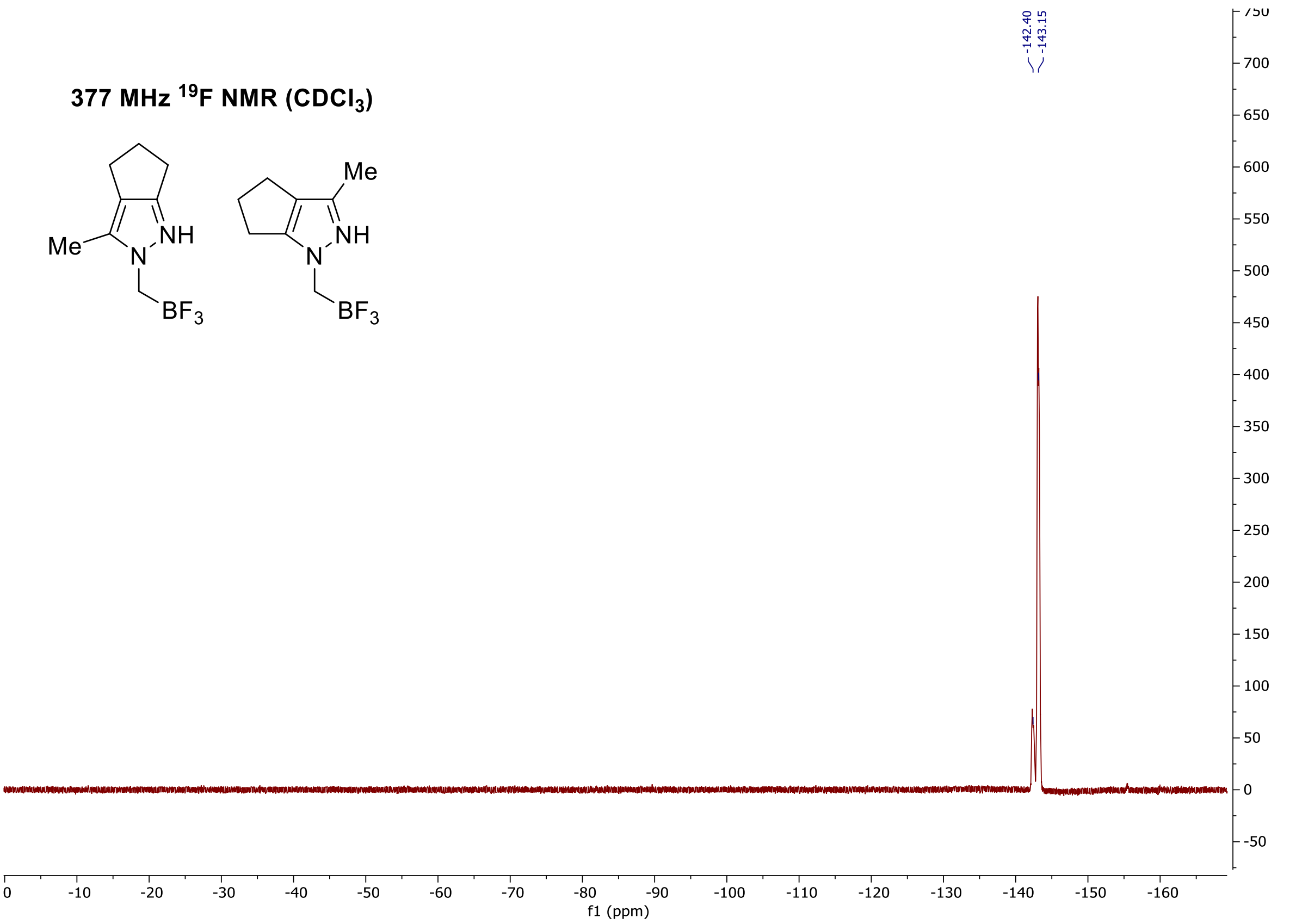
377 MHz ^{19}F NMR ($CDCl_3$)
Me
NH
N
BF_3
-142.40
-143.15
f1 (ppm)

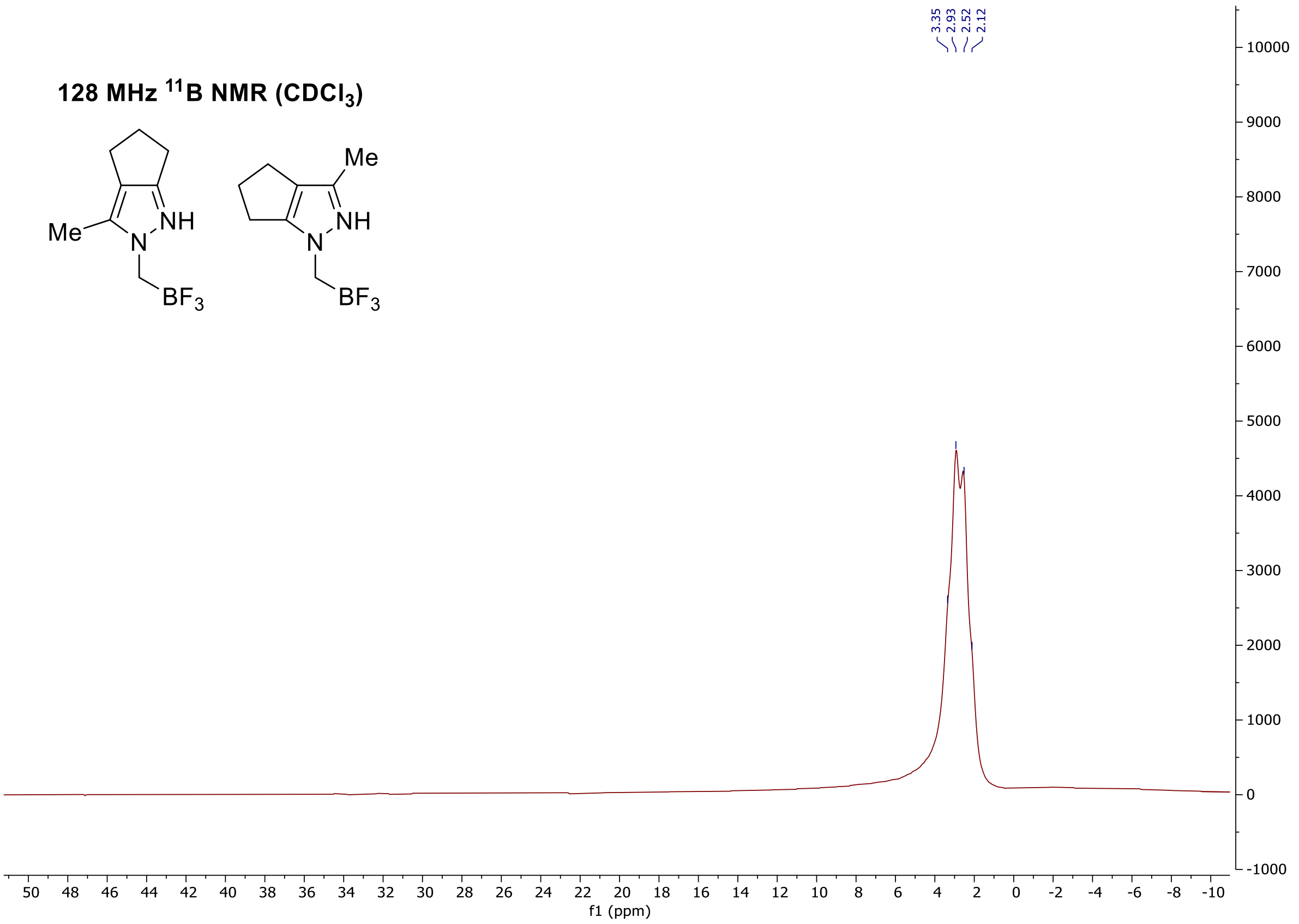
128 MHz ^{11}B NMR ($CDCl_3$)
Me
NH
N
BF_3
3.35
2.93
2.52
2.12
f1 (ppm)

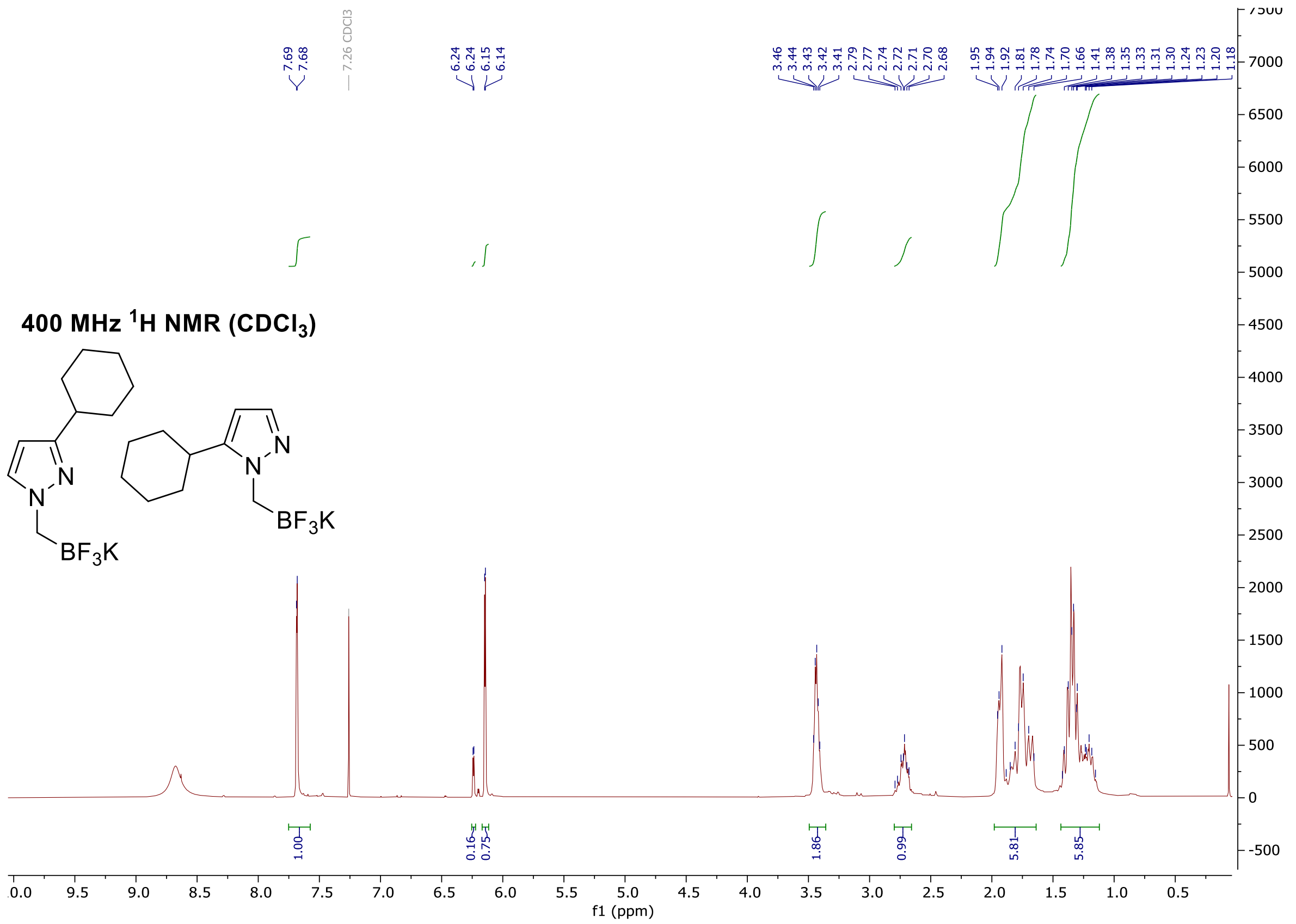
400 MHz ^{1}H NMR ($CDCl_3$)
BF$_3$K
BF$_3$K
f1 (ppm)

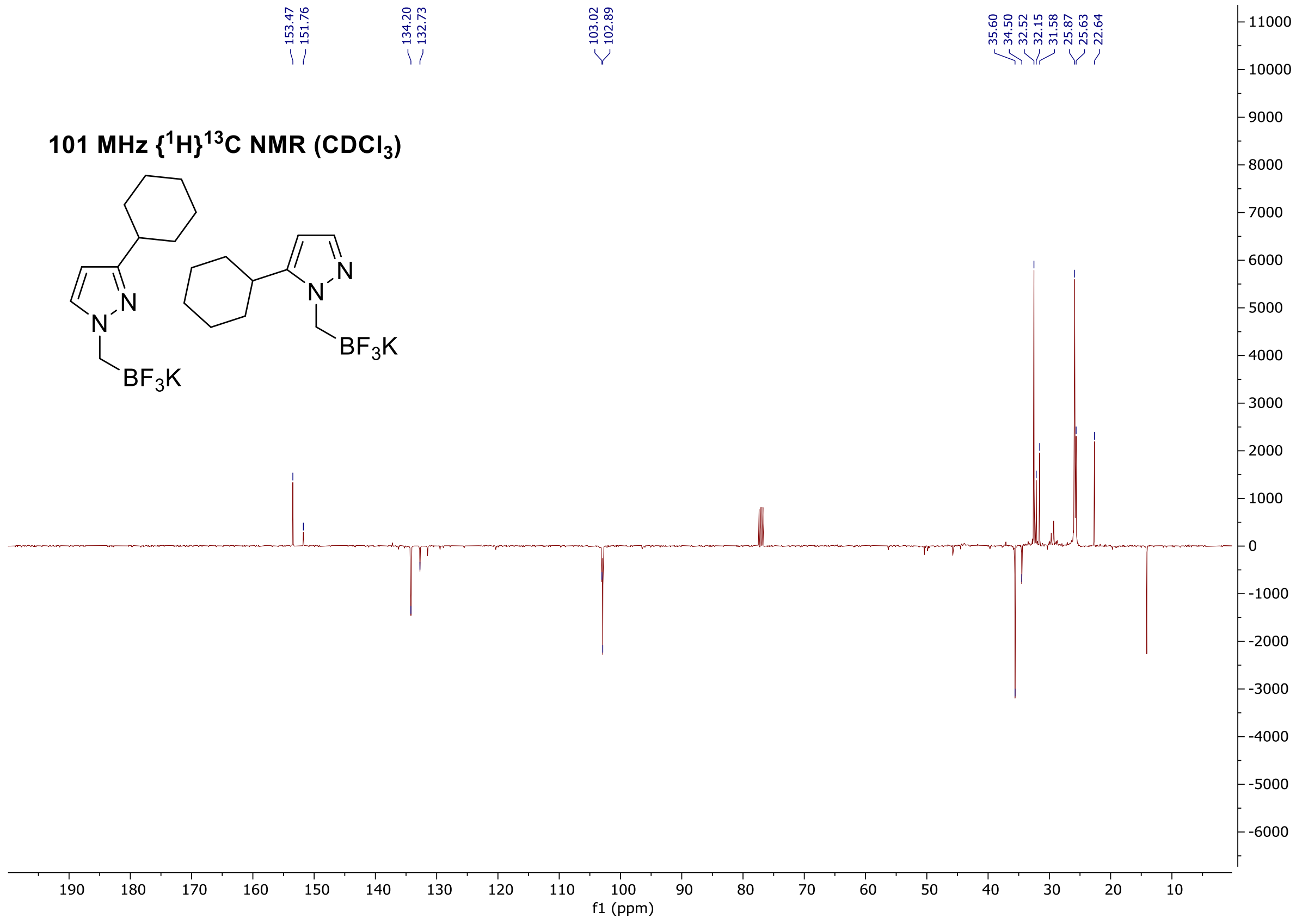
101 MHz {^{1}H}^{13}C NMR ($CDCl_3$)
BF$_3$K
BF$_3$K
f1 (ppm)

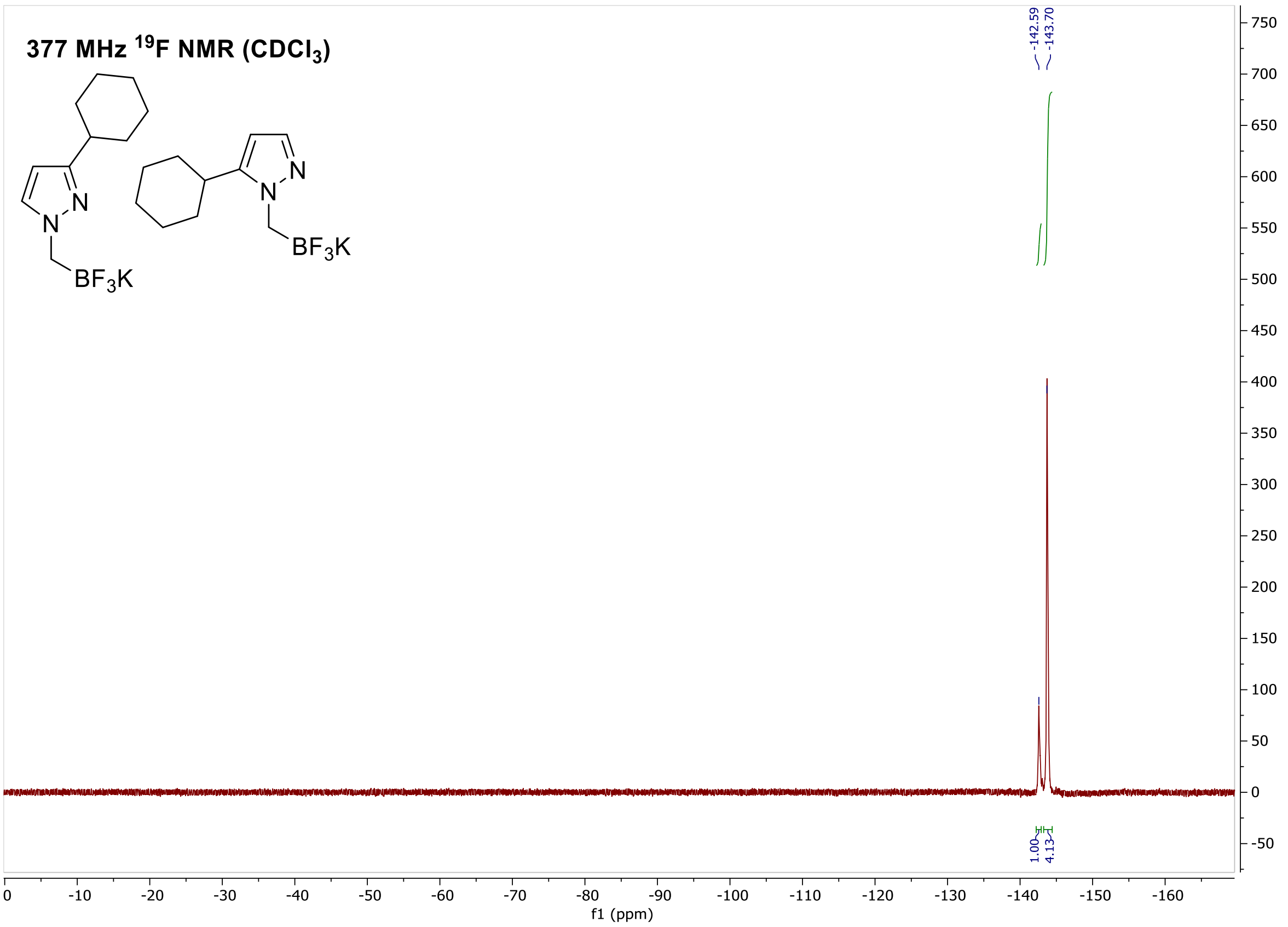

377 MHz ^{19}F NMR ($CDCl_3$)
N
N
BF3K
N
N
BF3K
-142.59
-143.70
1.00
4.13
f1 (ppm)

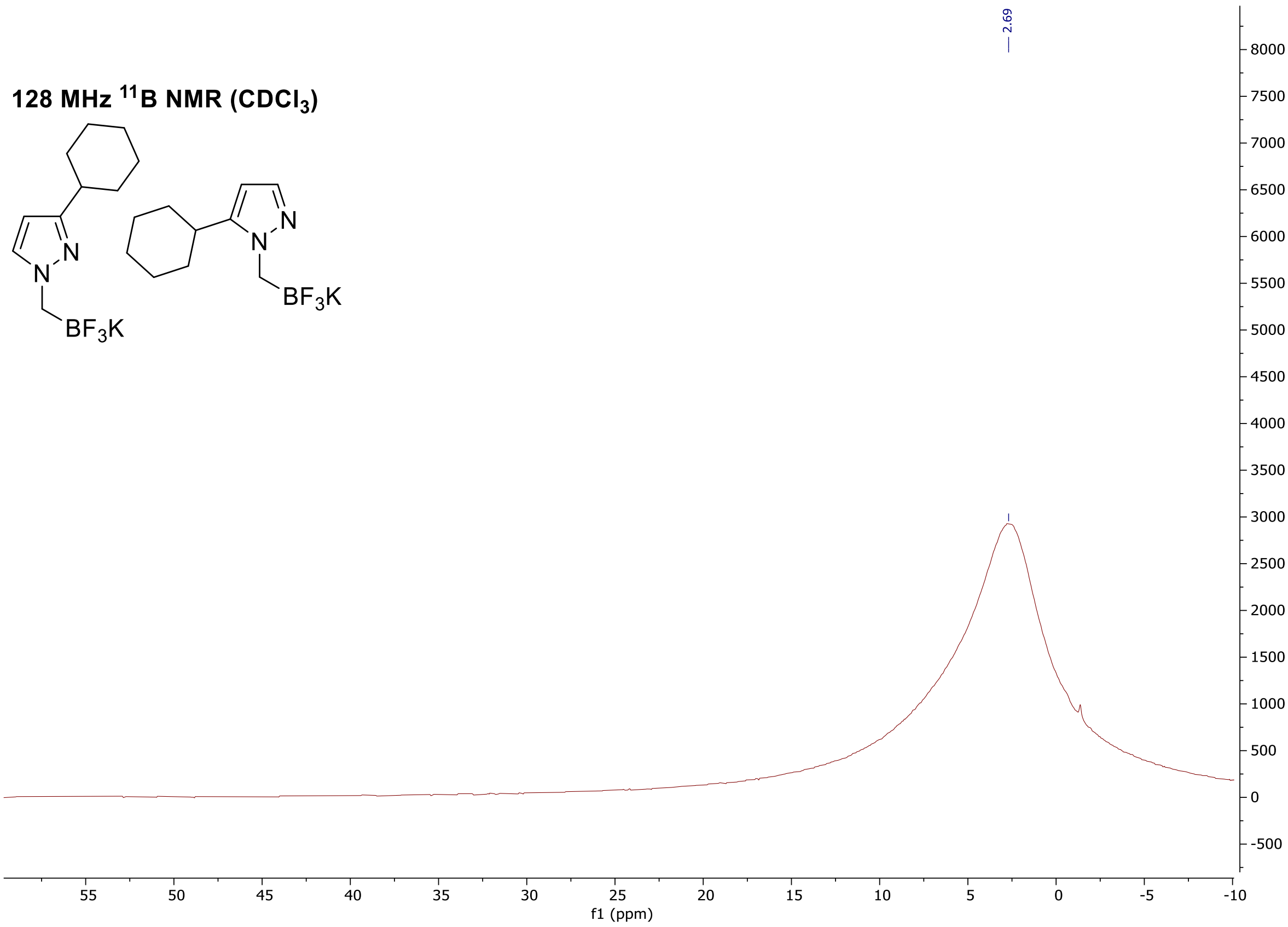

128 MHz ^{11}B NMR ($CDCl_3$)
N
N
BF3K
N
N
BF3K
2.69
f1 (ppm)

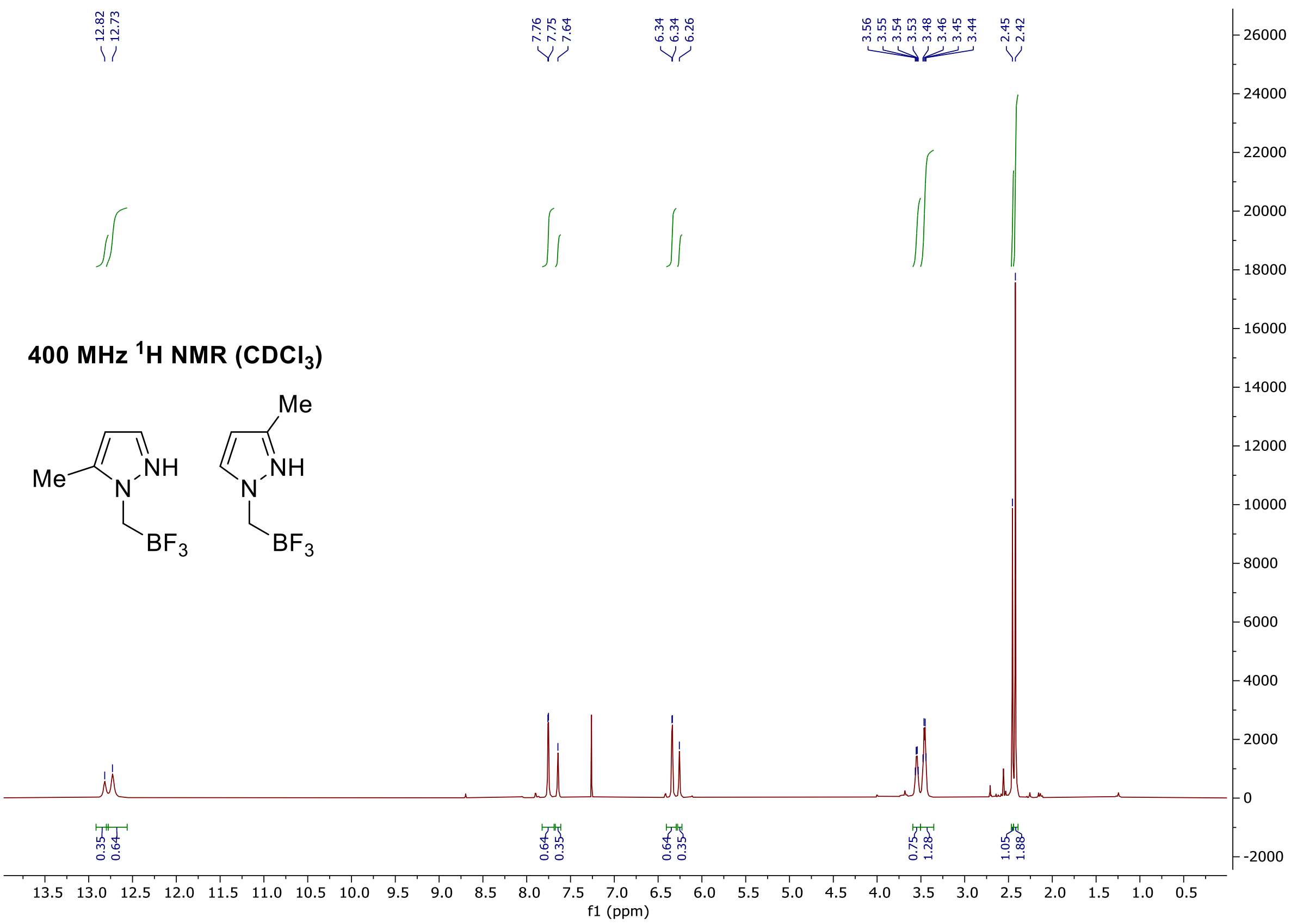

400 MHz 1H NMR (CDCl3)
Me
NH
N
BF3
f1 (ppm)

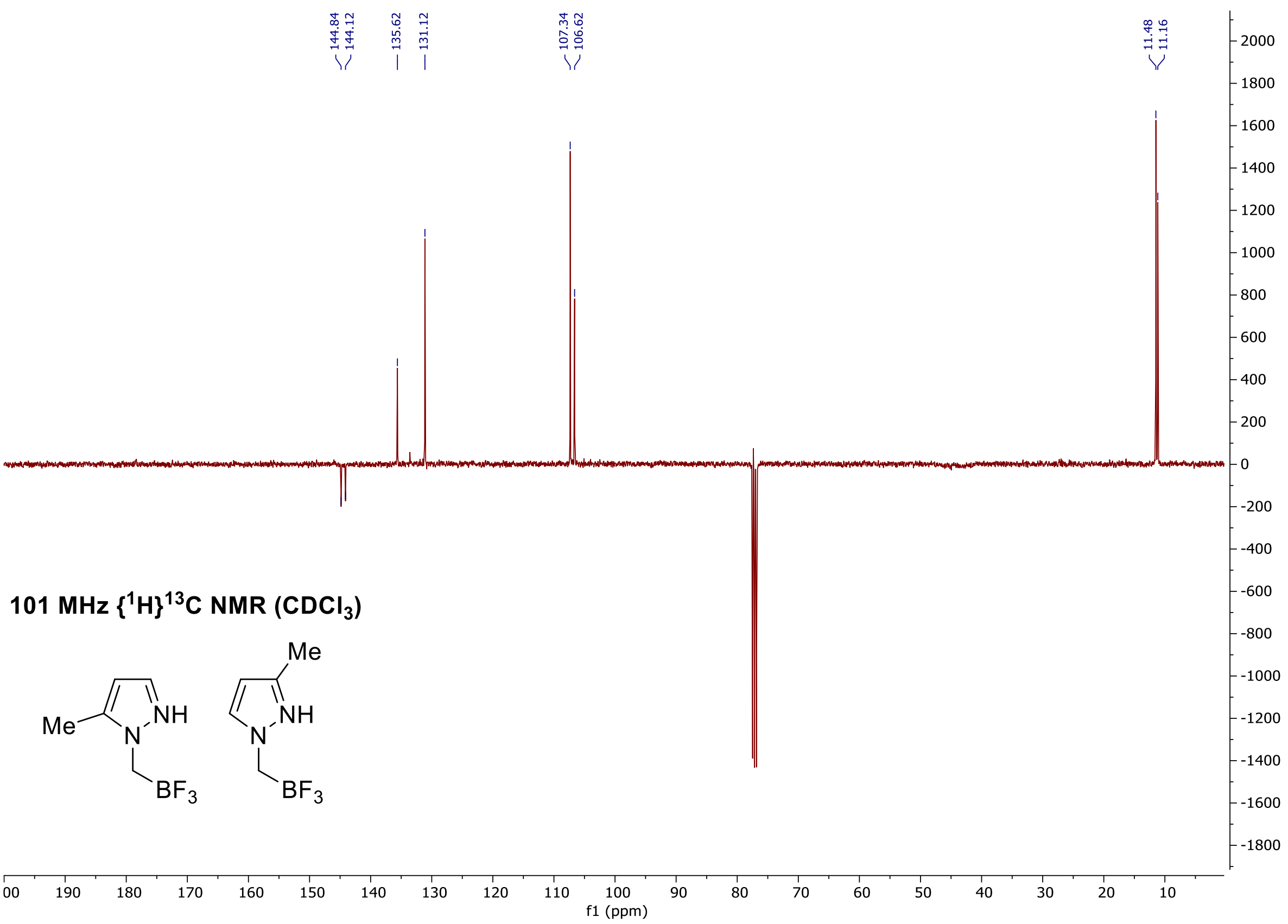

101 MHz {1H}13C NMR (CDCl3)
Me
NH
N
BF3
f1 (ppm)

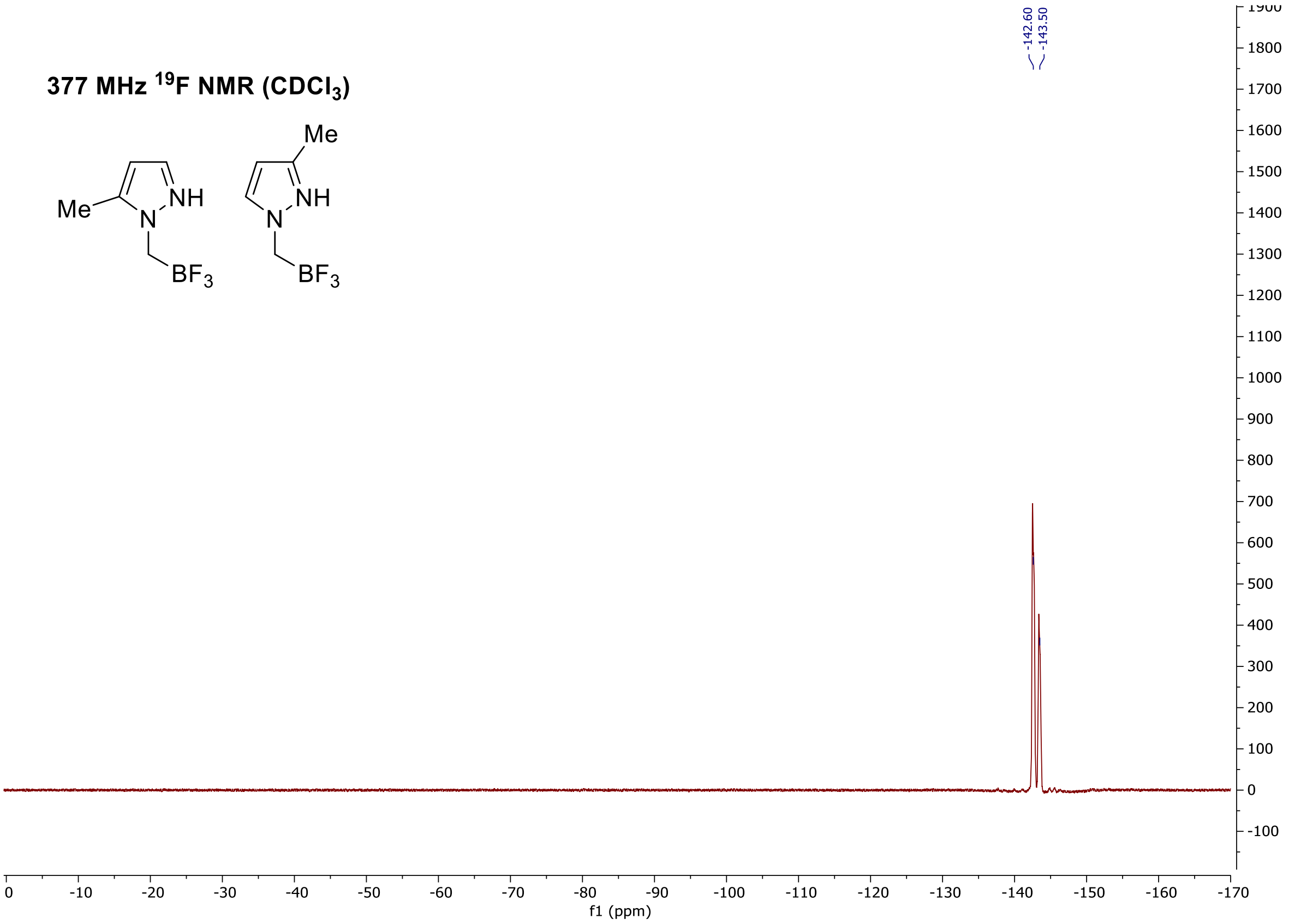
377 MHz 19F NMR (CDCl3)
Me
NH
N
BF3
-142.60
-143.50
f1 (ppm)

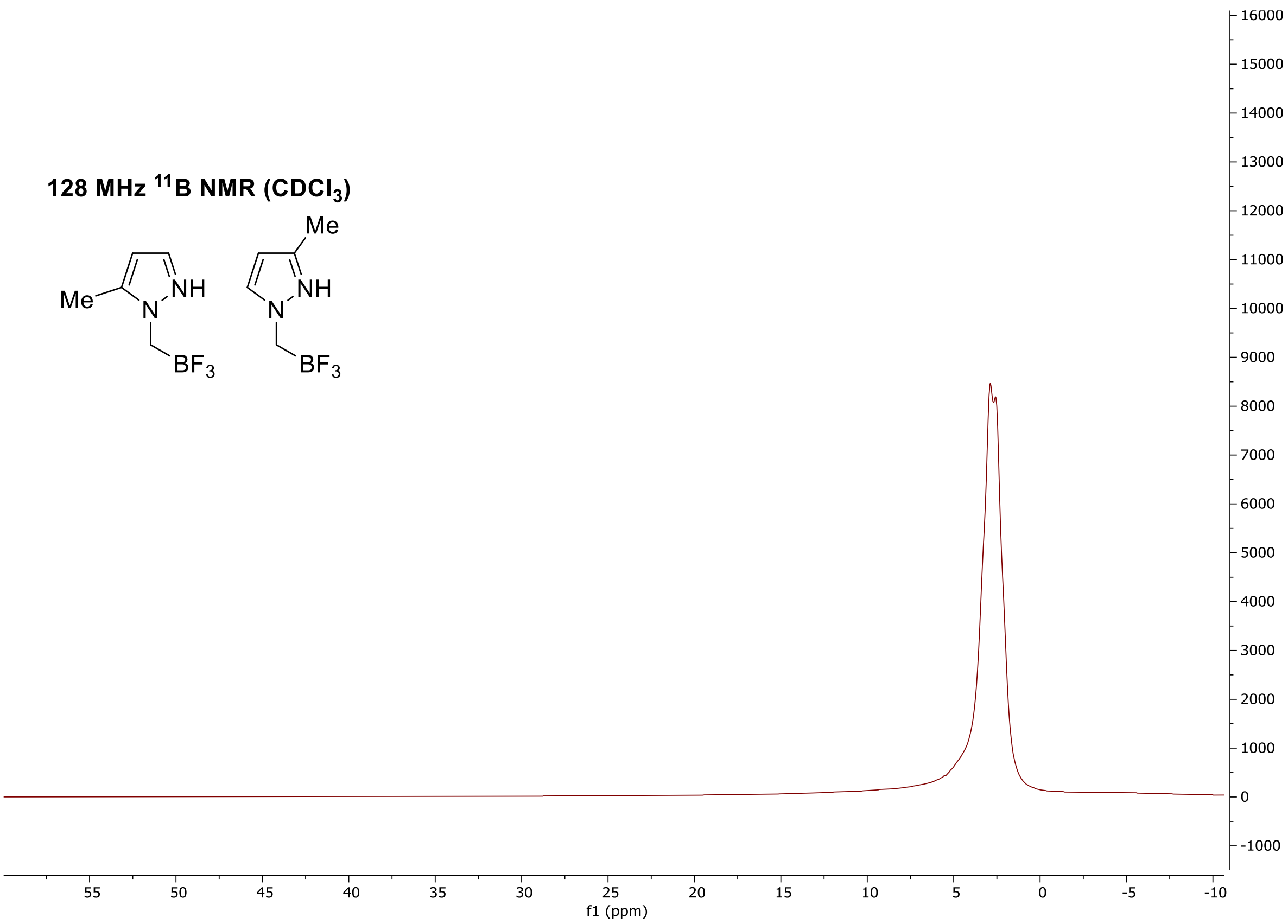
128 MHz 11B NMR (CDCl3)
Me
NH
N
BF3
f1 (ppm)

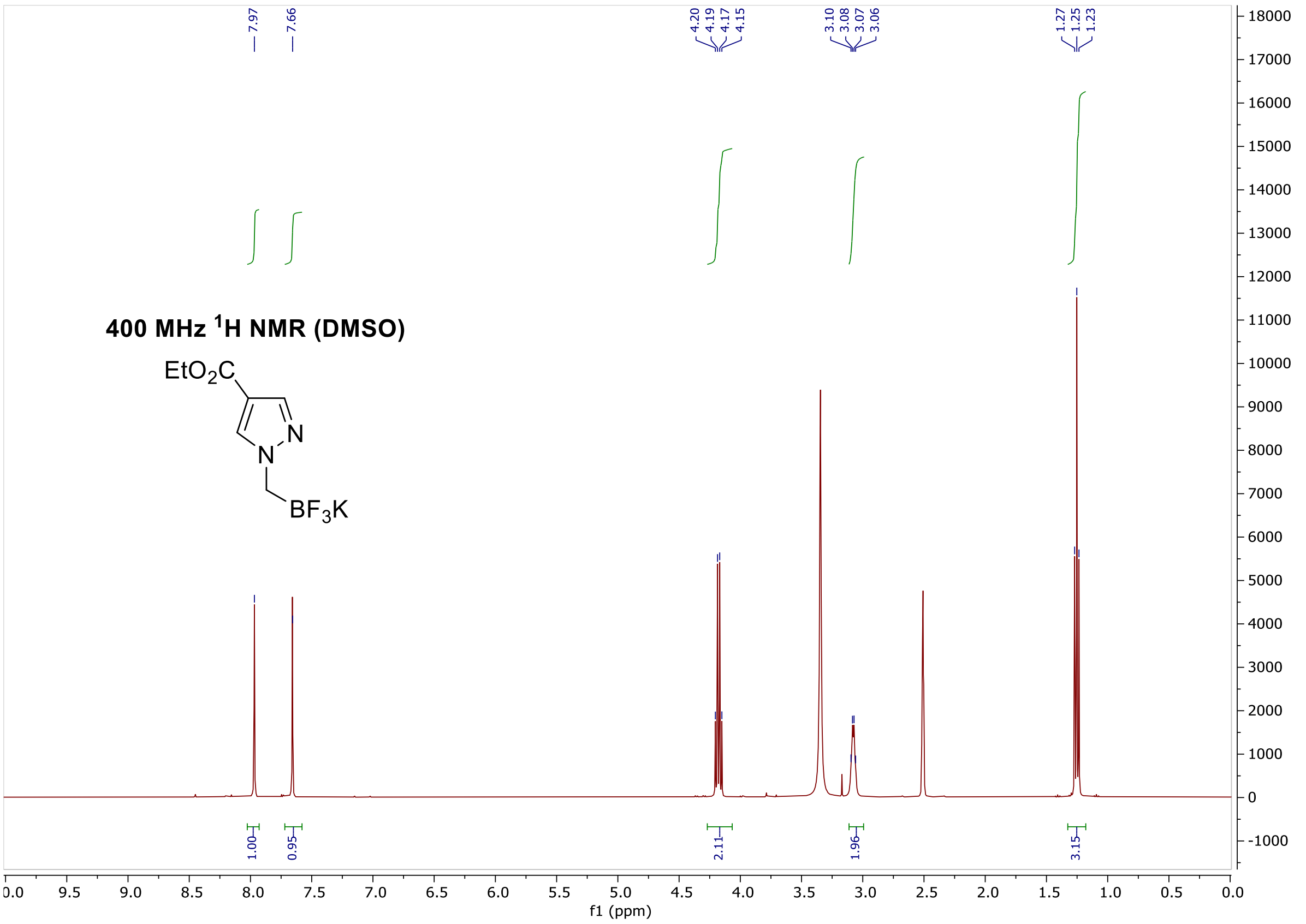
400 MHz ^{1}H NMR (DMSO)
EtO2C
N
N
BF3K
7.97
7.66
4.20
4.19
4.17
4.15
3.10
3.08
3.07
3.06
1.27
1.25
1.23
1.00
0.95
2.11
1.96
3.15
f1 (ppm)

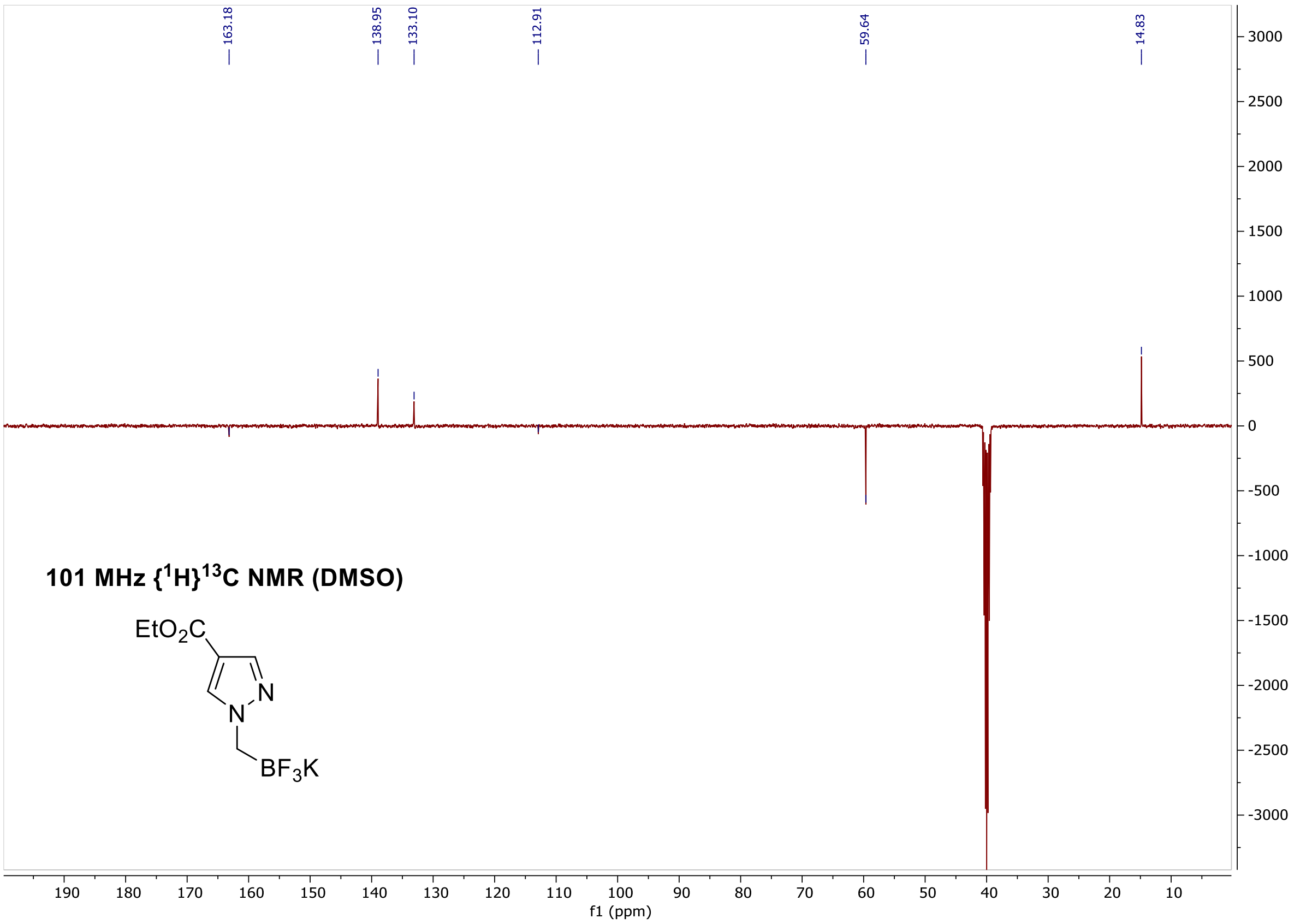
101 MHz {^{1}H}^{13}C NMR (DMSO)
EtO2C
N
N
BF3K
163.18
138.95
133.10
112.91
59.64
14.83
f1 (ppm)

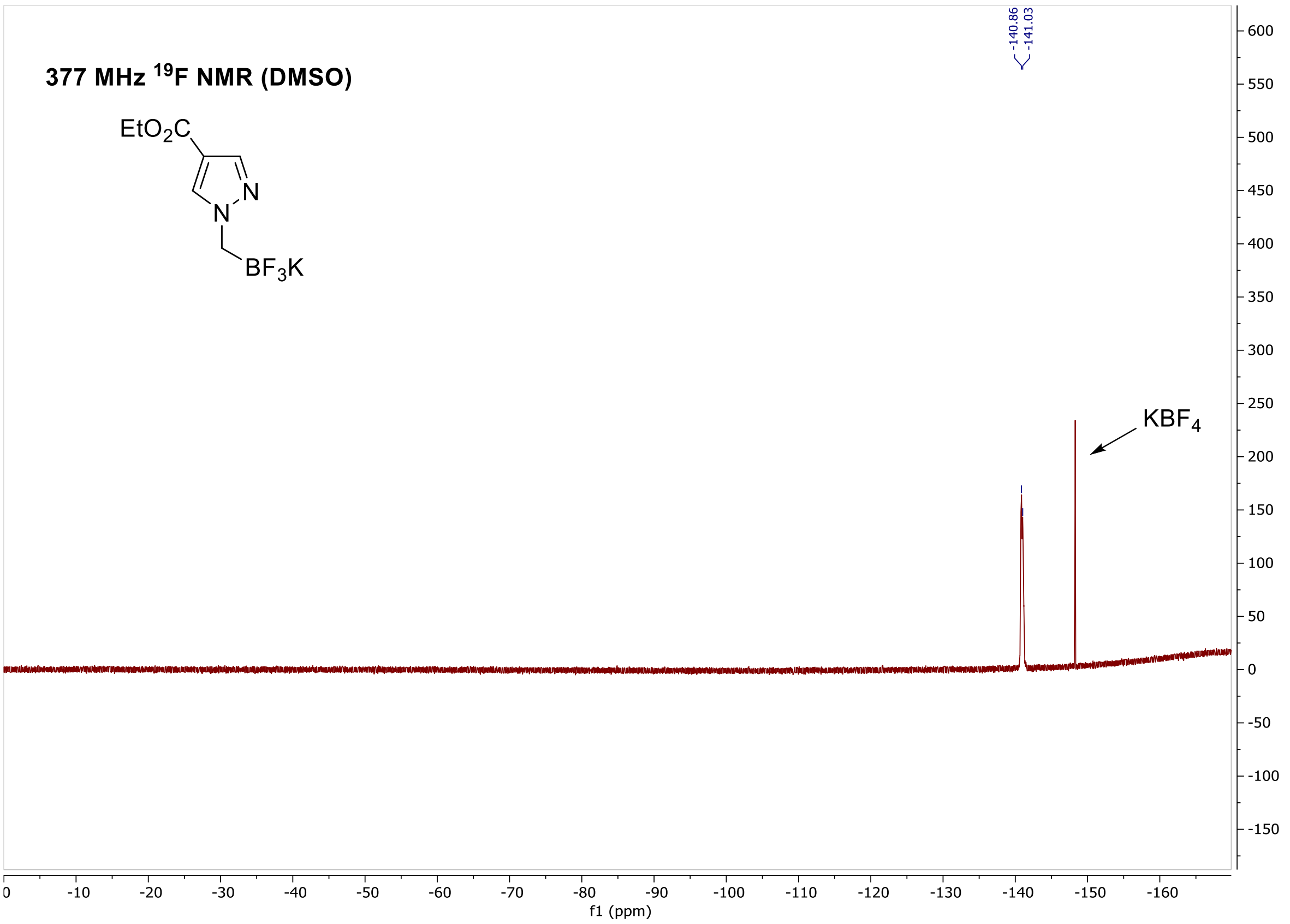
377 MHz 19F NMR (DMSO)
EtO2C
N
N
BF3K
KBF4
-140.86
-141.03
f1 (ppm)

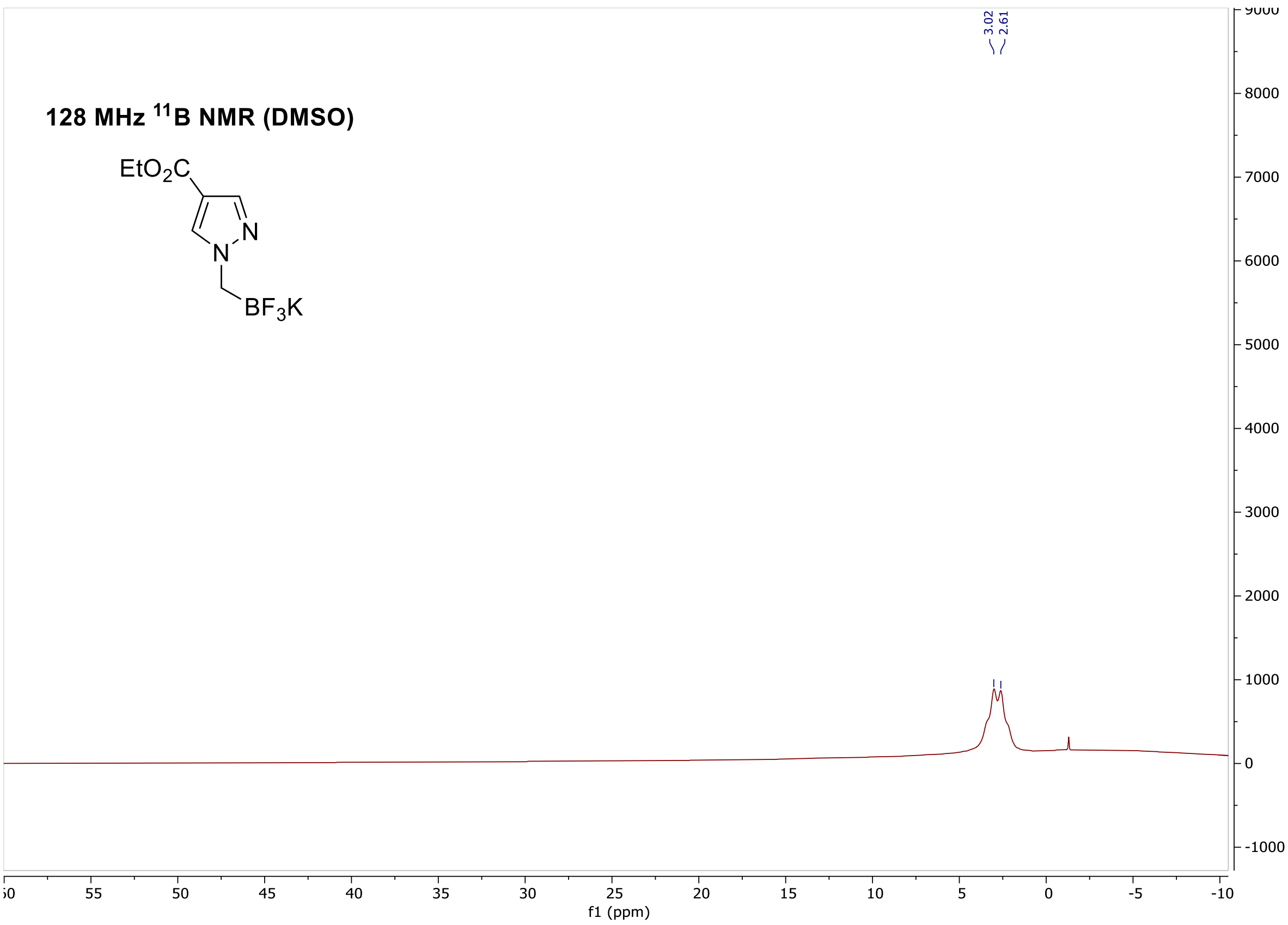
128 MHz 11B NMR (DMSO)
EtO2C
N
N
BF3K
3.02
2.61
f1 (ppm)

400 MHz ^1H NMR (DMSO)

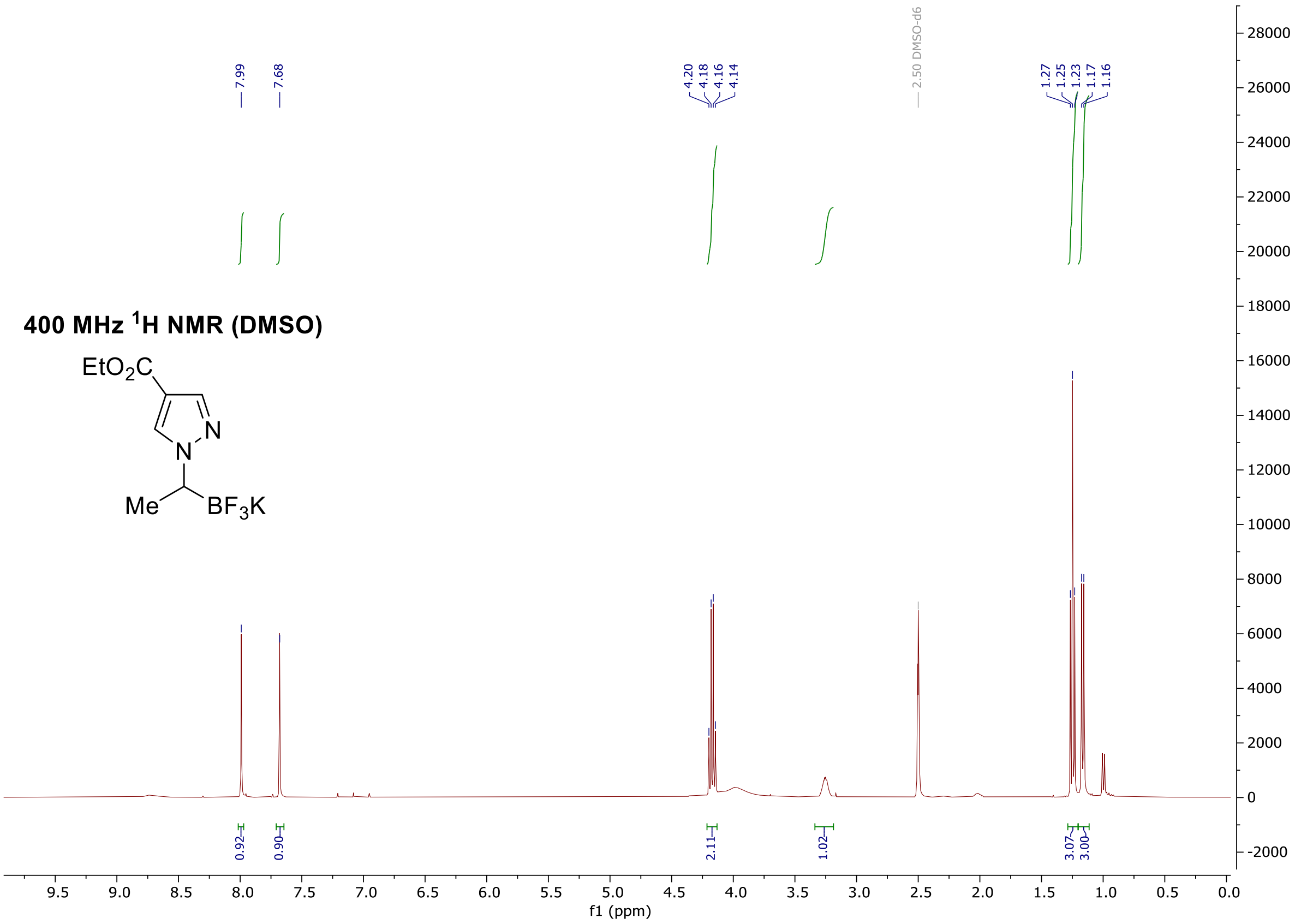

101 MHz {^1H}^{13}C NMR (DMSO)

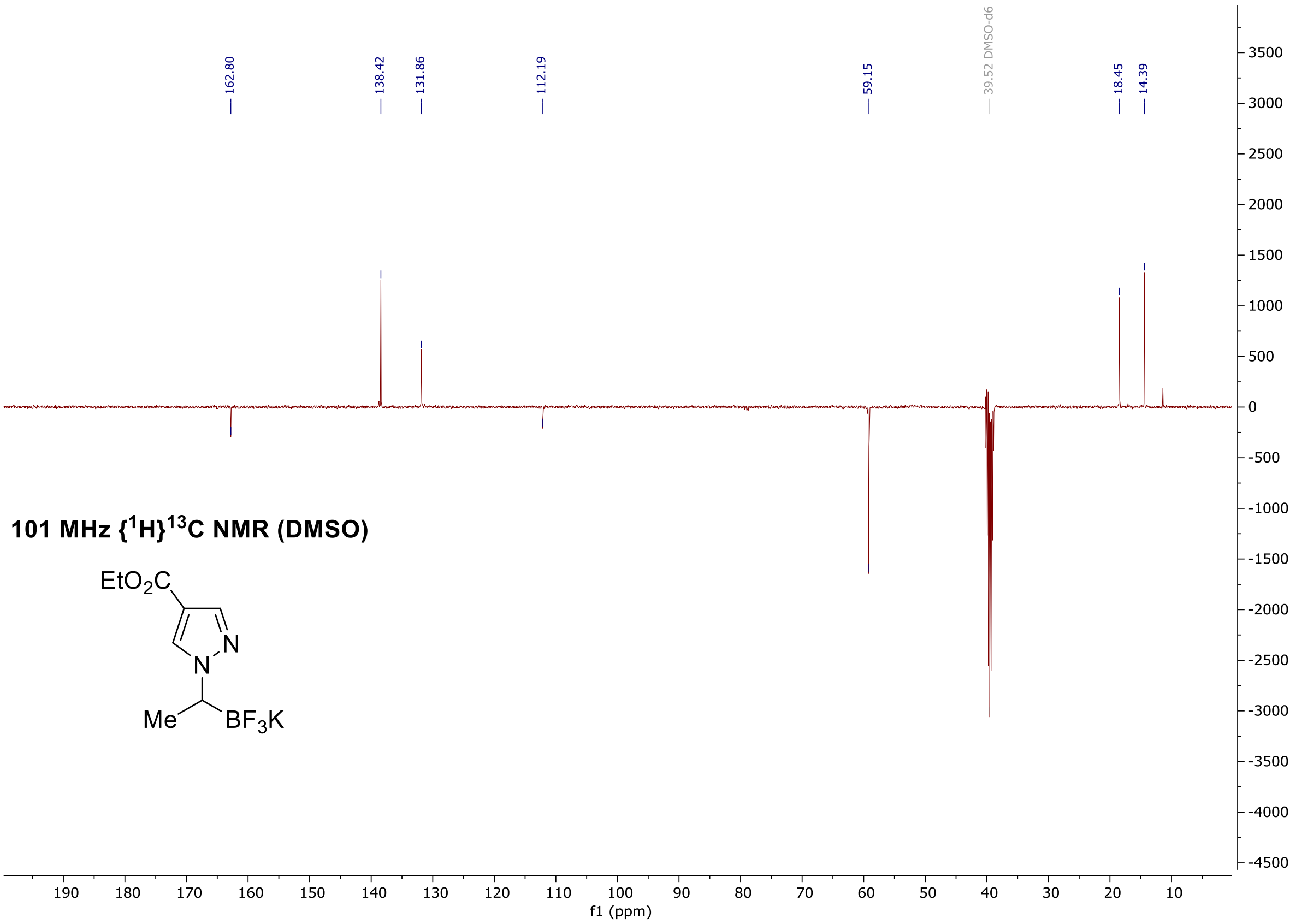

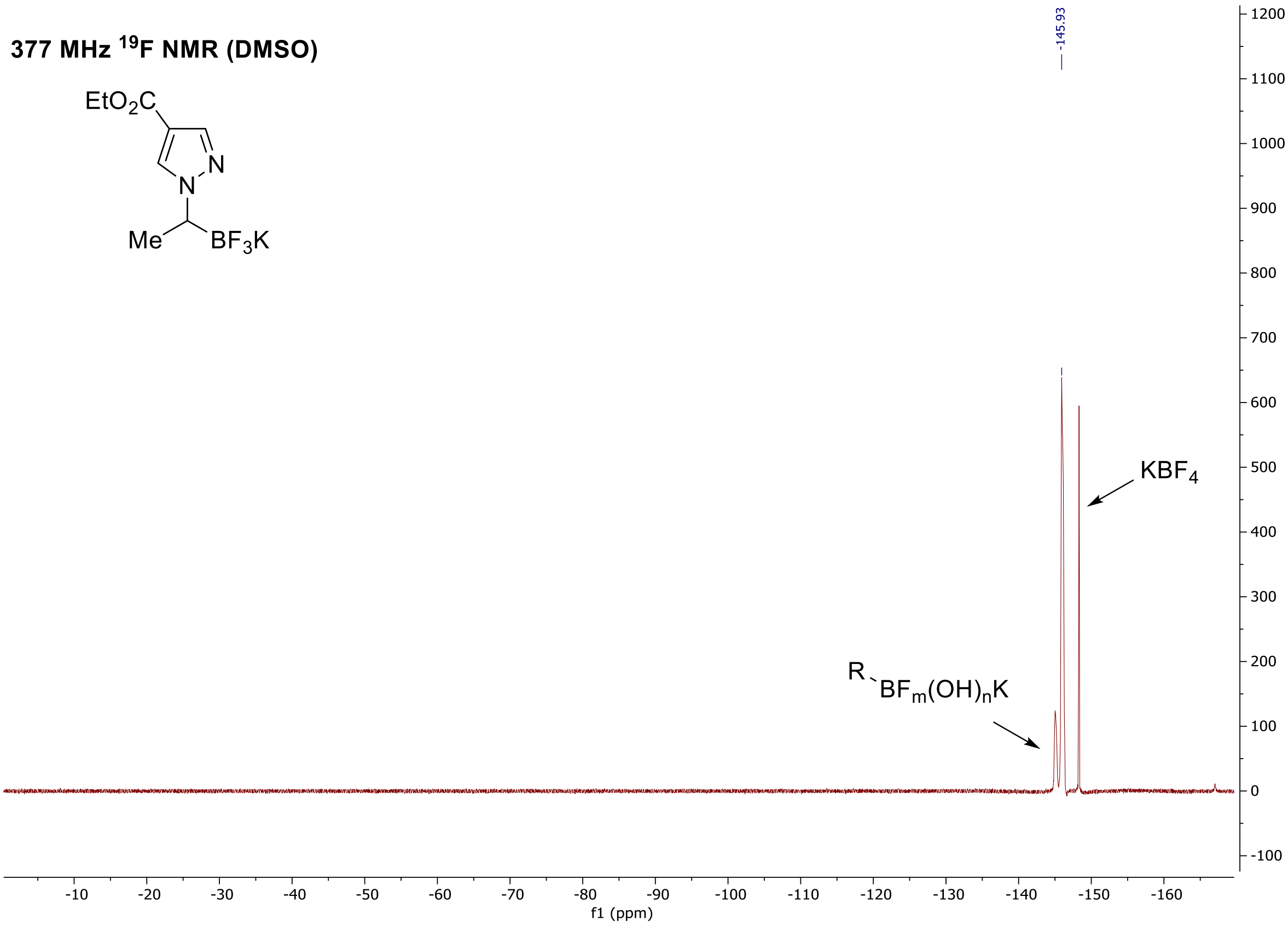
377 MHz 19F NMR (DMSO)
EtO2C
N
N
Me
BF3K
-145.93
KBF4
R
BFm(OH)nK
f1 (ppm)

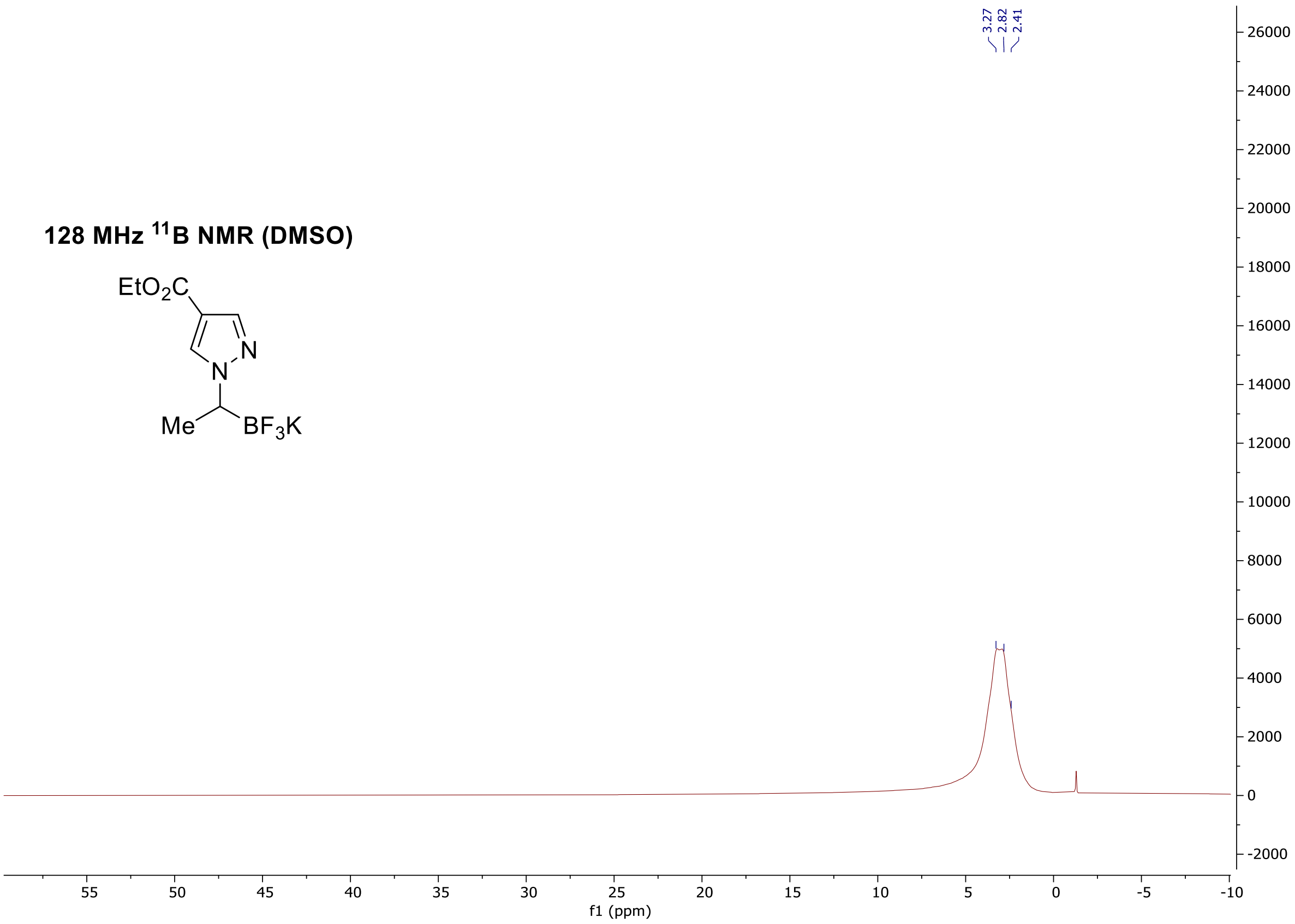
128 MHz 11B NMR (DMSO)
EtO2C
N
N
Me
BF3K
3.27
2.82
2.41
f1 (ppm)

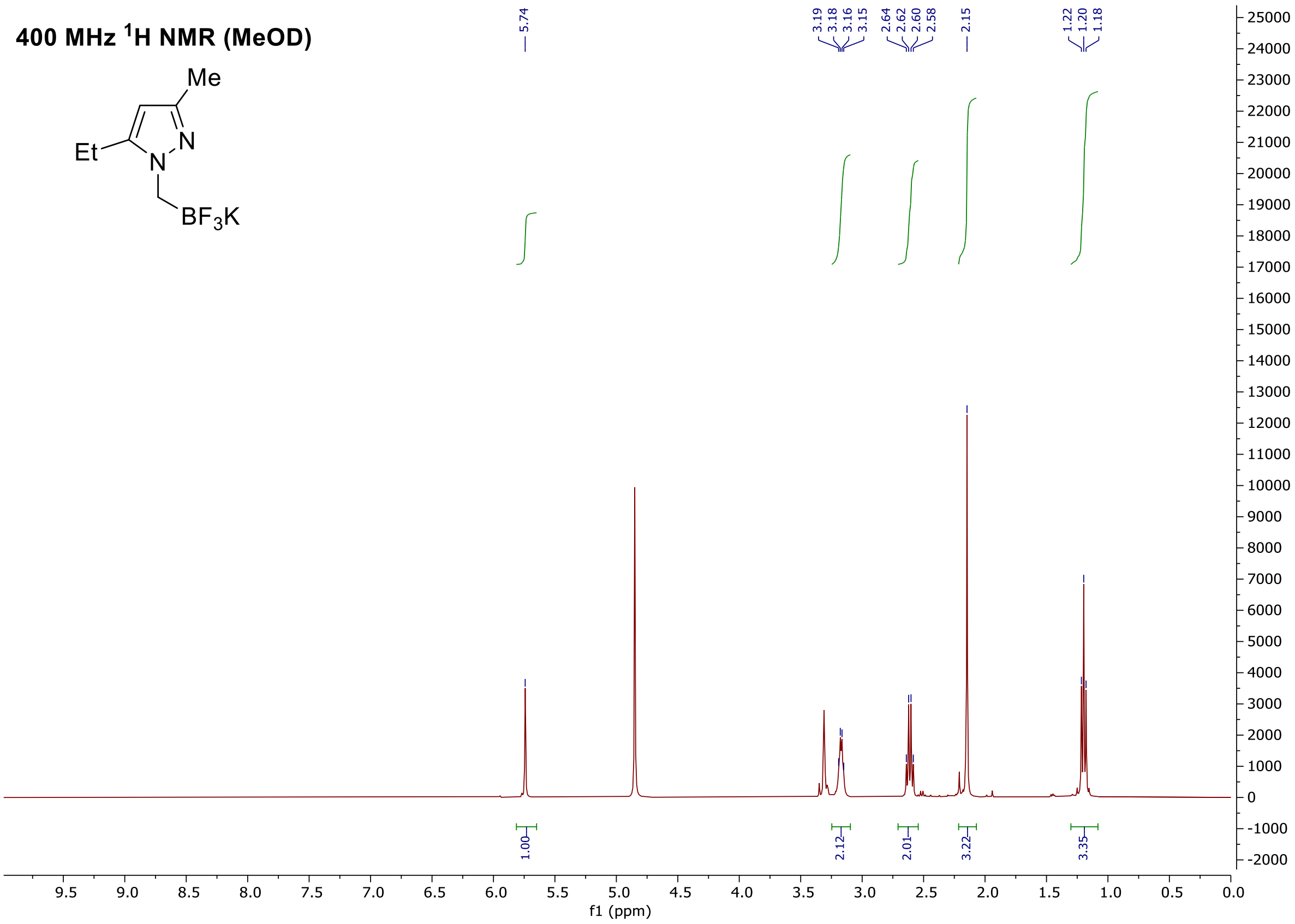
400 MHz ¹H NMR (MeOD)
Me
Et
N
N
BF3K
5.74
3.19
3.18
3.16
3.15
2.64
2.62
2.60
2.58
2.15
1.22
1.20
1.18
1.00
2.12
2.01
3.22
3.35
f1 (ppm)

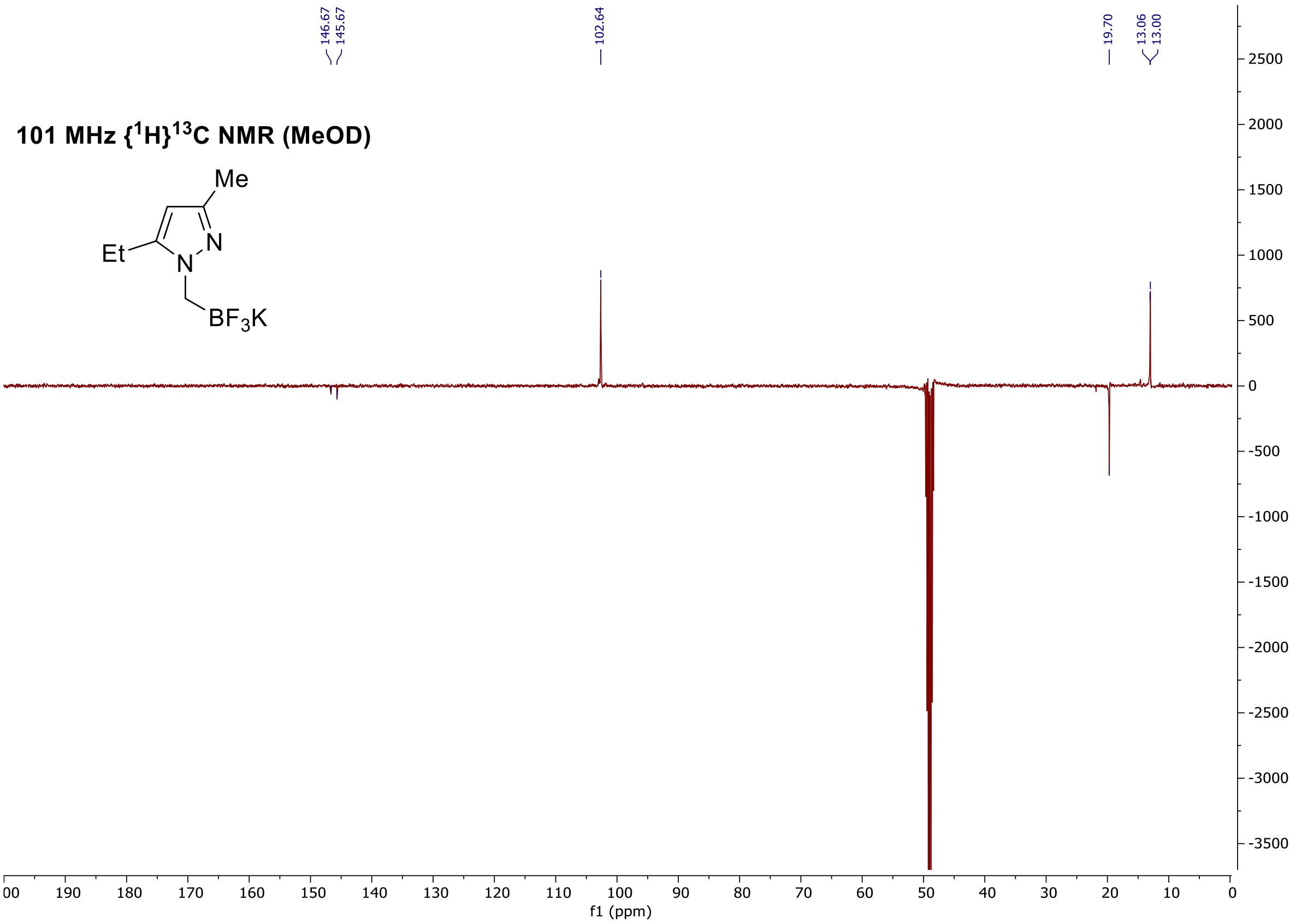
101 MHz {¹H}¹³C NMR (MeOD)
Me
Et
N
N
BF3K
146.67
145.67
102.64
19.70
13.06
13.00
f1 (ppm)

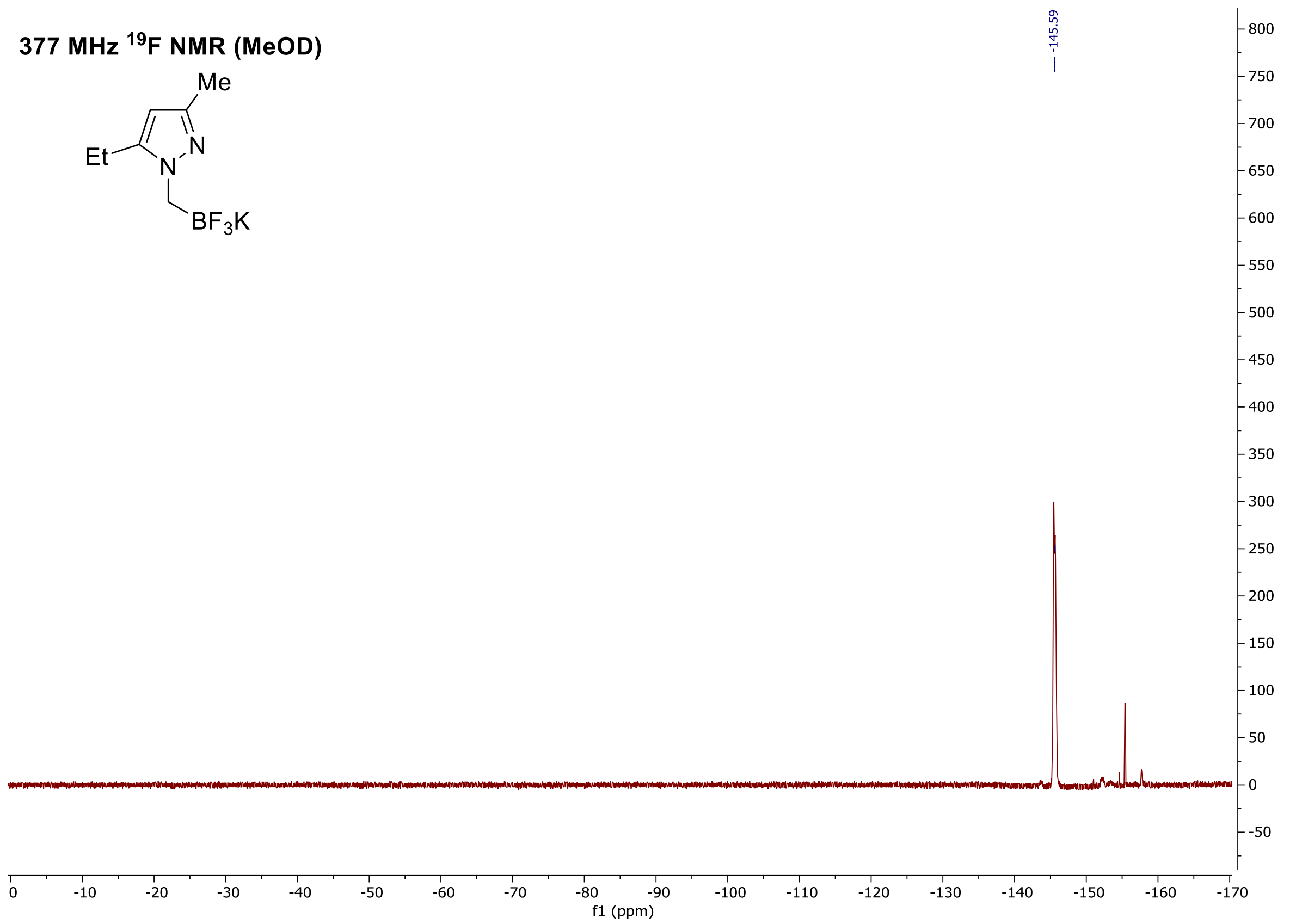
377 MHz ^{19}F NMR (MeOD)
Me
N
Et
N
BF_3K
-145.59
f1 (ppm)

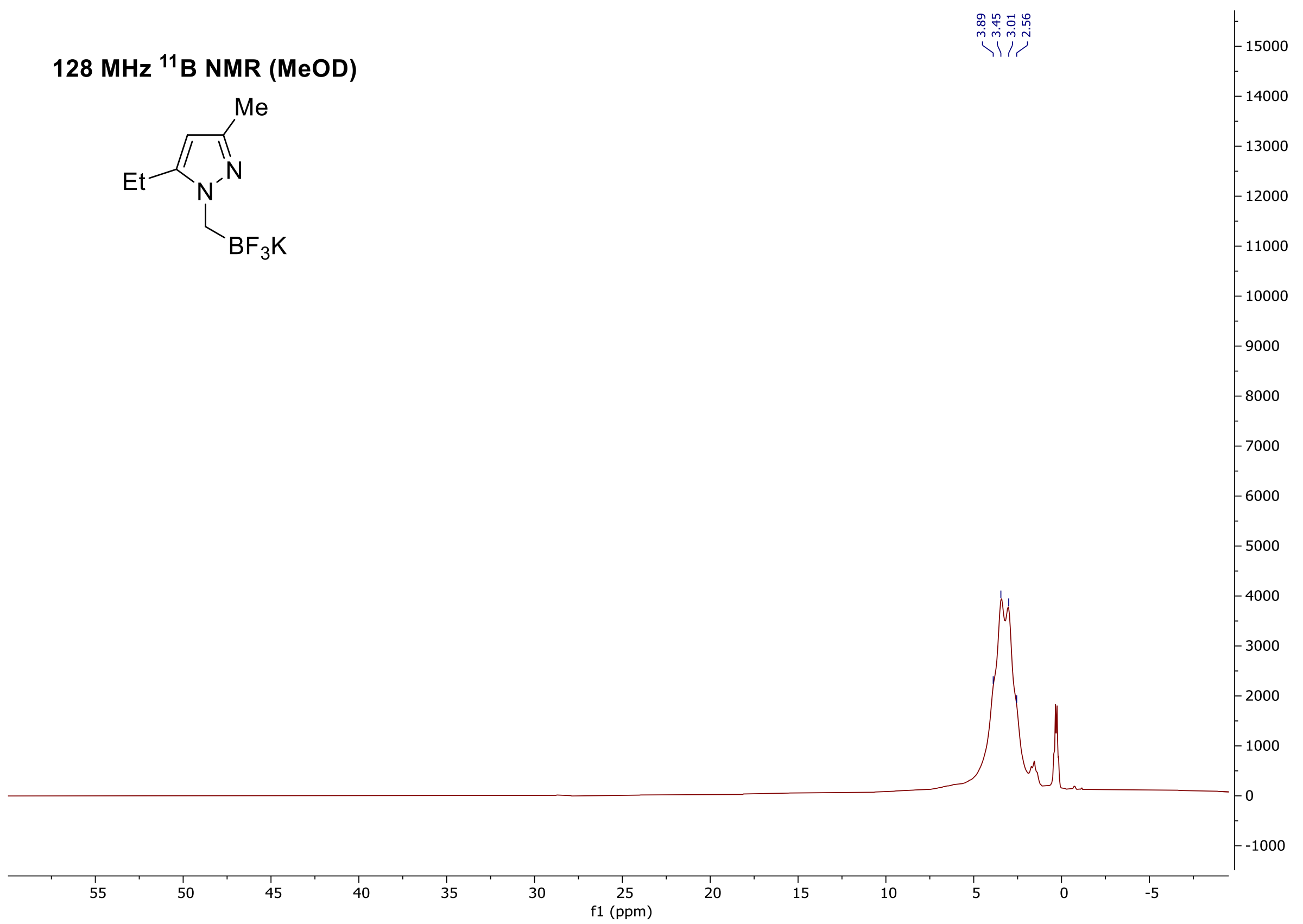
128 MHz ^{11}B NMR (MeOD)
Me
N
Et
N
BF_3K
3.89
3.45
3.01
2.56
f1 (ppm)

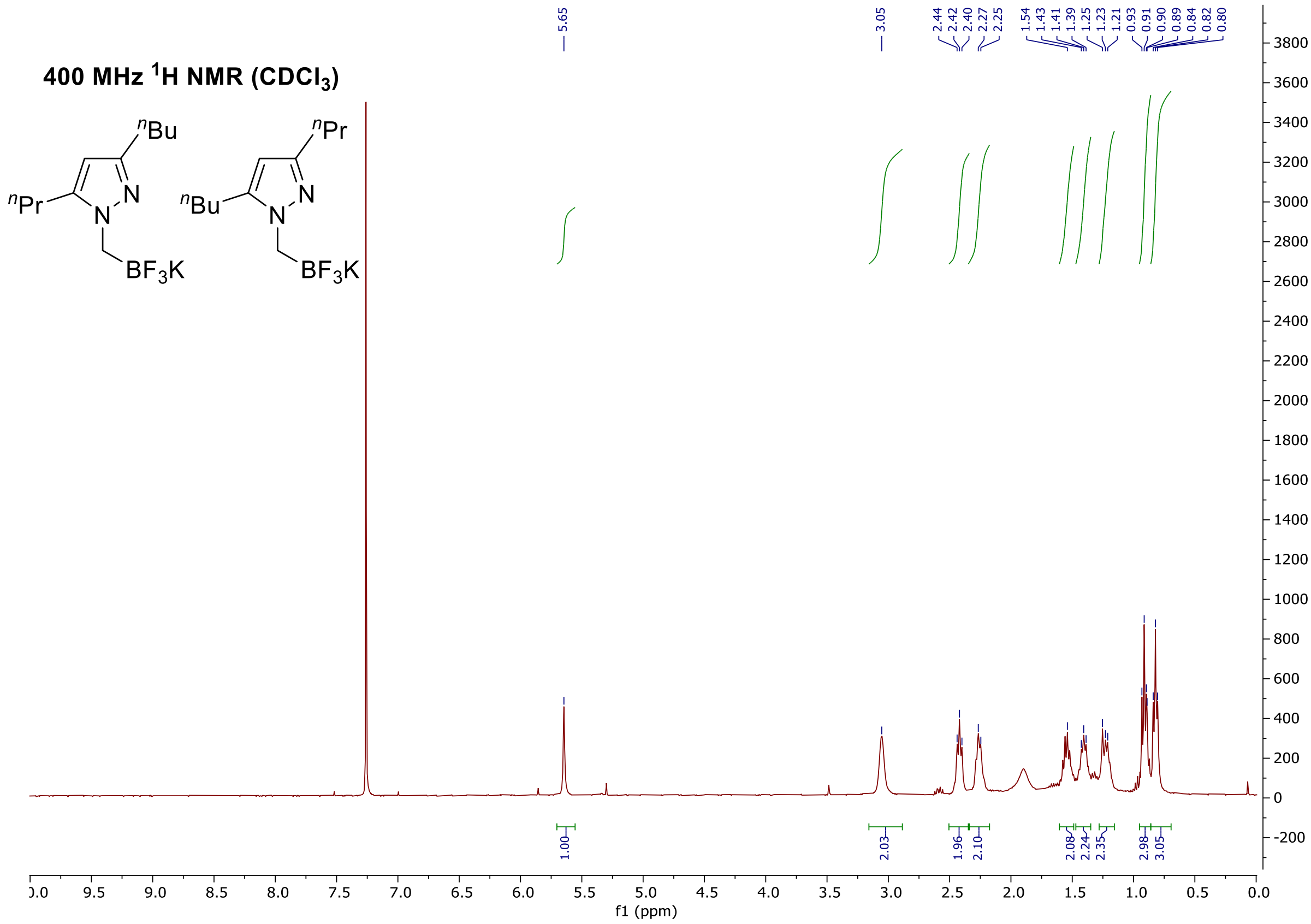
400 MHz ^{1}H NMR ($CDCl_3$)
^{n}Bu
^{n}Pr
N
N
BF_3K
^{n}Pr
^{n}Bu
5.65
3.05
2.44 2.42 2.40 2.27 2.25
1.54 1.43 1.41 1.39 1.25 1.23 1.21 0.93 0.91 0.90 0.89 0.84 0.82 0.80
1.00
2.03
1.96 2.10
2.08 2.24 2.35
2.98 3.05
f1 (ppm)

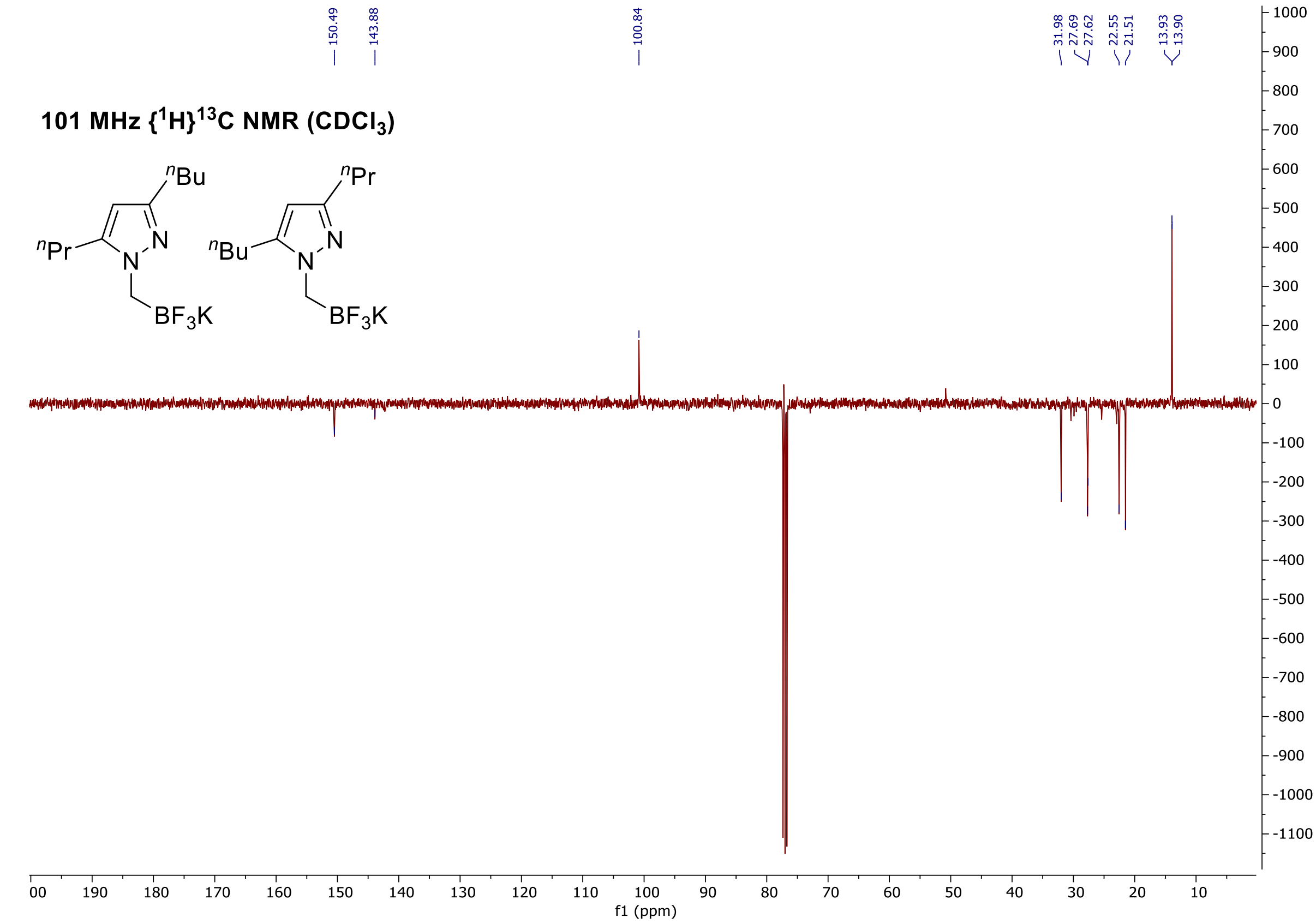
101 MHz {^{1}H}^{13}C NMR ($CDCl_3$)
^{n}Bu
^{n}Pr
N
N
BF_3K
^{n}Pr
^{n}Bu
150.49
143.88
100.84
31.98 27.69 27.62 22.55 21.51 13.93 13.90
f1 (ppm)

377 MHz ^{19}F NMR ($CDCl_3$)

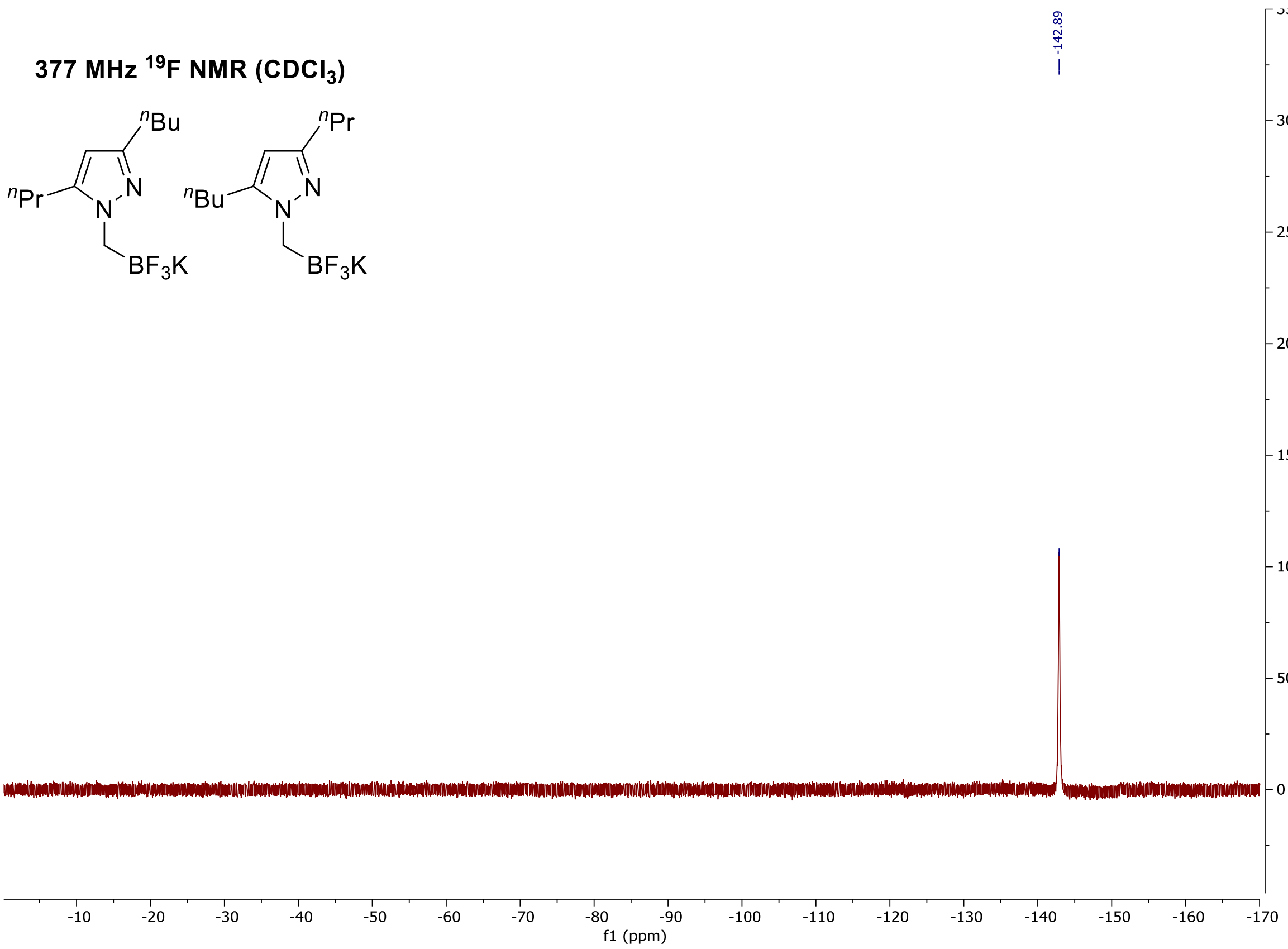

128 MHz ^{11}B NMR ($CDCl_3$)

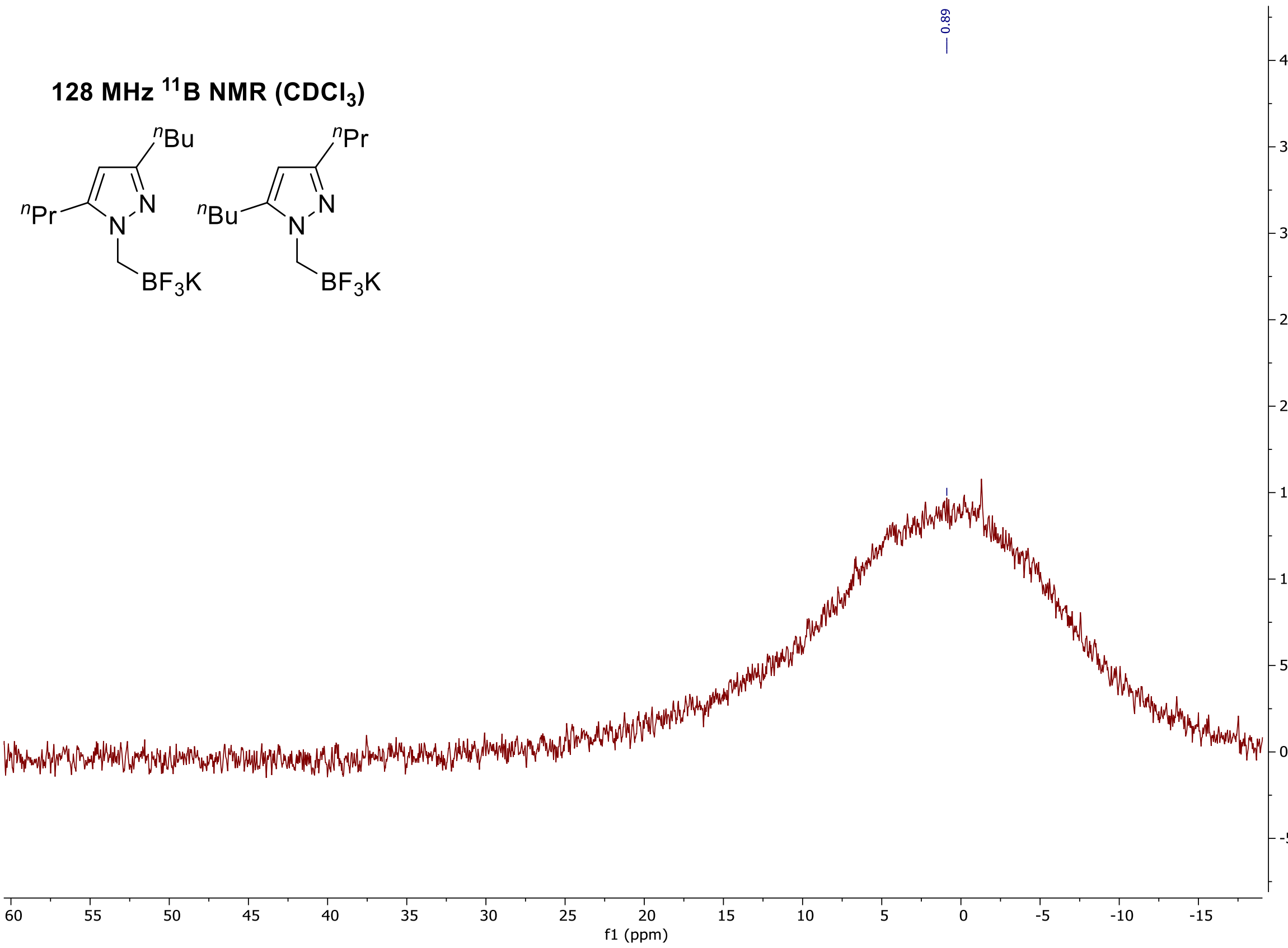

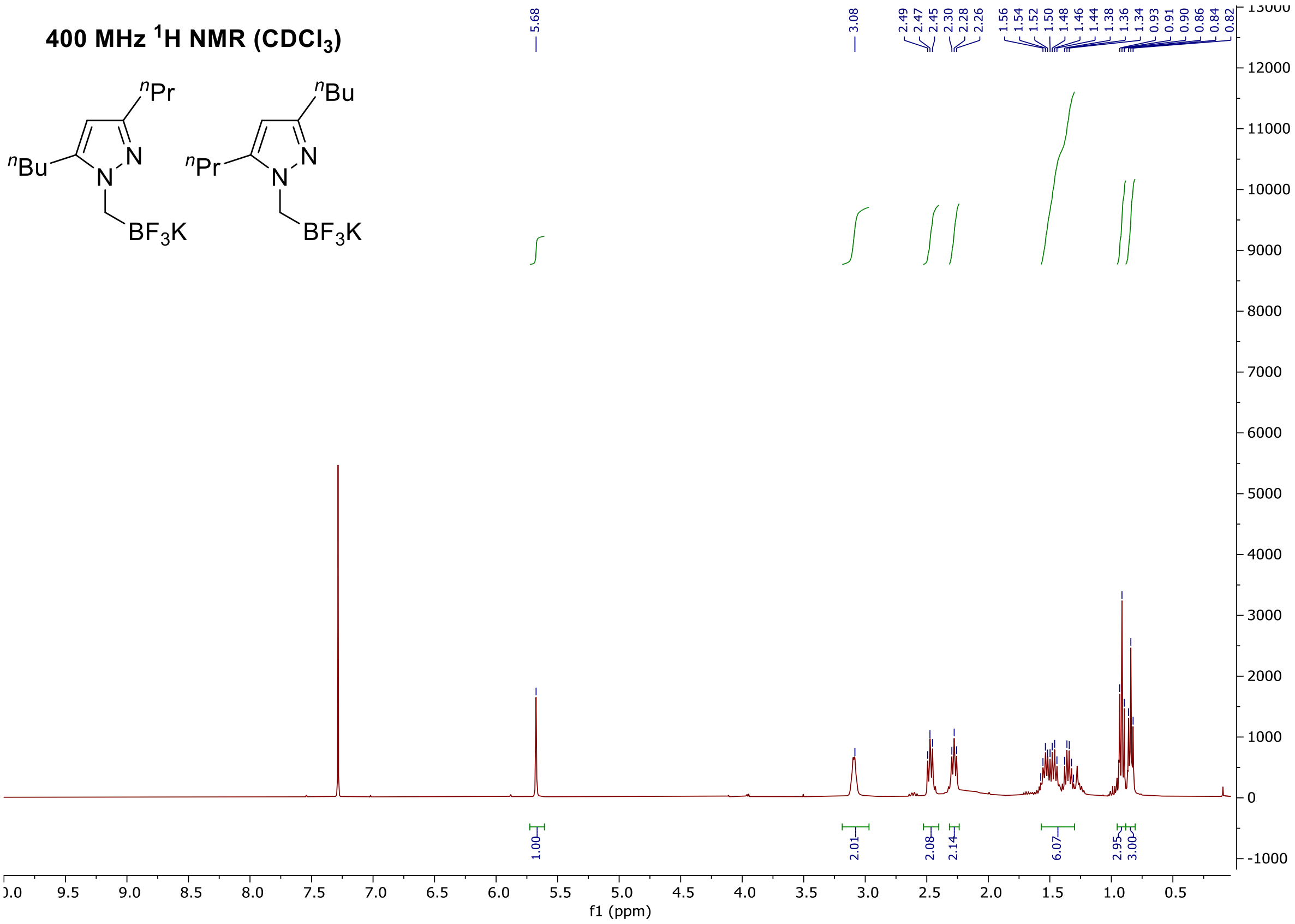
400 MHz ^{1}H NMR ($CDCl_3$)
5.68
3.08
2.49 2.47 2.45 2.30 2.28 2.26
1.56 1.54 1.52 1.50 1.48 1.46 1.44 1.38 1.36 1.34 0.93 0.91 0.90 0.86 0.84 0.82
1.00
2.01
2.08
2.14
6.07
2.95
3.00
f1 (ppm)

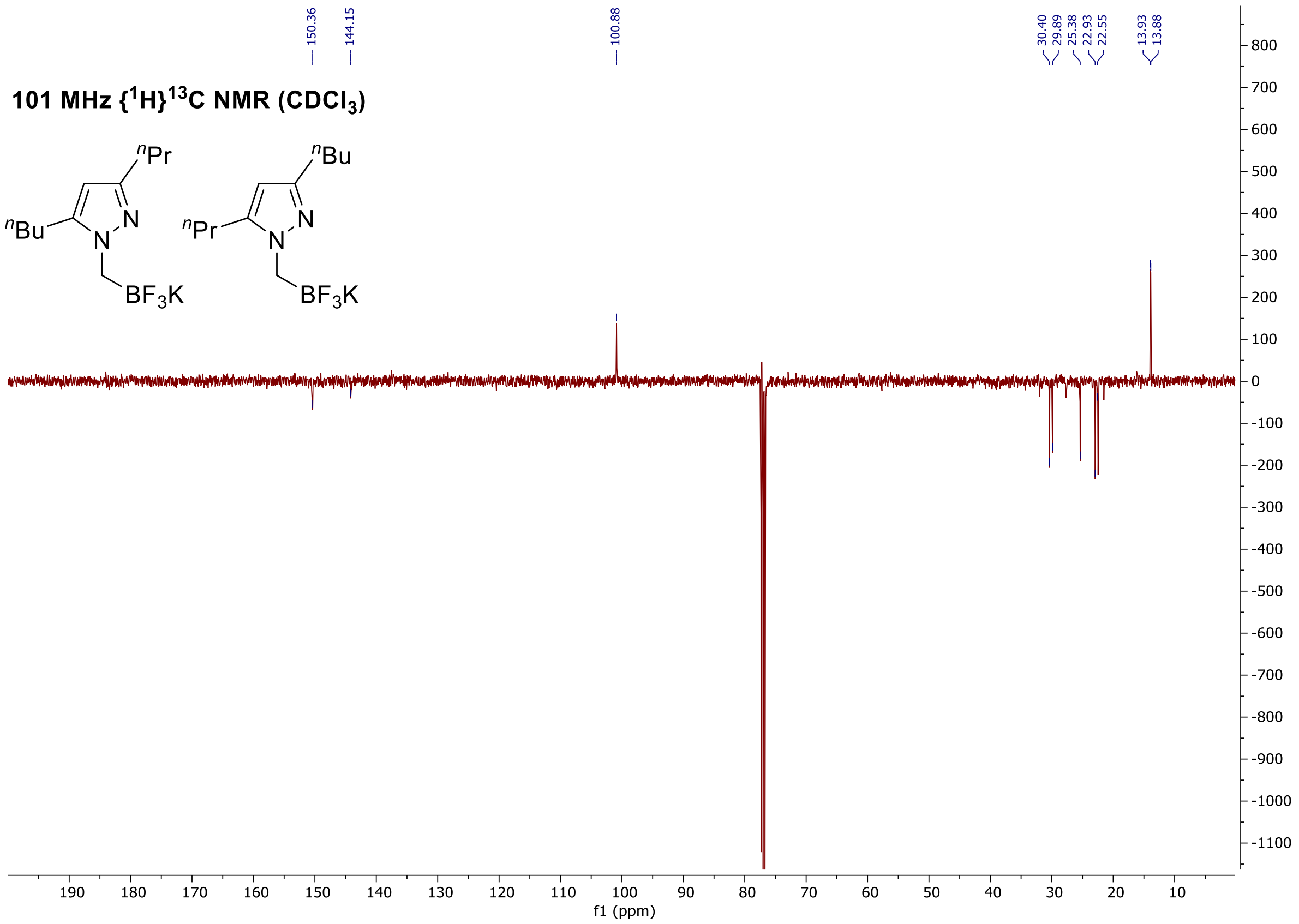
101 MHz {^{1}H}^{13}C NMR ($CDCl_3$)
150.36
144.15
100.88
30.40 29.89 25.38 22.93 22.55
13.93 13.88
f1 (ppm)

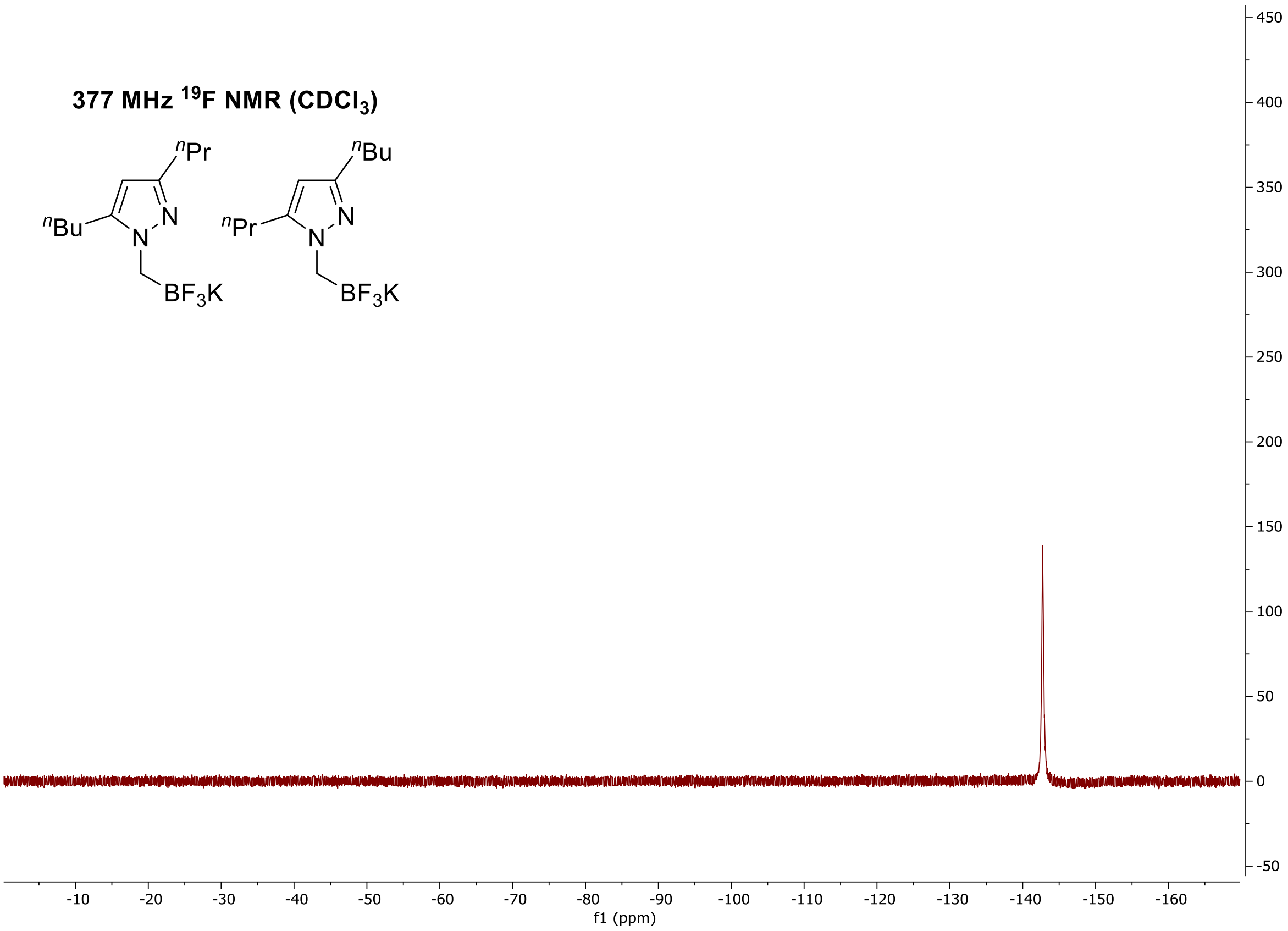
377 MHz 19F NMR (CDCl3)
nPr
nBu
N
N
BF3K
nBu
nPr
N
N
BF3K
f1 (ppm)

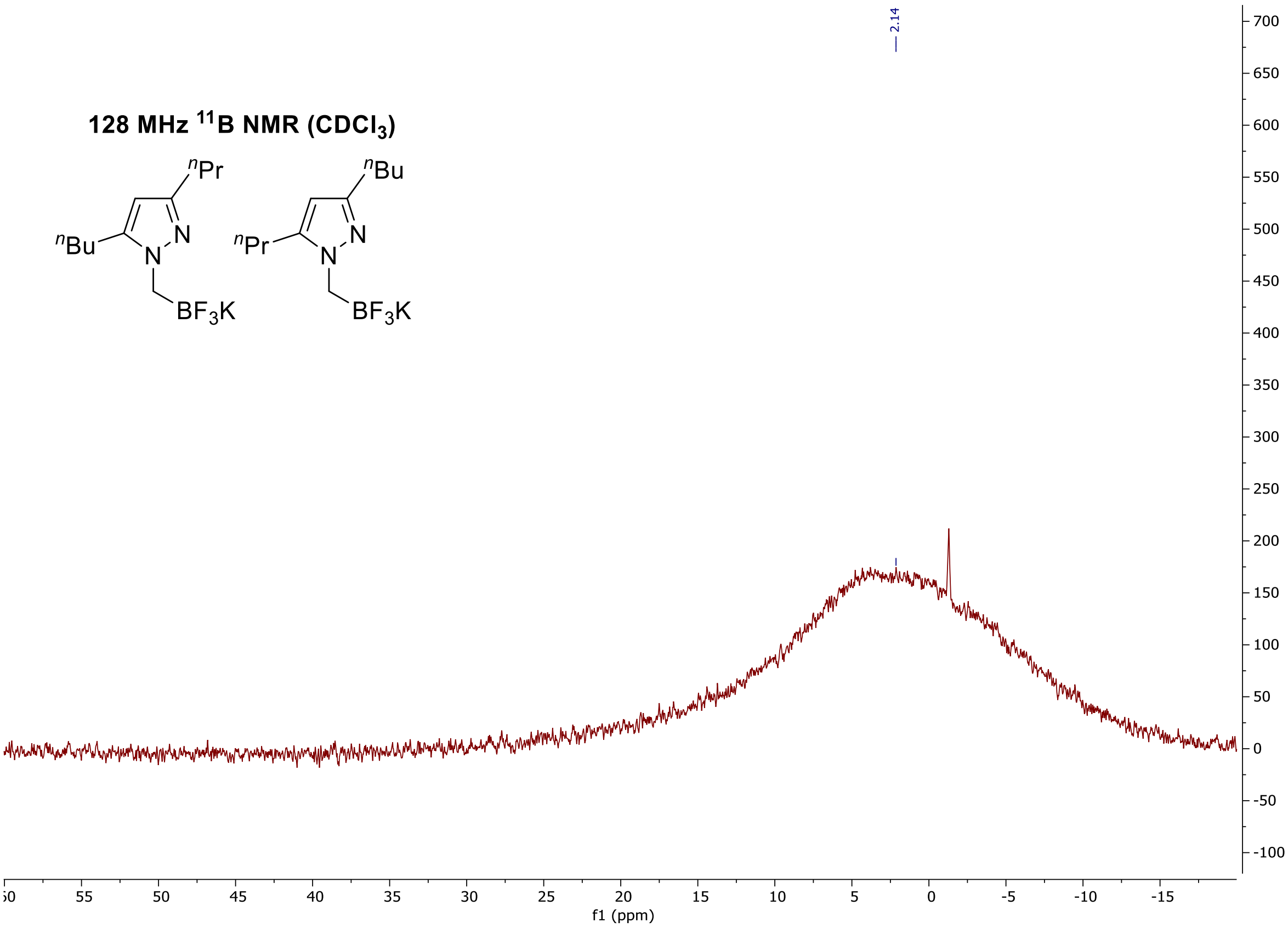
2.14
128 MHz 11B NMR (CDCl3)
nPr
nBu
N
N
BF3K
nBu
nPr
N
N
BF3K
f1 (ppm)

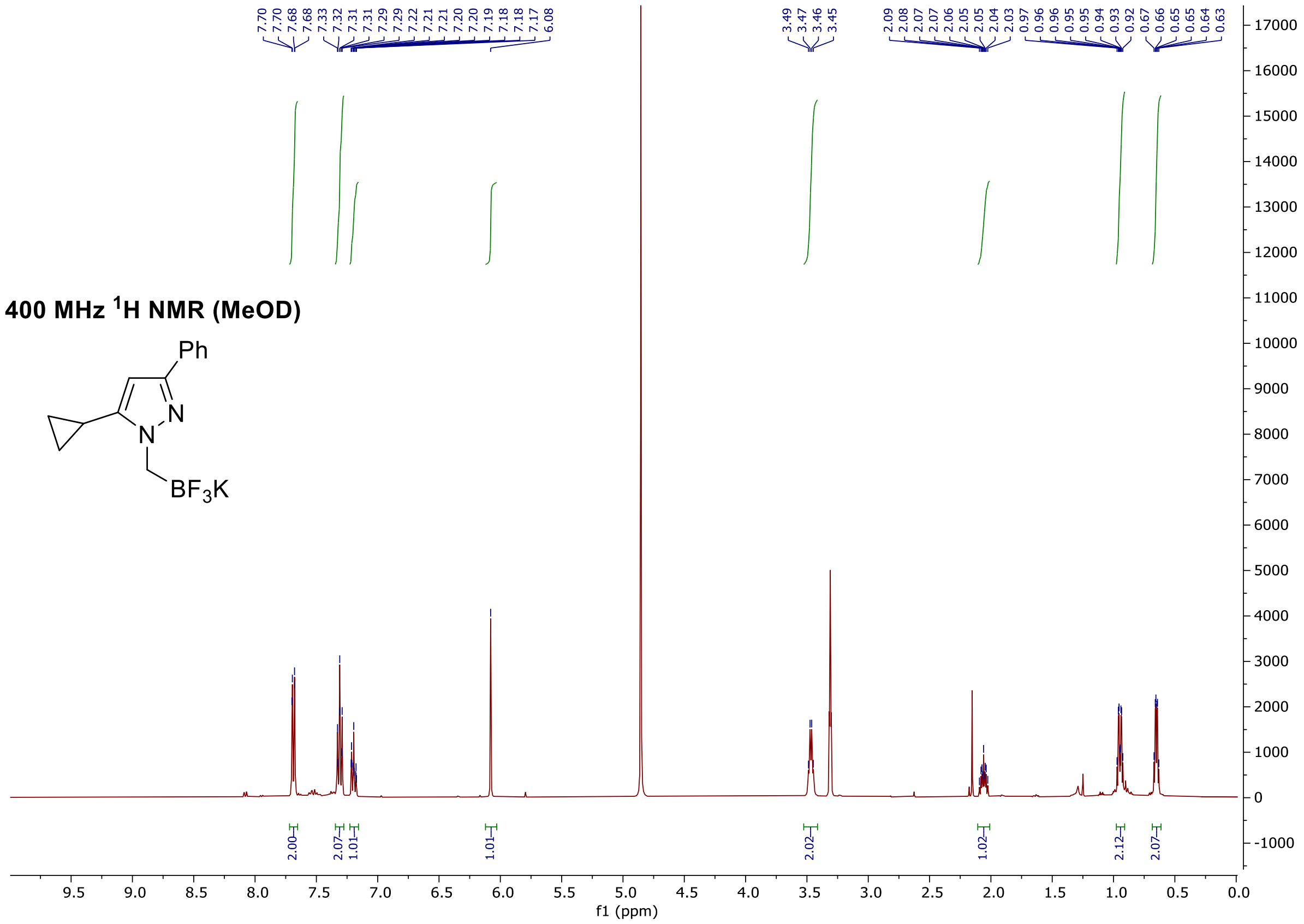
400 MHz ^{1}H NMR (MeOD)
Ph
N
N
BF$_3$K
f1 (ppm)

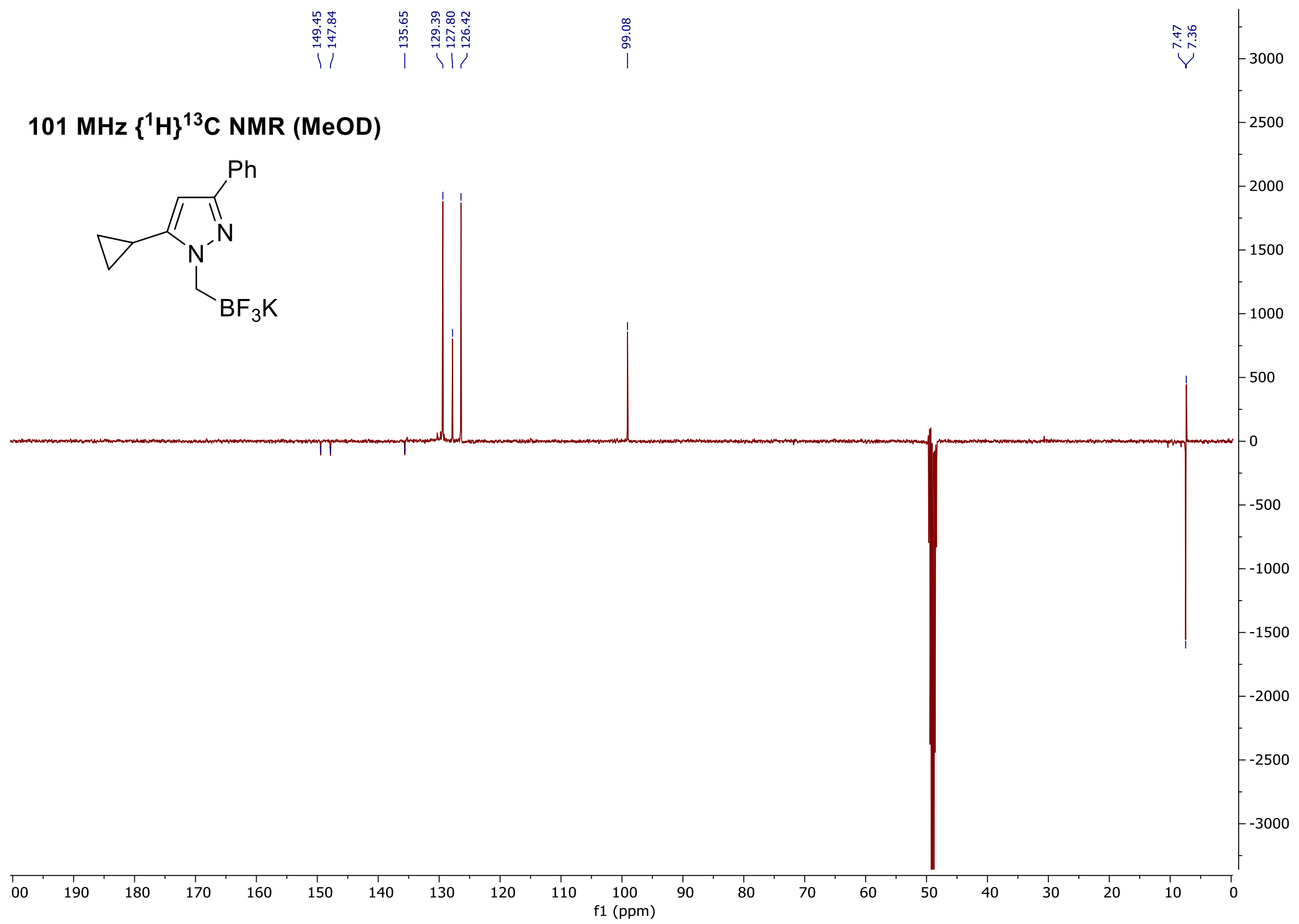
101 MHz {^{1}H}^{13}C NMR (MeOD)
Ph
N
N
BF$_3$K
f1 (ppm)

377 MHz ^{19}F NMR (MeOD)

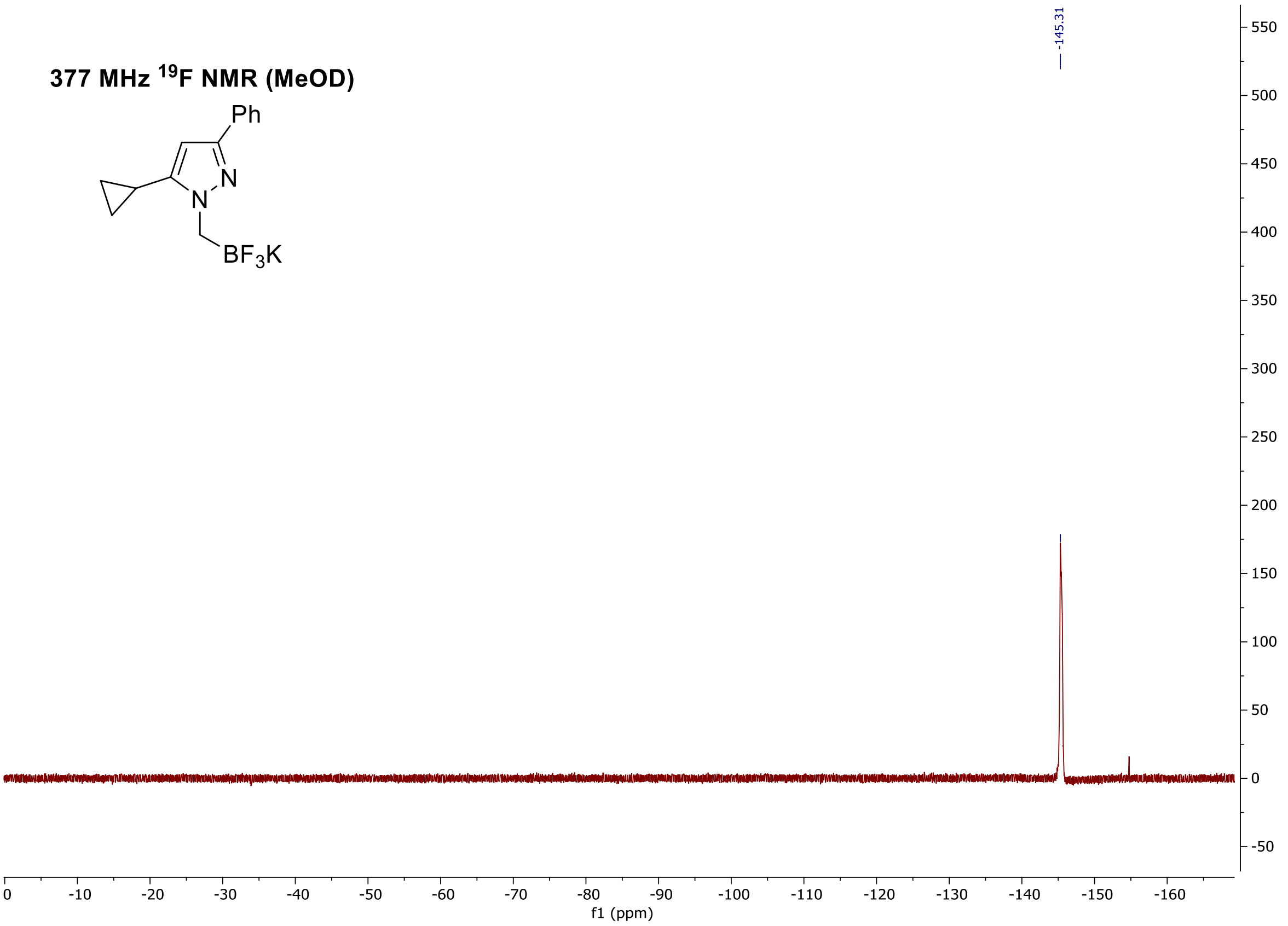

128 MHz ^{11}B NMR (MeOD)

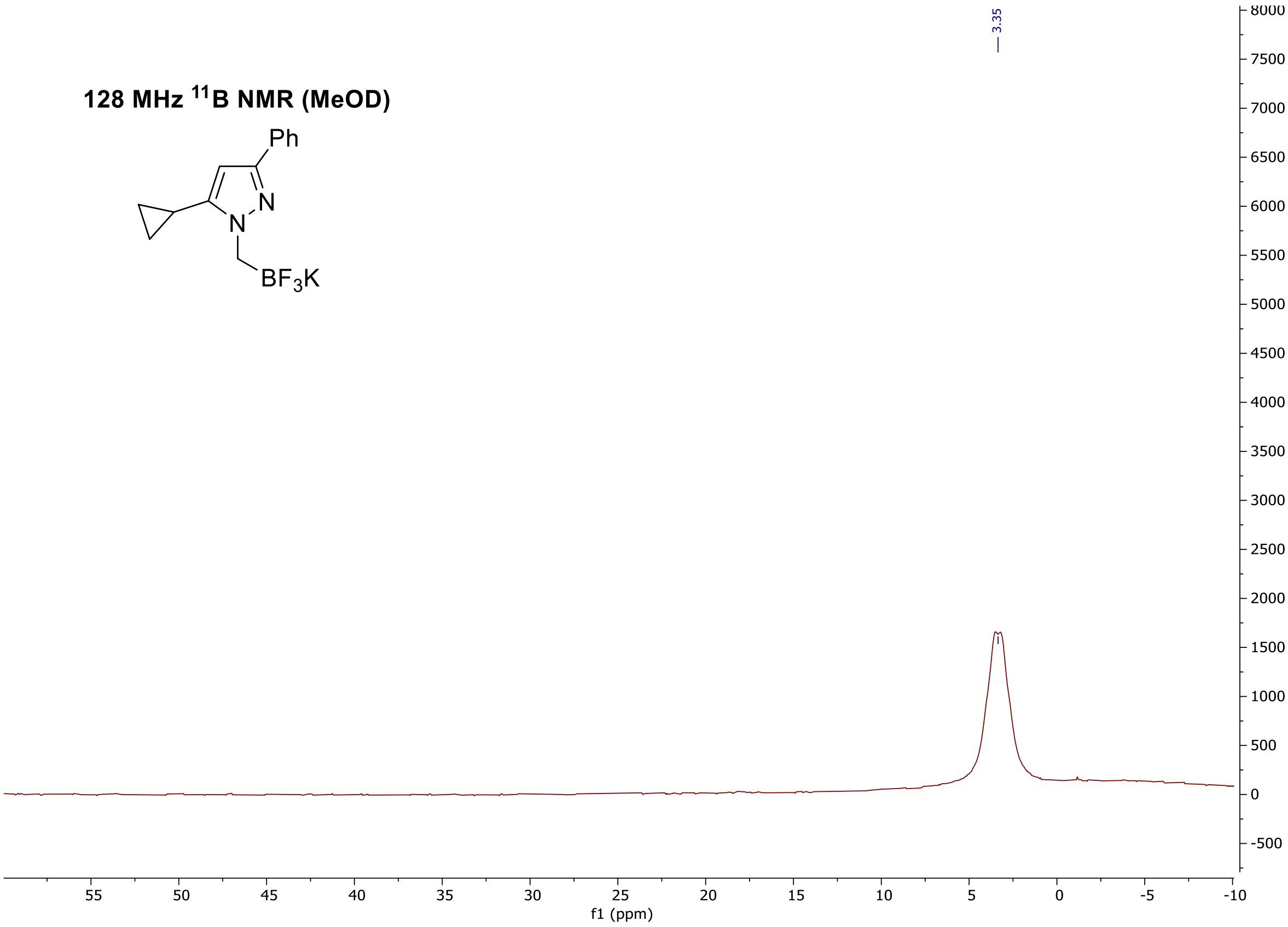

400 MHz ^{1}H NMR (d^6-DMSO)

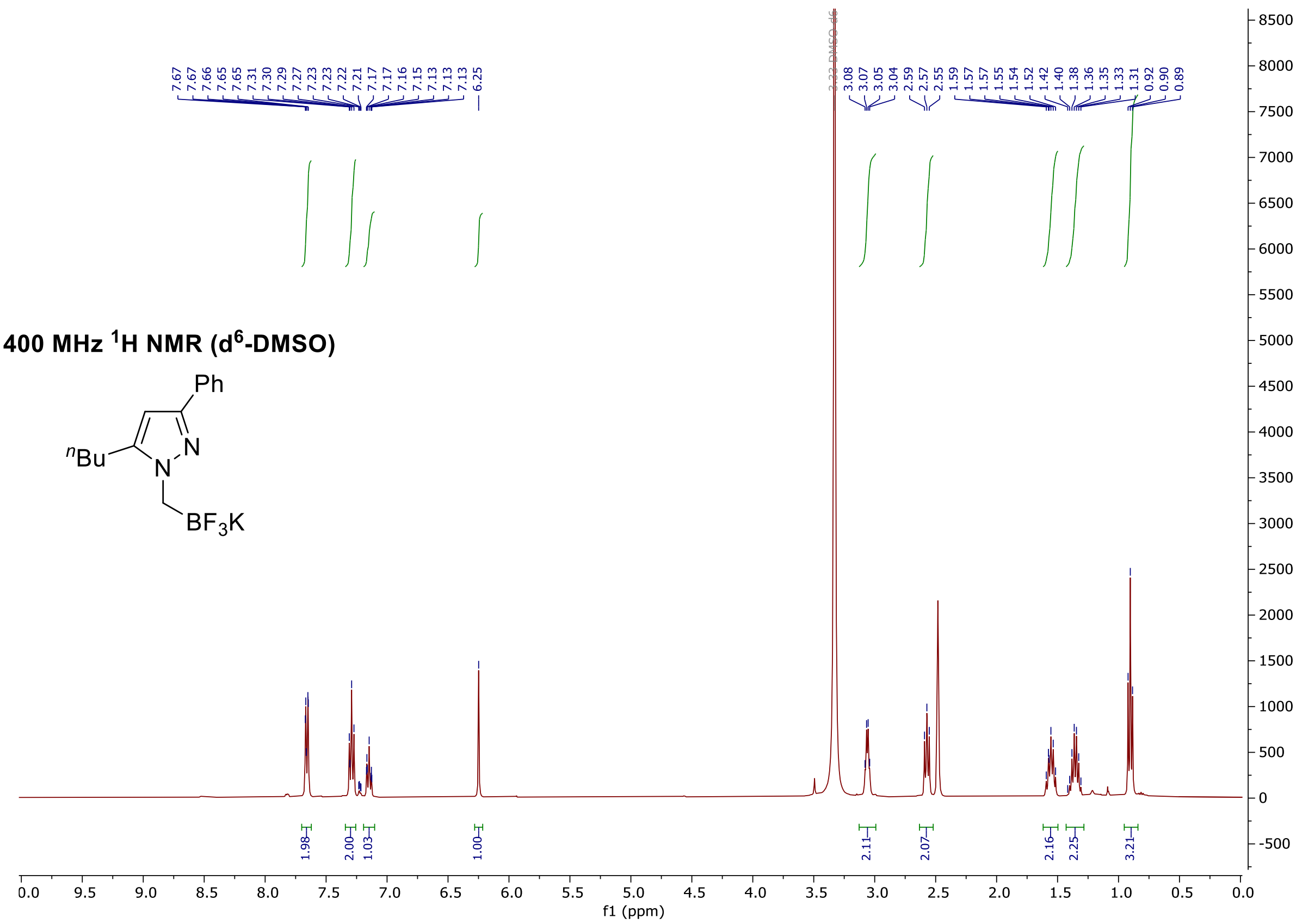

101 MHz {^{1}H}^{13}C NMR (d^6-DMSO)

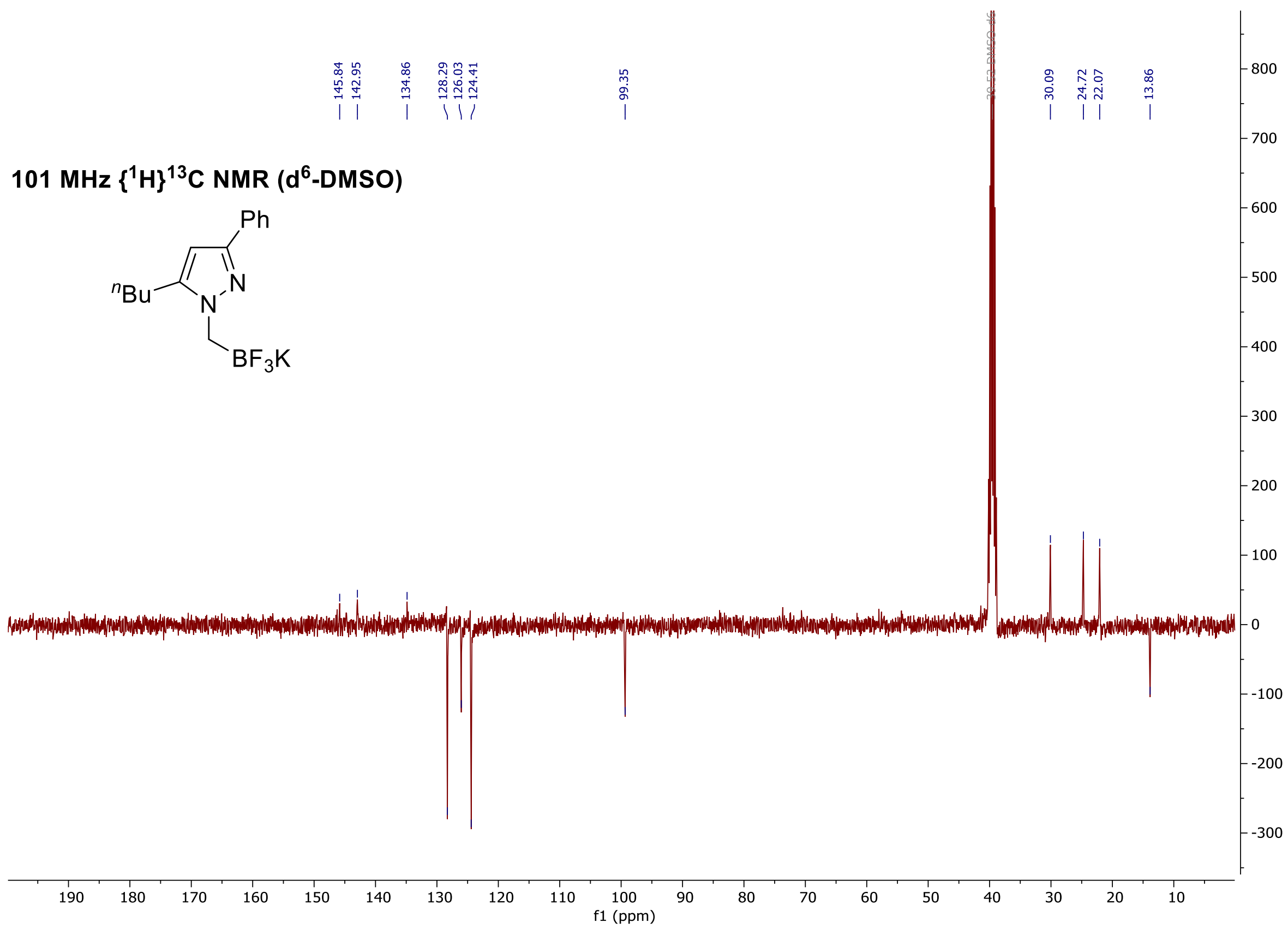

377 MHz ^{19}F NMR (d^{6}-DMSO)

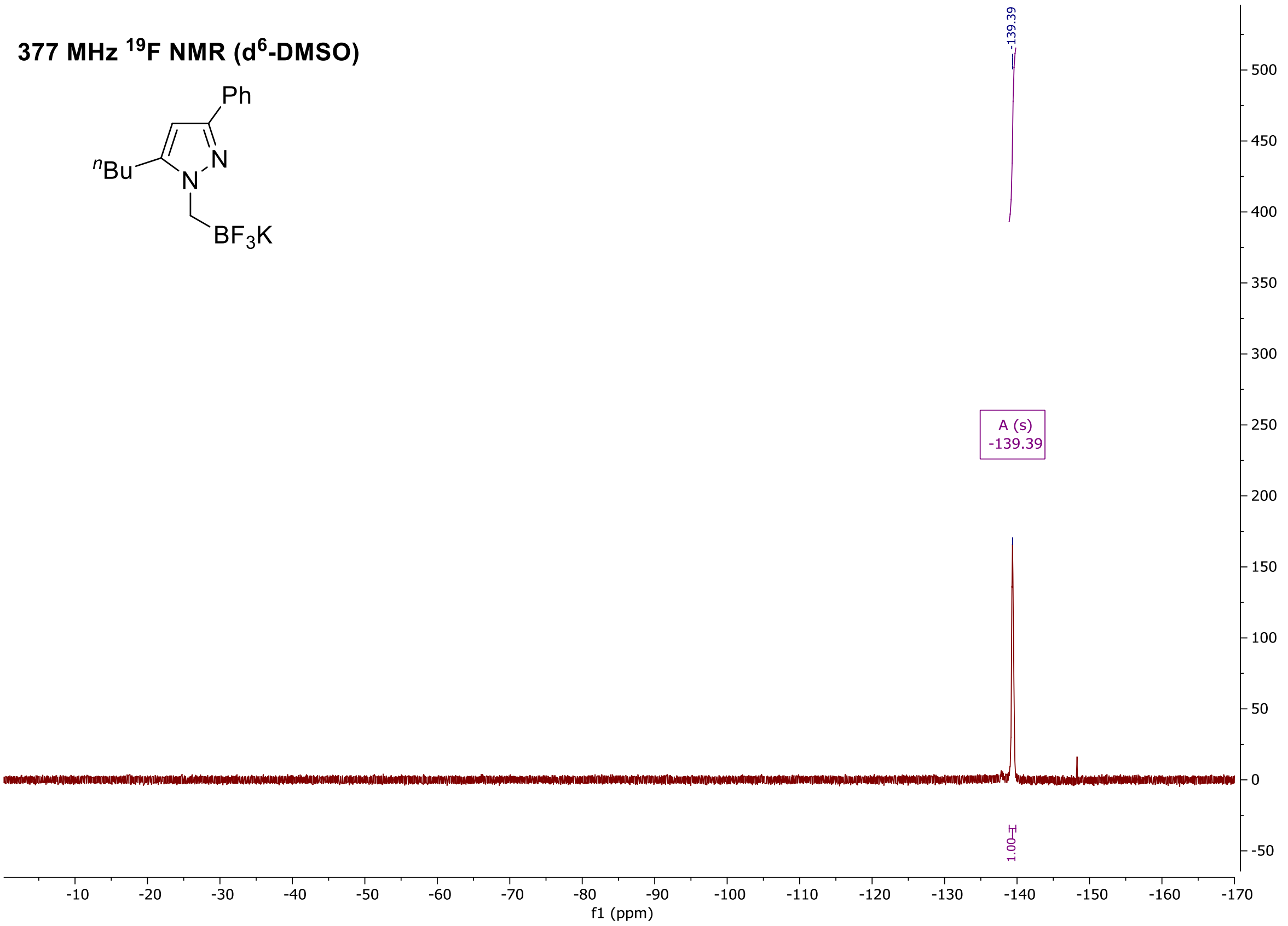

128 MHz ^{11}B NMR (d^{6}-DMSO)

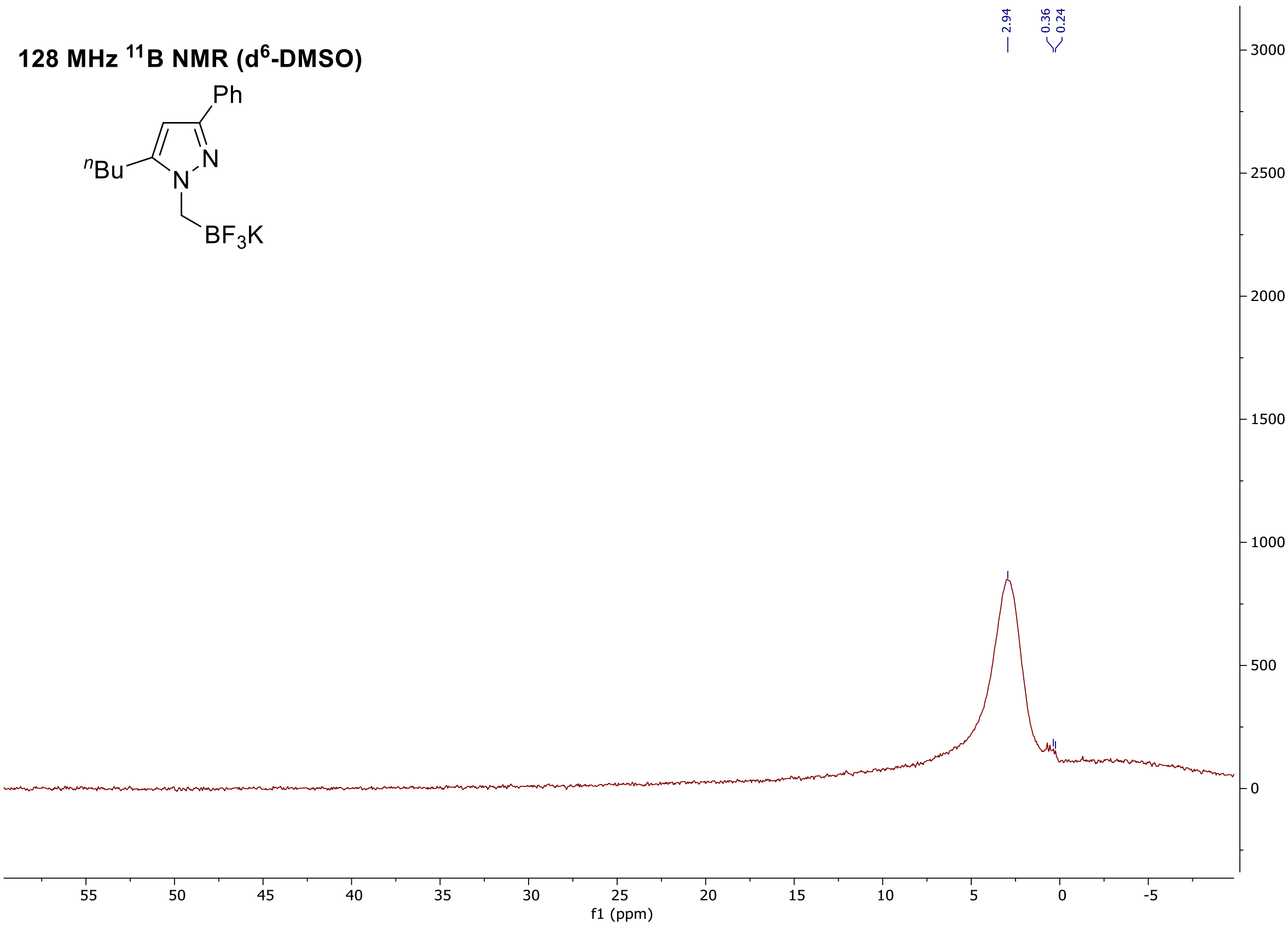

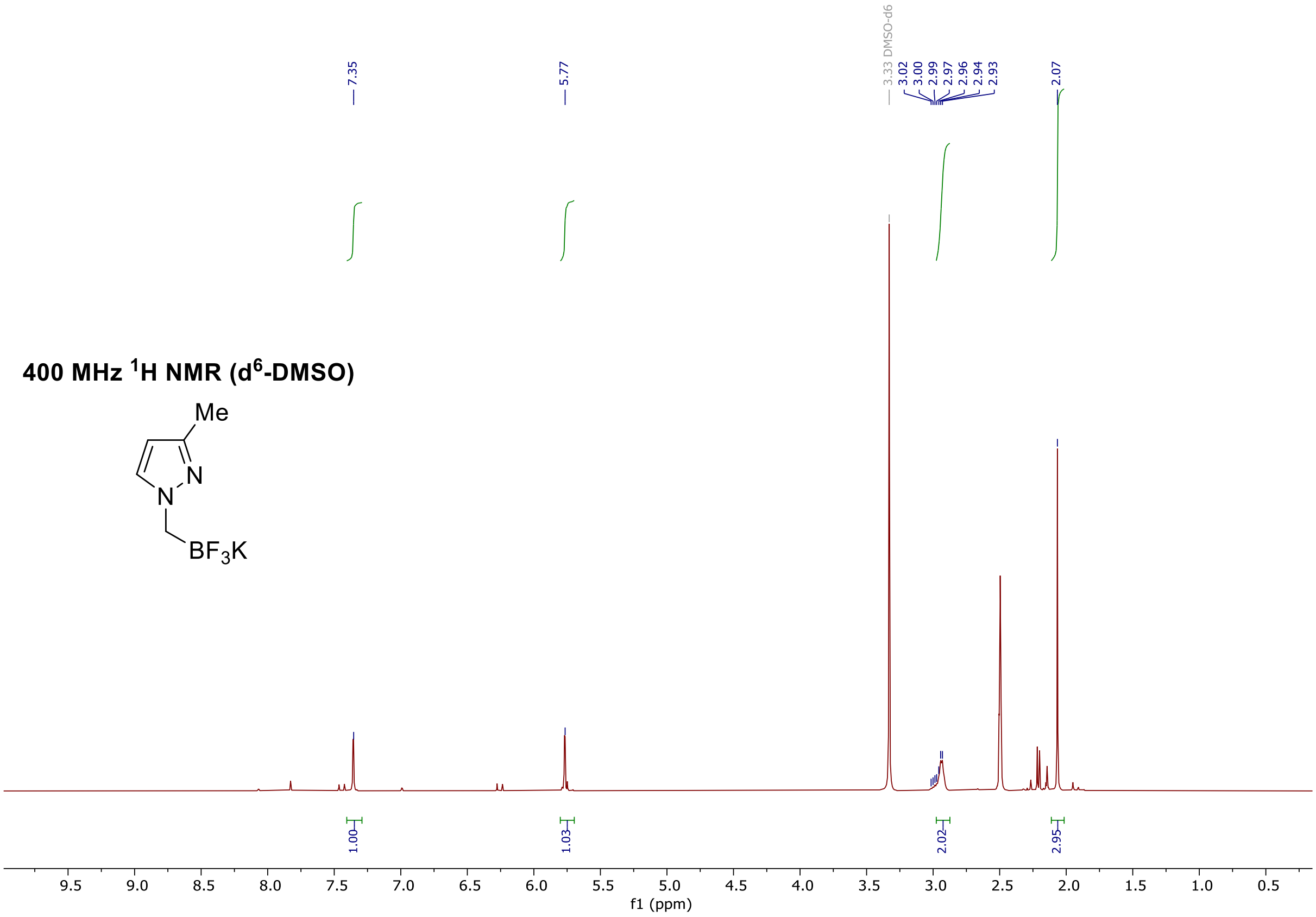
400 MHz ^{1}H NMR (d^6-DMSO)
Me
N
N
BF$_3$K
7.35
5.77
3.33 DMSO-d6
3.02
3.00
2.99
2.97
2.96
2.94
2.93
2.07
1.00
1.03
2.02
2.95
f1 (ppm)

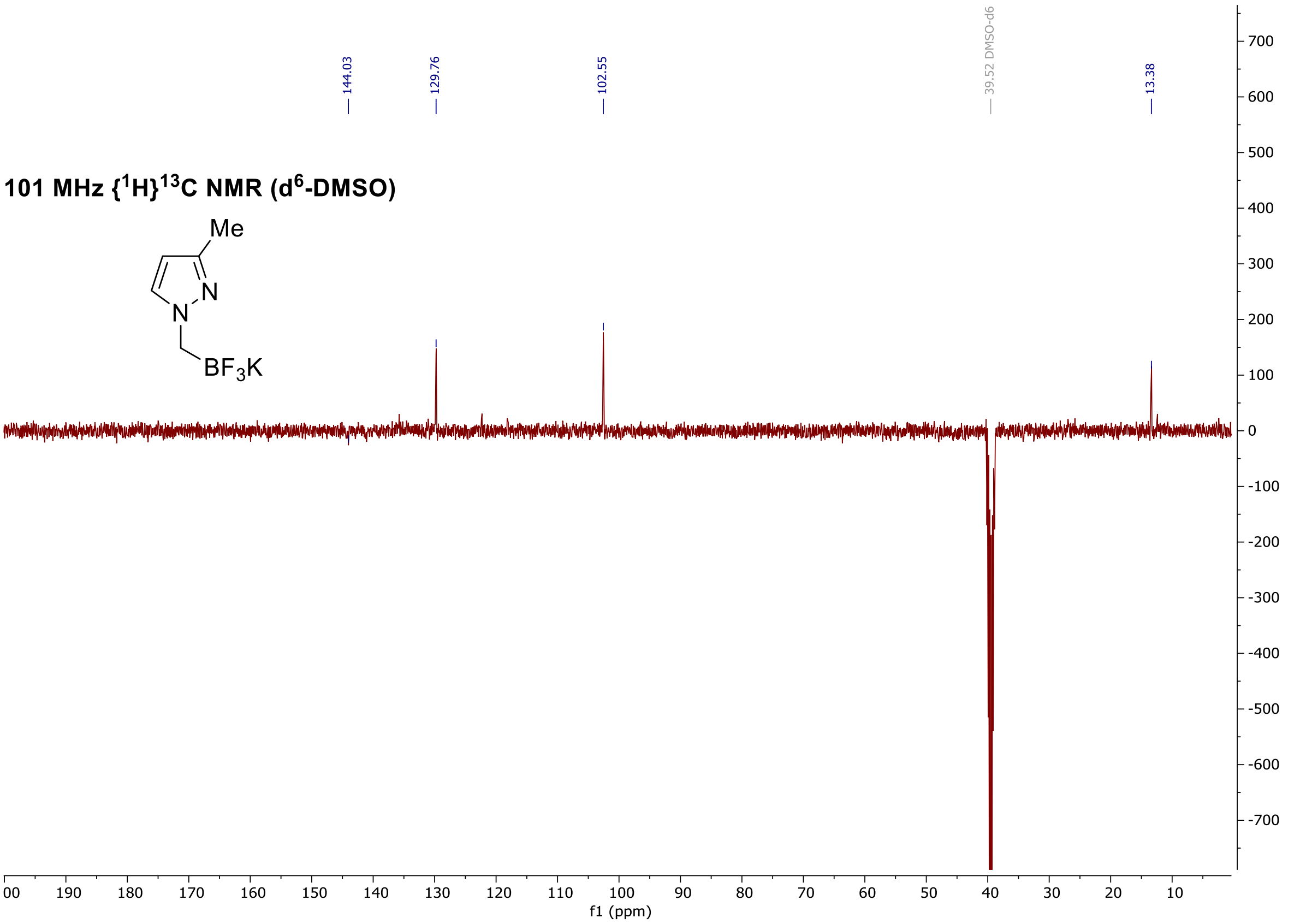
101 MHz {^{1}H}^{13}C NMR (d^6-DMSO)
Me
N
N
BF$_3$K
144.03
129.76
102.55
39.52 DMSO-d6
13.38
f1 (ppm)

377 MHz ^{19}F NMR (d^6-DMSO)

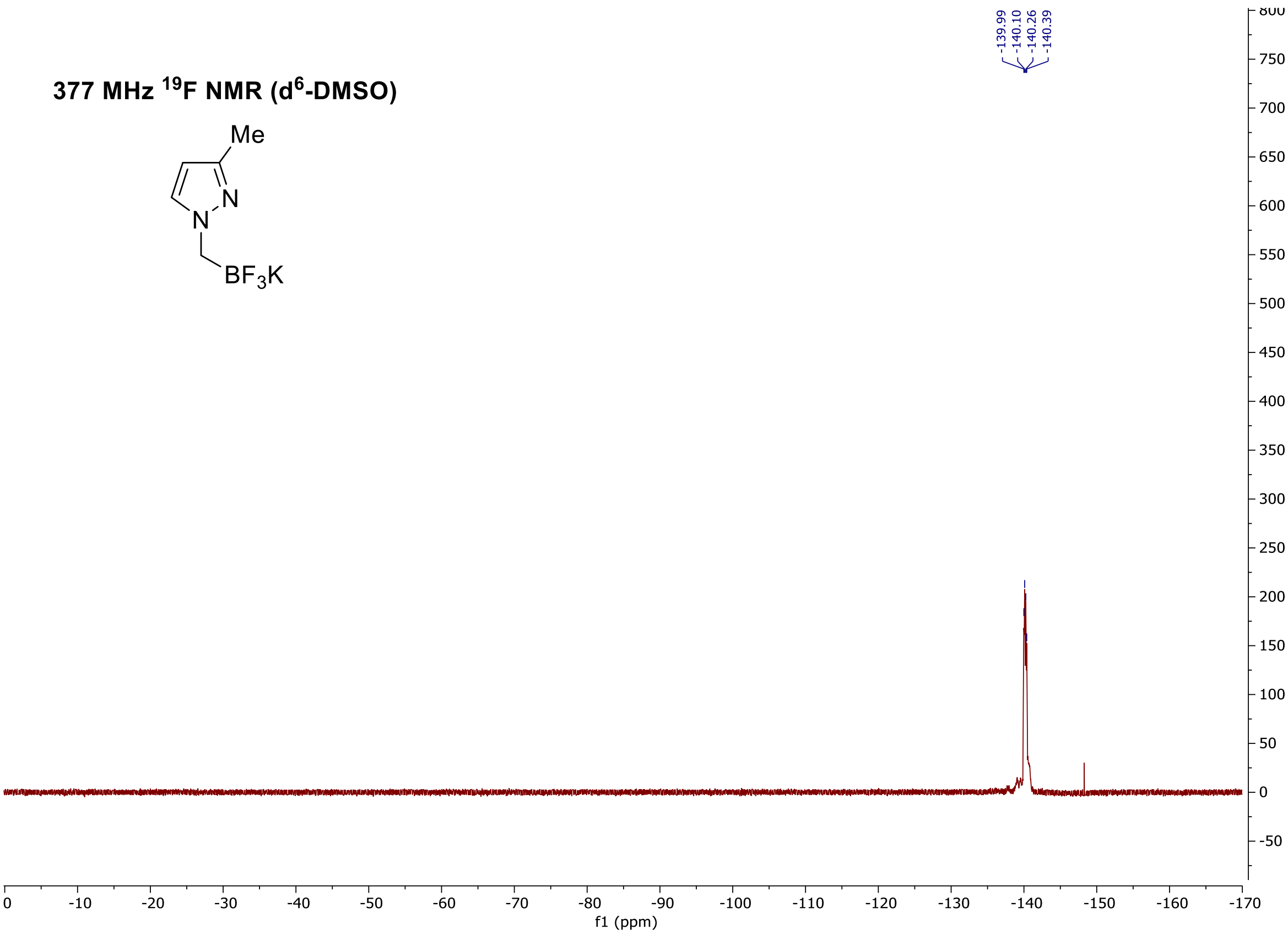

128 MHz ^{11}B NMR (d^6-DMSO)

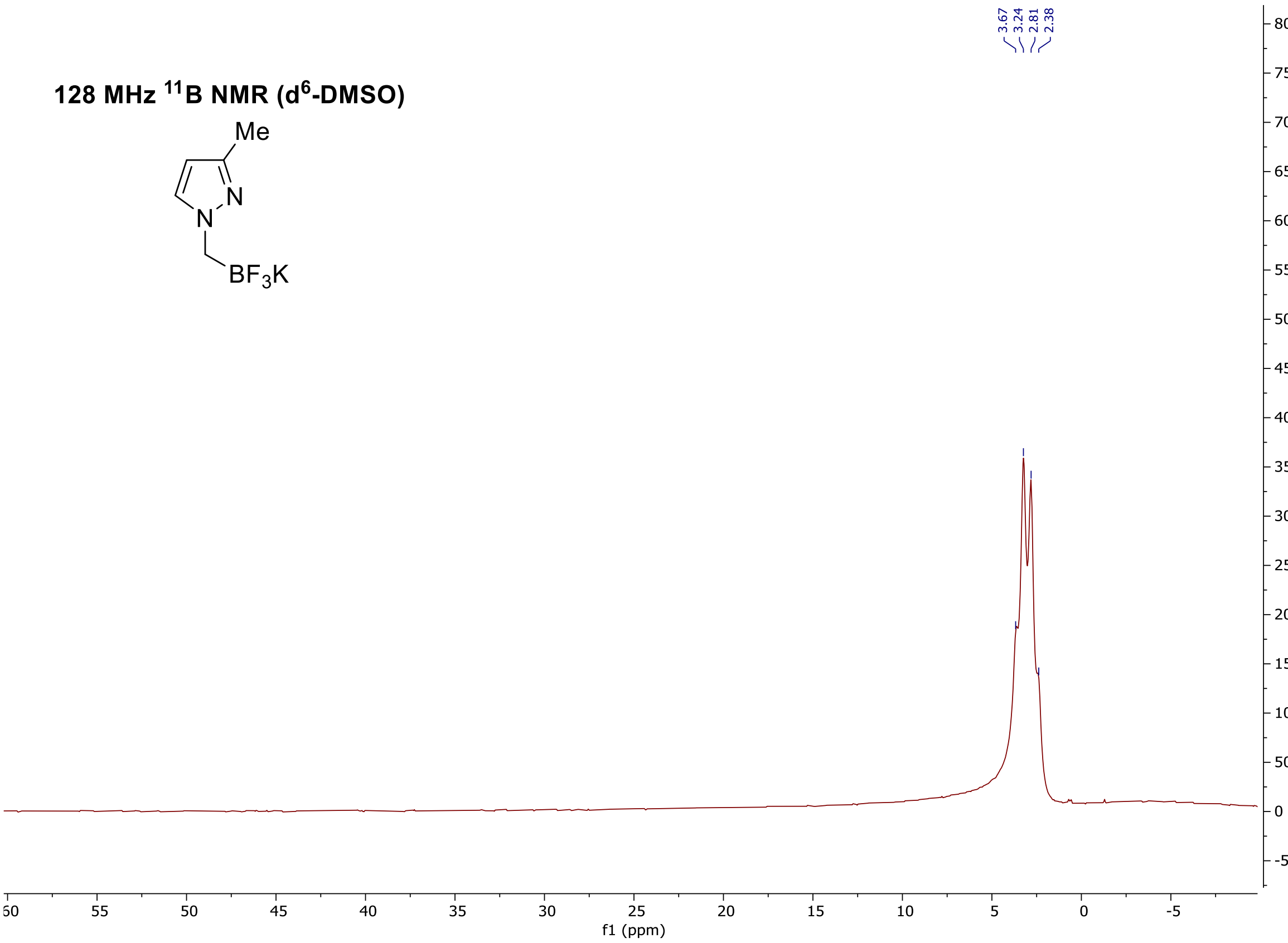

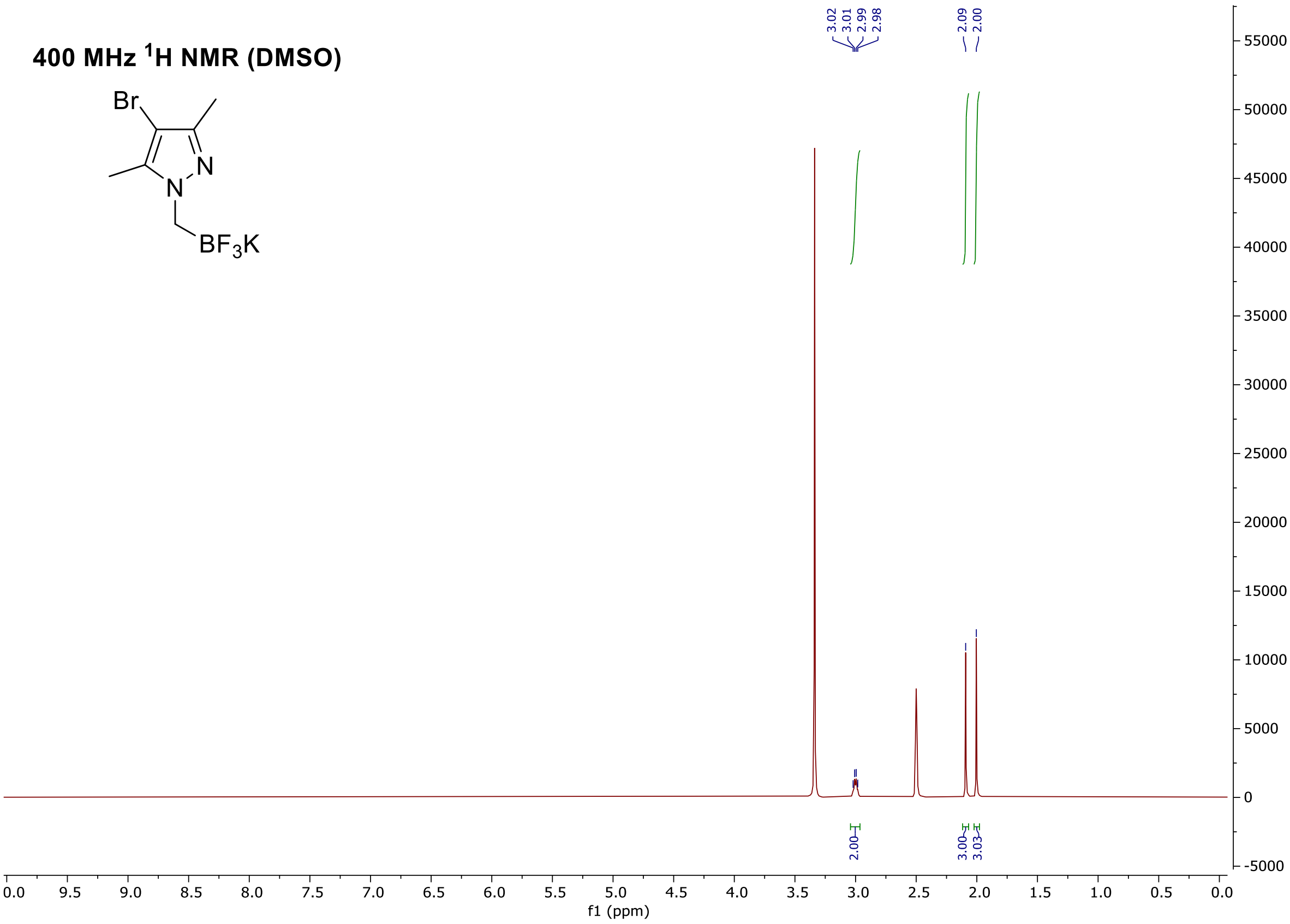
400 MHz 1H NMR (DMSO)
Br
N
N
BF3K
3.02
3.01
2.99
2.98
2.09
2.00
2.00
3.00
3.03
f1 (ppm)

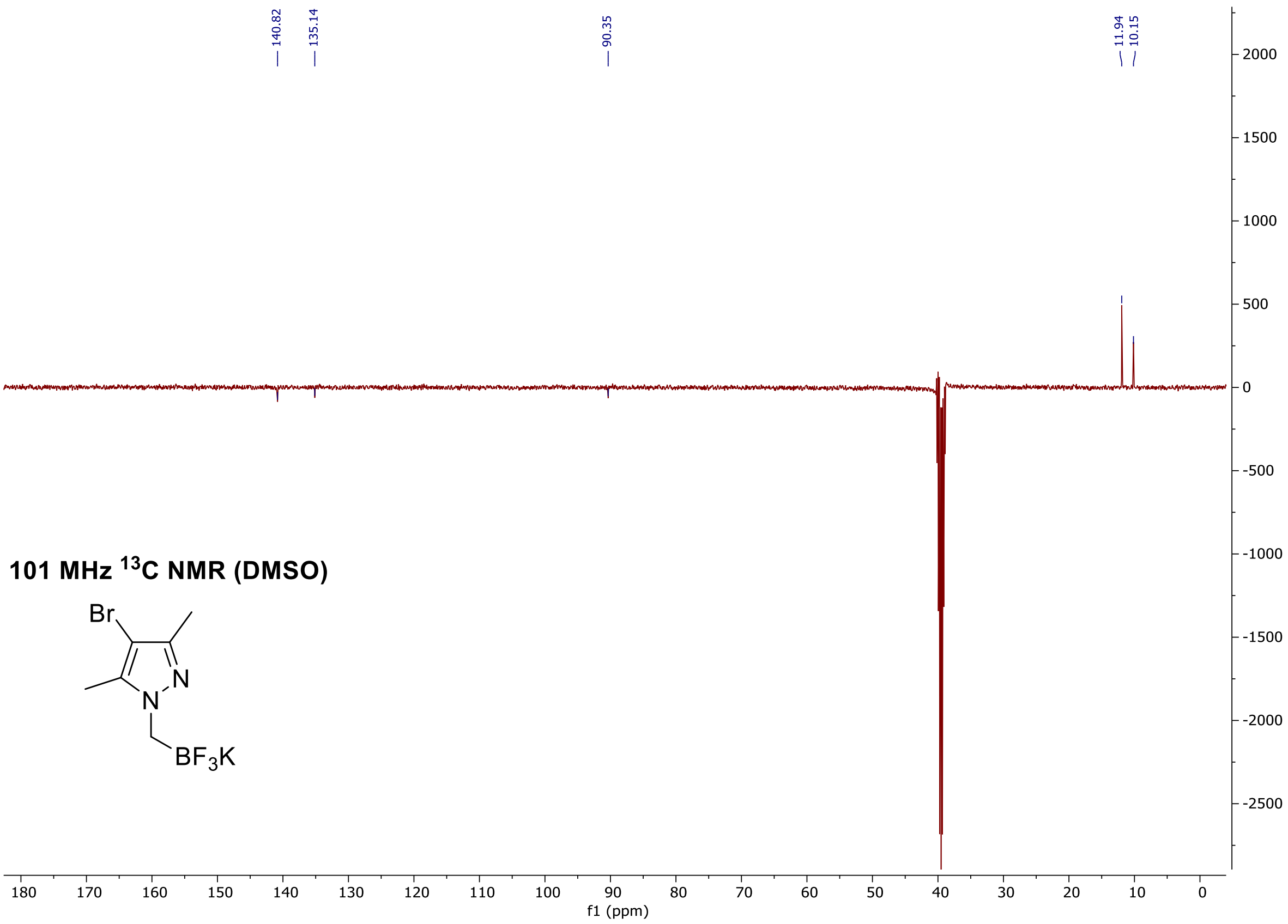
140.82
135.14
90.35
11.94
10.15
101 MHz 13C NMR (DMSO)
Br
N
N
BF3K
f1 (ppm)

377 MHz ^{19}F NMR (DMSO)

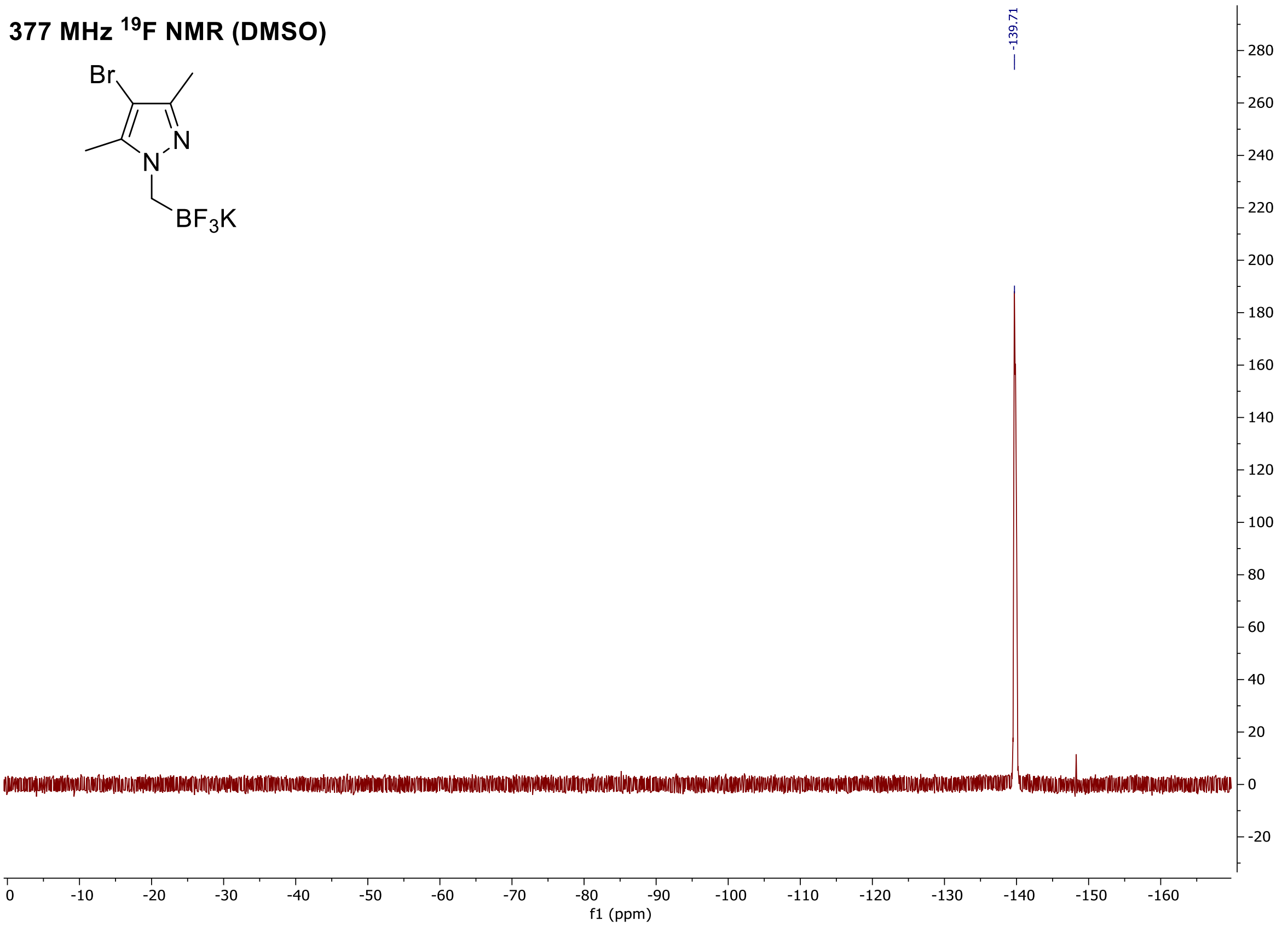

128 MHz ^{11}B NMR (DMSO)

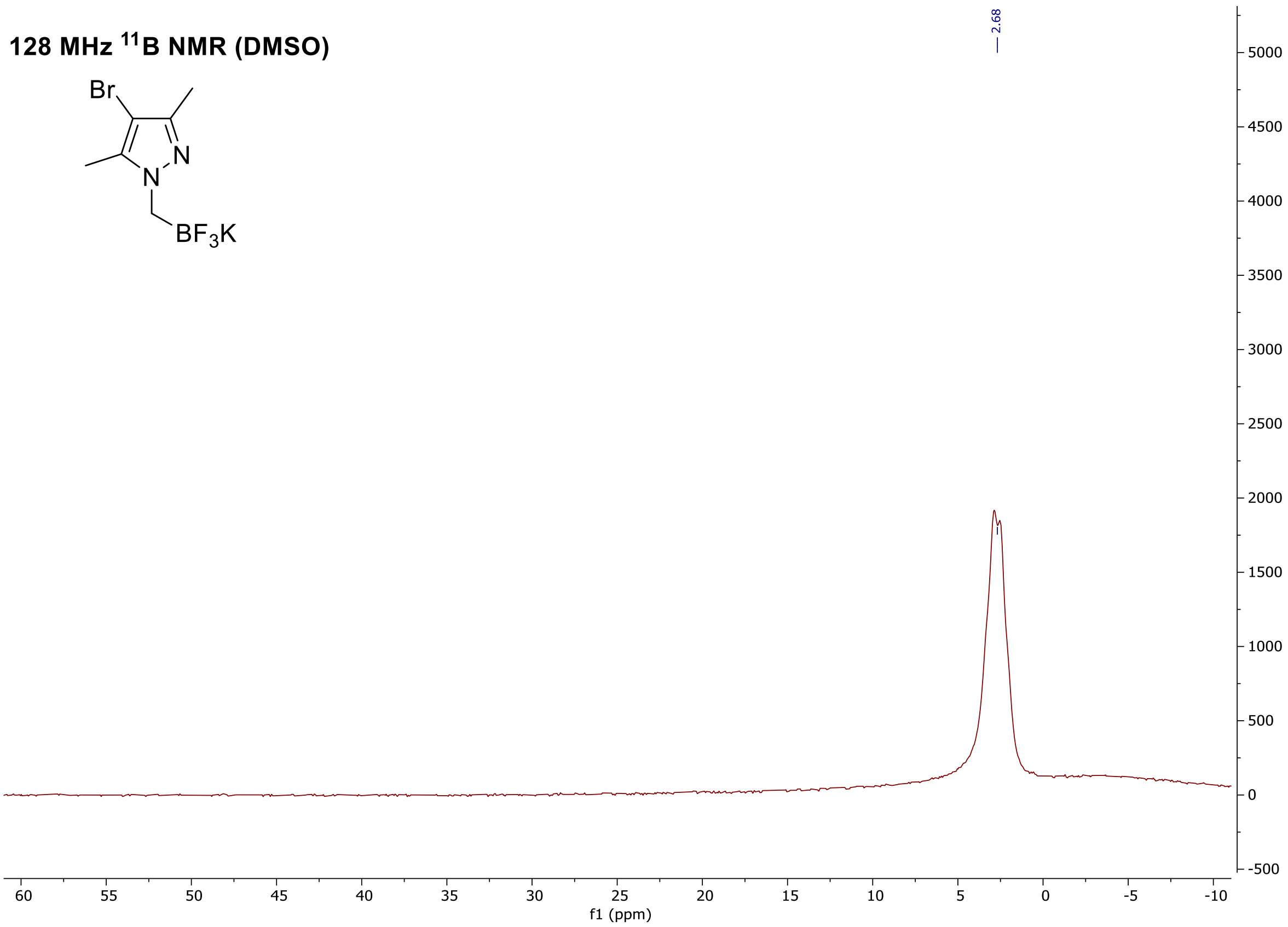

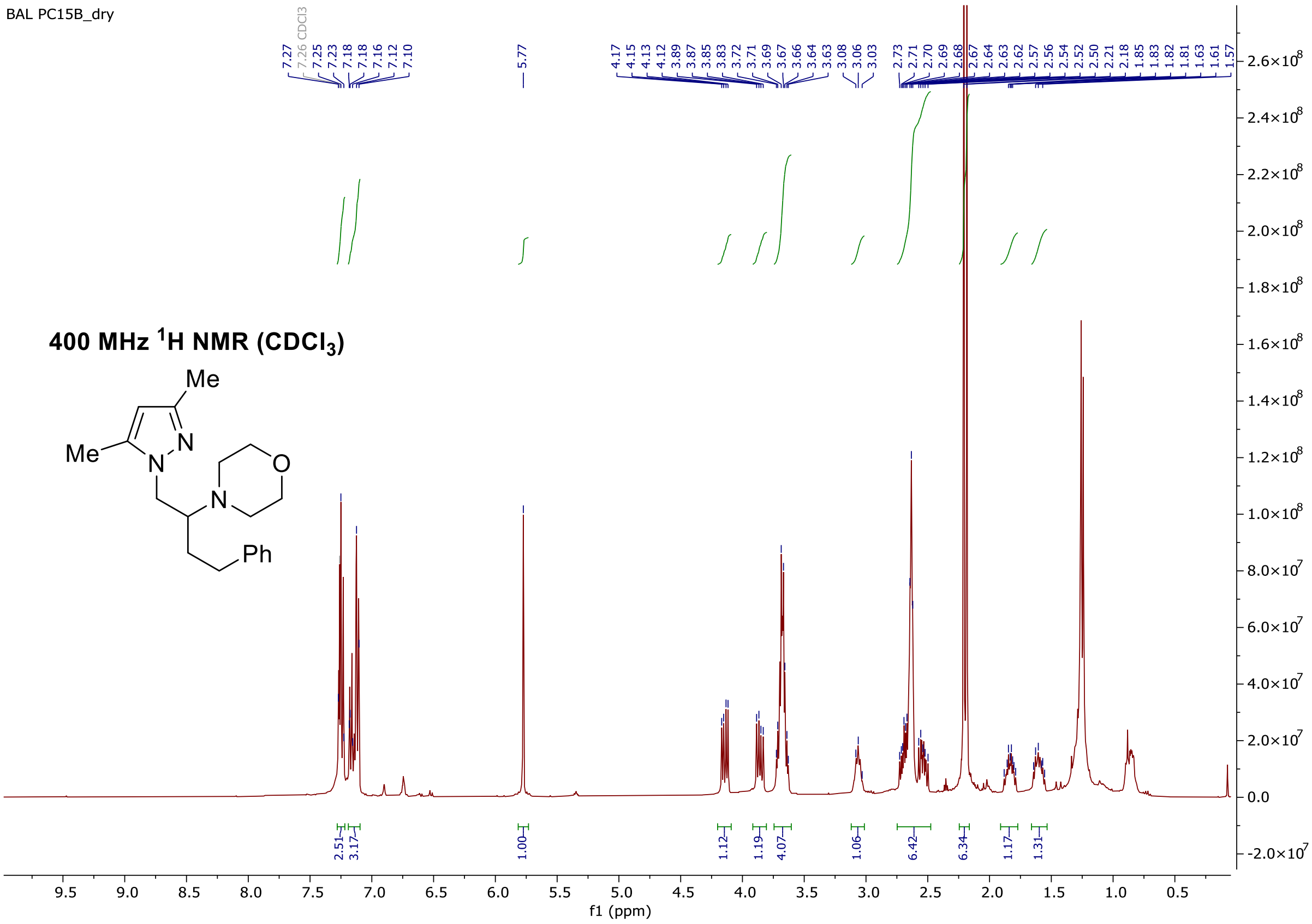
BAL PC15B_dry
400 MHz ¹H NMR (CDCl₃)
Me
Me
N
N
N
O
Ph
f1 (ppm)

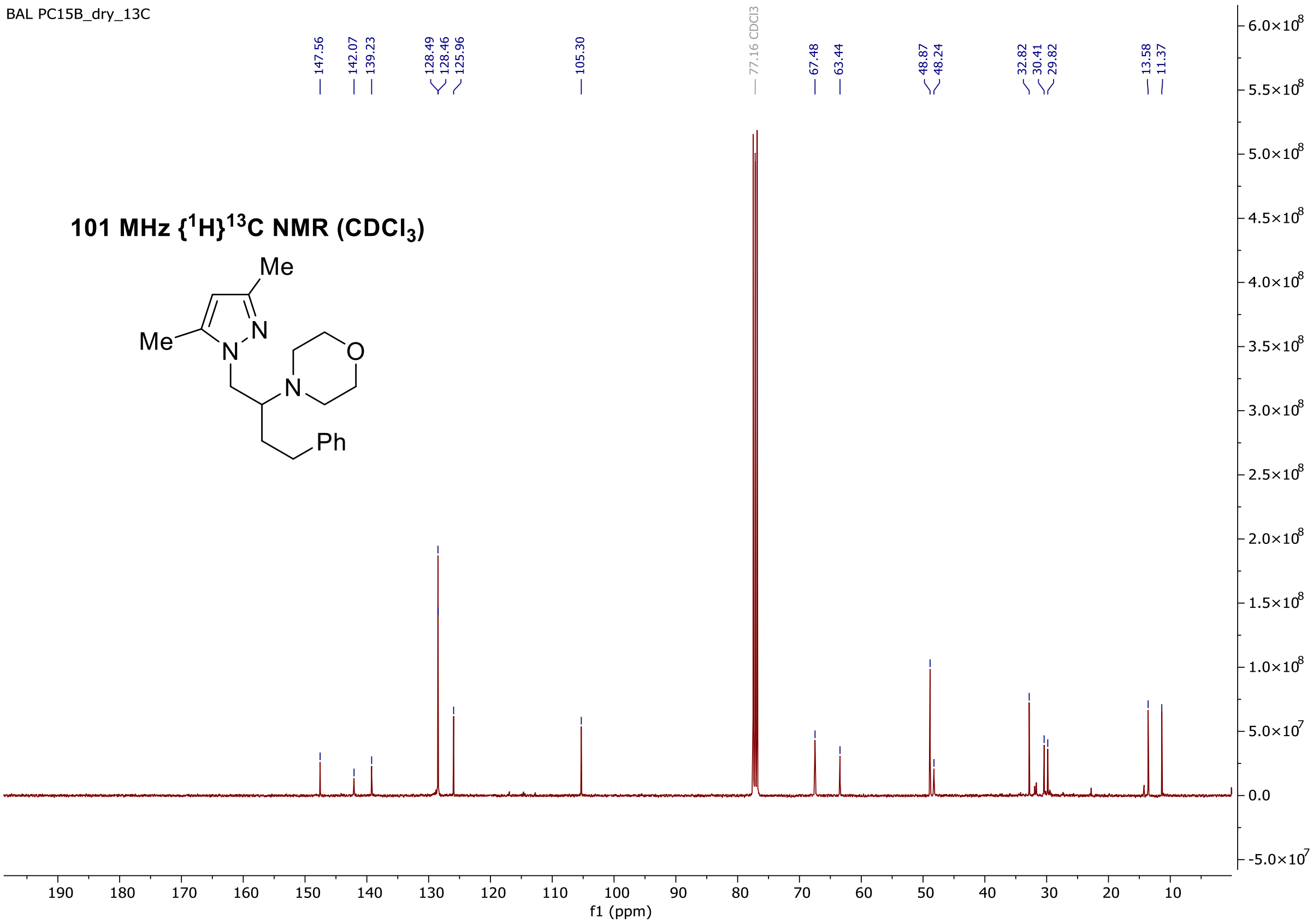
BAL PC15B_dry_13C
101 MHz {¹H}¹³C NMR (CDCl₃)
147.56
142.07
139.23
128.49
128.46
125.96
105.30
77.16 CDCl3
67.48
63.44
48.87
48.24
32.82
30.41
29.82
13.58
11.37
f1 (ppm)

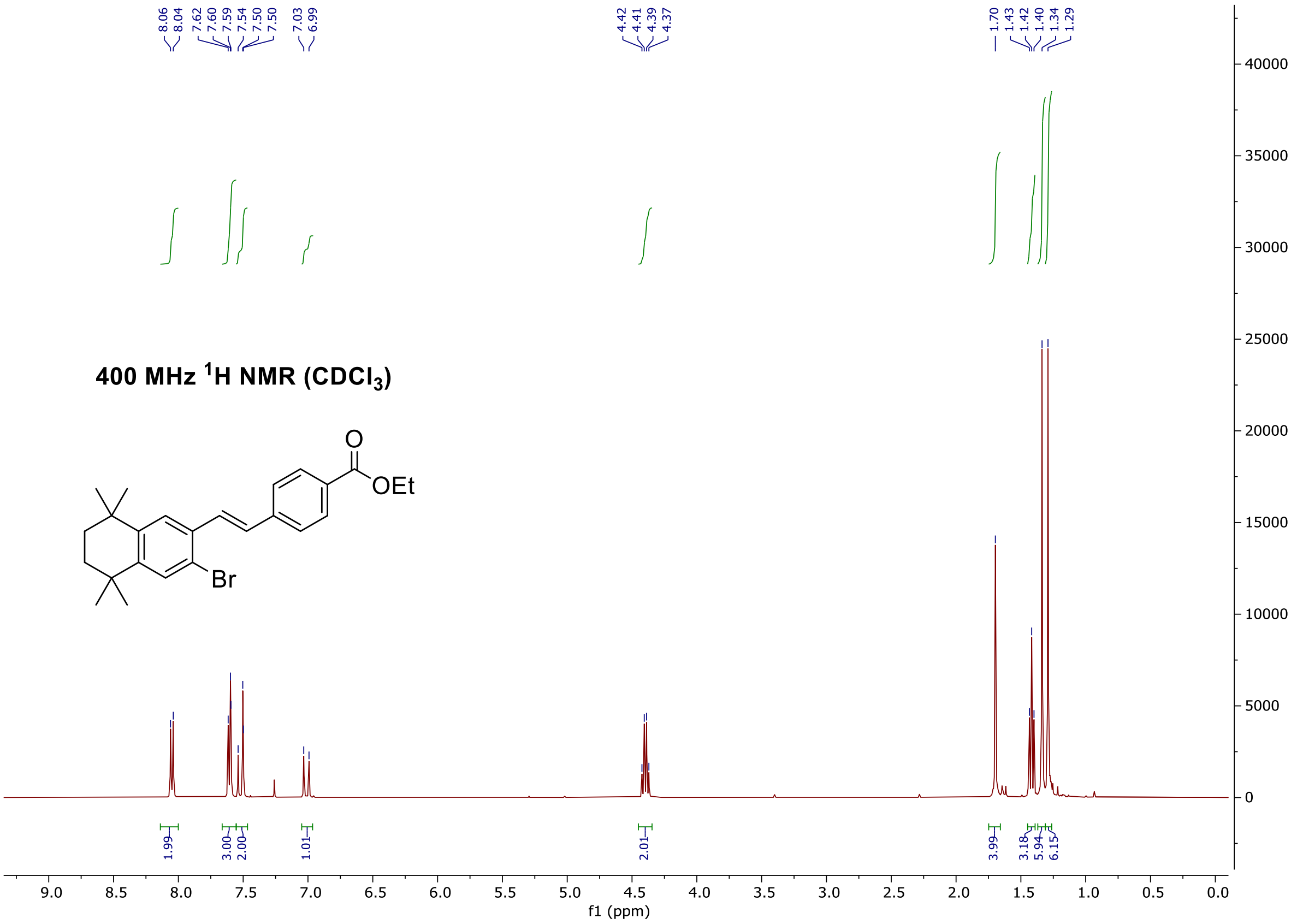
400 MHz ¹H NMR (CDCl₃)
OEt
O
Br
f1 (ppm)

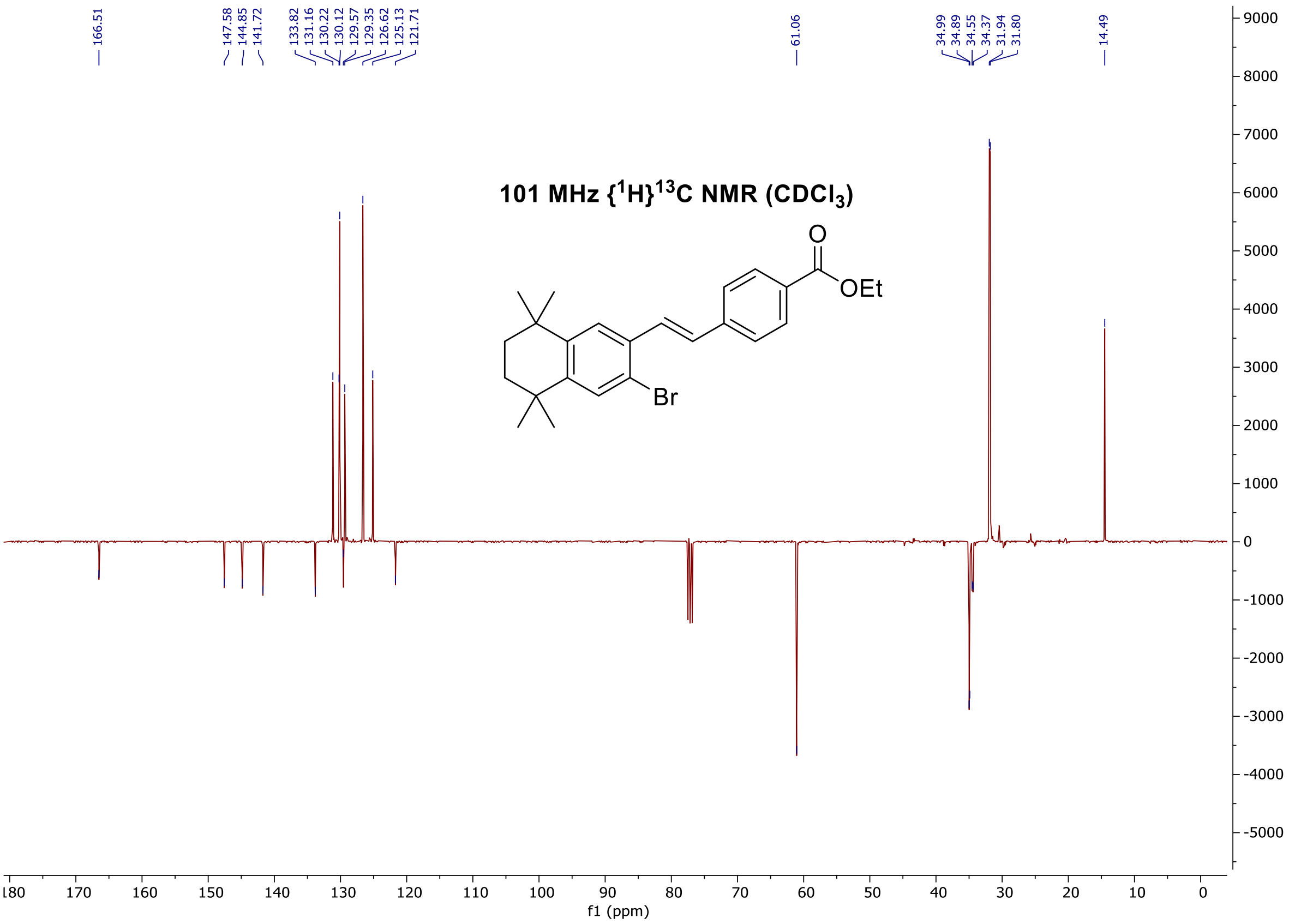
101 MHz {¹H}¹³C NMR (CDCl₃)
OEt
O
Br
f1 (ppm)

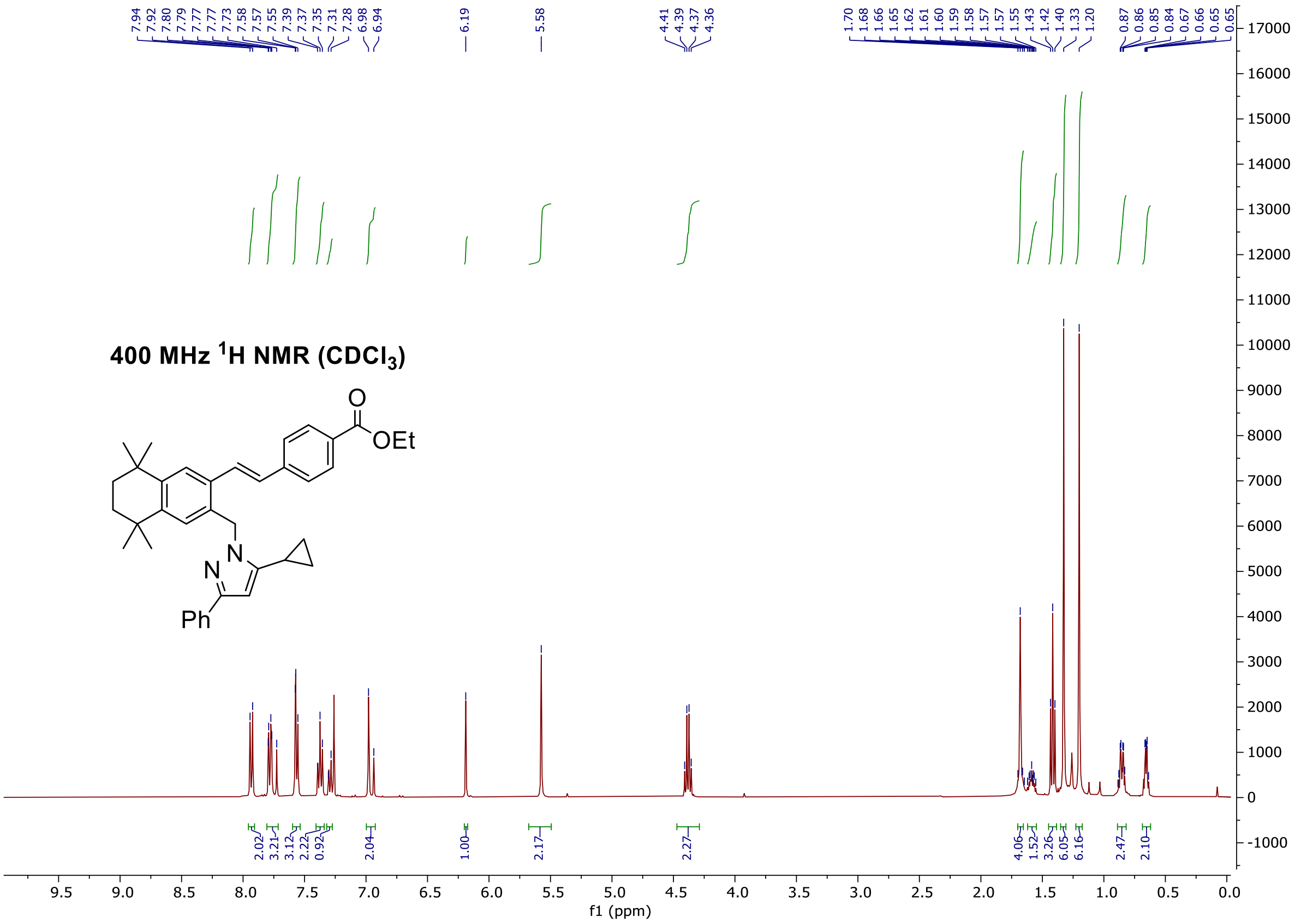
7.94
7.92
7.80
7.79
7.77
7.77
7.73
7.58
7.57
7.55
7.39
7.37
7.35
7.31
7.28
6.98
6.94
6.19
5.58
4.41
4.39
4.37
4.36
1.70
1.68
1.66
1.65
1.62
1.61
1.60
1.59
1.58
1.57
1.57
1.55
1.43
1.42
1.40
1.33
1.20
0.87
0.86
0.85
0.84
0.67
0.66
0.65
0.65
400 MHz ¹H NMR (CDCl3)
OEt
O
N
N
Ph
2.02
3.21
3.12
2.22
0.92
2.04
1.00
2.17
2.27
4.06
1.52
3.26
6.05
6.16
2.47
2.10
f1 (ppm)

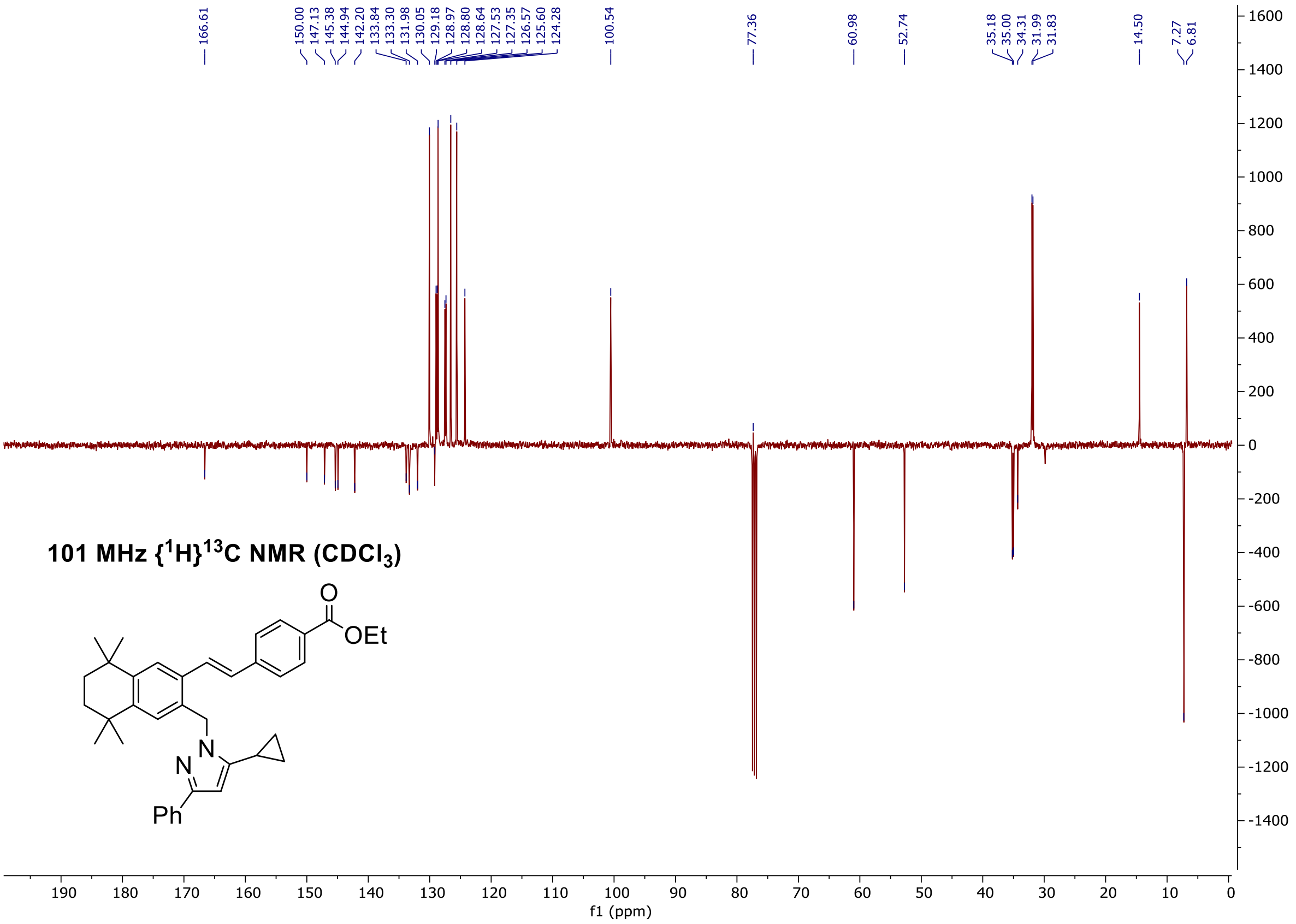
166.61
150.00
147.13
145.38
144.94
142.20
133.84
133.30
131.98
130.05
129.18
128.97
128.80
128.64
127.53
127.35
126.57
125.60
124.28
100.54
77.36
60.98
52.74
35.18
35.00
34.31
31.99
31.83
14.50
7.27
6.81
101 MHz {¹H}¹³C NMR (CDCl3)
OEt
O
N
N
Ph
f1 (ppm)

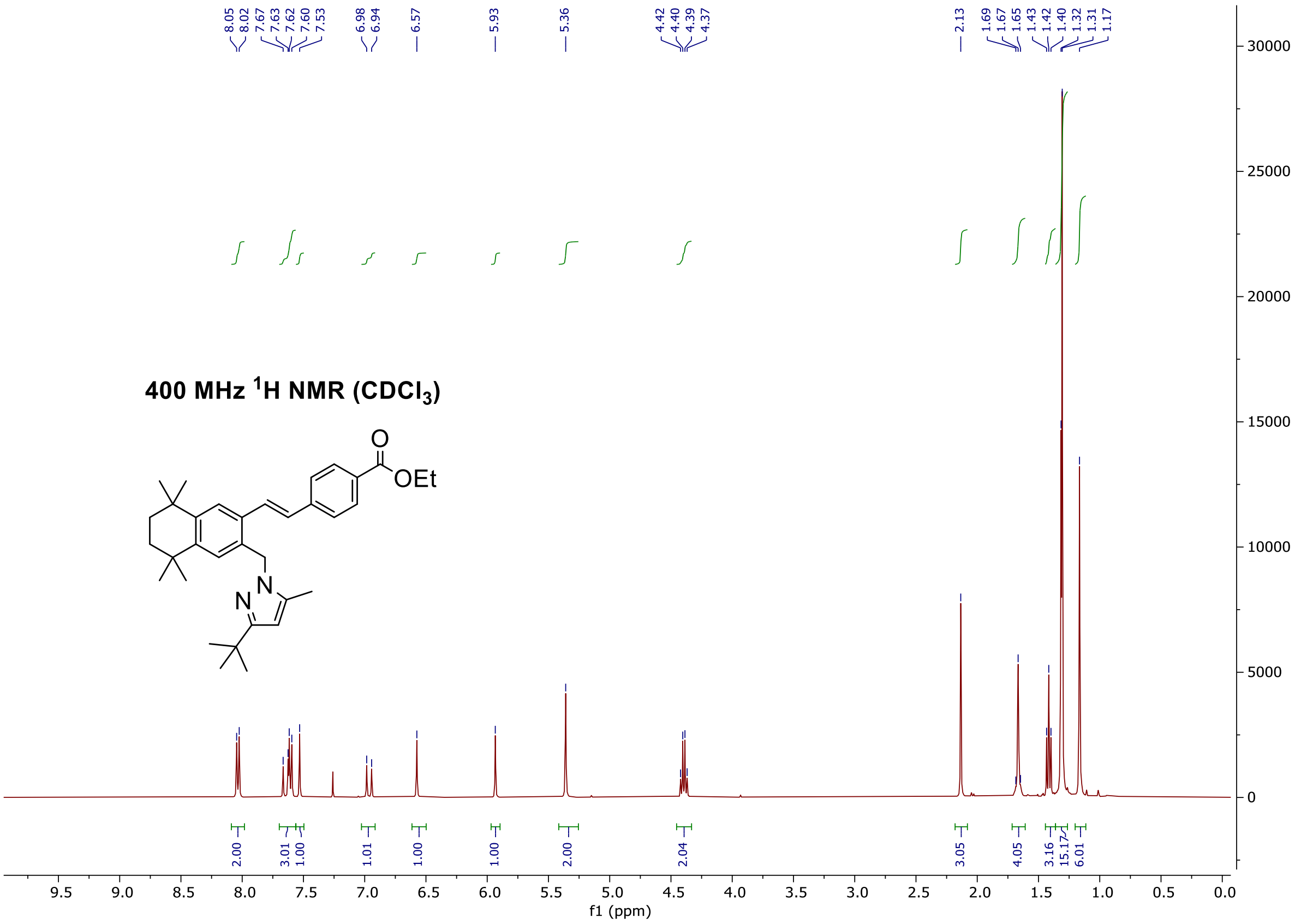
400 MHz ¹H NMR (CDCl₃)
OEt
N
f1 (ppm)

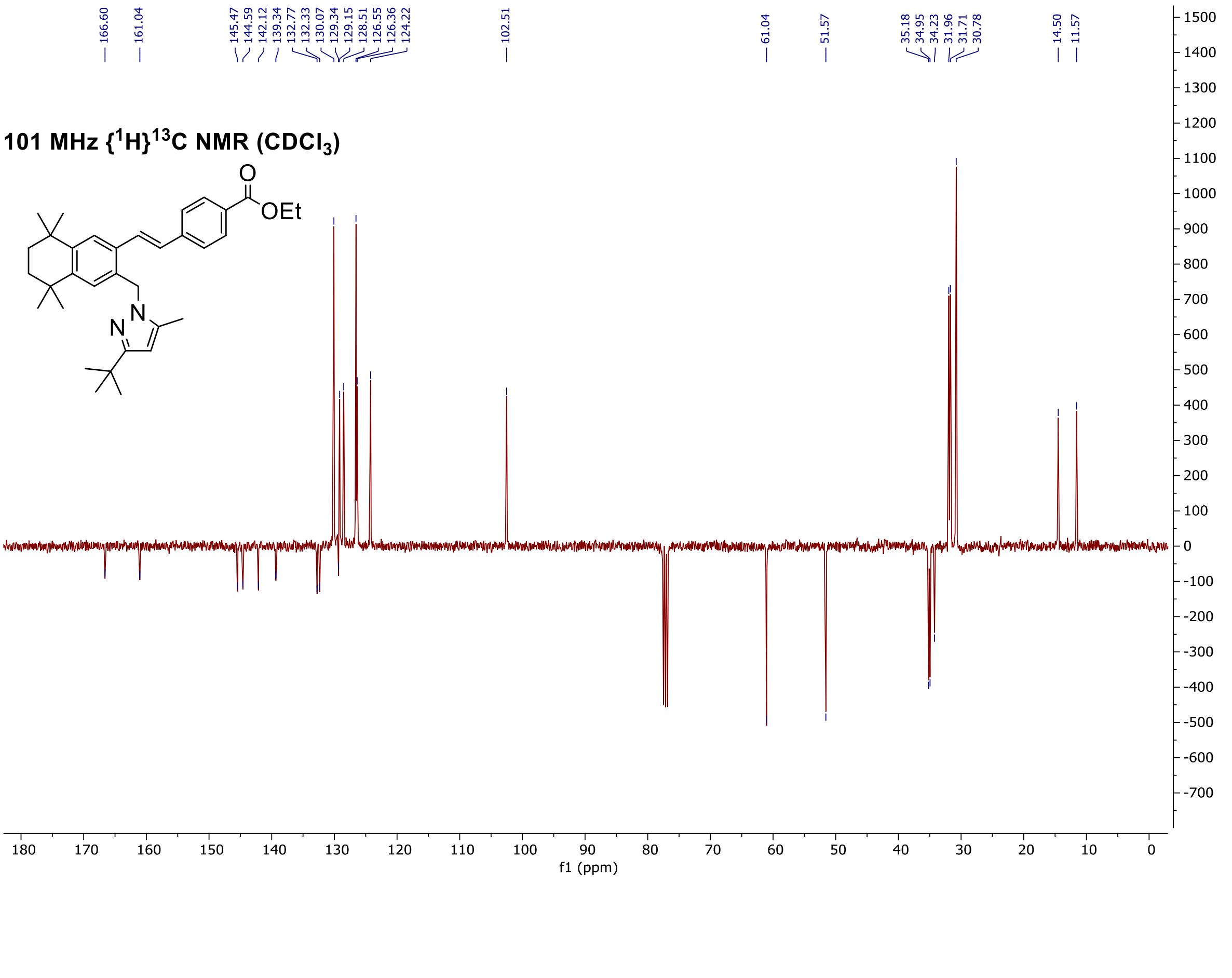
101 MHz {¹H}¹³C NMR (CDCl₃)
OEt
N
f1 (ppm)

Regiochemical Assignment of Selected Compounds

1-(2,4-Dinitrophenyl)-1-((trifluoroboraneyl)methyl)hydrazine, potassium salt (6)

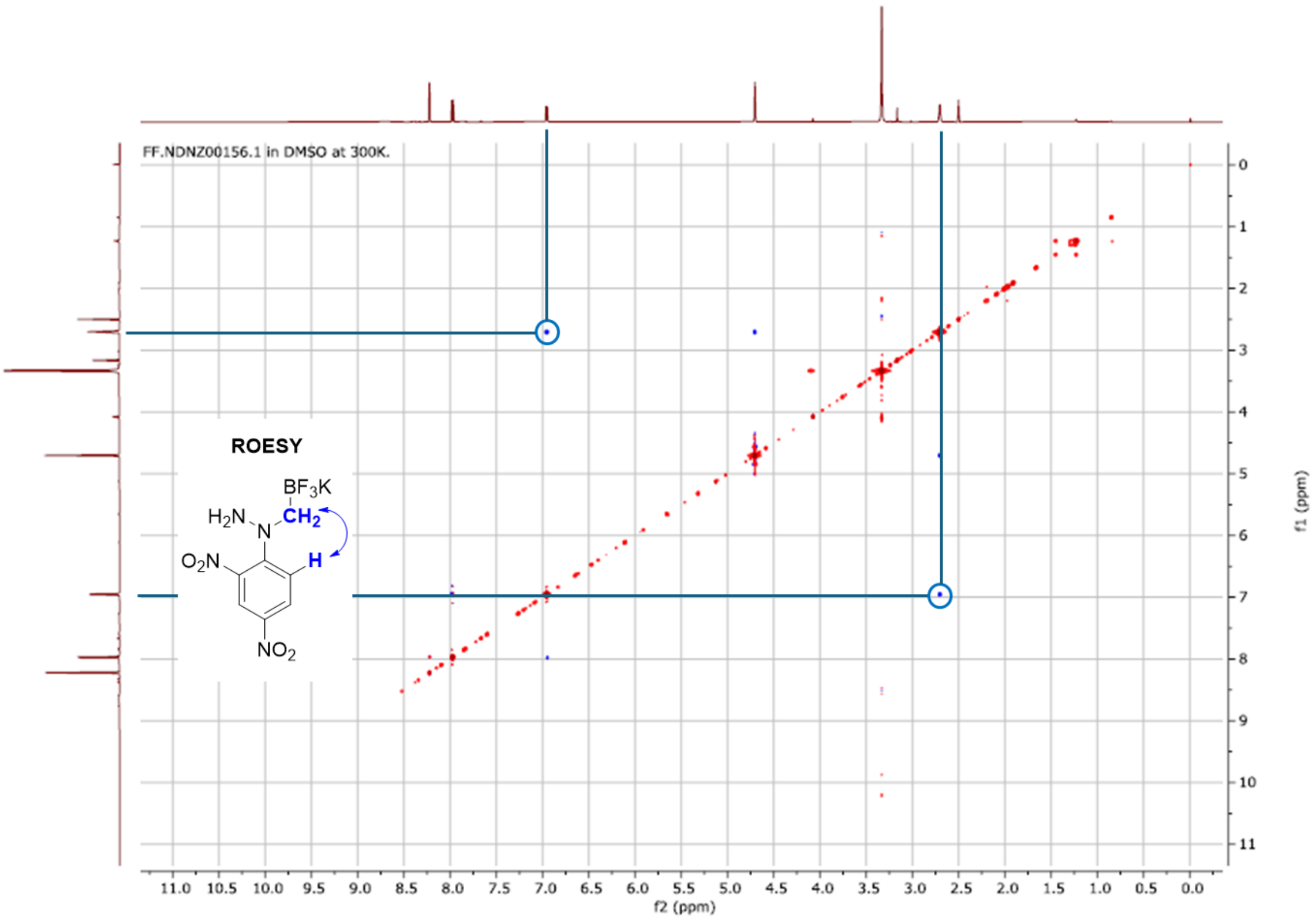

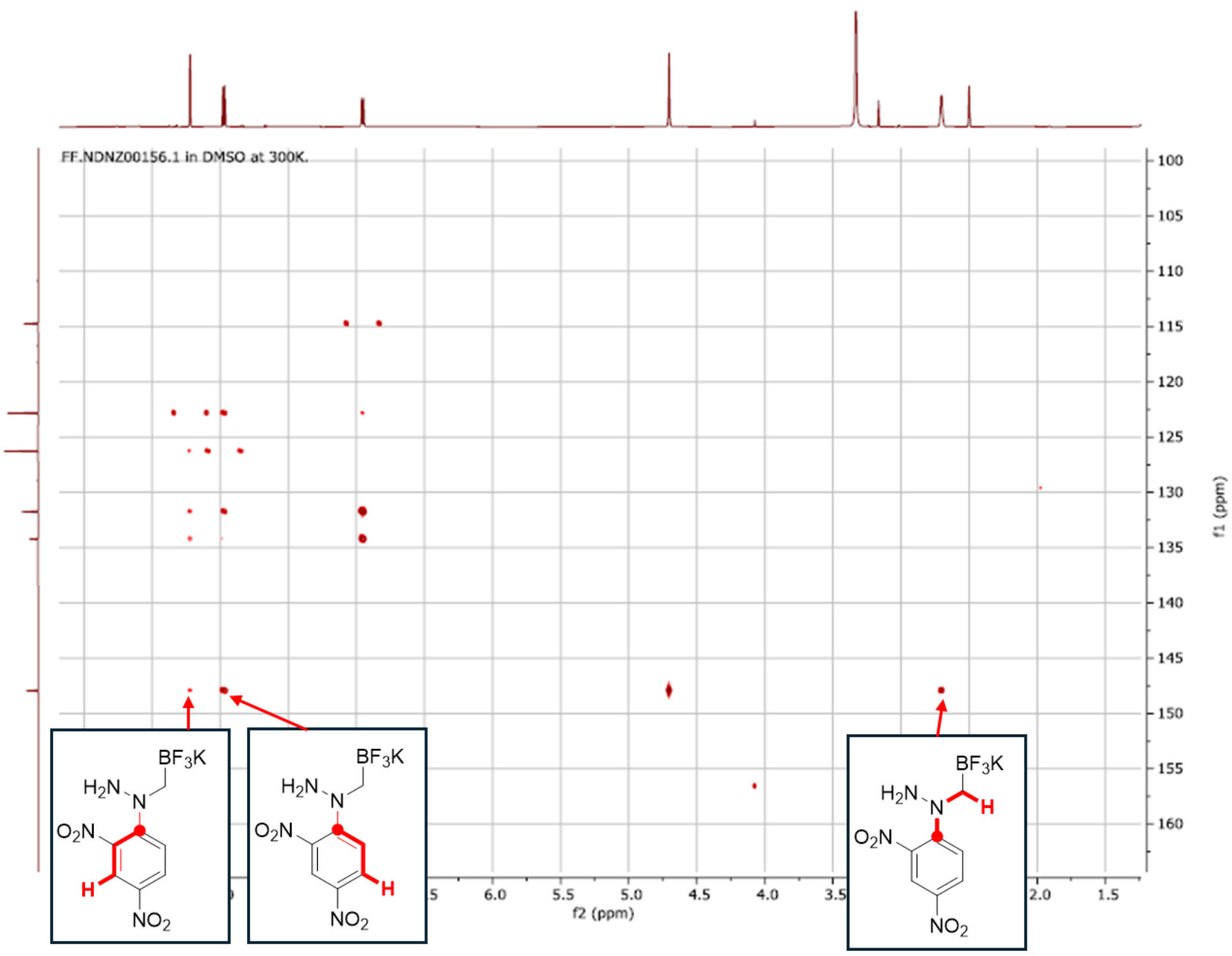

Potassium 3-tert-butyl-5-methyl(trifluoro(1-methylpyrazolyl)borate (17)

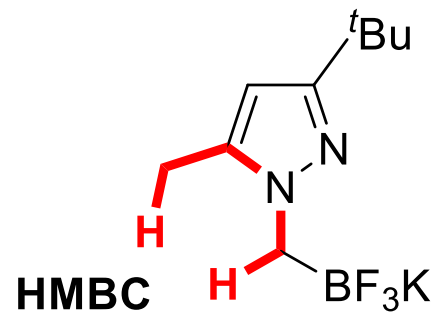

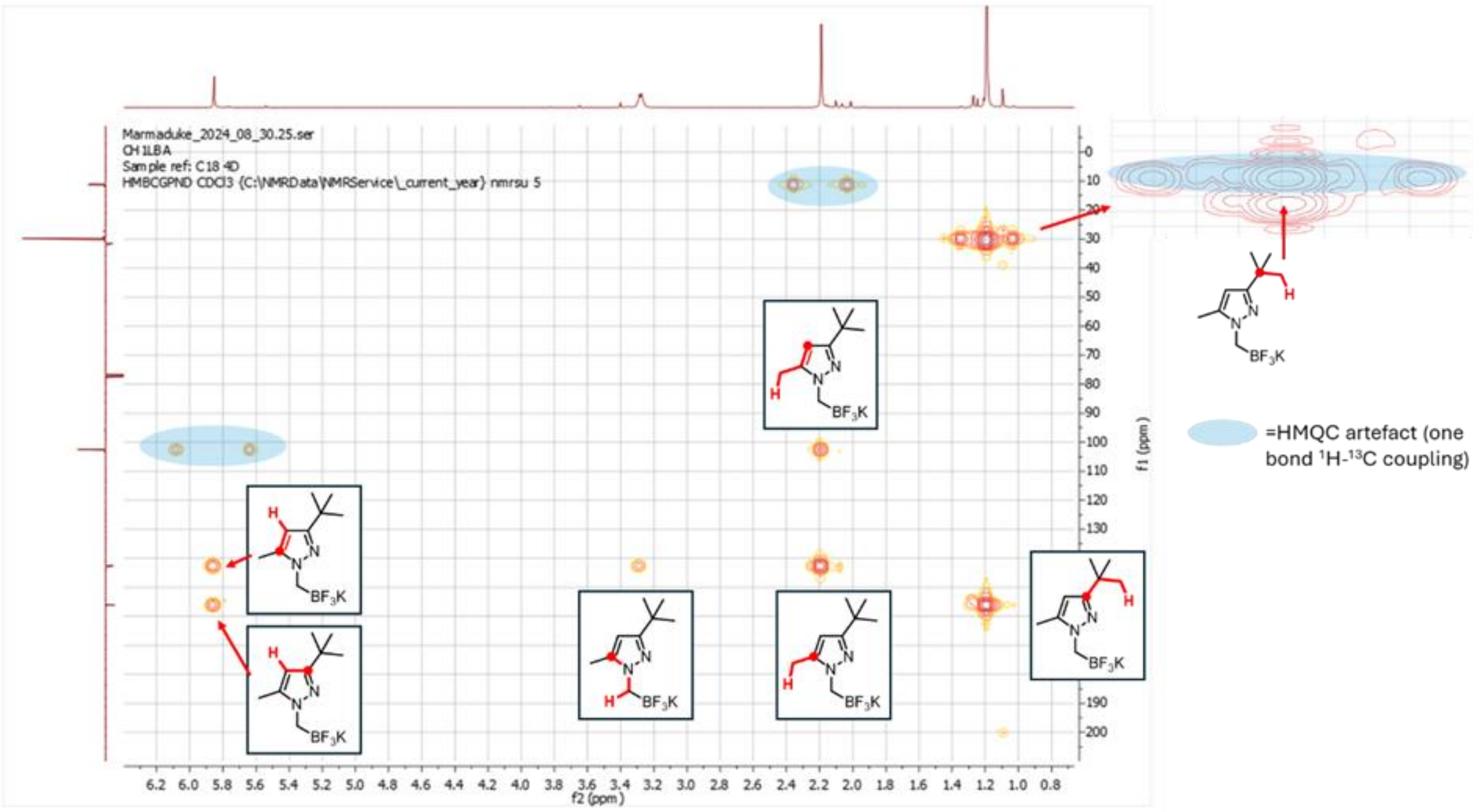

Potassium 3-carboxymethyl 5-methyl(trifluoro(1-methylpyrazolyl)borate (18)

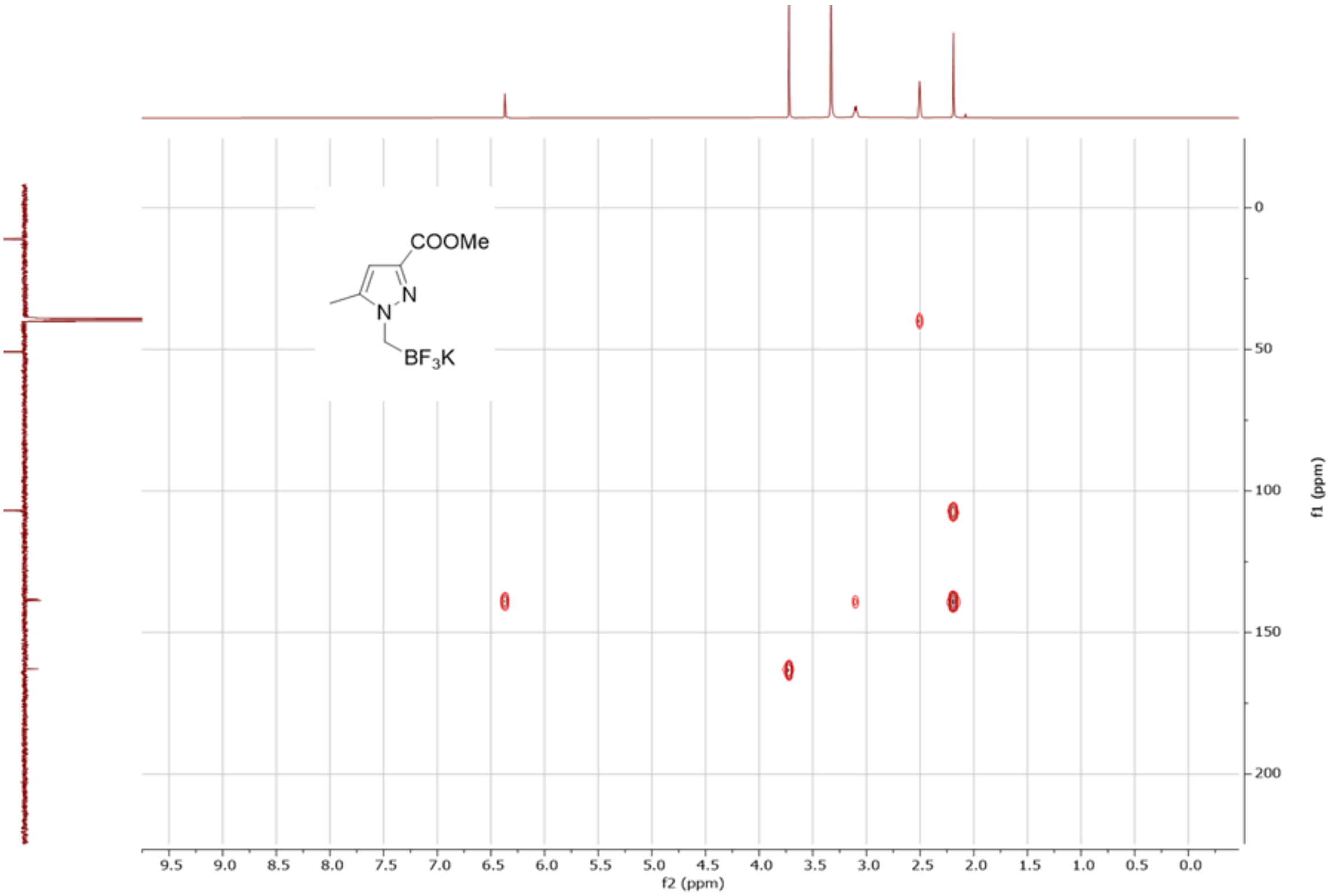

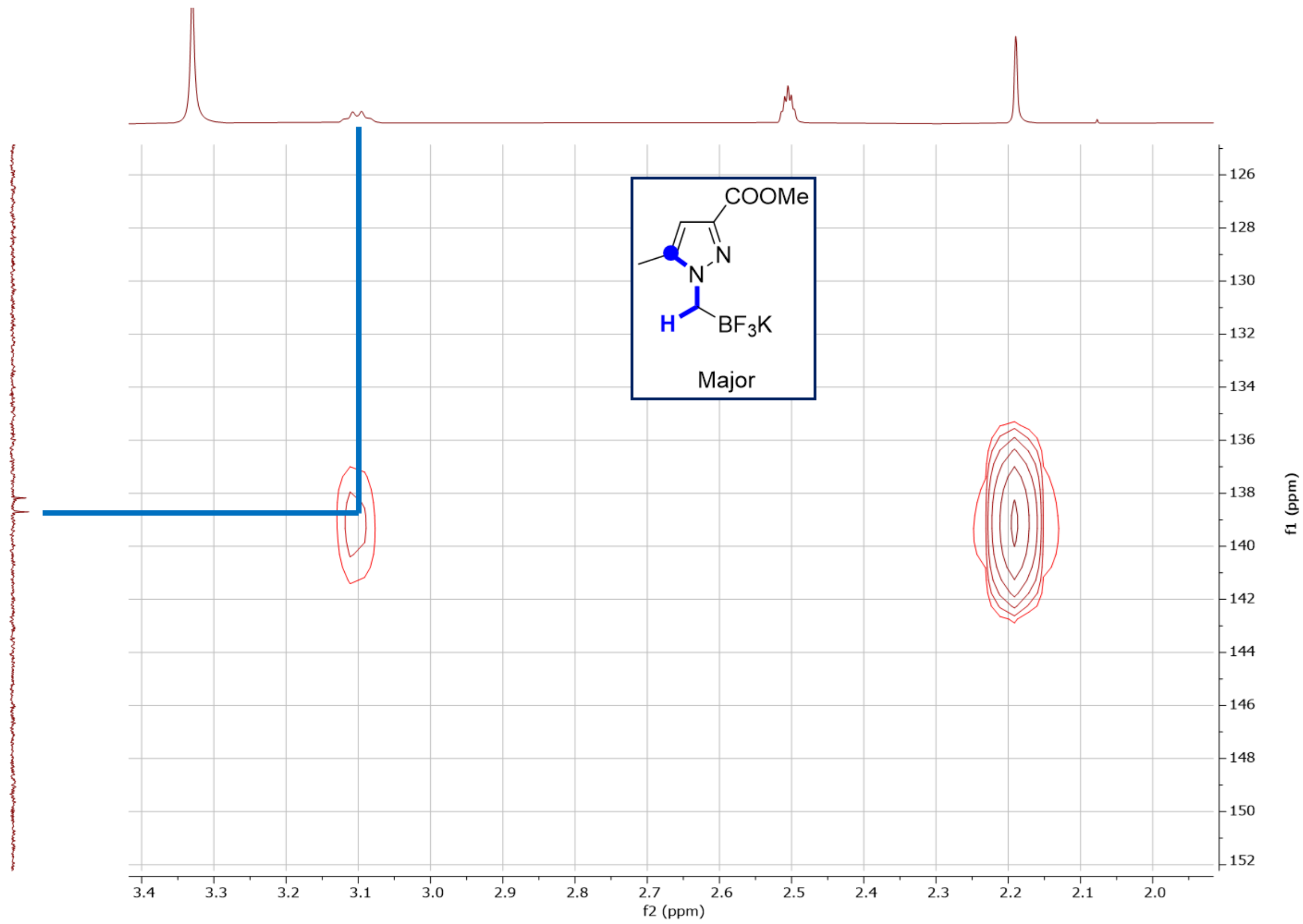
COOMe
N
N
H
BF3K
Major
f2 (ppm)
f1 (ppm)

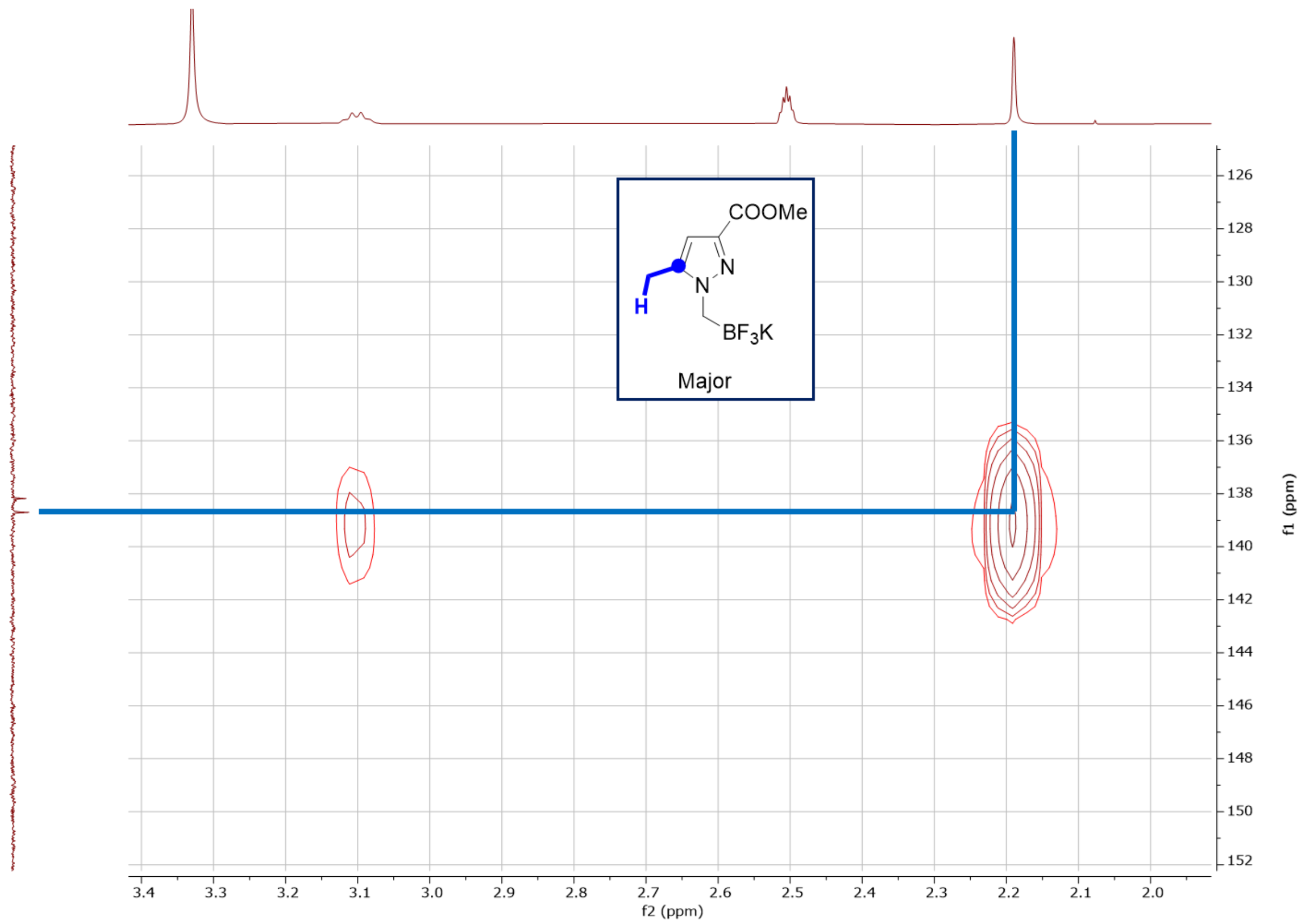
COOMe
N
N
H
BF3K
Major
f2 (ppm)
f1 (ppm)

Trifluoro((3-methyl-4,5,6,7-tetrahydro-2H-indazol-1-ium-2-yl)methyl)borate (19 major) and trifluoro((3-methyl-4,5,6,7-tetrahydro-1H-indazol-2-ium-1-yl)methyl)borate (19 minor)

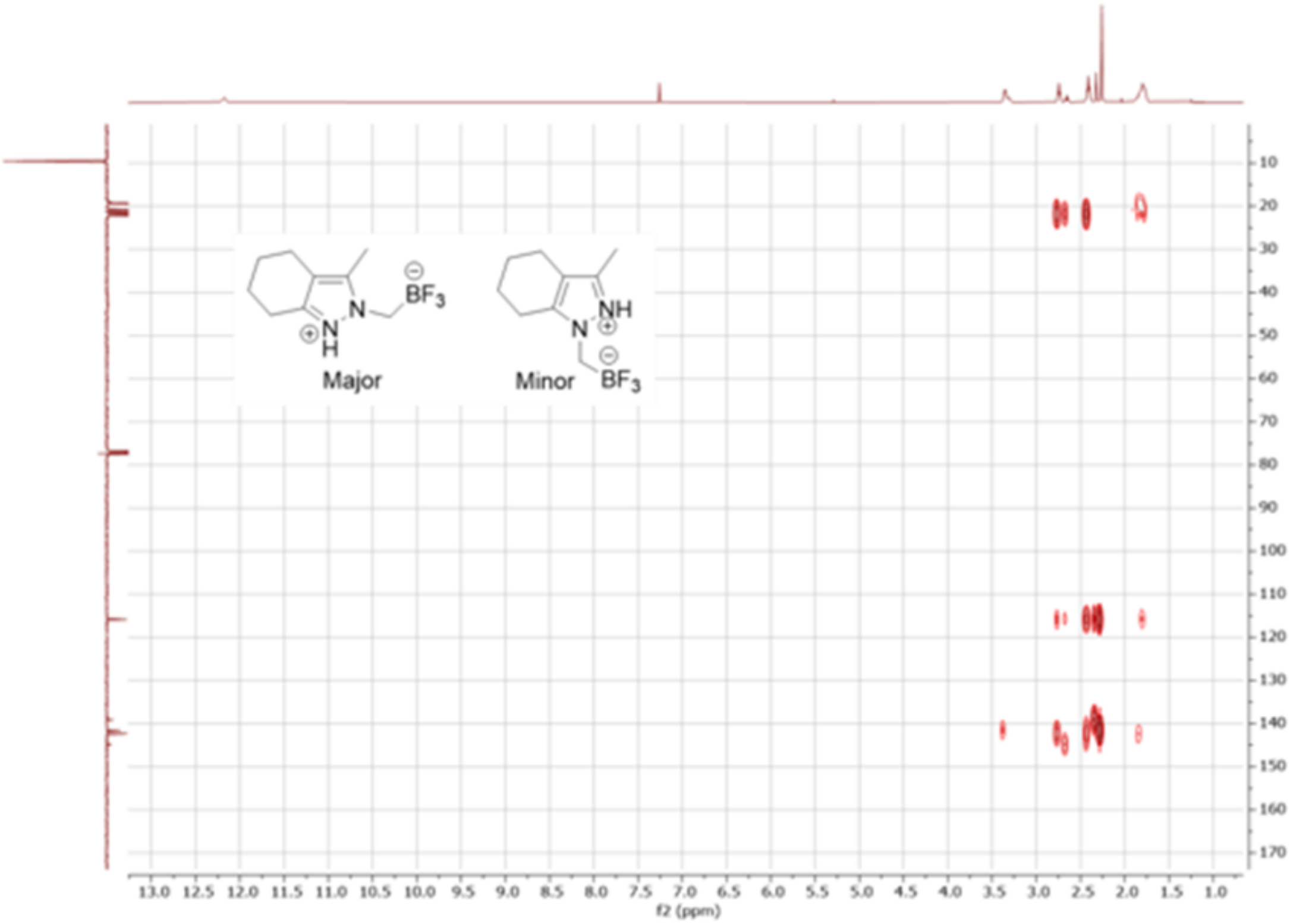

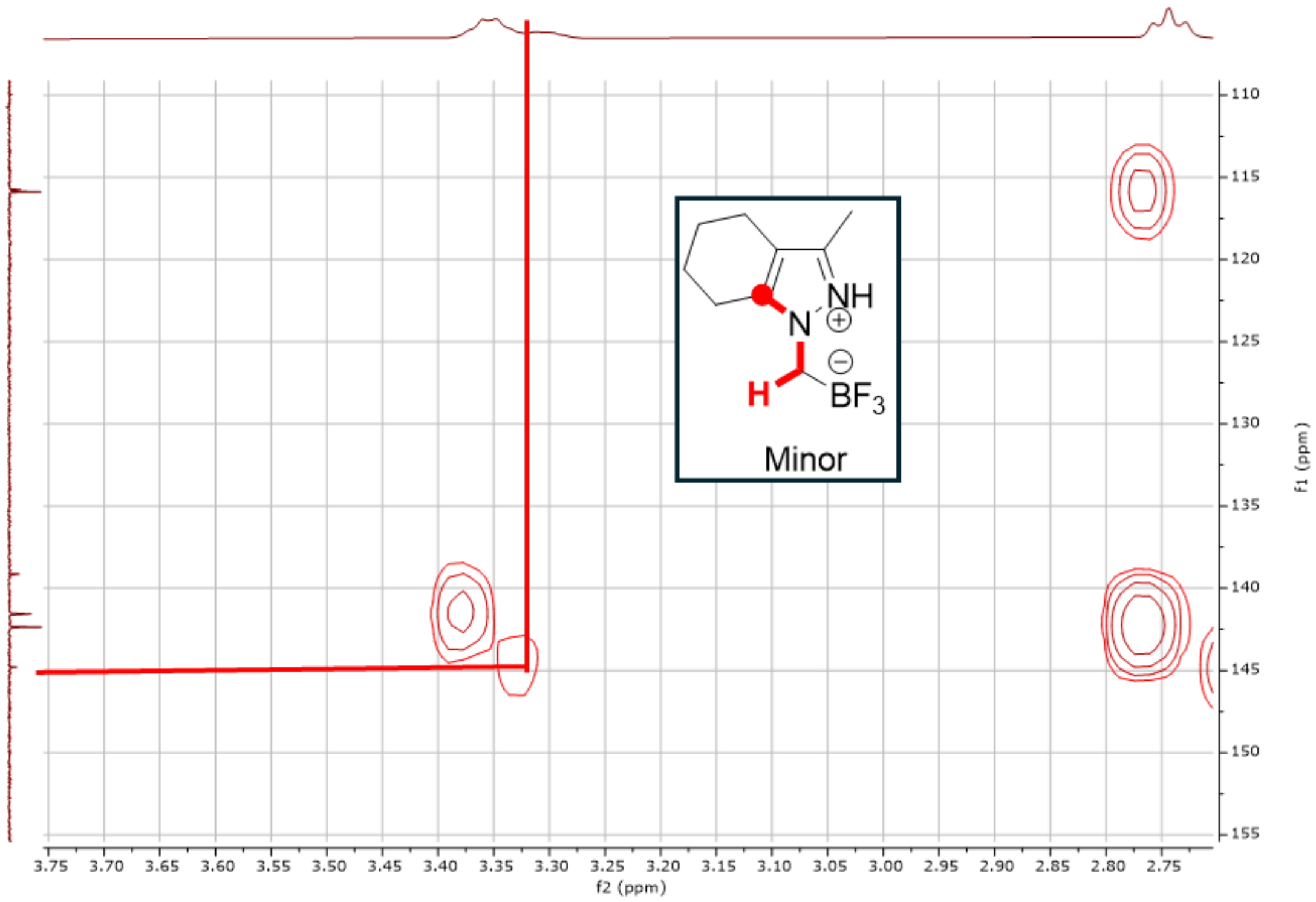
NH
N
BF3
H
Minor
f2 (ppm)
f1 (ppm)

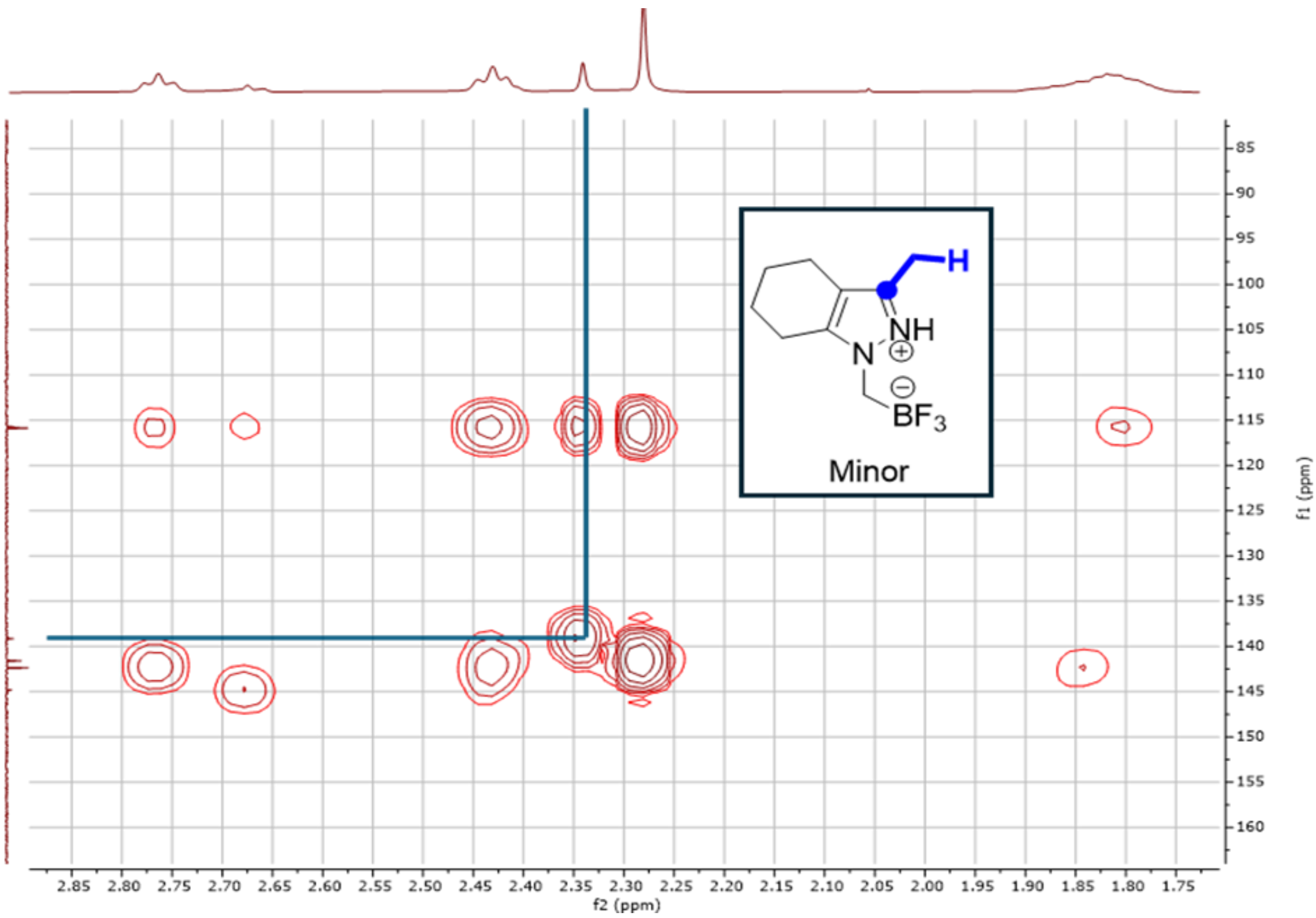
H
NH
N
BF3
Minor
f2 (ppm)
f1 (ppm)

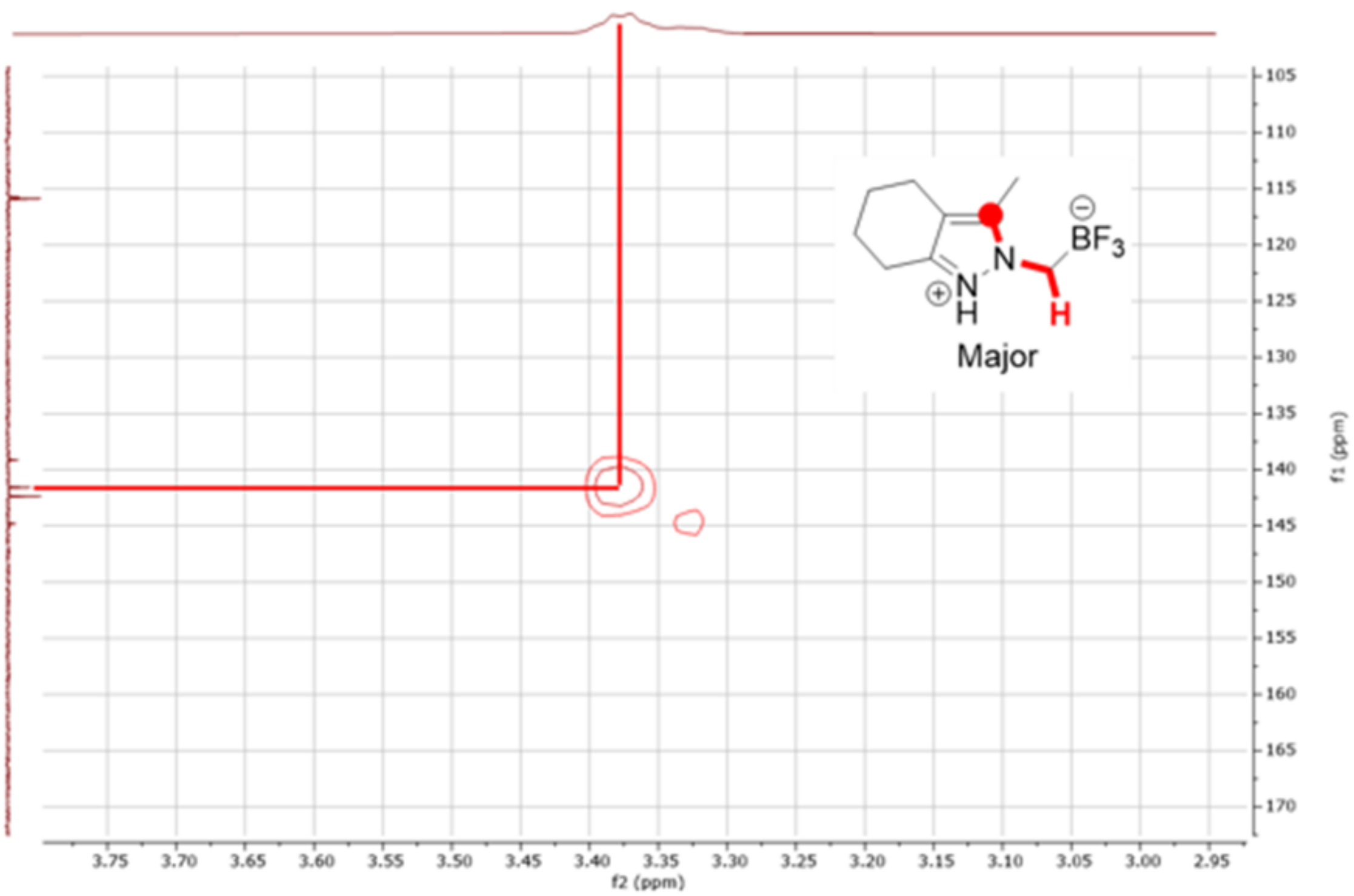
BF3
N
N
H
H
Major
f2 (ppm)
f1 (ppm)

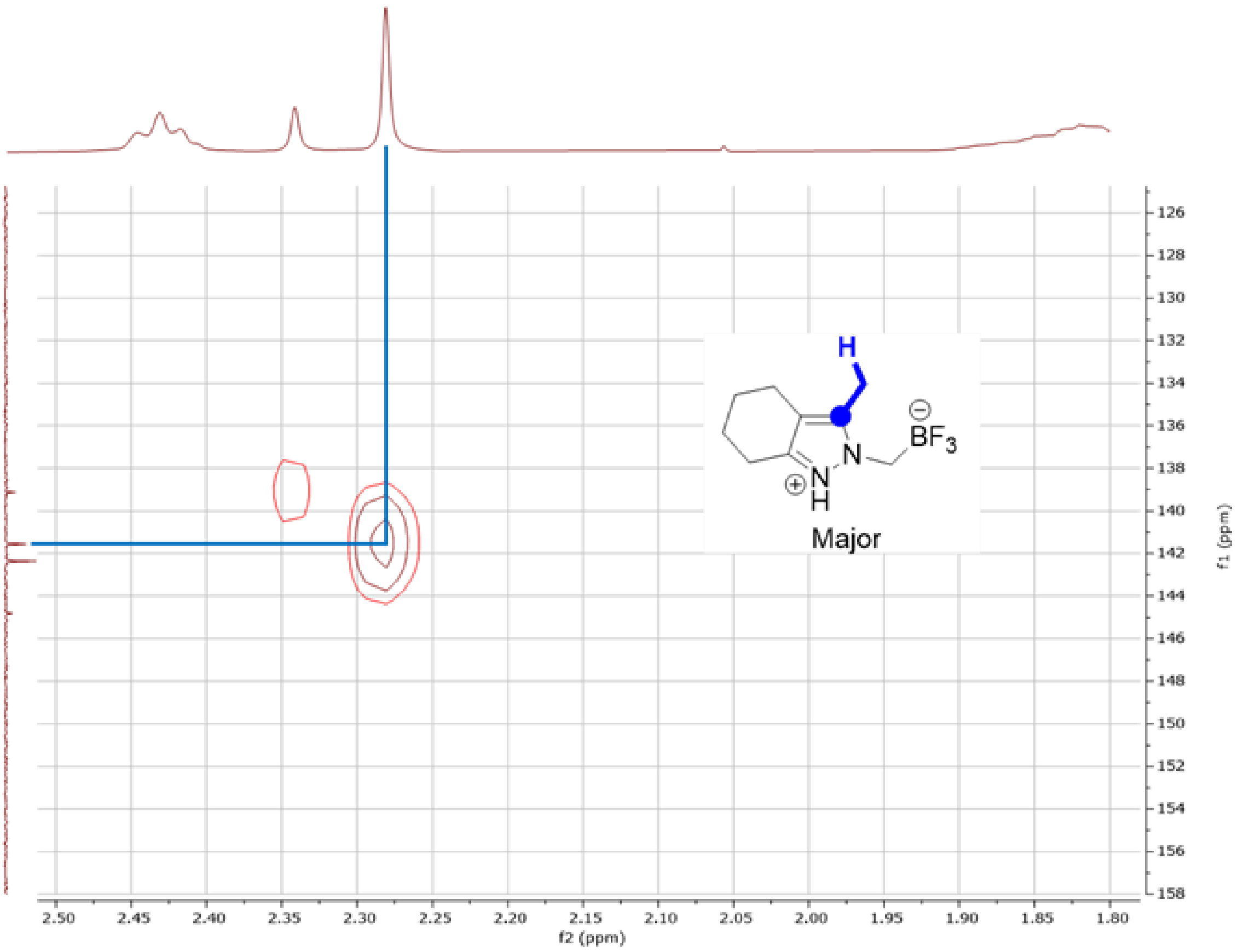
H
BF3
N
N
H
Major
f2 (ppm)
f1 (ppm)

3-Methyl(trifluoro(1-methylpyrazolyl)borate (x) and 5-methyl(trifluoro(1-methylpyrazolyl)borate (21)

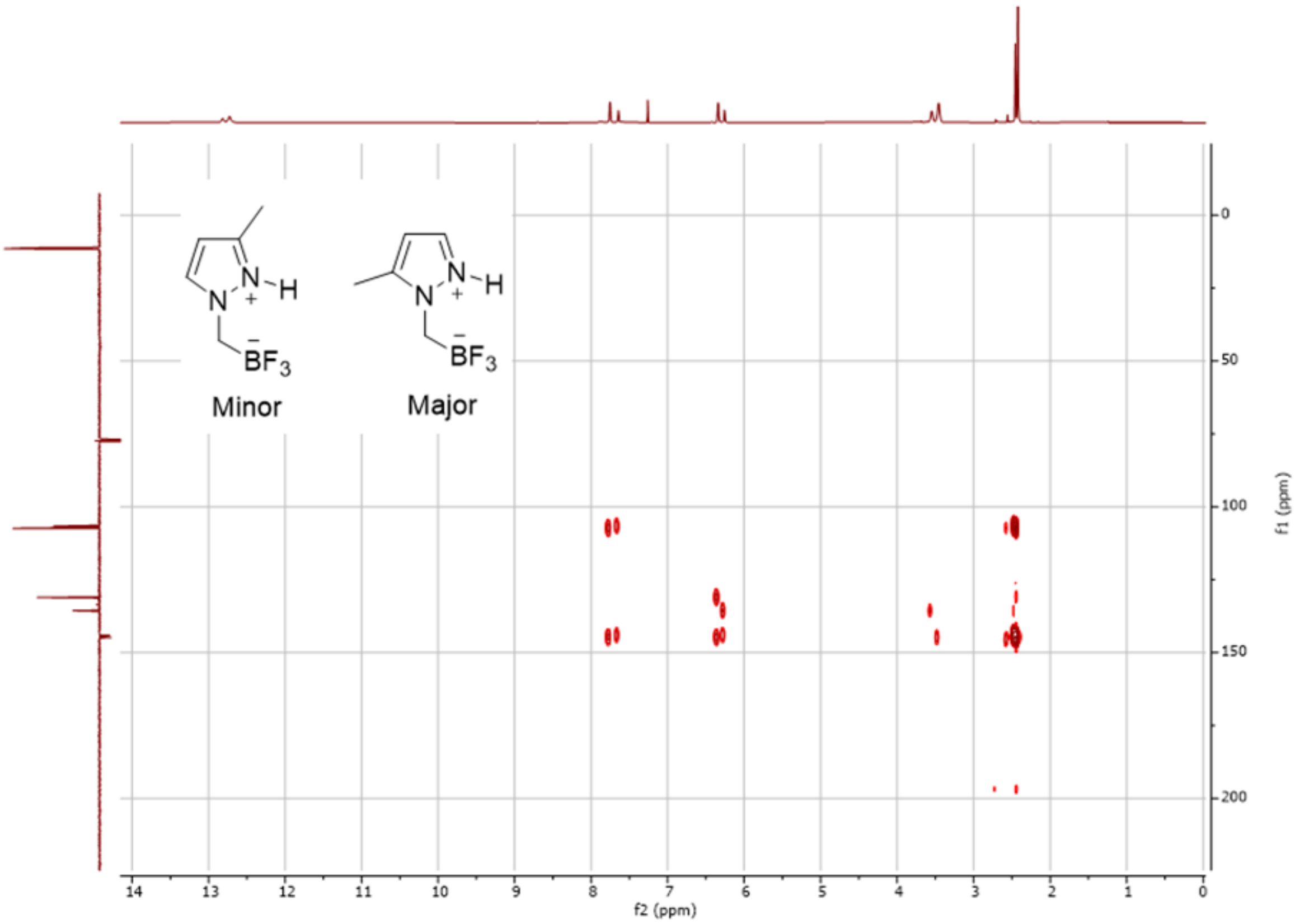

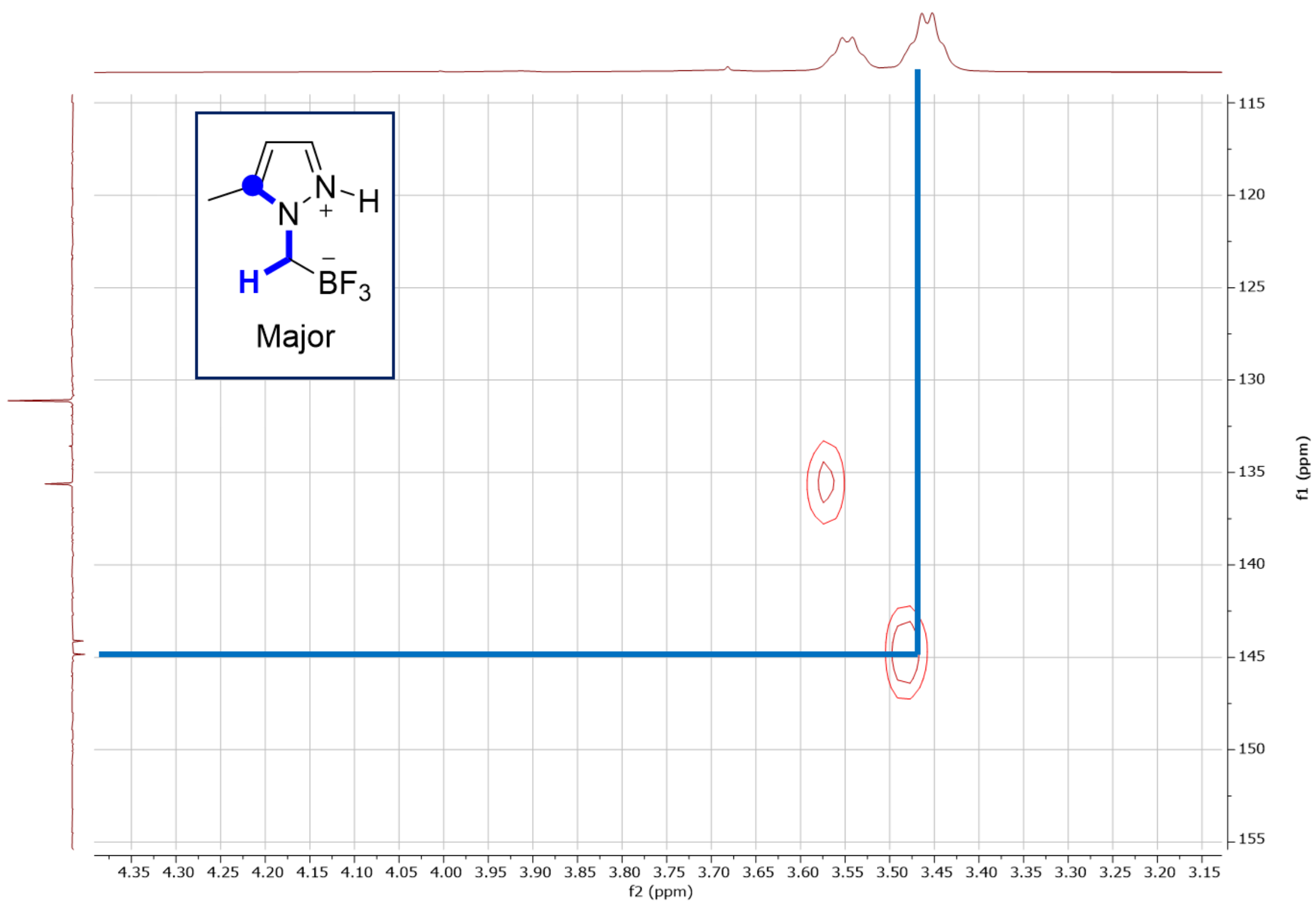
N
N
H
+
BF3
H
Major
f2 (ppm)
f1 (ppm)

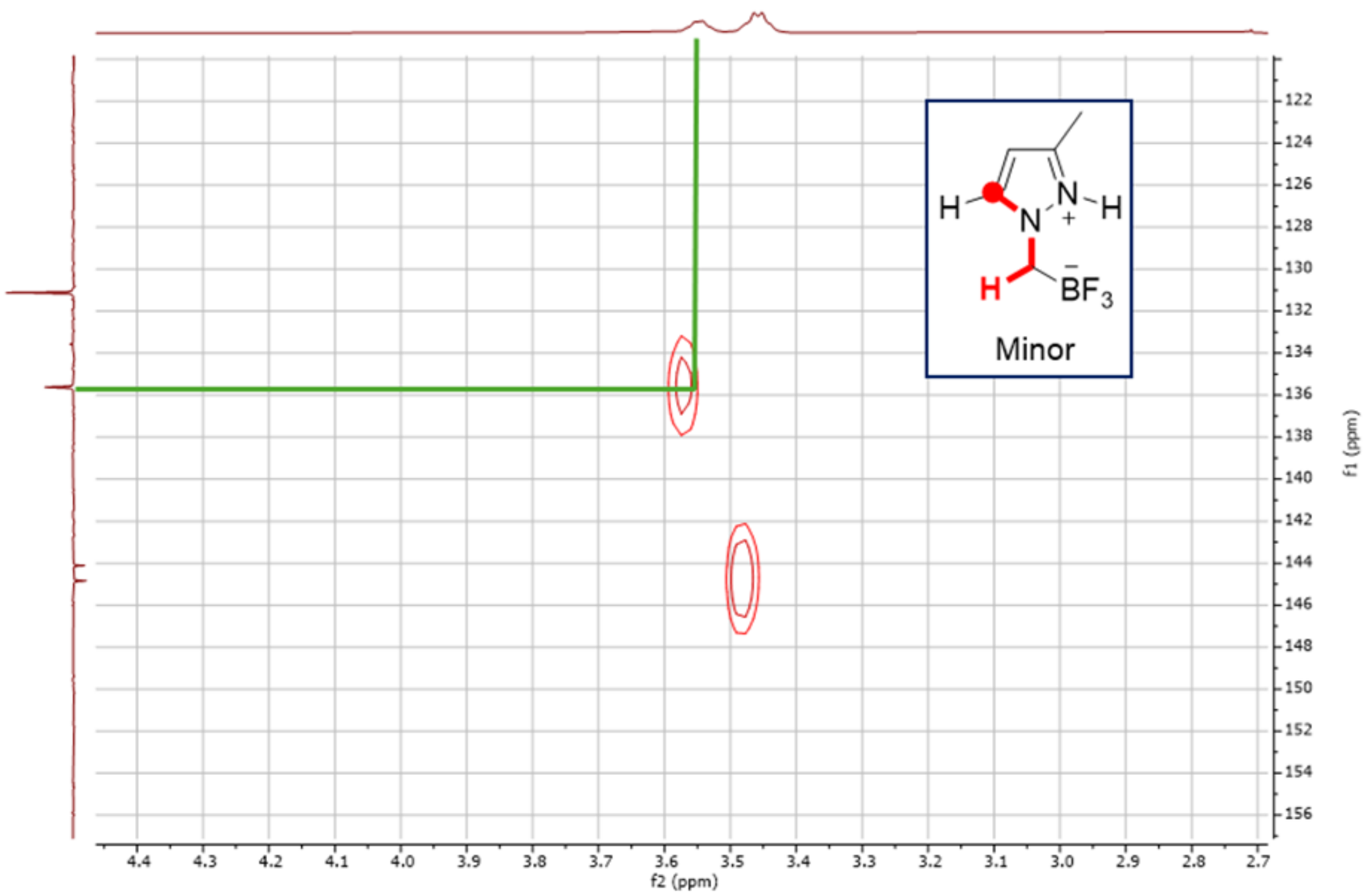
N
N
H
H
+
BF3
H
Minor
f2 (ppm)
f1 (ppm)

Potassium 3-methyl 5-ethyl-(trifluoro(1-methylpyrazolyl)borate (25)

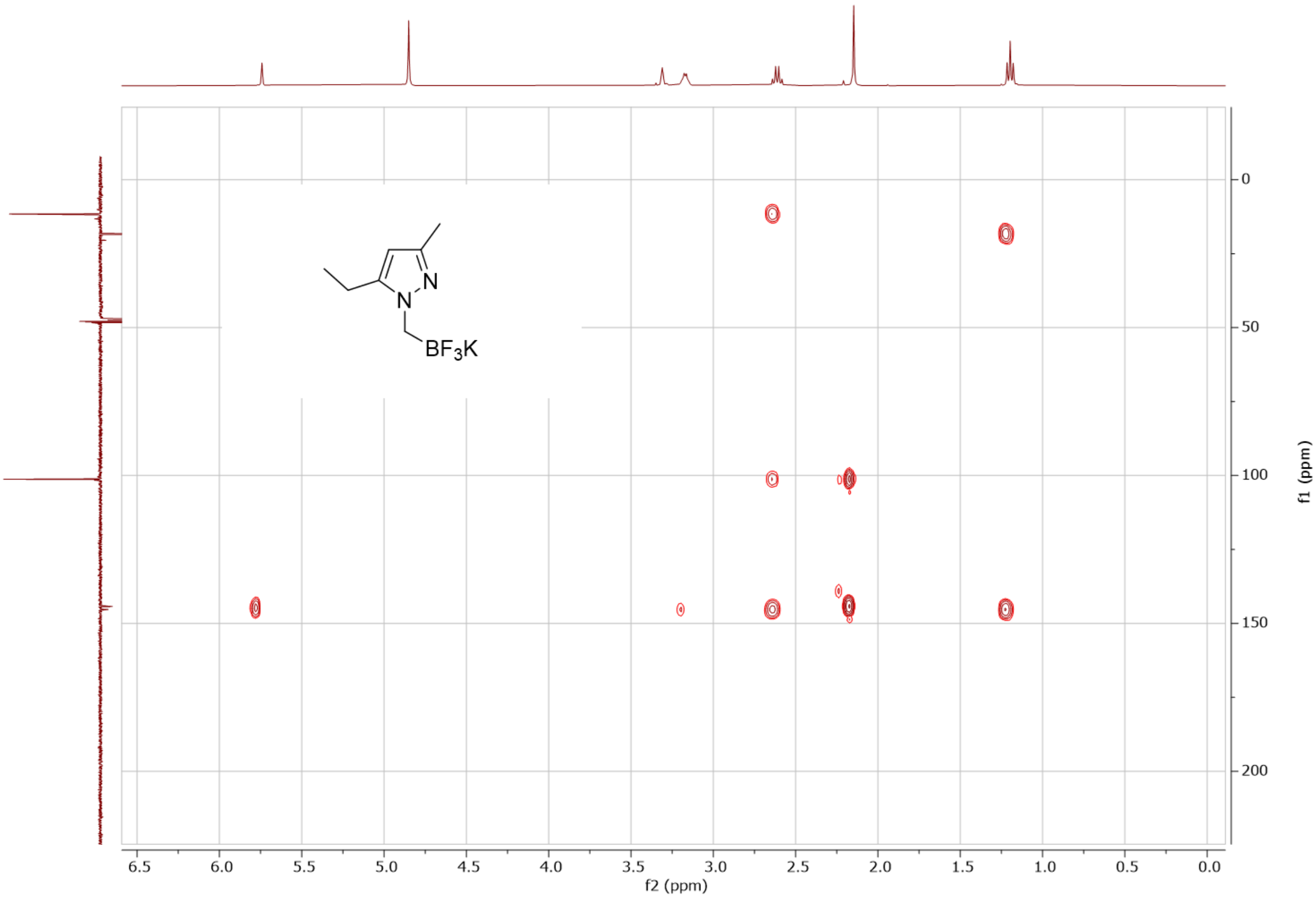

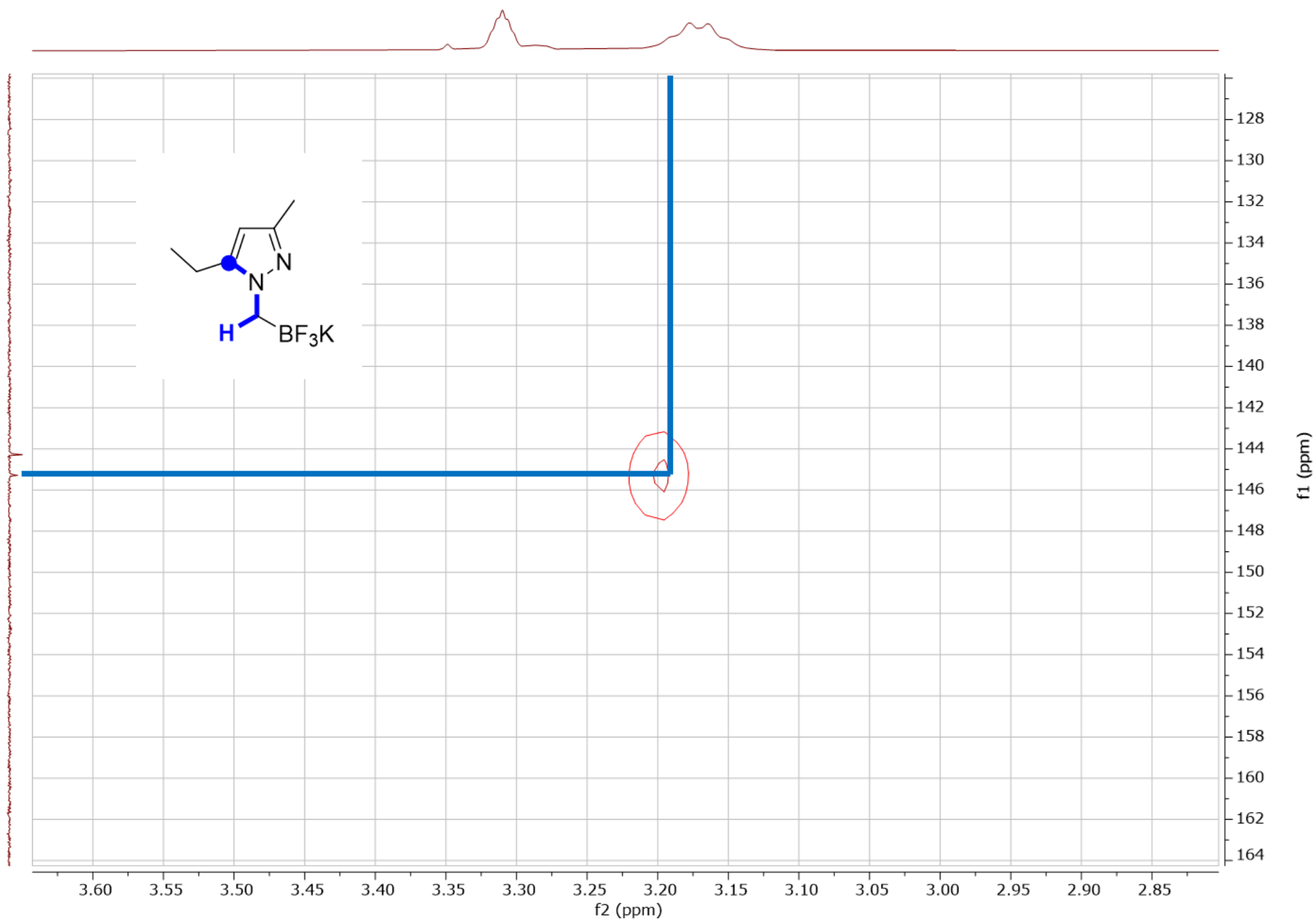
H
BF3K
N
N
f2 (ppm)
f1 (ppm)

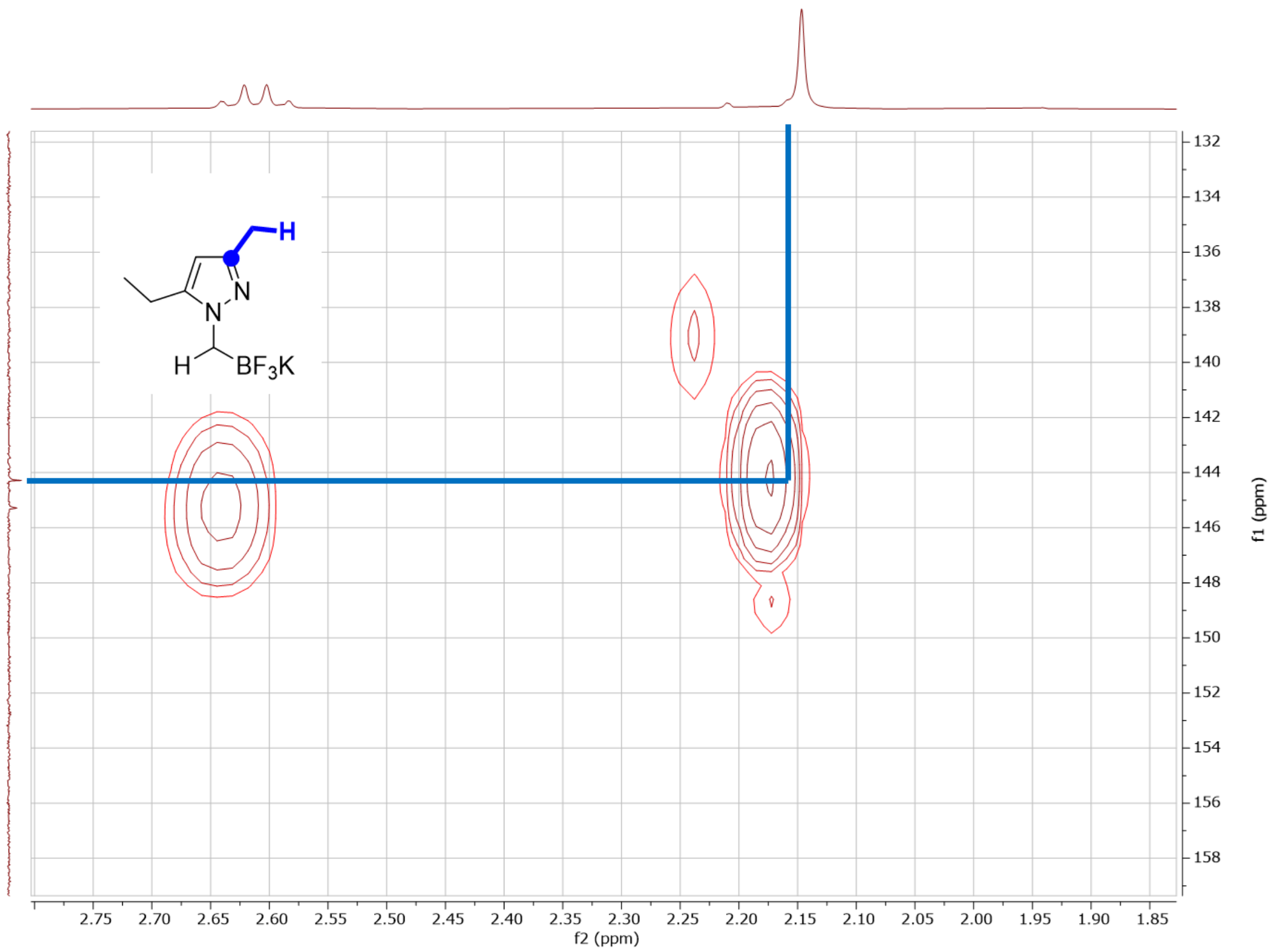
H
H
BF3K
N
N
f2 (ppm)
f1 (ppm)

Potassium 3-phenyl 5-cyclopropyl-(trifluoro(1-methylpyrazolyl)borate (28)

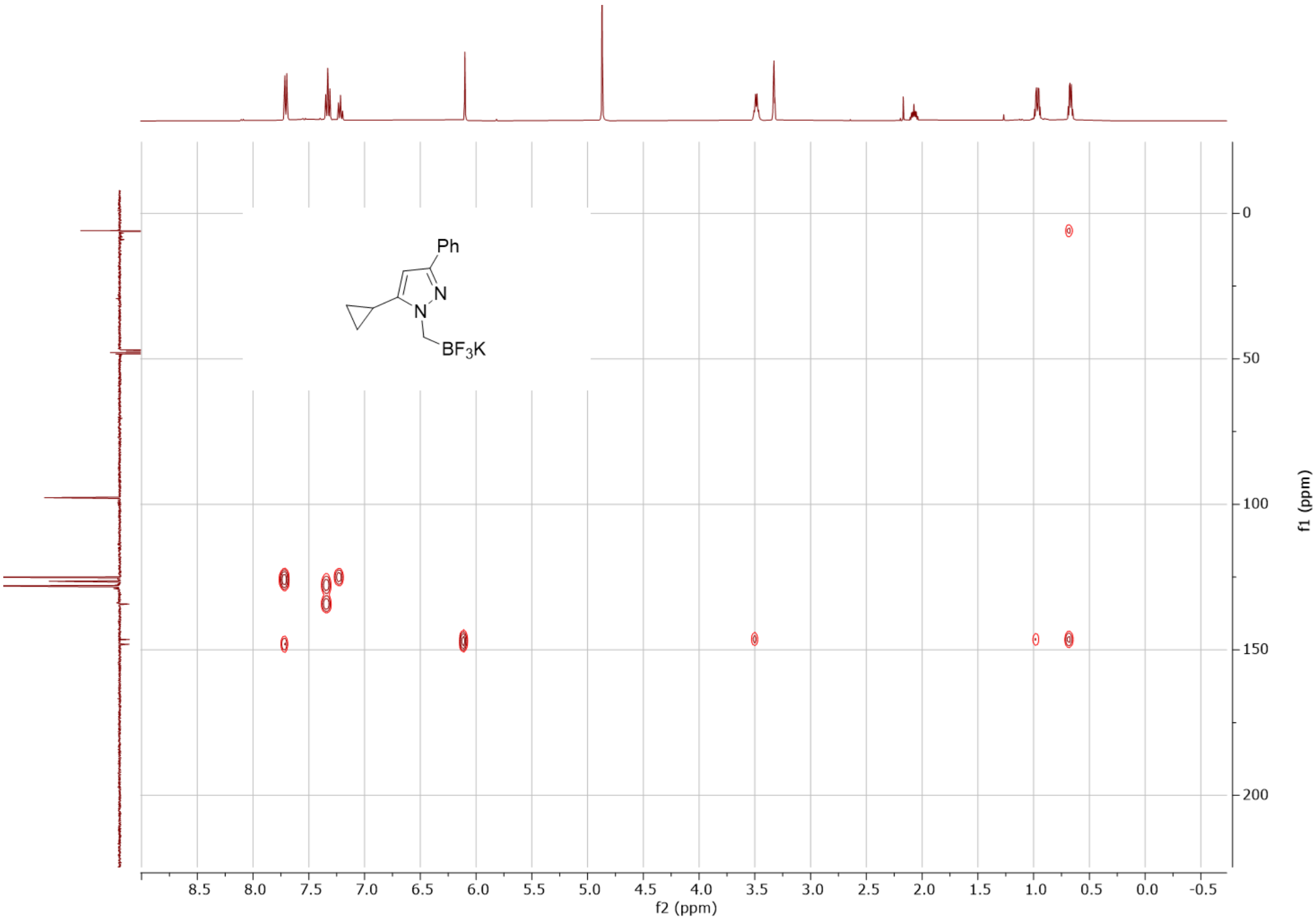

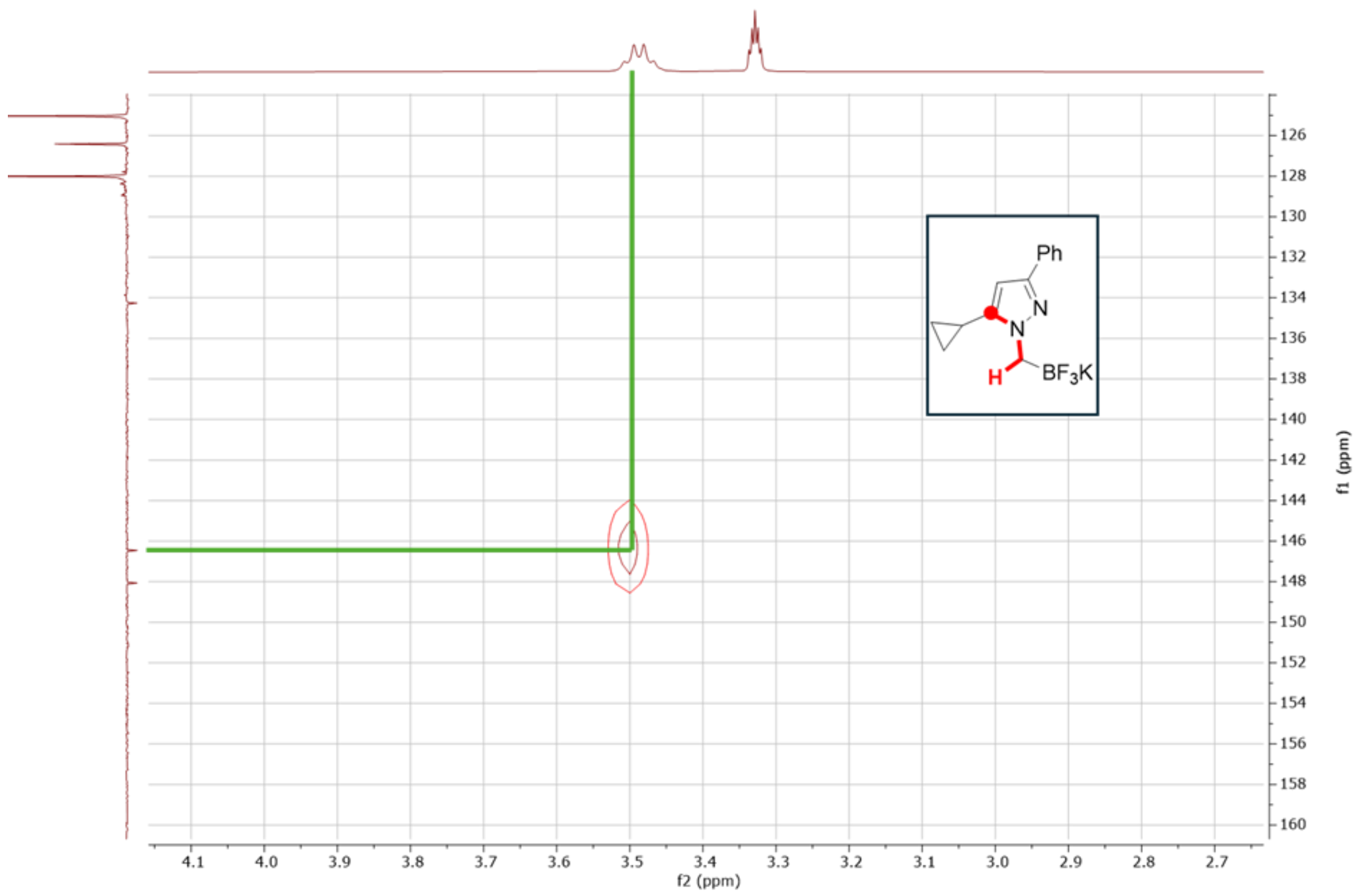
Ph
N
N
BF3K
H
f2 (ppm)
f1 (ppm)

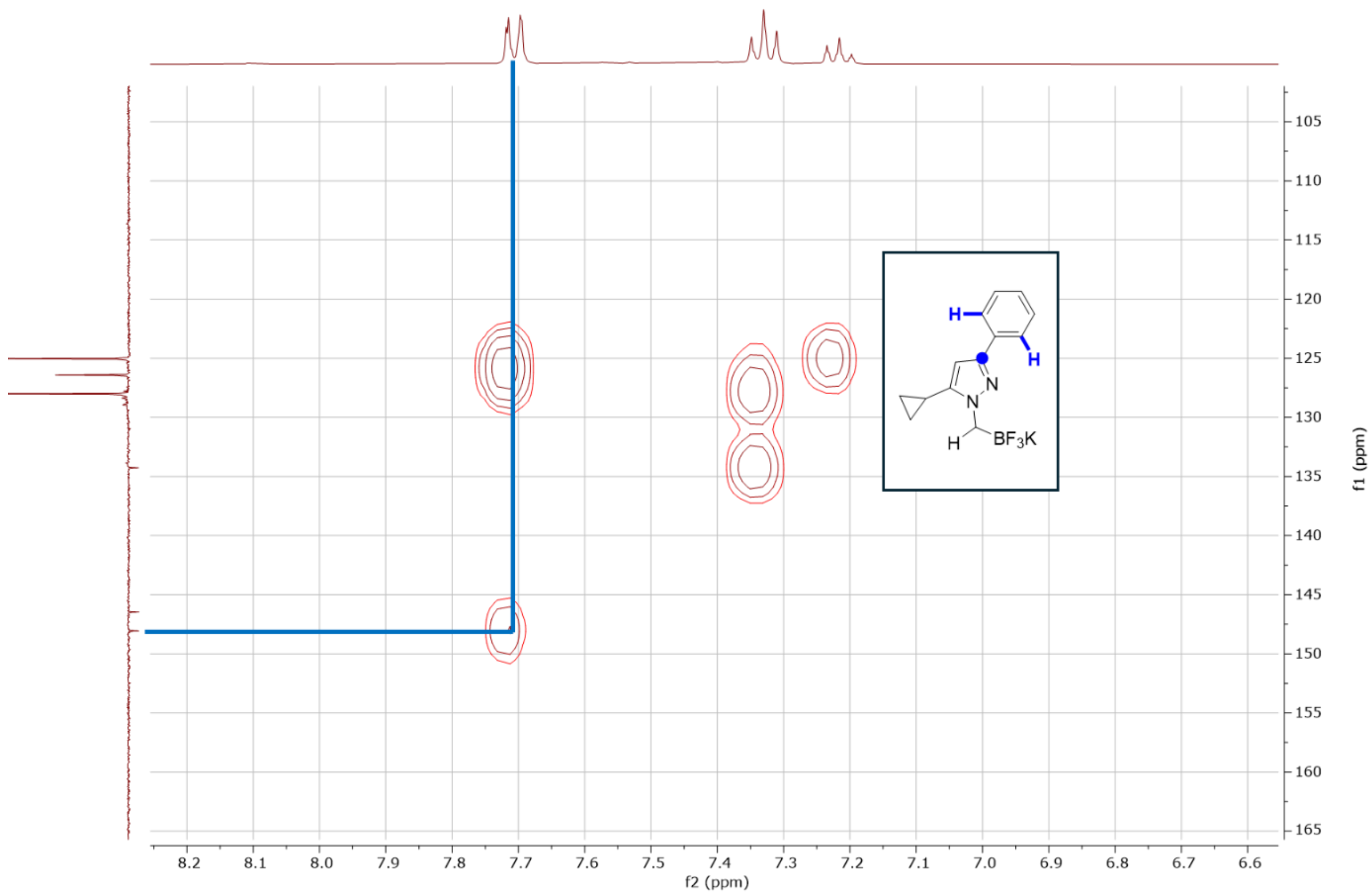
H
H
N
N
H
BF3K
f2 (ppm)
f1 (ppm)

Potassium 3-phenyl 5-n-butyl-(trifluoro(1-methylpyrazolyl)borate (29)

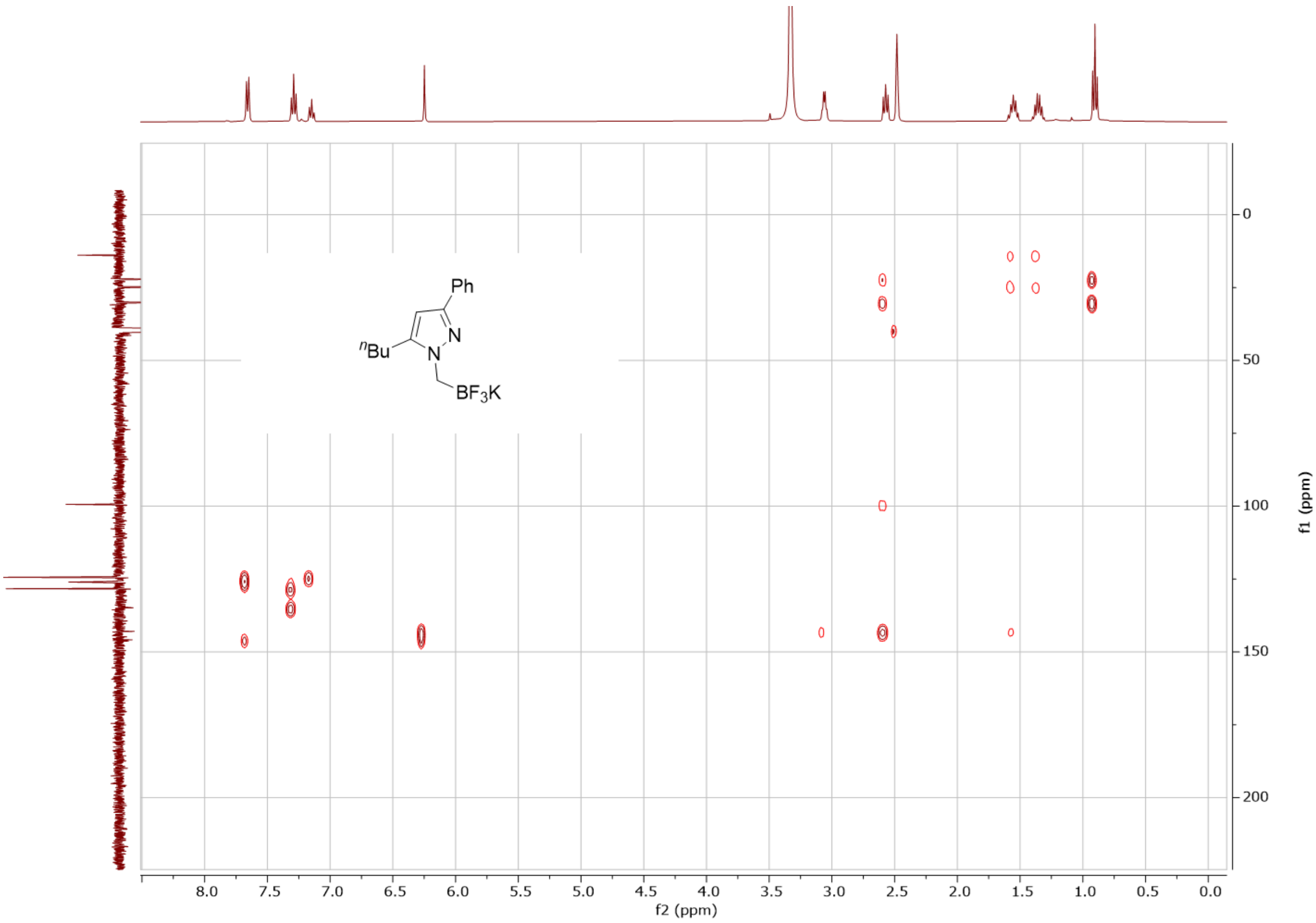

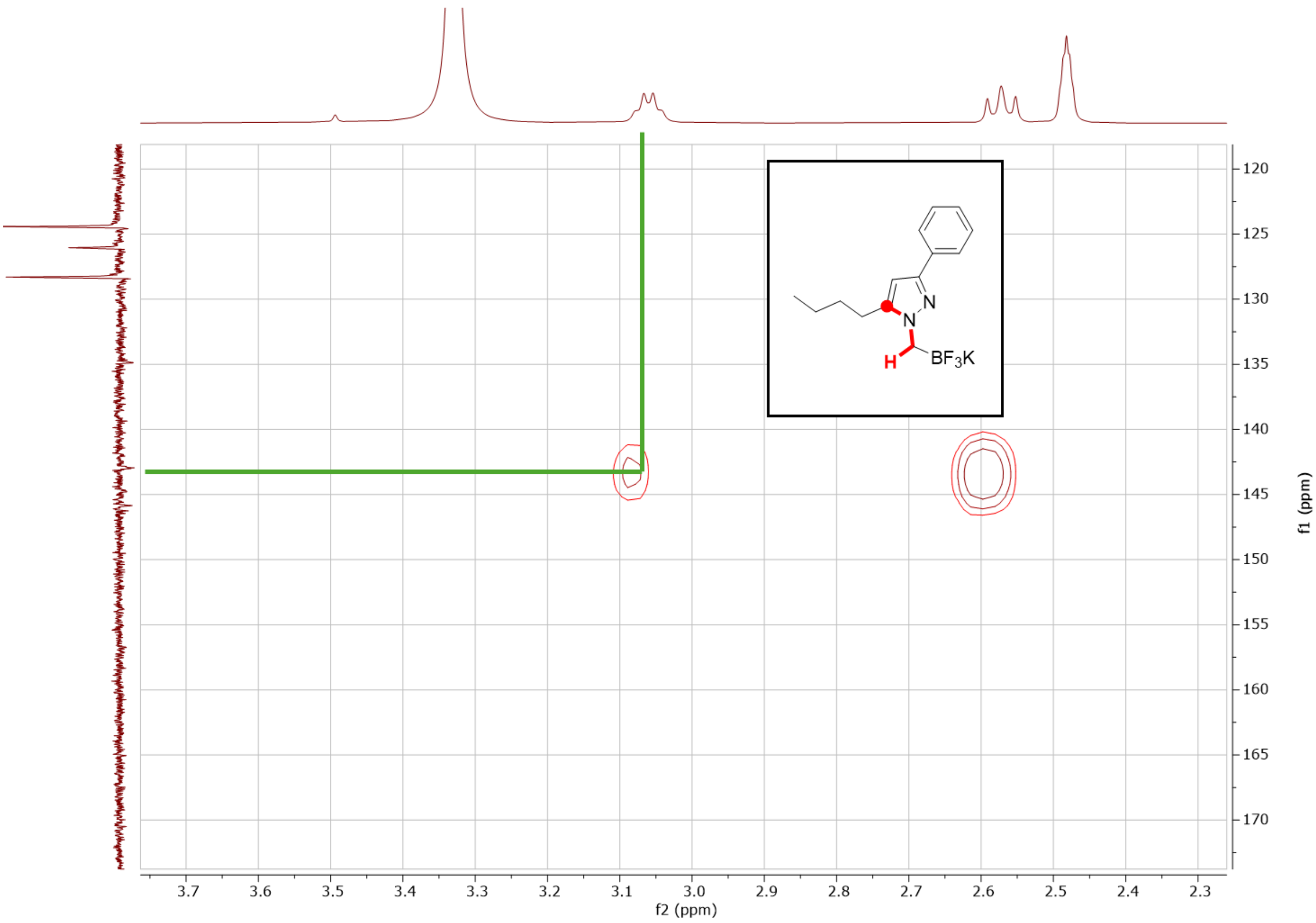

N
N
H
BF3K
f2 (ppm)
f1 (ppm)

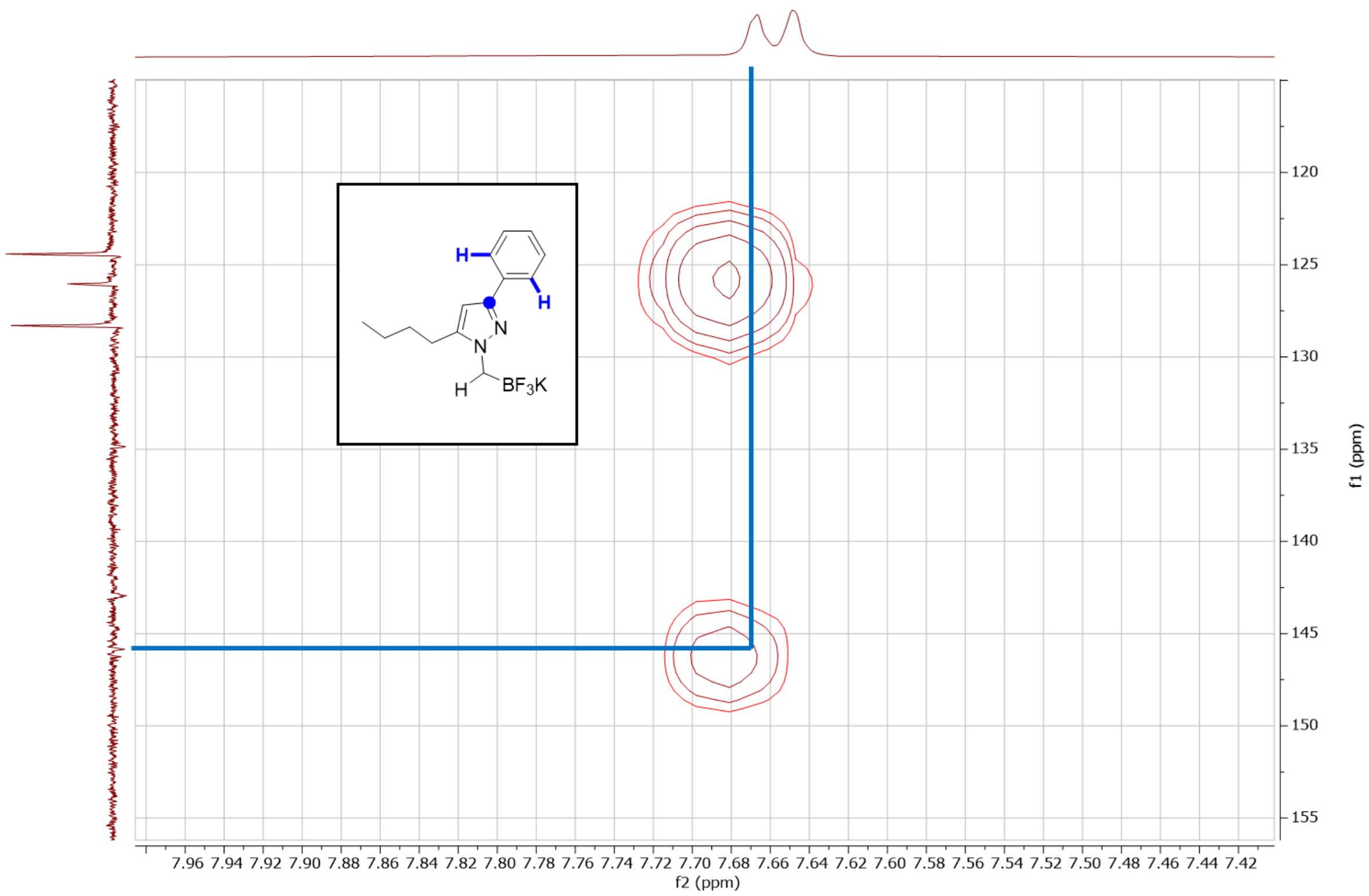

H
H
N
N
H
BF3K
f2 (ppm)
f1 (ppm)

3-Methyl(trifluoro(1-methylpyrazolyl)borate (30)

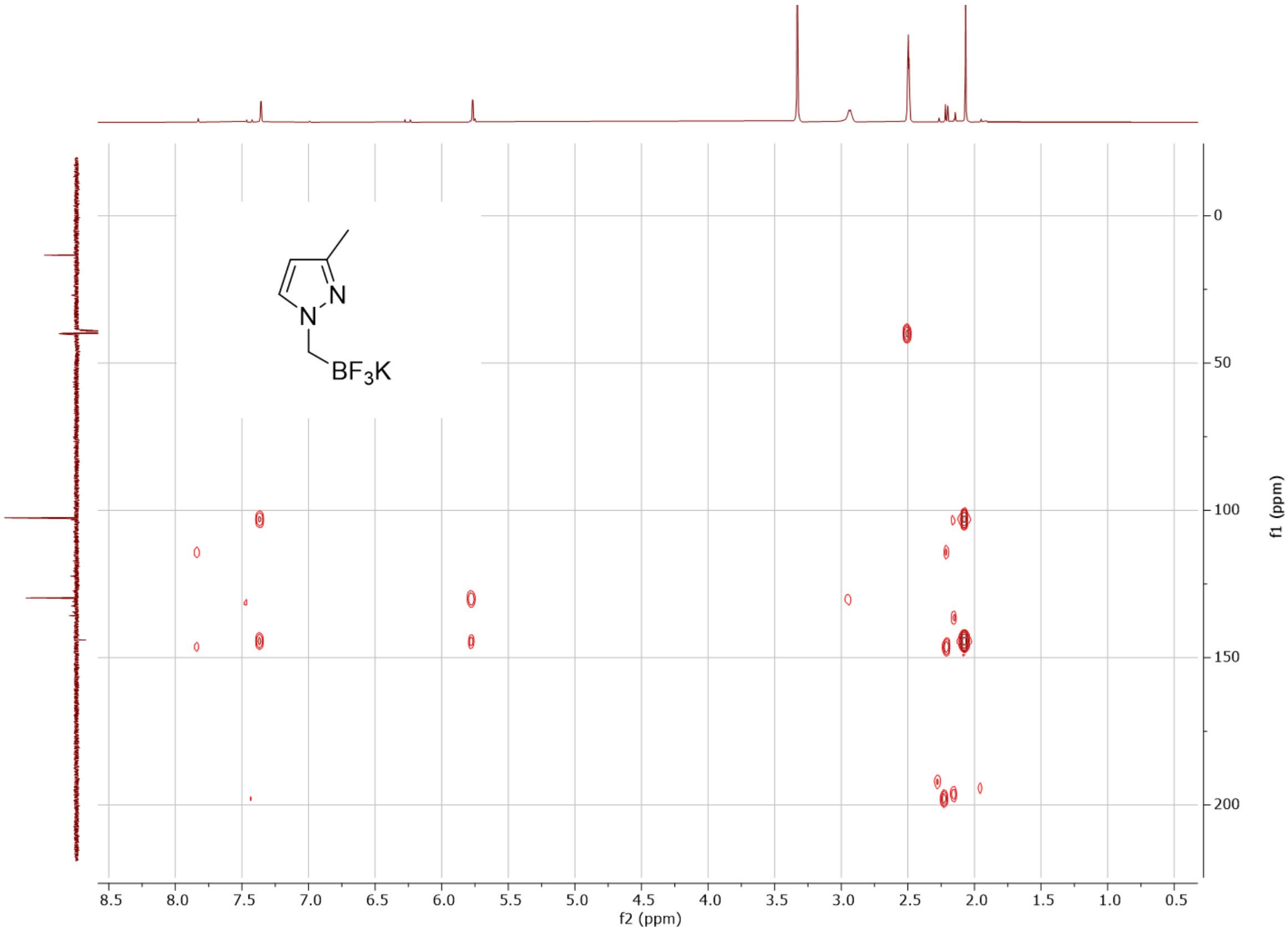

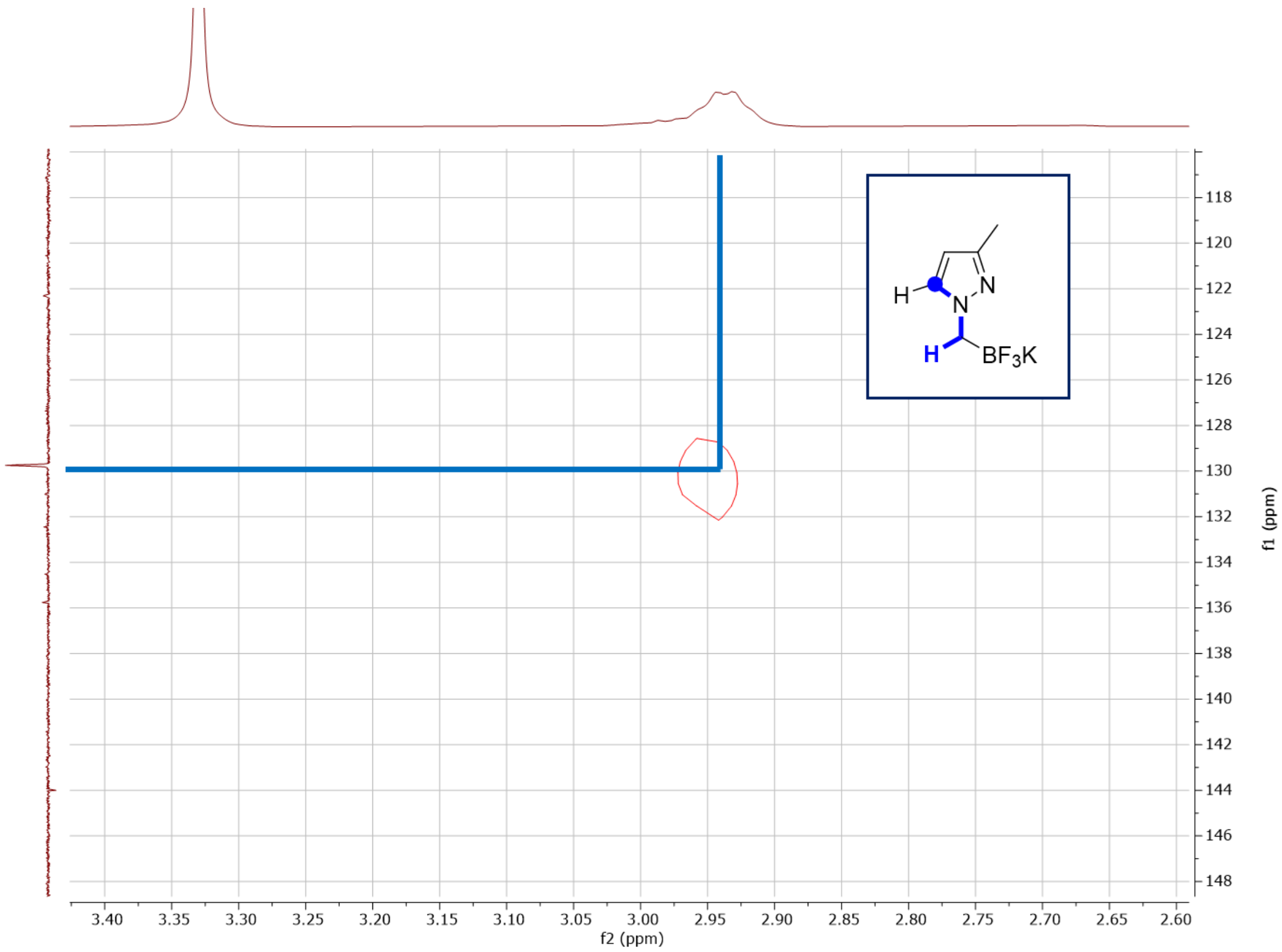
H
N
N
H
BF3K
f2 (ppm)
f1 (ppm)

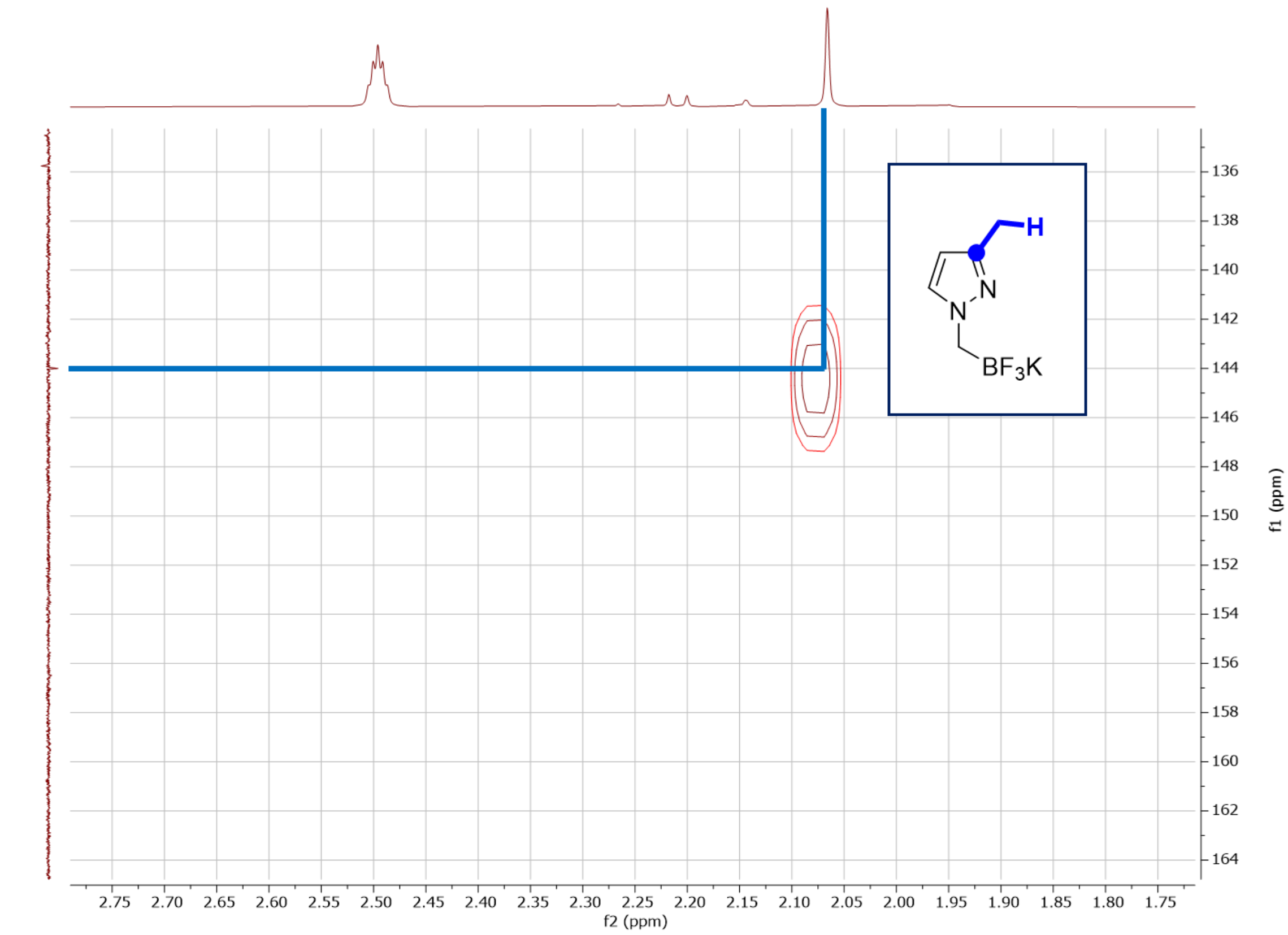
H
N
N
BF3K
f2 (ppm)
f1 (ppm)

Regiochemistry characterisation of compounds 26 and 27

Regioisomeric pyrazoles **26** and **27** were each formed in a ratio of ~85:15. The presence of major/minor isomeric mixtures was apparent by ^{13}C NMR spectroscopy and this technique was used to estimate regioisomer ratios according to the method of Woerpel.[1]

Comparison of $^{13}C\{^{1}H\}$ spectra of aliphatic methylene groups in regiosomers

Minor regiosomer signals in the top spectrum correspond to major signals in the bottom spectrum as indicated by red arrows. Minor regiosomer signals in the bottom spectrum correspond to major signals in the top spectrum as indicated by black arrows.

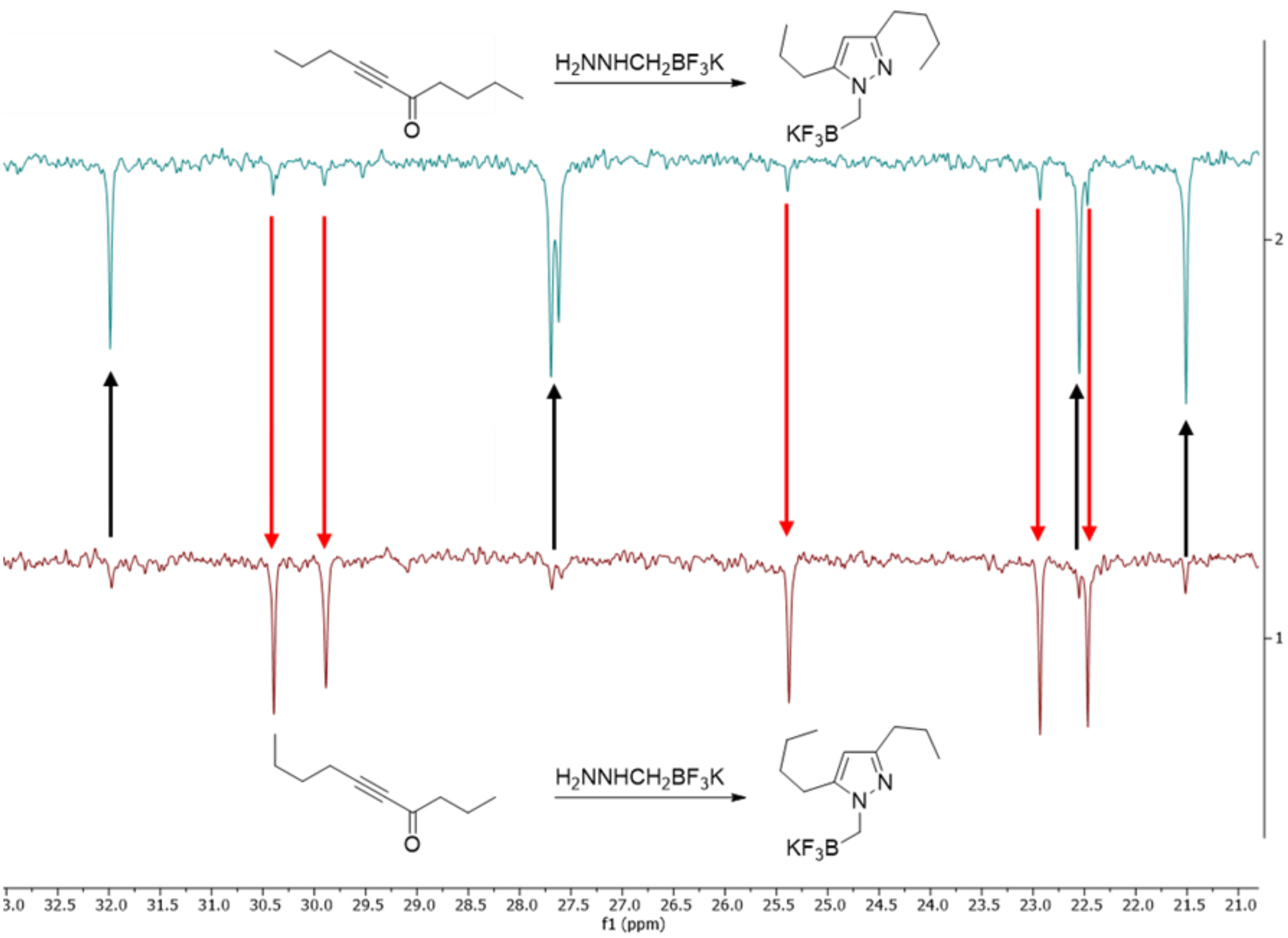

[1] D. A. L. Otte, D. E. Borchmann, C. Lin, M. Weck, K. A. Woerpel *Org. Lett.* **2014**, *16*, 1566–1569.

Integration of individual $^{13}C\{^{1}H\}$ spectra in 26 and 27

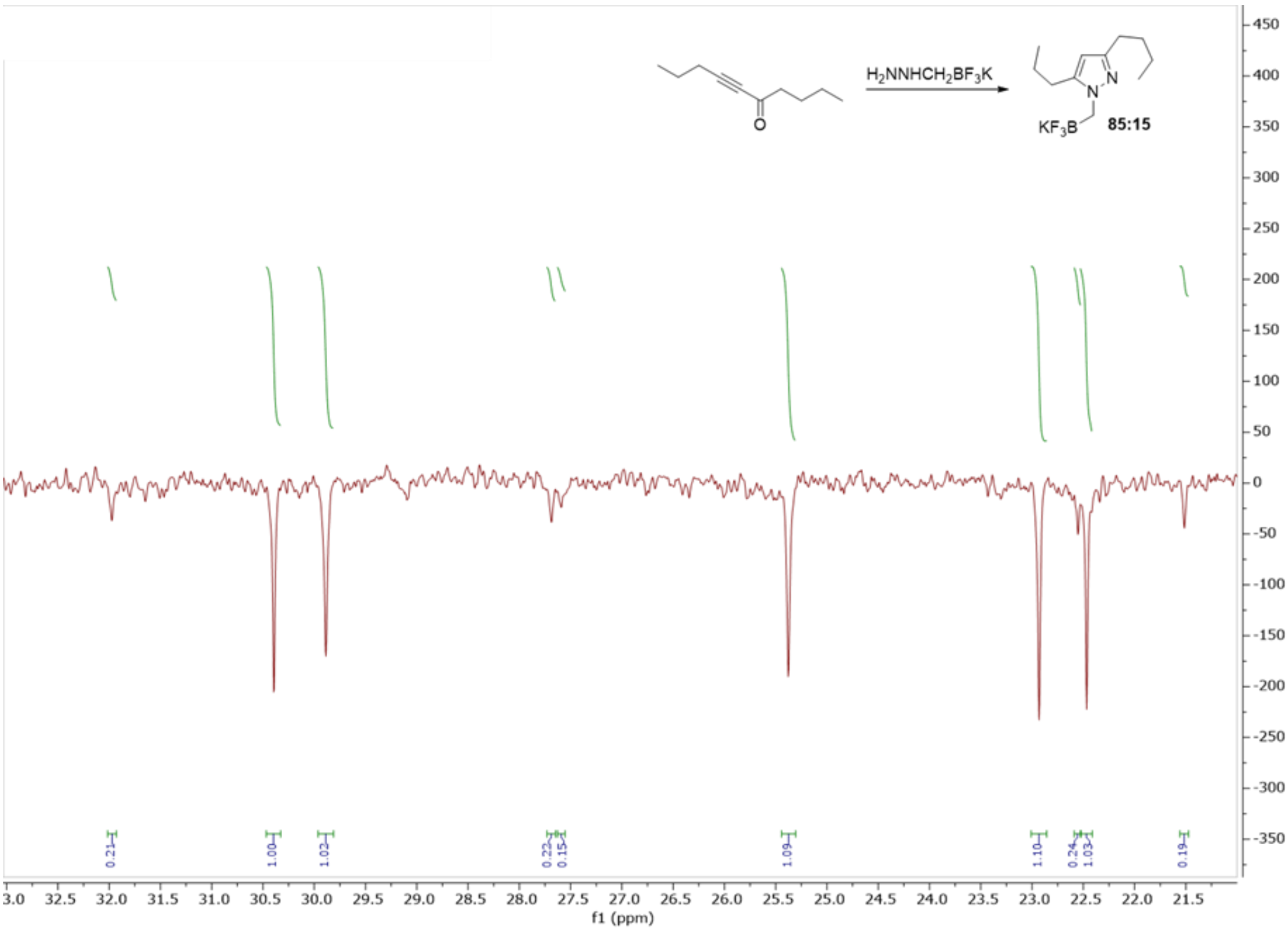

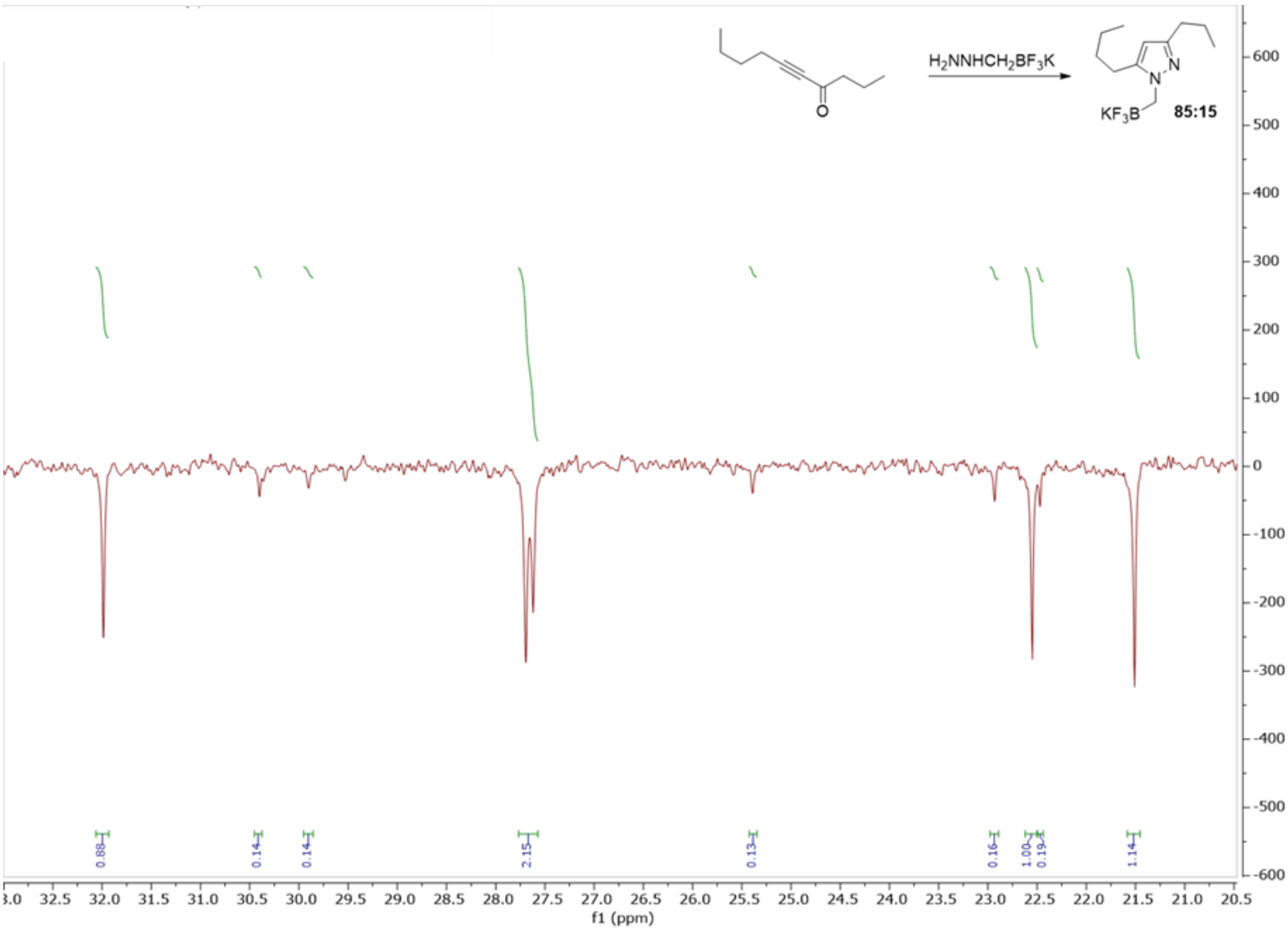

^{1}H-^{1}H COSY shows the connectivity of the ^{n}Pr and ^{n}Bu chains in pyrazole **26**:

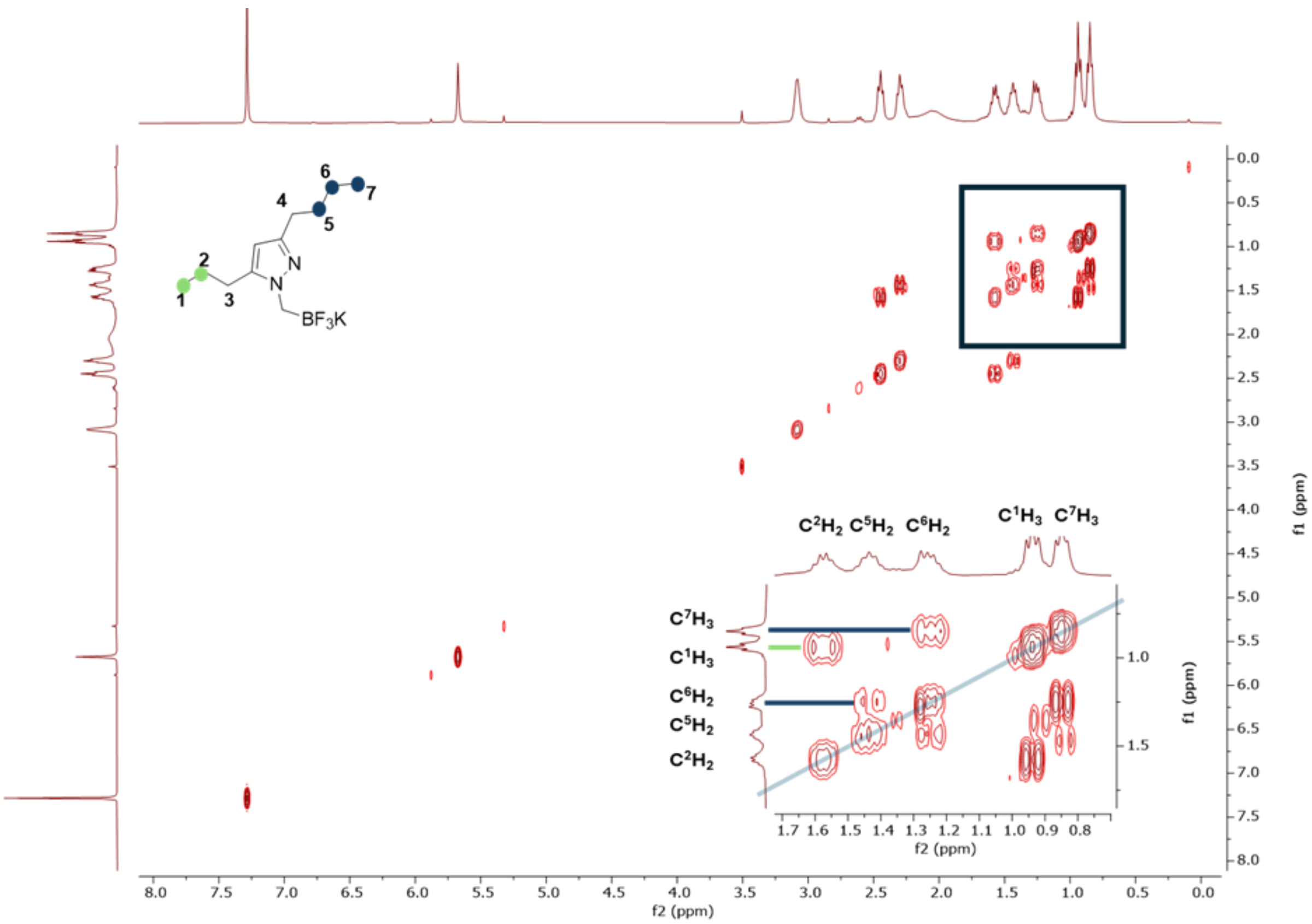

BF3K
C2H2 C5H2 C6H2
C1H3 C7H3
C7H3
C1H3
C6H2
C5H2
C2H2
f2 (ppm)
f1 (ppm)

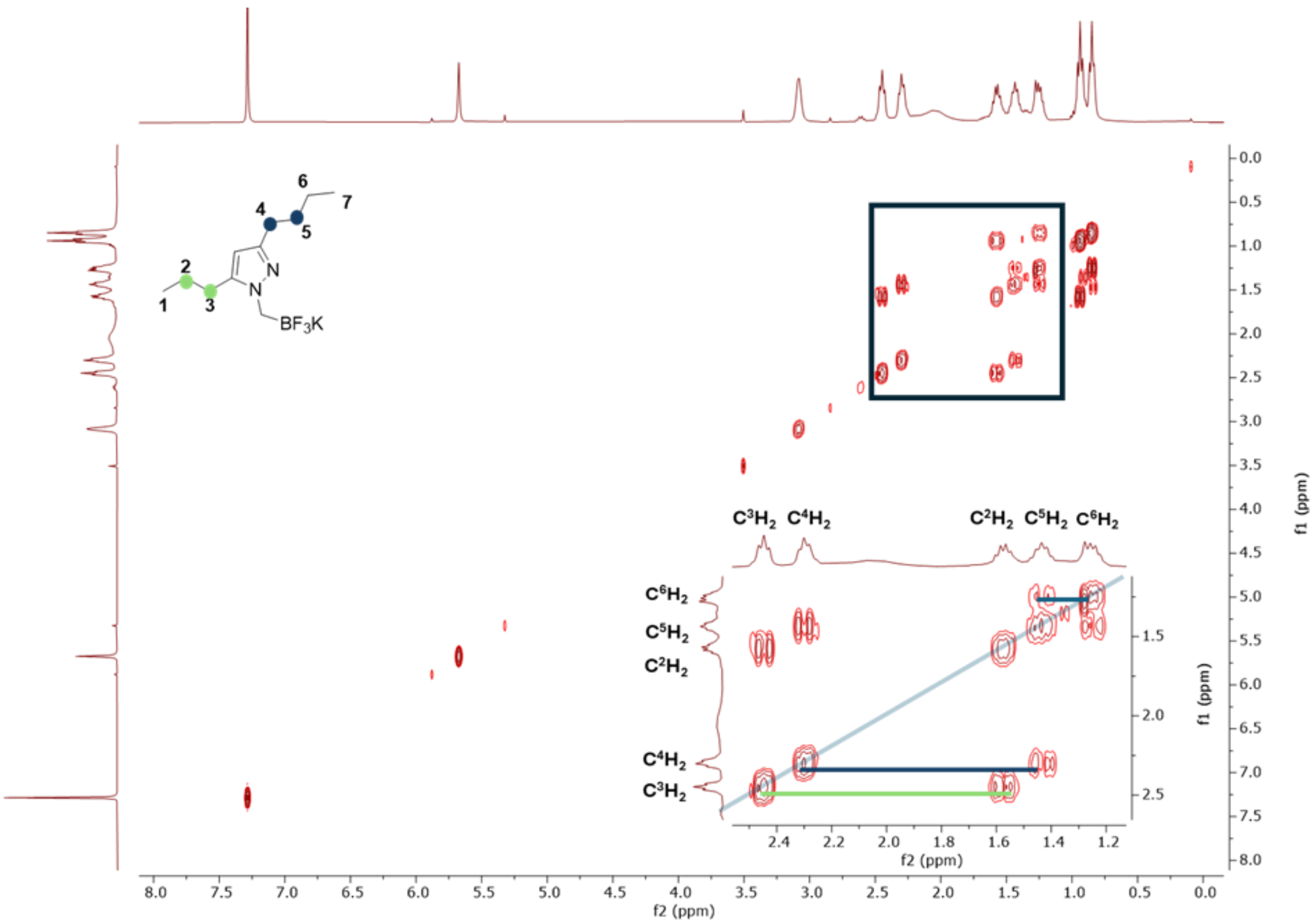

BF3K
C3H2 C4H2
C2H2 C5H2 C6H2
C6H2
C5H2
C2H2
C4H2
C3H2
f2 (ppm)
f1 (ppm)

NOESY spectroscopy confirmed regiochemistry of **26**:

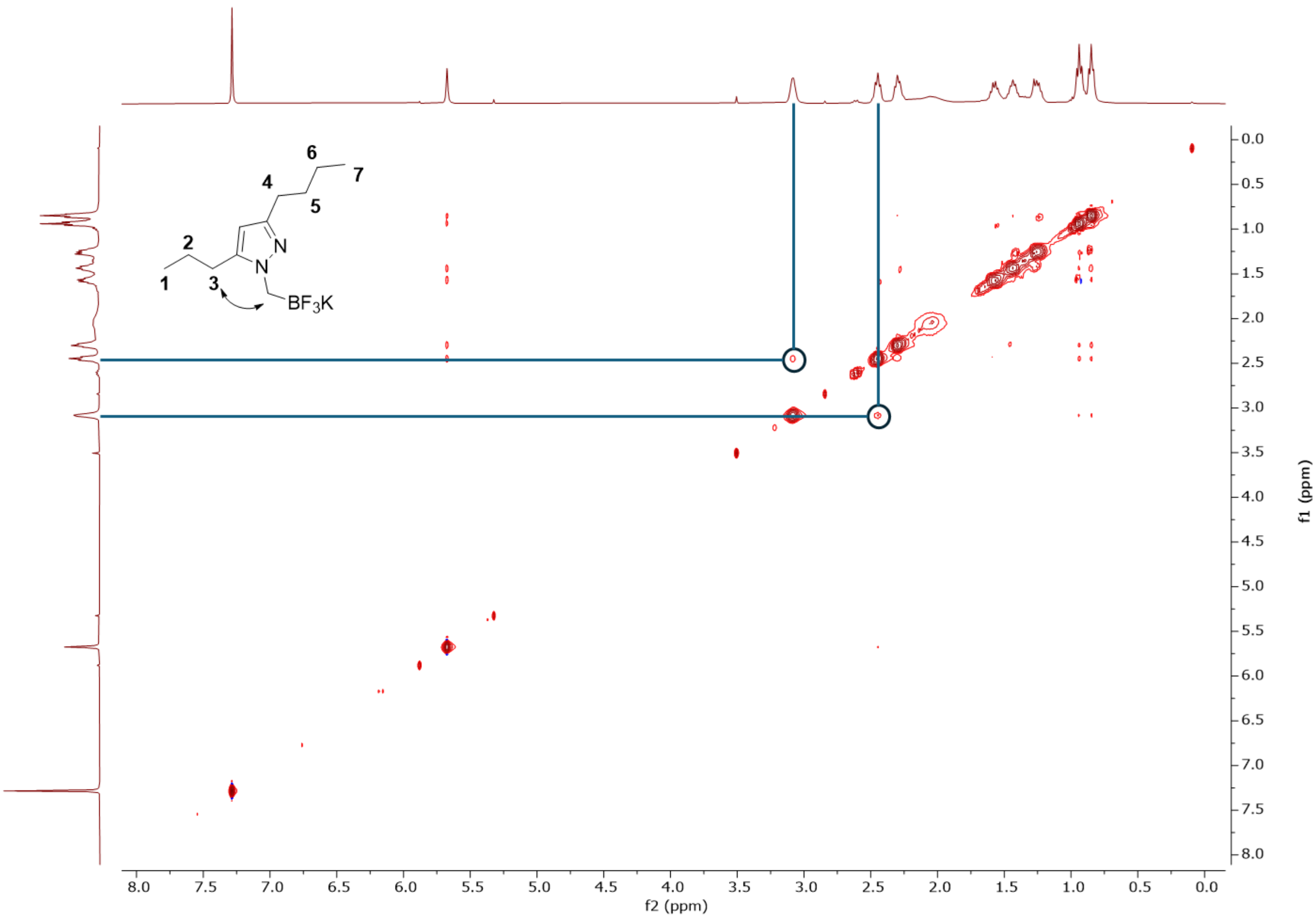

^{1}H-^{1}H COSY shows the connectivity of the ^{n}Pr and ^{n}Bu chains in pyrazole **27**:

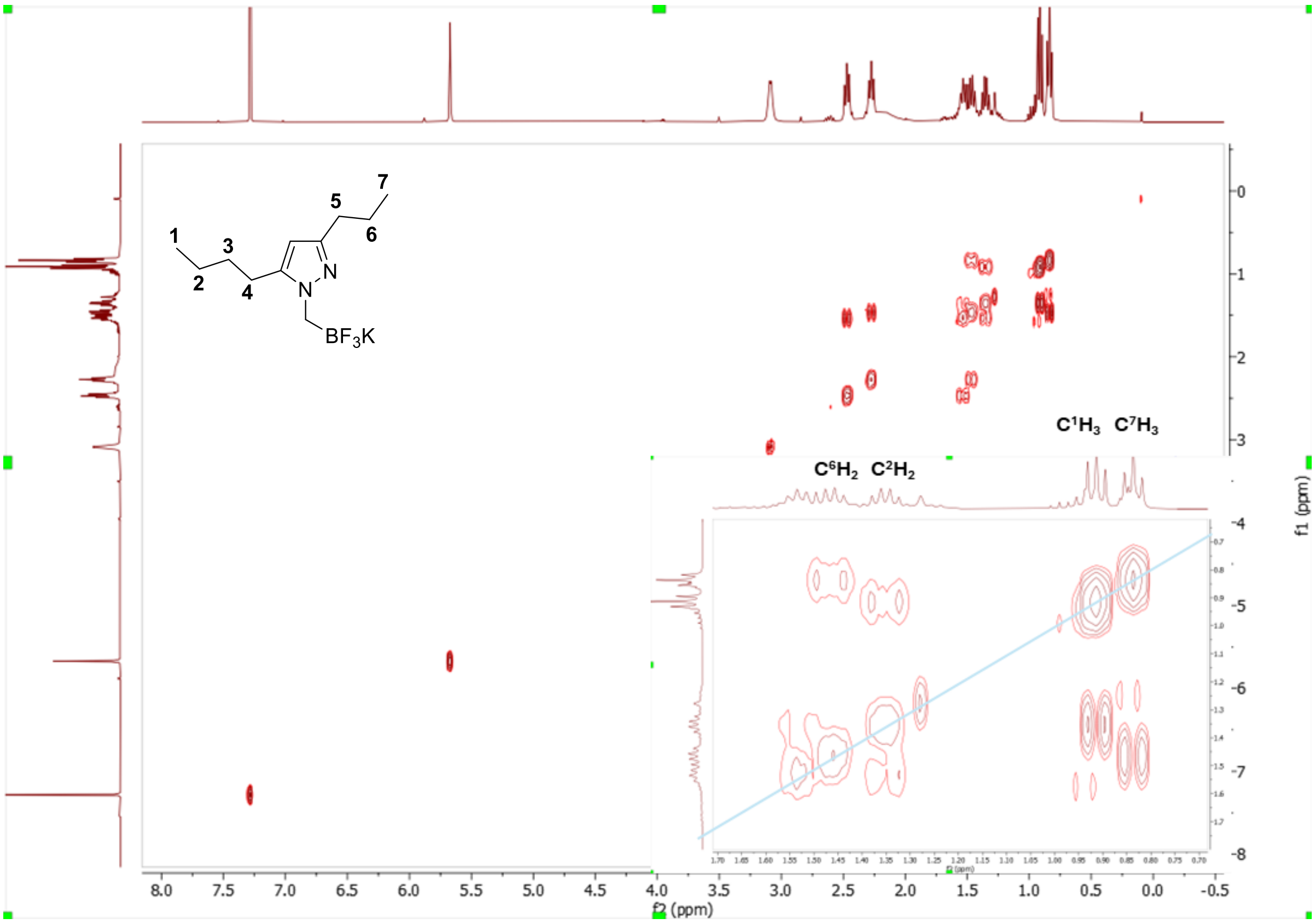

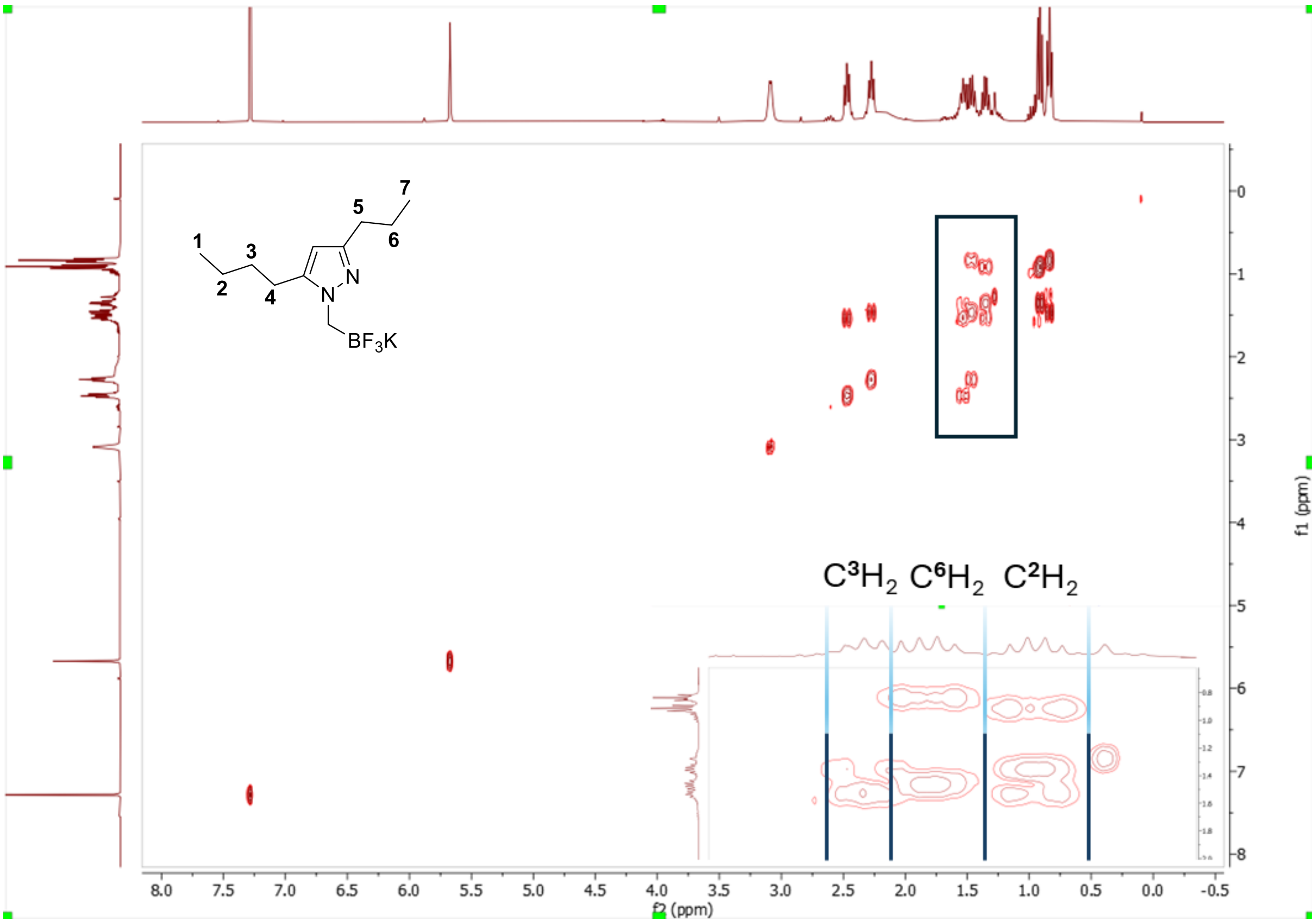

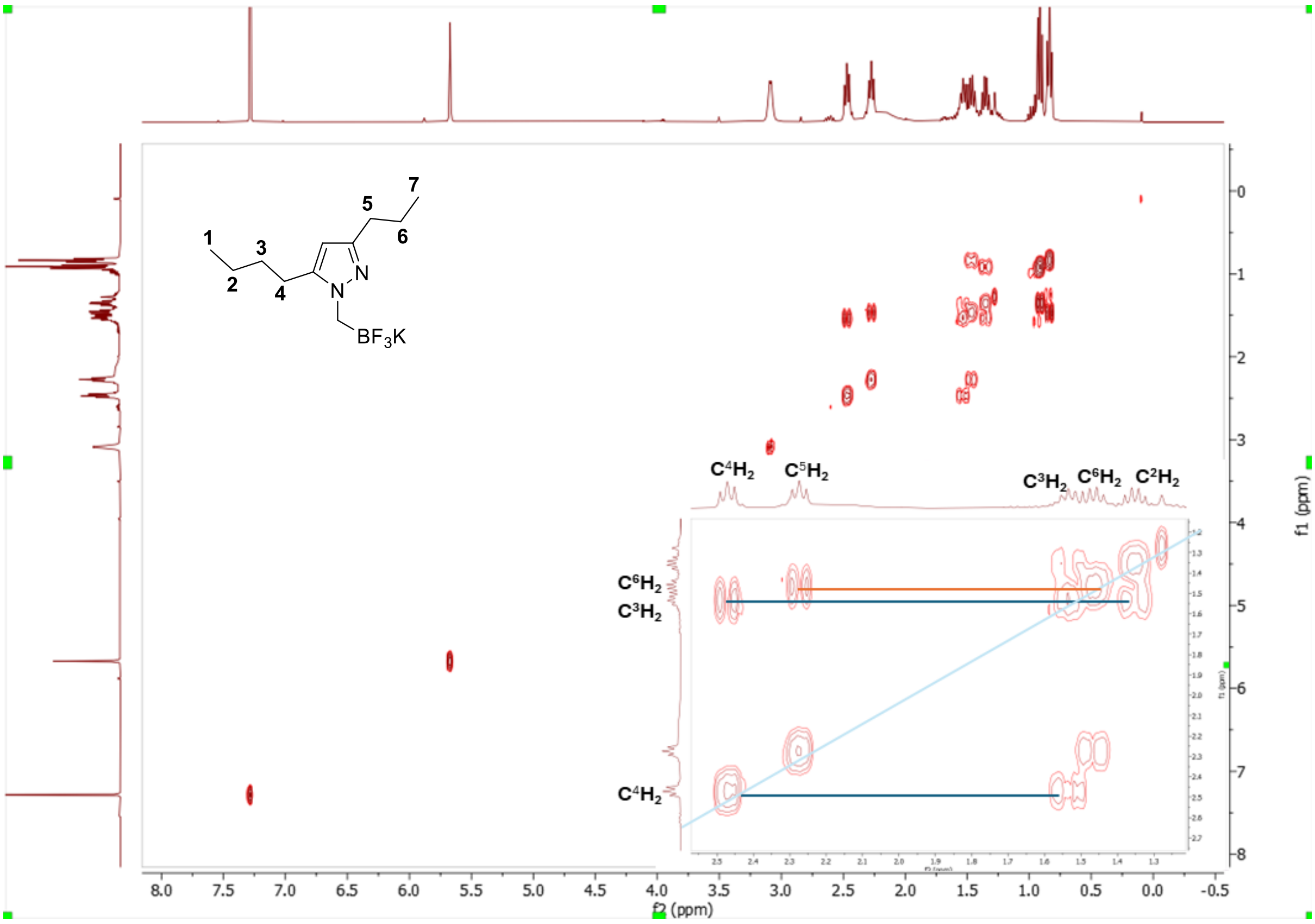

NOESY spectroscopy confirmed regiochemistry of **27**:

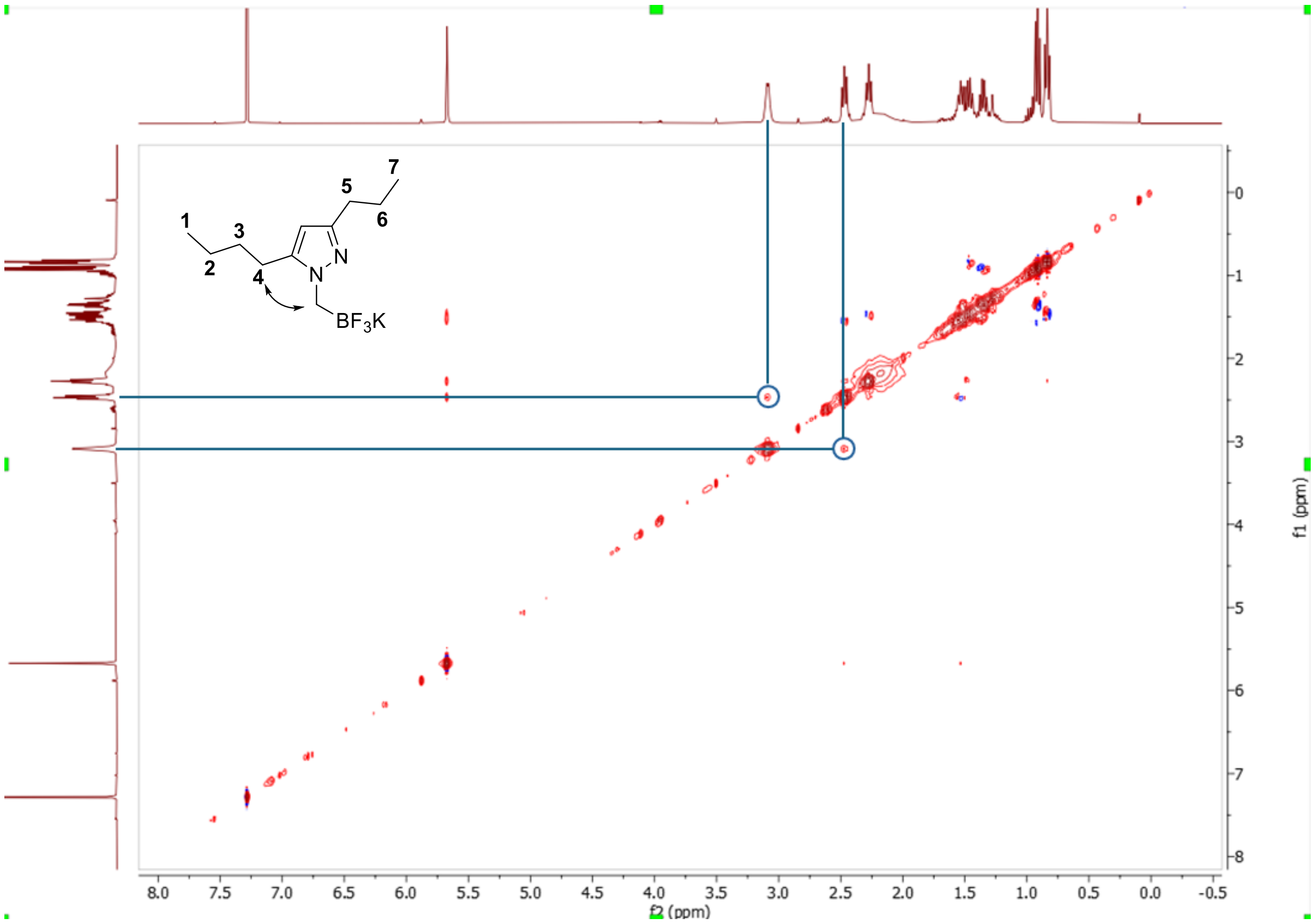

X-ray Crystallography

Figure S1. Potassium 1-trifluoro(*N*-ethylhydrazino)borate (**2**). Thermal ellipsoids shown at the 50% probability level.

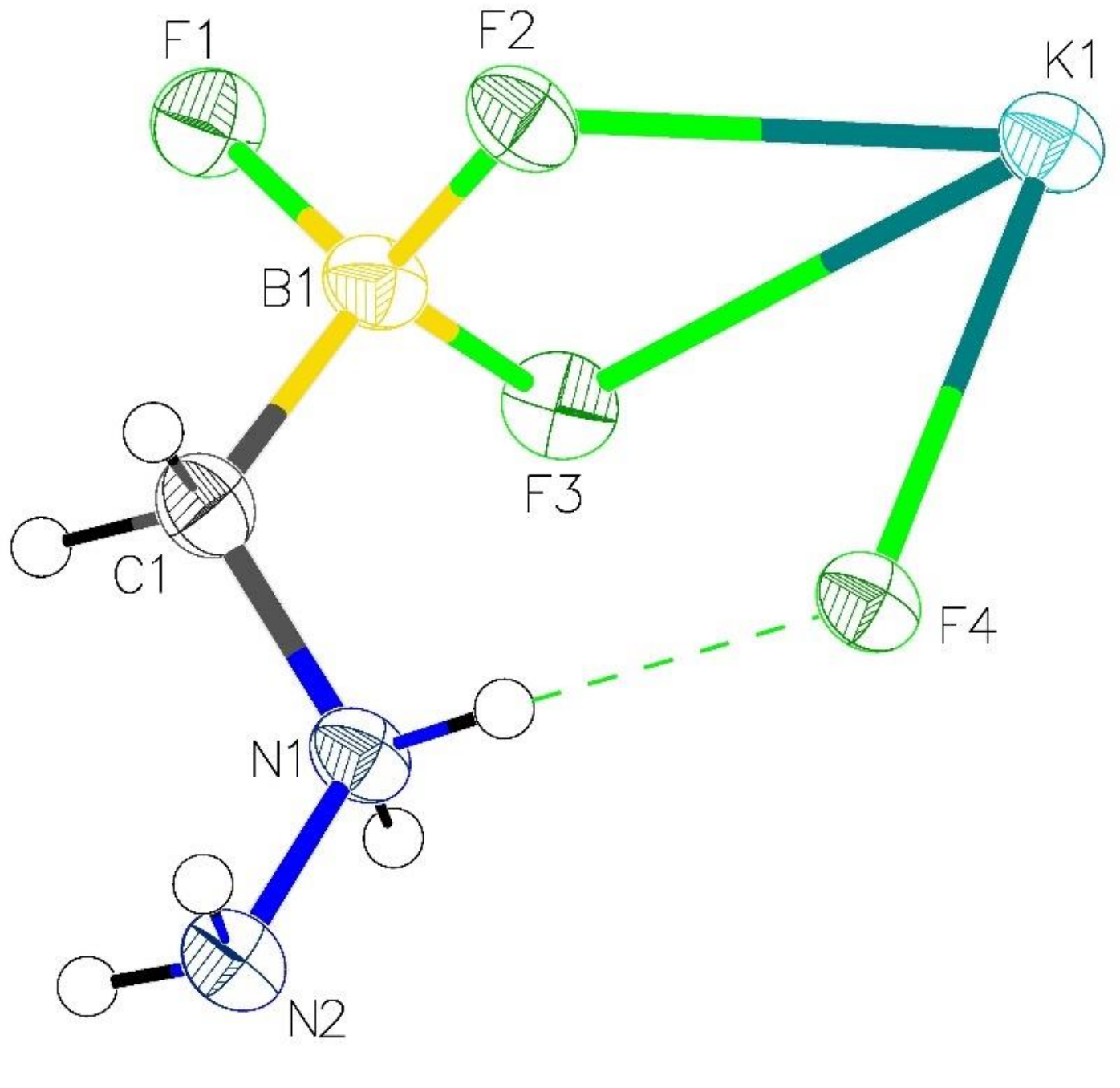

Experimental. A suitable colourless needle-shaped crystal 0.13×0.04×0.02 mm^3 was selected and mounted on a MiTeGen micromount in NVH oil on an XtaLAB Synergy R, HyPix-Arc 100 diffractometer. The crystal was kept at a steady T = 100.00(10) K during data collection. The structure was solved with the ShelXT 2018/2 (Sheldrick, 2018) structure solution program using the Intrinsic Phasing solution method and by using Olex2 (Dolomanov et al., 2009) as the graphical interface. The model was refined with version 2019/3 of ShelXL 2019/3 (Sheldrick, 2015) using Least Squares minimisation.

Crystal Data. $C_2H_{12}B_2F_7KN_4$, M_r = 285.88, monoclinic, $C2/c$ (No. 15), a = 17.7174(8) Å, b = 7.1701(3) Å, c = 7.7973(3) Å, β = 98.937(4)°, $\alpha = \gamma$ = 90°, V = 978.51(7) Å^3, T = 100.00(10) K, Z = 4, Z' = 0.5, μ(Cu K$_\alpha$) = 5.661, 4900 reflections measured, 1003 unique (R_{int} = 0.0932) which were used in all calculations. The final wR_2 was 0.1761 (all data) and R_1 was 0.0656 (I > 2(I)).

Formula	$C_2H_{12}B_2F_7KN_4$
$D_{calc.}$/ g cm^{-3}	1.941
μ/mm^{-1}	5.661
Formula Weight	285.88
Colour	colourless
Shape	needle
Size/mm^3	0.13×0.04×0.02
T/K	100.00(10)
Crystal System	monoclinic
Space Group	$C2/c$
a/Å	17.7174(8)
b/Å	7.1701(3)
c/Å	7.7973(3)
α/°	90
β/°	98.937(4)
γ/°	90
V/Å^3	978.51(7)
Z	4
Z'	0.5
Wavelength/Å	1.54184
Radiation type	Cu K$_\alpha$
Θ_{min}/°	6.672
Θ_{max}/°	77.741
Measured Refl.	4900
Independent Refl.	1003
Reflections with I > 2(I)	888
R_{int}	0.0932
Parameters	90
Restraints	0
Largest Peak	0.840
Deepest Hole	-0.699
GooF	1.043
wR_2 (all data)	0.1761
wR_2	0.1715
R_1 (all data)	0.0694
R_1	0.0656

Figure S2. Trifluoro((3-methyl-4,5,6,7-tetrahydro-2*H*-indazol-1-ium-2-yl)methyl)borate (**19 major**). Thermal ellipsoids shown at the 50% probability level.

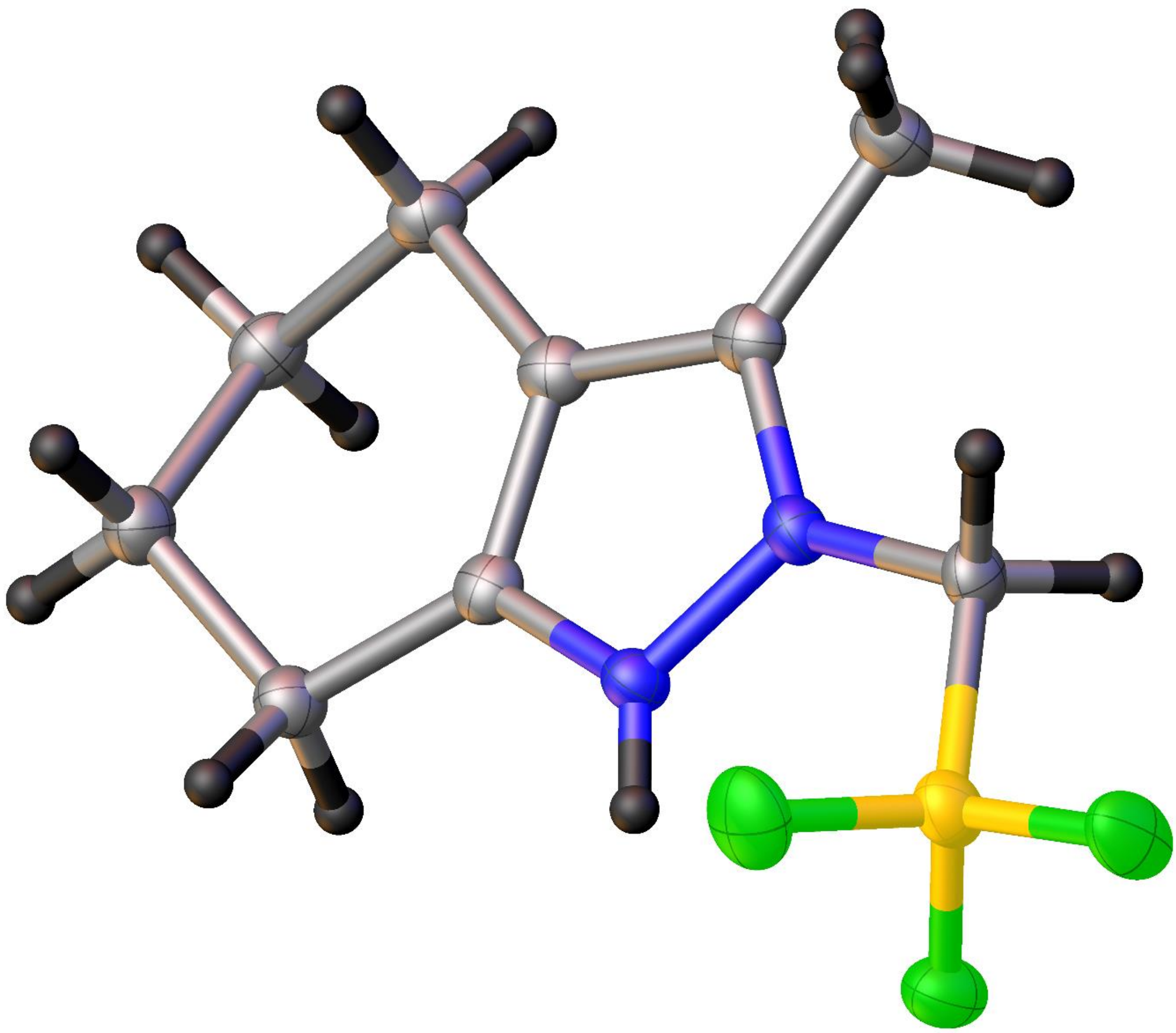

Experimental. A suitable colourless block shaped crystal 0.23×0.14×0.07 mm³ was selected and mounted on a MiTeGen microloop in fomblin oil on an XtaLAB Synergy R, HyPix-Arc 100 diffractometer. The crystal was kept at a steady T = 100.05(10) K during data collection. The structure was solved with the ShelXT 2018/2 (Sheldrick, 2018) structure solution program using the Intrinsic Phasing solution method and by using Olex2 (Dolomanov et al., 2009) as the graphical interface. The model was refined with version 2019/3 of ShelXL 2019/3 (Sheldrick, 2015) using Least Squares minimisation.

Crystal Data. $C_9H_{14}BF_3N_2$, M_r = 218.03, monoclinic, $P2_1/c$ (No. 14), a = 8.7162(2) Å, b = 15.3890(3) Å, c = 8.2319(2) Å, β = 114.597(3)°, $\alpha = \gamma$ = 90°, V = 1003.98(4) Å³, T = 100.05(10) K, Z = 4, Z' = 1, μ(Cu K$_\alpha$) = 1.076, 6264 reflections measured, 1965 unique (R_{int} = 0.0180) which were used in all calculations. The final wR_2 was 0.0903 (all data) and R_1 was 0.0349 (I > 2(I)).

Formula	$C_9H_{14}BF_3N_2$
$D_{calc.}$/ g cm^{-3}	1.442
μ/mm^{-1}	1.076
Formula Weight	218.03
Colour	colourless
Shape	block
Size/mm³	0.23×0.14×0.07
T/K	100.05(10)
Crystal System	monoclinic
Space Group	$P2_1/c$
a/Å	8.7162(2)
b/Å	15.3890(3)
c/Å	8.2319(2)
α/°	90
β/°	114.597(3)
γ/°	90
V/Å³	1003.98(4)
Z	4
Z'	1
Wavelength/Å	1.54184
Radiation type	Cu K$_\alpha$
Θ_{min}/°	5.582
Θ_{max}/°	77.709
Measured Refl.	6264
Independent Refl.	1965
Reflections with I > 2(I)	1825
R_{int}	0.0180
Parameters	141
Restraints	0
Largest Peak	0.305
Deepest Hole	-0.220
GooF	1.041
wR_2 (all data)	0.0903
wR_2	0.0888
R_1 (all data)	0.0371
R_1	0.0349

Figure S3. Trifluoro((3-methyl-5,6-dihydrocyclopenta[c]pyrazol-2-ium-1(*4H*)-yl)methyl)borate (**20 major**). Thermal ellipsoids shown at the 50% probability level.

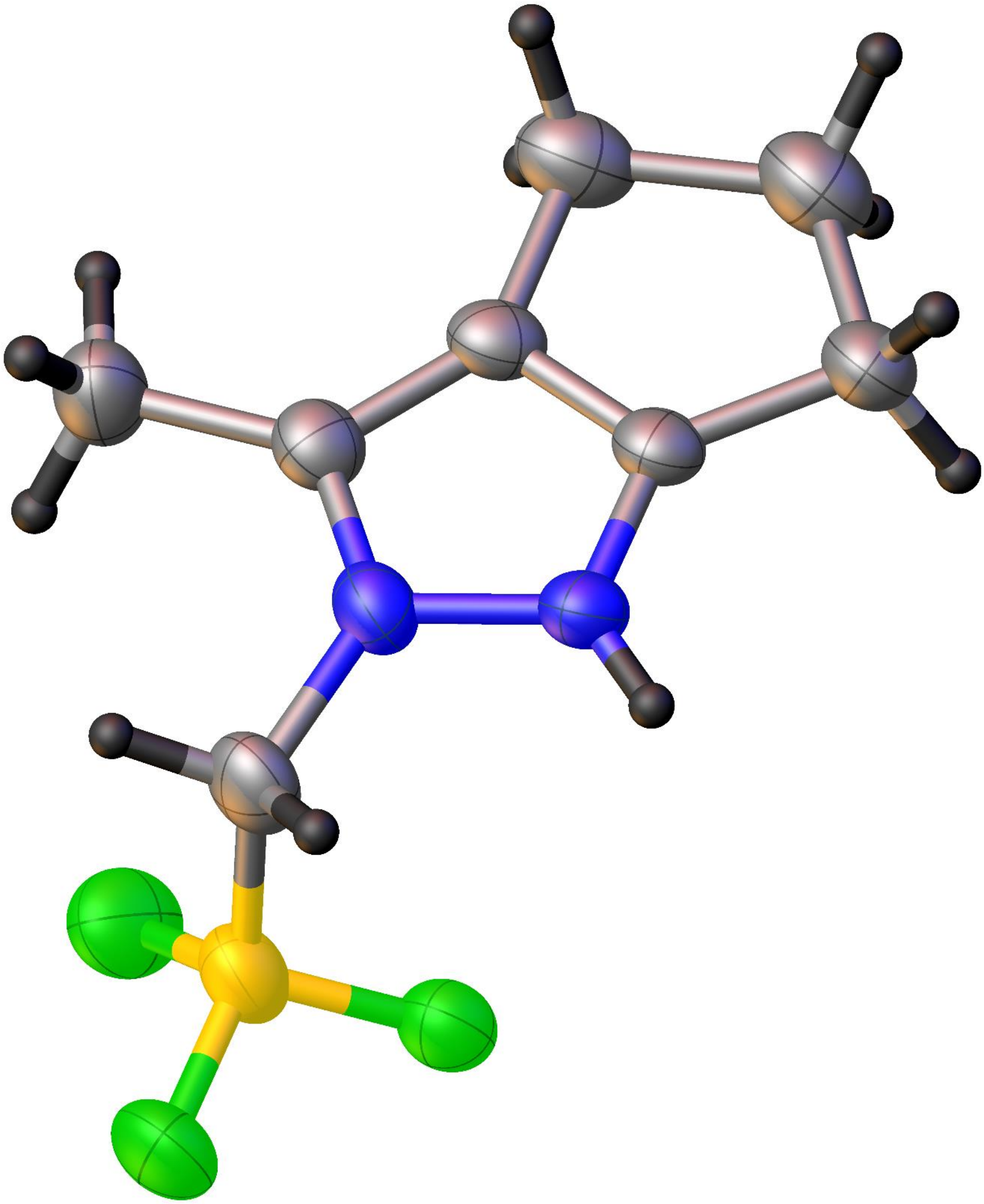

Experimental. Single colourless block-shaped crystals of OJH489r C91 minor were obtained by recrystallisation from $CDCl_3$ in an NMR tube. A suitable crystal 0.09×0.06×0.05 mm^3 was selected and mounted on a MiTeGen microloop in fomblin oil on an XtaLAB Synergy R, HyPix-Arc 100 diffractometer. The crystal was kept at a steady T = 100.00(10) K during data collection. The structure was solved with the ShelXT (Sheldrick, 2015) structure solution program using the Intrinsic Phasing solution method and by using Olex2 (Dolomanov et al., 2009) as the graphical interface. The model was refined with version 2019/3 of ShelXL 2019/3 (Sheldrick, 2015) using Least Squares minimisation.

Crystal Data. $C_8H_{12}BF_3N_2$, M_r = 204.01, monoclinic, $P2_1/c$ (No. 14), a = 8.5066(6) Å, b = 14.7589(11) Å, c = 8.0178(5) Å, β = 109.705(8)°, $\alpha = \gamma$ = 90°, V = 947.68(12) Å^3, T = 100.00(10) K, Z = 4, Z' = 1, μ(Cu K_α) = 1.101, 5980 reflections measured, 1868 unique (R_{int} = 0.0329) which were used in all calculations. The final wR_2 was 0.2203 (all data) and R_1 was 0.0837 (I > 2(I)).

Formula	$C_8H_{12}BF_3N_2$
$D_{calc.}$/ g cm^{-3}	1.430
μ/mm^{-1}	1.101
Formula Weight	204.01
Colour	colourless
Shape	block
Size/mm^3	0.09×0.06×0.05
T/K	100.00(10)
Crystal System	monoclinic
Space Group	$P2_1/c$
a/Å	8.5066(6)
b/Å	14.7589(11)
c/Å	8.0178(5)
α/°	90
β/°	109.705(8)
γ/°	90
V/Å^3	947.68(12)
Z	4
Z'	1
Wavelength/Å	1.54184
Radiation type	Cu K_α
Θ_{min}/°	5.524
Θ_{max}/°	77.637
Measured Refl.	5980
Independent Refl.	1868
Reflections with I > 2(I)	1606
R_{int}	0.0329
Parameters	132
Restraints	0
Largest Peak	0.842
Deepest Hole	-0.256
GooF	1.149
wR_2 (all data)	0.2203
wR_2	0.2124
R_1 (all data)	0.0946
R_1	0.0837

Figure S4. Trifluoro((3-methyl-5,6-dihydrocyclopenta[c]pyrazol-1-ium-2(4*H*)-yl)methyl)borate (**20 minor**). Thermal ellipsoids shown at the 50% probability level.

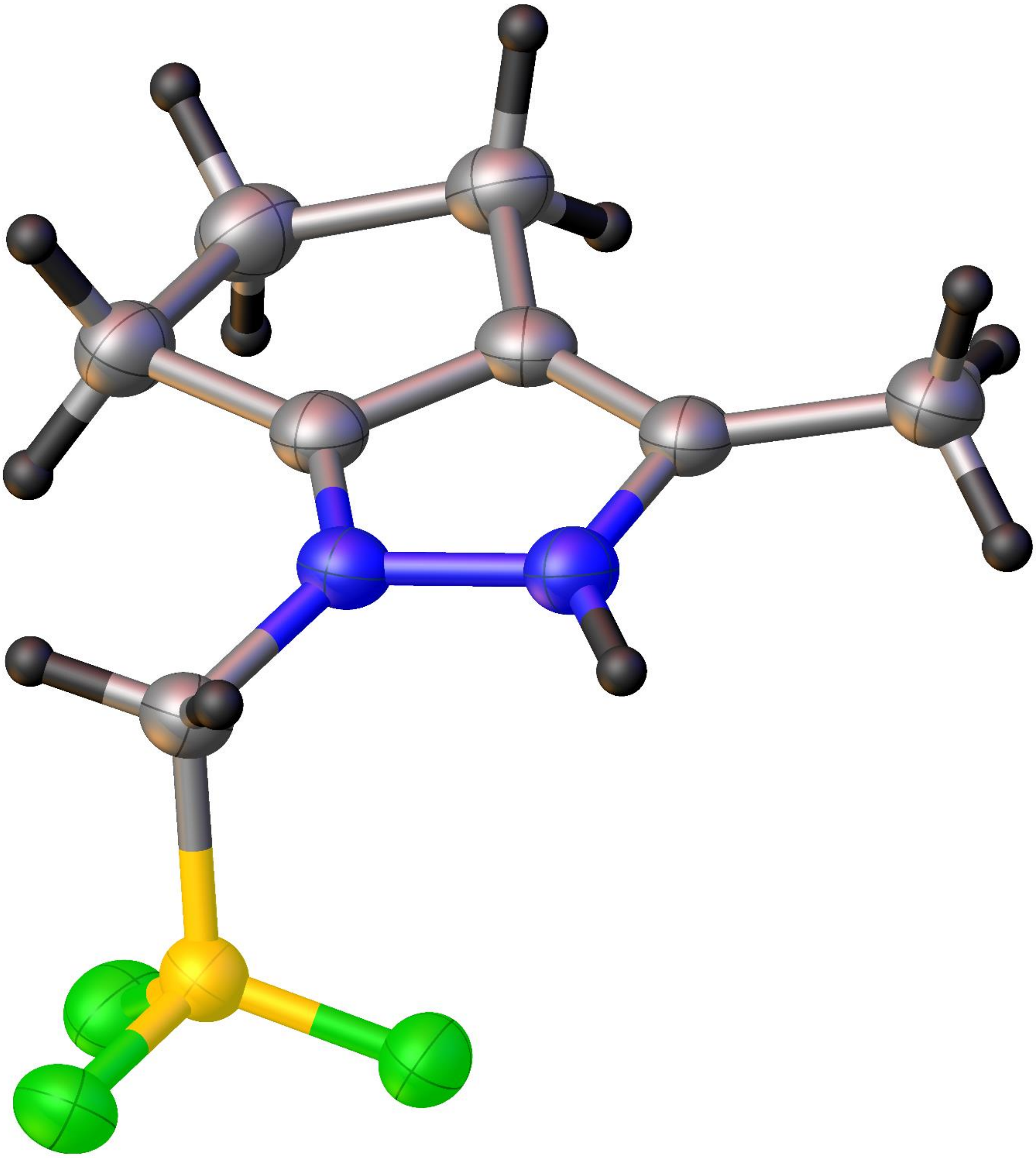

Experimental. Single colourless block-shaped crystals of OJH490r C91 were obtained by recrystallisation from $CDCl_3$ in an NMR tube. A suitable crystal 0.27×0.10×0.06 mm^3 was selected and mounted on a MiTeGen microloop in fomblin oil on an XtaLAB Synergy R, HyPix-Arc 100 diffractometer. The crystal was kept at a steady T = 100.00(10) K during data collection. The structure was solved with the ShelXT (Sheldrick, 2015) structure solution program using the Intrinsic Phasing solution method and by using Olex2 (Dolomanov et al., 2009) as the graphical interface. The model was refined with version 2019/3 of ShelXL 2019/3 (Sheldrick, 2015) using Least Squares minimisation.

Crystal Data. $C_8H_{12}BF_3N_2$, M_r = 204.01, monoclinic, $P2_1/n$ (No. 14), a = 7.8155(5) Å, b = 8.7793(7) Å, c = 13.5683(10) Å, β = 95.761(7)°, $\alpha = \gamma$ = 90°, V = 926.29(12) Å^3, T = 100.00(10) K, Z = 4, Z' = 1, μ(Cu K$_\alpha$) = 1.126, 5562 reflections measured, 1799 unique (R_{int} = 0.0307) which were used in all calculations. The final wR_2 was 0.1543 (all data) and R_1 was 0.0506 (I > 2(I)).

Formula	$C_8H_{12}BF_3N_2$
$D_{calc.}$/ g cm^{-3}	1.463
μ/mm^{-1}	1.126
Formula Weight	204.01
Colour	colourless
Shape	block
Size/mm^3	0.27×0.10×0.06
T/K	100.00(10)
Crystal System	monoclinic
Space Group	$P2_1/n$
a/Å	7.8155(5)
b/Å	8.7793(7)
c/Å	13.5683(10)
α/°	90
β/°	95.761(7)
γ/°	90
V/Å^3	926.29(12)
Z	4
Z'	1
Wavelength/Å	1.54184
Radiation type	Cu K$_\alpha$
Θ_{min}/°	6.013
Θ_{max}/°	77.839
Measured Refl.	5562
Independent Refl.	1799
Reflections with I > 2(I)	1542
R_{int}	0.0307
Parameters	132
Restraints	0
Largest Peak	0.343
Deepest Hole	-0.258
GooF	1.098
wR_2 (all data)	0.1543
wR_2	0.1481
R_1 (all data)	0.0584
R_1	0.0506

1. Dolomanov, O.V., Bourhis, L.J., Gildea, R.J, Howard, J.A.K. & Puschmann, H. (2009), J. Appl. Cryst. 42, 339-341.
2. Sheldrick, G.M. (2015). Acta Cryst. A71, 3-8.
3. Sheldrick, G.M. (2008). Acta Cryst. A64, 112-122.
